# Supplementary material for: Effectiveness of integrated chronic care models for cardiometabolic multimorbidity in sub-Saharan Africa: a systematic review and meta-analysis
Source: BMJ Open. 2023 Jun 27;13(6):e073652. doi: 10.1136/bmjopen-2023-073652 (PMC10410889; doi:10.1136/bmjopen-2023-073652)
Supplement: Supplementary data [file bmjopen-2023-073652supp001.pdf]

## List of online supplementary files

**Online supplementary file 1: Classification of interventions**

| Intervention component | Feature |
|---|---|
| Self-management support | • Self-care interventions based on technological aid<br>• Self-help groups<br>• Family-oriented supports<br>• Motivational support<br>• Behaviour therapy |
| Delivery system design | • Practice team functioning<br>• Patient care planning and follow-up<br>• Coordination between primary care and specialist services |
| Decision support | • Practice clinical guidelines<br>• Provider education<br>• Involvement of specialists in improving primary care |
| Clinical information system | • Disease registry<br>• Reminders to providers<br>• Feedback to providers |
| Healthcare organization | • Organizational goals and resources for chronic disease management<br>• Quality improvement strategies<br>• Incentives |
| Community linkages | • Linking patients to outside resources<br>• Activities with community-based organizations<br>• Professionals working out in the community |

**Supplementary file 2: Search strategies for electronic databases**

1. **PubMED (Medline) Search strategy**

**#1** ("elevated blood pressure"[MeSH Terms] OR "high blood pressure"[MeSH Terms] OR "raised blood pressure"[MeSH Terms] OR "diastolic blood pressure"[MeSH Terms] OR "systolic blood pressure"[MeSH Terms] OR "arterial blood pressure"[MeSH Terms] OR "uncontrolled blood pressure"[MeSH Terms] OR "uncontrolled hypertension"[All Fields] OR "elevated blood pressure"[All Fields] OR "high blood pressure"[All Fields] OR "raised blood pressure"[All Fields] OR "diastolic blood pressure"[All Fields] OR "systolic blood pressure"[All Fields] OR "arterial blood pressure"[All Fields] OR "uncontrolled blood pressure"[All Fields] OR "uncontrolled hypertension"[All Fields])

**#2** ("diabetes mellitus"[MeSH Terms] OR "diabetes mellitus, type 2"[MeSH Terms] OR "diabetes type 2"[MeSH Terms] OR "T2DM"[MeSH Terms] OR "diabetes type II"[MeSH Terms] OR "diabetes"[MeSH Terms] OR "glucose intolerance"[MeSH Terms] OR "insulin resistance"[MeSH Terms] OR "hyperglycemia"[MeSH Terms] OR "hyperglycaemia"[MeSH Terms] OR "hypoglycaemia"[MeSH Terms] OR "hypoglycaemia"[MeSH Terms] OR "high blood sugar"[MeSH Terms] OR "elevated blood sugar"[MeSH Terms] OR "raised blood sugar"[MeSH Terms] OR "high blood glucose"[MeSH Terms] OR "elevated blood glucose"[MeSH Terms] OR "raised blood glucose"[MeSH Terms] OR "diabetes mellitus"[All Fields] OR "diabetes mellitus, type 2"[All Fields] OR "diabetes type 2"[All Fields] OR "T2DM"[All Fields] OR "diabetes type II"[All Fields] OR "diabetes"[All Fields] OR "glucose intolerance"[All Fields] OR "insulin resistance"[All Fields] OR "hyperglycemia"[All Fields] OR "hyperglycaemia"[All Fields] OR "hypoglycemia"[All Fields] OR "hypoglycaemia"[All Fields] OR "high blood sugar"[All Fields] OR "elevated blood sugar"[All Fields] OR "raised blood sugar"[All Fields] OR "high blood glucose"[All Fields] OR "elevated blood glucose"[All Fields] OR "raised blood glucose"[All Fields])



Otieno P, *et al. BMJ Open* 2023; 13:e073652. doi: 10.1136/bmjopen-2023-073652

**#3** ("stroke"[MeSH Terms] OR "transient ischemic attack"[MeSH Terms] OR "ischemic attack"[MeSH Terms] OR "angina"[MeSH Terms] OR "angina pectoris"[MeSH Terms] OR "heart attack"[MeSH Terms] OR "ischemic heart diseases "[MeSH Terms] OR "transient ischaemic attack"[MeSH Terms] OR "coronary heart disease"[MeSH Terms] OR "coronary disease"[MeSH Terms] OR "heart failure"[MeSH Terms] OR "peripheral vascular disease"[MeSH Terms] OR "Peripheral Vascular diseases"[MeSH Terms] OR "atrial fibrillation"[MeSH Terms] OR "cardiovascular disease"[MeSH Terms] OR "heart disease"[MeSH Terms] OR "stroke"[All Fields] OR "transient ischemic attack"[All Fields] OR "ischemic attack"[All Fields] OR "angina"[All Fields] OR "angina pectoris"[All Fields] OR "heart attack"[All Fields] OR "ischemic heart diseases "[All Fields] OR "transient ischaemic attack"[All Fields] OR "coronary heart disease"[All Fields] OR "coronary disease"[All Fields] OR "heart failure"[All Fields] OR "peripheral vascular disease"[All Fields] OR "Peripheral Vascular diseases"[All Fields] OR "atrial fibrillation"[All Fields] OR "cardiovascular disease"[All Fields] OR "heart disease"[All Fields])

**#4** ("Hypercholesterolemia"[MeSH Terms] OR "Dyslipidemias"[MeSH Terms] OR "blood lipid"[MeSH Terms] OR "Cholesterol"[MeSH Terms] OR "high cholesterol"[MeSH Terms] OR "elevated cholesterol"[MeSH Terms] OR "raised cholesterol"[MeSH Terms] OR "low density lipoprotein "[MeSH Terms] OR "high density lipoprotein"[MeSH Terms] OR "Dyslipidemia"[MeSH Terms] OR "Dyslipidaemia"[MeSH Terms] OR "Hypercholesterolemia"[MeSH Terms] OR "hypercholesterolaemia "[MeSH Terms] OR "hypercholesterolimia "[MeSH Terms] OR "Hypertriglyceridemia"[MeSH Terms] OR "Hypertriglyceridemia"[MeSH Terms] OR "Hypertriglyceridaemia"[MeSH Terms] OR "Hyperlipidemia"[MeSH Terms] OR "Hyperlipidemias"[MeSH Terms] OR "Hyperlipidaemia"[MeSH Terms] OR "LDL"[MeSH Terms] OR "HDL"[MeSH Terms] OR "Hypercholesterolemia"[All Fields] OR "Dyslipidemias"[All Fields] OR "blood lipid"[All Fields] OR "Cholesterol"[All Fields] OR "high cholesterol"[All Fields] OR "elevated cholesterol"[All Fields] OR "raised cholesterol"[All Fields] OR "low density lipoprotein "[All Fields] OR "high density lipoprotein"[All Fields] OR "Dyslipidemia"[All Fields] OR "Dyslipidaemia"[All Fields] OR "Hypercholesterolemia"[All Fields] OR "hypercholesterolaemia "[All Fields] OR "hypercholesterolimia "[All Fields] OR "Hypertriglyceridemia"[All Fields] OR "Hypertriglyceridemia"[All Fields] OR "Hypertriglyceridaemia"[All Fields] OR "Hyperlipidemia"[All Fields] OR "Hyperlipidemias"[All Fields] OR "Hyperlipidaemia"[All Fields] OR "LDL"[All Fields] OR "HDL"[All Fields])

**#5** ("Comorbid"[All Fields] OR "co-morbid"[All Fields] OR "co-morbidity"[All Fields] OR "Multimorbidity"[All Fields] OR "multi-morbid"[All Fields] OR "multi morbidity"[All Fields] OR "Multimorbidity"[All Fields] OR "Comorbidity"[All Fields] OR "multi-disease"[All Fields] OR "Multidisease"[All Fields] OR multi disease[All Fields] OR "multiple condition"[All Fields] OR "multi-condition"[All Fields] OR "multi condition"[All Fields] OR "multiple illness"[All Fields] OR "multi-illness"[All Fields] OR "multi illness"[All Fields] OR "multiple syndrome"[All Fields] OR "multi-syndrome"[All Fields] OR "multi syndrome"[All Fields] OR "concurrent condition"[All Fields] OR "concurrent illness"[All Fields] OR "concurrent disease"[All Fields] OR "co-existing disease"[All Fields] OR "coexisting disease"[All Fields] OR "co-existing illness"[All Fields] OR "coexisting illness"[All Fields] OR "co-existing syndrome"[All Fields] OR "coexisting syndrome"[All Fields] OR "co-existing condition"[All Fields] OR "coexisting condition"[All Fields] OR "co-occurring disease"[All Fields] OR "co-occuring disease"[All Fields] OR "co-occuring disease"[All Fields] OR "co-occurring illness"[All Fields] OR "co occurring illness"[All Fields] OR "cooccurring illness"[All Fields] OR "co-occurring syndrome"[All Fields] OR "co occurring syndrome"[All Fields] OR "cooccurring syndrome"[All Fields] OR "co-occurring condition"[All Fields] OR "co occurring condition"[All Fields] OR "cooccurring condition"[All Fields])

**#6** ("Wagner model"[All Fields] OR "Wagner* model*"[All Fields] OR "Wagner* chronic care model*"[All Fields] OR "Wagner chronic care model"[All Fields] OR "chronic care model*"[All Fields] OR "model "[All Fields] OR "collaborative care"[All Fields] OR "chronic care framework"[All Fields] OR "chronic disease care"[All Fields] OR "chronic illness care"[All Fields] OR care model[All Fields] OR "model care"[All Fields] OR "Wagner* model*"[All Fields] OR "Wagner* chronic care model*"[All Fields] OR "Wagner chronic care model"[All Fields] OR "theory"[All Fields] OR "concept"[All Fields] OR "framework"[All Fields] OR "model"[All Fields] OR "programme"[All Fields] OR "approach"[All Fields] OR "clinical pathway"[All Fields] OR "care pathway"[All Fields] OR "critical path"[All Fields] OR "vertical integration"[All Fields] OR "virtual integration"[All Fields] OR "physician system integration"[All Fields] OR "provider system integration"[All Fields] OR "functional



Otieno P, *et al. BMJ Open* 2023; 13:e073652. doi: 10.1136/bmjopen-2023-073652

integration"[All Fields] OR "horizontal integration"[All Fields] OR "clinical integration"[All Fields] OR "case management"[All Fields] OR "delivery of health care, integrated"[All Fields] OR "disease management"[All Fields] OR "patient care management"[All Fields] OR "patient-centred care"[All Fields] OR "accountable care organisations"[All Fields] OR "continuity of patient care"[All Fields] OR "case management"[All Fields] OR "comprehensive health care"[All Fields] OR "delivery of health care, integrated"[All Fields] OR "managed care programmes"[All Fields] OR "patient-centred care"[All Fields] OR "care delivery"[All Fields] OR "integrated care"[All Fields] OR "comprehensive care"[All Fields] OR "care coordination"[All Fields] OR "managed care"[All Fields] OR "accountable care "[All Fields] OR "accountable care organisations"[All Fields] OR "accountable care organisation"[All Fields] OR "collaborative care"[All Fields] OR "disease management"[All Fields] OR "case-management"[All Fields] OR "case management"[All Fields] OR "shared care"[All Fields] OR "accountable care"[All Fields] OR "patient-centred"[All Fields] OR "patient centred"[All Fields] OR "person-centred"[All Fields] OR "person centred"[All Fields] OR "multidisciplinary care"[All Fields] OR "interdisciplinary care"[All Fields] OR "inter-disciplinary care"[All Fields] OR "cross disciplinary care"[All Fields] OR "cross-disciplinary care"[All Fields] OR "multiple interventions"[All Fields] OR "care chain"[All Fields] OR "care chains"[All Fields] OR "care continuity"[All Fields] OR "care continuation"[All Fields] OR "care transition"[All Fields] OR "care transitions"[All Fields] OR "chain of care"[All Fields] OR "chains of care"[All Fields] OR "continuity of care"[All Fields] OR "cross sectoral care"[All Fields] OR "delivery of health care integrated"[All Fields] OR "integrated medicine"[All Fields] OR "integrated social network"[All Fields] OR "integrated social networks"[All Fields] OR "integration of care"[All Fields] OR "intersectoral care"[All Fields] OR "intrasectoral care"[All Fields] OR "linked care"[All Fields] OR "management model"[All Fields] OR "patient care management"[All Fields] OR "seamless care"[All Fields] OR "service network"[All Fields] OR "service networks"[All Fields] OR "transition of care"[All Fields] OR "transitional care"[All Fields] OR "transmural care"[All Fields] OR "whole system thinking"[All Fields] OR "holistic care"[All Fields])

**#7** ("Angola "[All Fields] OR "Benin "[All Fields] OR "Botswana "[All Fields] OR "Burkina Faso"[All Fields] OR "Upper Volta"[All Fields] OR "Burundi "[All Fields] OR "Cameroon "[All Fields] OR "Cameroons "[All Fields] OR "Cape Verde"[All Fields] OR "Central African Republic"[All Fields] OR Chad[All Fields] OR "Comoros "[All Fields] OR "Comoro Islands"[All Fields] OR "Comores"[All Fields] OR "Mayotte "[All Fields] OR "Congo "[All Fields] OR "Zaire "[All Fields] OR "Cote d'Ivoire"[All Fields] OR "Ivory Coast"[All Fields] OR "Democratic Republic of the Congo"[All Fields] OR "Djibouti "[All Fields] OR "French Somaliland"[All Fields] OR "Eritrea "[All Fields] OR "Ethiopia "[All Fields] OR "Gabon "[All Fields] OR "Gabonese Republic"[All Fields] OR "Gambia "[All Fields] OR "Ghana "[All Fields] OR "Gold Coast"[All Fields] OR "Guinea "[All Fields] OR "Kenya "[All Fields] OR "Lesotho"[All Fields] OR "Basutoland"[All Fields] OR "Liberia "[All Fields] OR "Madagascar "[All Fields] OR "Malagasy Republic "[All Fields] OR "Malawi "[All Fields] OR "Nyasaland "[All Fields] OR "Mali "[All Fields] OR "Mauritania "[All Fields] OR "Mauritius "[All Fields] OR "Mozambique"[All Fields] OR "Namibia"[All Fields] OR "Niger"[All Fields] OR "Nigeria"[All Fields] OR "Rwanda"[All Fields] OR "Sao Tome"[All Fields] OR "Seychelles"[All Fields] OR "Senegal "[All Fields] OR "Sierra Leone"[All Fields] OR "Somalia "[All Fields] OR "South Africa"[All Fields] OR "Sudan "[All Fields] OR "Eswatini"[All Fields] OR "Tanzania"[All Fields] OR "Togo "[All Fields] OR "Togolese Republic"[All Fields] OR "Uganda"[All Fields] OR "Zambia"[All Fields] OR "Zimbabwe "[All Fields] OR "Rhodesia"[All Fields] OR "Sub-Saharan Africa"[All Fields] OR "Africa South of the Sahara"[All Fields])

**#8** ((#1 OR #2 OR #3 OR #4 OR #5 ) AND #6 AND #7) AND Restricted (1999-2022, RCTs)

**2. Web of Science Search Strategy**

**#1** elevated blood pressure OR high blood pressure OR raised blood pressure OR diastolic blood pressure OR systolic blood pressure OR arterial blood pressure OR uncontrolled blood pressure OR uncontrolled hypertension OR elevated blood pressure OR high blood pressure OR raised blood pressure OR diastolic blood pressure OR systolic blood pressure OR arterial blood pressure OR uncontrolled blood pressure OR uncontrolled hypertension

**#2** diabetes mellitus OR diabetes mellitus, type 2 OR diabetes type 2 OR T2DM OR diabetes type II OR diabetes OR glucose intolerance OR insulin resistance OR hyperglycemia OR hyperglycaemia OR hypoglycaemia

OR hypoglycaemia OR high blood sugar OR elevated blood sugar OR raised blood sugar OR high blood glucose OR elevated blood glucose OR raised blood glucose OR diabetes mellitus OR diabetes mellitus, type 2 OR diabetes type 2 OR T2DM OR diabetes type II OR diabetes OR glucose intolerance OR insulin resistance OR hyperglycemia OR hyperglycaemia OR hypoglycemia OR hypoglycaemia OR high blood sugar OR elevated blood sugar OR raised blood sugar OR high blood glucose OR elevated blood glucose OR raised blood glucose

**#3** stroke OR transient ischemic attack OR ischemic attack OR angina OR angina pectoris OR heart attack OR ischemic heart diseases OR transient ischaemic attack OR coronary heart disease OR coronary disease OR heart failure OR peripheral vascular disease OR Peripheral Vascular diseases OR atrial fibrillation OR cardiovascular disease OR heart disease OR stroke OR transient ischemic attack OR ischemic attack OR angina OR angina pectoris OR heart attack OR ischemic heart diseases OR transient ischaemic attack OR coronary heart disease OR coronary disease OR heart failure OR peripheral vascular disease OR Peripheral Vascular diseases OR atrial fibrillation OR cardiovascular disease OR heart disease

**#4** Hypercholesterolemia OR Dyslipidemias OR blood lipid OR Cholesterol OR high cholesterol OR elevated cholesterol OR raised cholesterol OR low density lipoprotein OR high density lipoprotein OR Dyslipidemia OR Dyslipidaemia OR Hypercholesterolemia OR hypercholesterolaemia OR hypercholesterolimia OR Hypertriglyceridemia OR Hypertriglyceridemia OR Hypertriglyceridaemia OR Hyperlipidemia OR Hyperlipidemias OR Hyperlipidaemia OR LDL OR HDL OR Hypercholesterolemia OR Dyslipidemias OR blood lipid OR Cholesterol OR high cholesterol OR elevated cholesterol OR raised cholesterol OR low density lipoprotein OR high density lipoprotein OR Dyslipidemia OR Dyslipidaemia OR Hypercholesterolemia OR hypercholesterolaemia OR hypercholesterolimia OR Hypertriglyceridemia OR Hypertriglyceridemia OR Hypertriglyceridaemia OR Hyperlipidemia OR Hyperlipidemias OR Hyperlipidaemia OR LDL OR HDL

**#5** Comorbid OR co-morbid OR co-morbidity OR Multimorbidity OR multi-morbid OR multi morbidity OR Multimorbidity OR Comorbidity OR multi-disease OR Multidisease OR multi disease OR multiple condition OR multi-condition OR multi condition OR multiple illness OR multi-illness OR multi illness OR multiple syndrome OR multi-syndrome OR multi syndrome OR concurrent condition OR concurrent illness OR concurrent disease OR co-existing disease OR coexisting disease OR co-existing illness OR coexisting illness OR co-existing syndrome OR coexisting syndrome OR co-existing condition OR coexisting condition OR co-occurring disease OR co-occuring disease OR co-occuring disease OR co-occurring illness OR co occurring illness OR cooccurring illness OR co-occurring syndrome OR co occurring syndrome OR cooccurring syndrome OR co-occurring condition OR co occurring condition OR cooccurring condition

**#6** Wagner model OR Wagner* model* OR Wagner* chronic care model* OR Wagner chronic care model OR chronic care model* OR model OR collaborative care OR chronic care framework OR chronic disease care OR chronic illness care OR care model OR model care OR Wagner* model* OR Wagner* chronic care model* OR Wagner chronic care model OR theory OR concept OR framework OR model OR programme OR approach OR clinical pathway OR care pathway OR critical path OR vertical integration OR virtual integration OR physician system integration OR provider system integration OR functional integration OR horizontal integration OR clinical integration OR case management OR delivery of health care, integrated OR disease management OR patient care management OR patient-centred care OR accountable care organisations OR continuity of patient care OR case management OR comprehensive health care OR delivery of health care, integrated OR managed care programmes OR patient-centred care OR care delivery OR integrated care OR comprehensive care OR care coordination OR managed care OR accountable care OR accountable care organisations OR accountable care organisation OR collaborative care OR disease management OR case-management OR case management OR shared care OR accountable care OR patient-centred OR patient centred OR person-centred OR person centred OR multidisciplinary care OR interdisciplinary care OR inter-disciplinary care OR cross disciplinary care OR cross-disciplinary care OR multiple interventions OR care chain OR care chains OR care continuity OR care continuation OR care transition OR care transitions OR chain of care OR chains of care OR continuity of care OR cross sectoral care OR delivery of health care integrated OR integrated medicine OR integrated social network OR integrated social networks OR integration of care OR intersectoral care OR intrasectoral care OR linked care OR management model OR patient

care management OR seamless care OR service network OR service networks OR transition of care OR transitional care OR transmural care OR whole system thinking OR holistic care

**#7** Angola OR Benin OR Botswana OR Burkina Faso OR Upper Volta OR Burundi OR Cameroon OR Cameroons OR Cape Verde OR Central African Republic OR Chad OR Comoros OR Comoro Islands OR Comores OR Mayotte OR Congo OR Zaire OR Cote d'Ivoire OR Ivory Coast OR Democratic Republic of the Congo OR Djibouti OR French Somaliland OR Eritrea OR Ethiopia OR Gabon OR Gabonese Republic OR Gambia OR Ghana OR Gold Coast OR Guinea OR Kenya OR Lesotho OR Basutoland OR Liberia OR Madagascar OR Malagasy Republic OR Malawi OR Nyasaland OR Mali OR Mauritania OR Mauritius OR Mozambique OR Namibia OR Niger OR Nigeria OR Rwanda OR Sao Tome OR Seychelles OR Senegal OR Sierra Leone OR Somalia OR South Africa OR Sudan OR Eswatini OR Tanzania OR Togo OR Togolese Republic OR Uganda OR Zambia OR Zimbabwe OR Rhodesia OR Sub-Saharan Africa OR Africa South of the Sahara

**#8** trial* OR RCT

**#9** ((#1 OR #2 OR #3 OR #4 OR #5 ) AND #6 AND #7 AND #8) and Clinical Trial (Document Types) and 1999 or 2000 or 2001 or 2002 or 2003 or 2004 or 2005 or 2006 or 2007 or 2008 or 2009 or 2011 or 2010 or 2012 or 2013 or 2014 or 2015 or 2016 or 2017 or 2018 or 2019 or 2020 or 2021 or 2022 (Publication Years)

3. **SCOPUS**

( TITLE-ABS-KEY ( trial* OR rct ) ) AND ( ( ( TITLE-ABS-KEY ( "wagner model" OR "wagner* model*" OR "wagner* chronic care model*" OR "wagner chronic care model" OR "chronic care model*" OR "model " OR "collaborative care" OR "chronic care framework" OR "chronic disease care" OR "chronic illness care" OR care AND model OR "model care" OR "wagner* model*" OR "wagner* chronic care model*" OR "wagner chronic care model" OR "theory" OR "concept" OR "framework" OR "model" OR "programme" OR "approach" OR "clinical pathway" OR "care pathway" OR "critical path" OR "vertical integration" OR "virtual integration" OR "physician system integration" OR "provider system integration" OR "functional integration" OR "horizontal integration" OR "clinical integration" OR "case management" OR "delivery of health care, integrated" OR "disease management" OR "patient care management" OR "patient-centred care" ) ) OR ( TITLE-ABS-KEY ( "accountable care organisations" OR "continuity of patient care" OR "case management" OR "comprehensive health care" OR "delivery of health care, integrated" OR "managed care programmes" OR "patient-centred care" OR "care delivery" OR "integrated care" OR "comprehensive care" OR "care coordination" OR "managed care" OR "accountable care " OR "accountable care organisations" OR "accountable care organisation" OR "collaborative care" OR "disease management" OR "case-management" OR "case management" OR "shared care" OR "accountable care" OR "patient-centred" OR "patient centred" OR "person-centred" OR "person centred" OR "multidisciplinary care" OR "interdisciplinary care" OR "inter-disciplinary care" OR "cross disciplinary care" OR "cross-disciplinary care" OR "multiple interventions" OR "care chain" OR "care chains" OR "care continuity" OR "care continuation" OR "care transition" ) ) OR ( TITLE-ABS-KEY ( "care transitions" OR "chain of care" OR "chains of care" OR "continuity of care" OR "cross sectoral care" OR "delivery of health care integrated" OR "integrated medicine" OR "integrated social network" OR "integrated social networks" OR "integration of care" OR "intersectoral care" OR "intrasectoral care" OR "linked care" OR "management model" OR "patient care management" OR "seamless care" OR "service network" OR "service networks" OR "transition of care" OR "transitional care" OR "transmural care" OR "whole system thinking" OR "holistic care" ) ) ) AND ( ( TITLE-ABS-KEY ( ( "elevated blood pressure" OR "high blood pressure" OR "raised blood pressure" OR "diastolic blood pressure" OR "systolic blood pressure" OR "arterial blood pressure" OR "uncontrolled blood pressure" OR "uncontrolled hypertension" OR "elevated blood pressure" OR "high blood pressure" OR "raised blood pressure" OR "diastolic blood pressure" OR "systolic blood pressure" OR "arterial blood pressure" OR "uncontrolled blood pressure" OR "uncontrolled hypertension" ) ) ) OR ( TITLE-ABS-KEY ( ( "diabetes mellitus" OR "diabetes mellitus, type 2" OR "diabetes type 2" OR "t2dm" OR "diabetes type ii" OR "diabetes" OR "glucose intolerance" OR "insulin resistance" OR "hyperglycemia" OR "hyperglycaemia" OR "hypoglycaemia" OR "hypoglycaemia" OR "high blood sugar" OR "elevated blood sugar" OR "raised blood sugar" OR "high blood glucose" OR "elevated blood

glucose" OR "raised blood glucose" OR "diabetes mellitus" OR "diabetes mellitus, type 2" OR "diabetes type 2" OR "t2dm" OR "diabetes type ii" OR "diabetes" OR "glucose intolerance" OR "insulin resistance" OR "hyperglycemia" OR "hyperglycaemia" OR "hypoglycemia" OR "hypoglycaemia" OR "high blood sugar" OR "elevated blood sugar" OR "raised blood sugar" OR "high blood glucose" OR "elevated blood glucose" OR "raised blood glucose" ) ) ) OR ( TITLE-ABS-KEY ( ( "stroke" OR "transient ischemic attack" OR "ischemic attack" OR "angina" OR "angina pectoris" OR "heart attack" OR "ischemic heart diseases " OR "transient ischaemic attack" OR "coronary heart disease" OR "coronary disease" OR "heart failure" OR "peripheral vascular disease" OR "peripheral vascular diseases" OR "atrial fibrillation" OR "cardiovascular disease" OR "heart disease" OR "stroke" OR "transient ischemic attack" OR "ischemic attack" OR "angina" OR "angina pectoris" OR "heart attack" OR "ischemic heart diseases " OR "transient ischaemic attack" OR "coronary heart disease" OR "coronary disease" OR "heart failure" OR "peripheral vascular disease" OR "peripheral vascular diseases" OR "atrial fibrillation" OR "cardiovascular disease" OR "heart disease" ) ) ) OR ( TITLE-ABS-KEY ( ( "hypercholesterolemia" OR "dyslipidemias" OR "blood lipid" OR "cholesterol" OR "high cholesterol" OR "elevated cholesterol" OR "raised cholesterol" OR "low density lipoprotein " OR "high density lipoprotein" OR "dyslipidemia" OR "dyslipidaemia" OR "hypercholesterolemia" OR "hypercholesterolaemia " OR "hypercholesterolimia " OR "hypertriglyceridemia" OR "hypertriglyceridemia" OR "hypertriglyceridaemia" OR "hyperlipidemia" OR "hyperlipidemias" OR "hyperlipidaemia" OR "ldl" OR "hdl" OR "hypercholesterolemia" OR "dyslipidemias" OR "blood lipid" OR "cholesterol" OR "high cholesterol" OR "elevated cholesterol" OR "raised cholesterol" OR "low density lipoprotein " OR "high density lipoprotein" OR "dyslipidemia" OR "dyslipidaemia" OR "hypercholesterolemia" OR "hypercholesterolaemia " OR "hypercholesterolimia " OR "hypertriglyceridemia" OR "hypertriglyceridemia" OR "hypertriglyceridaemia" OR "hyperlipidemia" OR "hyperlipidemias" OR "hyperlipidaemia" OR "ldl" OR "hdl" ) ) ) OR ( TITLE-ABS-KEY ( ( "comorbid" OR "co-morbid" OR "co-morbidity" OR "multimorbidity" OR "multi-morbid" OR "multi morbidity" OR "multimorbidity" OR "comorbidity" OR "multi-disease" OR "multidisease" OR multi AND disease OR "multiple condition" OR "multi-condition" OR "multi condition" OR "multiple illness" OR "multi-illness" OR "multi illness" OR "multiple syndrome" OR "multi-syndrome" OR "multi syndrome" OR "concurrent condition" OR "concurrent illness" OR "concurrent disease" OR "co-existing disease" OR "coexisting disease" OR "co-existing illness" OR "coexisting illness" OR "co-existing syndrome" OR "coexisting syndrome" OR "co-existing condition" OR "coexisting condition" OR "co-occurring disease" OR "co-occuring disease" OR "co-occuring disease" OR "co-occurring illness" OR "co occurring illness" OR "cooccurring illness" OR "co-occurring syndrome" OR "co occurring syndrome" OR "cooccurring syndrome" OR "co-occurring condition" OR "co occurring condition" OR "cooccurring condition" ) ) ) ) AND ( TITLE-ABS-KEY ( ( "angola " OR "benin " OR "botswana " OR "burkina faso" OR "upper volta" OR "burundi " OR "cameroon " OR "cameroons " OR "cape verde" OR "central african republic" OR chad OR "comoros " OR "comoro islands" OR "comores" OR "mayotte " OR "congo " OR "zaire " OR "cote d'ivoire" OR "ivory coast" OR "democratic republic of the congo" OR "djibouti " OR "french somaliland" OR "eritrea " OR "ethiopia " OR "gabon " OR "gabonese republic" OR "gambia " OR "ghana " OR "gold coast" OR "guinea " OR "kenya " OR "lesotho" OR "basutoland" OR "liberia " OR "madagascar " OR "malagasy republic " OR "malawi " OR "nyasaland " OR "mali " OR "mauritania " OR "mauritius " OR "mozambique" OR "namibia" OR "niger" OR "nigeria" OR "rwanda" OR "sao tome" OR "seychelles" OR "senegal " OR "sierra leone" OR "somalia " OR "south africa" OR "sudan " OR "Eswatini" OR "tanzania" OR "togo " OR "Togolese republic" OR "uganda" OR "zambia" OR "zimbabwe " OR "rhodesia" OR "sub-saharan africa" OR "africa south of the sahara" ) ) ) ) ) AND ( LIMIT-TO ( PUBYEAR , 2022 ) OR LIMIT-TO ( PUBYEAR , 2021 ) OR LIMIT-TO ( PUBYEAR , 2020 ) OR LIMIT-TO ( PUBYEAR , 2019 ) OR LIMIT-TO ( PUBYEAR , 2018 ) OR LIMIT-TO ( PUBYEAR , 2017 ) OR LIMIT-TO ( PUBYEAR , 2016 ) OR LIMIT-TO ( PUBYEAR , 2015 ) OR LIMIT-TO ( PUBYEAR , 2014 ) OR LIMIT-TO ( PUBYEAR , 2013 ) OR LIMIT-TO ( PUBYEAR , 2012 ) OR LIMIT-TO ( PUBYEAR , 2011 ) OR LIMIT-TO ( PUBYEAR , 2010 ) OR LIMIT-TO ( PUBYEAR , 2009 ) OR LIMIT-TO ( PUBYEAR , 2008 ) OR LIMIT-TO ( PUBYEAR , 2007 ) OR LIMIT-TO ( PUBYEAR , 2006 ) OR LIMIT-TO ( PUBYEAR , 2005 ) OR LIMIT-

TO ( PUBYEAR , 2004 ) OR LIMIT-TO ( PUBYEAR , 2003 ) OR LIMIT-TO ( PUBYEAR , 2002 ) OR LIMIT-TO ( PUBYEAR , 2001 ) OR LIMIT-TO ( PUBYEAR , 2000 ) OR LIMIT-TO ( PUBYEAR , 1999 ) )

4. **Embase**

**#1** ("elevated blood pressure"/ OR "high blood pressure"/ OR "raised blood pressure"/ OR "diastolic blood pressure"/ OR "systolic blood pressure"/ OR "arterial blood pressure"/ OR "uncontrolled blood pressure"/ OR "uncontrolled hypertension" OR "elevated blood pressure" OR "high blood pressure" OR "raised blood pressure" OR "diastolic blood pressure" OR "systolic blood pressure" OR "arterial blood pressure" OR "uncontrolled blood pressure" OR "uncontrolled hypertension")

**#2** ("diabetes mellitus"/ OR "diabetes mellitus, type 2"/ OR "diabetes type 2"/ OR "T2DM"/ OR "diabetes type II"/ OR "diabetes"/ OR "glucose intolerance"/ OR "insulin resistance"/ OR "hyperglycemia"/ OR "hyperglycaemia"/ OR "hypoglycaemia"/ OR "hypoglycaemia"/ OR "high blood sugar"/ OR "elevated blood sugar"/ OR "raised blood sugar"/ OR "high blood glucose"/ OR "elevated blood glucose"/ OR "raised blood glucose"/ OR "diabetes mellitus" OR "diabetes mellitus, type 2" OR "diabetes type 2" OR "T2DM" OR "diabetes type II" OR "diabetes" OR "glucose intolerance" OR "insulin resistance" OR "hyperglycemia" OR "hyperglycaemia" OR "hypoglycemia" OR "hypoglycaemia" OR "high blood sugar" OR "elevated blood sugar" OR "raised blood sugar" OR "high blood glucose" OR "elevated blood glucose" OR "raised blood glucose")

**#3** ("stroke"/ OR "transient ischemic attack"/ OR "ischemic attack"/ OR "angina"/ OR "angina pectoris"/ OR "heart attack"/ OR "ischemic heart diseases "/ OR "transient ischaemic attack"/ OR "coronary heart disease"/ OR "coronary disease"/ OR "heart failure"/ OR "peripheral vascular disease"/ OR "Peripheral Vascular diseases"/ OR "atrial fibrillation"/ OR "cardiovascular disease"/ OR "heart disease"/ OR "stroke" OR "transient ischemic attack" OR "ischemic attack" OR "angina" OR "angina pectoris" OR "heart attack" OR "ischemic heart diseases " OR "transient ischaemic attack" OR "coronary heart disease" OR "coronary disease" OR "heart failure" OR "peripheral vascular disease" OR "Peripheral Vascular diseases" OR "atrial fibrillation" OR "cardiovascular disease" OR "heart disease")

**#4** ("Hypercholesterolemia"/ OR "Dyslipidemias"/ OR "blood lipid"/ OR "Cholesterol"/ OR "high cholesterol"/ OR "elevated cholesterol"/ OR "raised cholesterol"/ OR "low density lipoprotein "/ OR "high density lipoprotein"/ OR "Dyslipidemia"/ OR "Dyslipidaemia"/ OR "Hypercholesterolemia"/ OR "hypercholesterolaemia "/ OR "hypercholesterolimia "/ OR "Hypertriglyceridemia"/ OR "Hypertriglyceridemia"/ OR "Hypertriglyceridaemia"/ OR "Hyperlipidemia"/ OR "Hyperlipidemias"/ OR "Hyperlipidaemia"/ OR "LDL"/ OR "HDL"/ OR "Hypercholesterolemia" OR "Dyslipidemias" OR "blood lipid" OR "Cholesterol" OR "high cholesterol" OR "elevated cholesterol" OR "raised cholesterol" OR "low density lipoprotein " OR "high density lipoprotein" OR "Dyslipidemia" OR "Dyslipidaemia" OR "Hypercholesterolemia" OR "hypercholesterolaemia " OR "hypercholesterolimia " OR "Hypertriglyceridemia" OR "Hypertriglyceridemia" OR "Hypertriglyceridaemia" OR "Hyperlipidemia" OR "Hyperlipidemias" OR "Hyperlipidaemia" OR "LDL" OR "HDL")

**#5** ("Comorbid" OR "co-morbid" OR "co-morbidity" OR "Multimorbidity" OR "multi-morbid" OR "multi morbidity" OR "Multimorbidity" OR "Comorbidity" OR "multi-disease" OR "Multidisease" OR multi disease OR "multiple condition" OR "multi-condition" OR "multi condition" OR "multiple illness" OR "multi-illness" OR "multi illness" OR "multiple syndrome" OR "multi-syndrome" OR "multi syndrome" OR "concurrent condition" OR "concurrent illness" OR "concurrent disease" OR "co-existing disease" OR "coexisting disease" OR "co-existing illness" OR "coexisting illness" OR "co-existing syndrome" OR "coexisting syndrome" OR "co-existing condition" OR "coexisting condition" OR "co-occurring disease" OR "co-occuring disease" OR "co-occuring disease" OR "co-occurring illness" OR "co occurring illness" OR "cooccurring illness" OR "co-occurring syndrome" OR "co occurring syndrome" OR "cooccurring syndrome" OR "co-occurring condition" OR "co occurring condition" OR "cooccurring condition")

**#6** ("Wagner model" OR "Wagner* model*" OR "Wagner* chronic care model*" OR "Wagner chronic care model" OR "chronic care model*" OR "model " OR "collaborative care" OR "chronic care framework" OR "chronic disease care" OR "chronic illness care" OR care model OR "model care" OR "Wagner* model*" OR "Wagner* chronic care model*" OR "Wagner chronic care model" OR "theory" OR "concept" OR "framework" OR "model"



Otieno P, *et al. BMJ Open* 2023; 13:e073652. doi: 10.1136/bmjopen-2023-073652

OR "programme" OR "approach" OR "clinical pathway" OR "care pathway" OR "critical path" OR "vertical integration" OR "virtual integration" OR "physician system integration" OR "provider system integration" OR "functional integration" OR "horizontal integration" OR "clinical integration" OR "case management" OR "delivery of health care, integrated" OR "disease management" OR "patient care management" OR "patient-centred care" OR "accountable care organisations" OR "continuity of patient care" OR "case management" OR "comprehensive health care" OR "delivery of health care, integrated" OR "managed care programmes" OR "patient-centred care" OR "care delivery" OR "integrated care" OR "comprehensive care" OR "care coordination" OR "managed care" OR "accountable care " OR "accountable care organisations" OR "accountable care organisation" OR "collaborative care" OR "disease management" OR "case-management" OR "case management" OR "shared care" OR "accountable care" OR "patient-centred" OR "patient centred" OR "person-centred" OR "person centred" OR "multidisciplinary care" OR "interdisciplinary care" OR "inter-disciplinary care" OR "cross disciplinary care" OR "cross-disciplinary care" OR "multiple interventions" OR "care chain" OR "care chains" OR "care continuity" OR "care continuation" OR "care transition" OR "care transitions" OR "chain of care" OR "chains of care" OR "continuity of care" OR "cross sectoral care" OR "delivery of health care integrated" OR "integrated medicine" OR "integrated social network" OR "integrated social networks" OR "integration of care" OR "intersectoral care" OR "intrasectoral care" OR "linked care" OR "management model" OR "patient care management" OR "seamless care" OR "service network" OR "service networks" OR "transition of care" OR "transitional care" OR "transmural care" OR "whole system thinking" OR "holistic care")

**#7** ("Angola " OR "Benin " OR "Botswana " OR "Burkina Faso" OR "Upper Volta" OR "Burundi " OR "Cameroon " OR "Cameroons " OR "Cape Verde" OR "Central African Republic" OR Chad OR "Comoros " OR "Comoro Islands" OR "Comores" OR "Mayotte " OR "Congo " OR "Zaire " OR "Cote d'Ivoire" OR "Ivory Coast" OR "Democratic Republic of the Congo" OR "Djibouti " OR "French Somaliland" OR "Eritrea " OR "Ethiopia " OR "Gabon " OR "Gabonese Republic" OR "Gambia " OR "Ghana " OR "Gold Coast" OR "Guinea " OR "Kenya " OR "Lesotho" OR "Basutoland" OR "Liberia " OR "Madagascar " OR "Malagasy Republic " OR "Malawi " OR "Nyasaland " OR "Mali " OR "Mauritania " OR "Mauritius " OR "Mozambique" OR "Namibia" OR "Niger" OR "Nigeria" OR "Rwanda" OR "Sao Tome" OR "Seychelles" OR "Senegal " OR "Sierra Leone" OR "Somalia " OR "South Africa" OR "Sudan " OR "Eswatini" OR "Tanzania" OR "Togo " OR "Togolese Republic" OR "Uganda" OR "Zambia" OR "Zimbabwe " OR "Rhodesia" OR "Sub-Saharan Africa" OR "Africa South of the Sahara")

**#8** ((#1 OR #2 OR #3 OR #4 OR #5 ) AND #6 AND #7) & 1999 - 2022 & RCTs

5. **Cochrane Library**

**#1** (("elevated blood pressure"(MeSH Terms) OR "high blood pressure"(MeSH Terms) OR "raised blood pressure"(MeSH Terms) OR "diastolic blood pressure"(MeSH Terms) OR "systolic blood pressure"(MeSH Terms) OR "arterial blood pressure"(MeSH Terms) OR "uncontrolled blood pressure"(MeSH Terms) OR "uncontrolled hypertension" OR "elevated blood pressure" OR "high blood pressure" OR "raised blood pressure" OR "diastolic blood pressure" OR "systolic blood pressure" OR "arterial blood pressure" OR "uncontrolled blood pressure" OR "uncontrolled hypertension")):ti,ab,kw (Word variations have been searched)

**#2** (("diabetes mellitus"(MeSH Terms) OR "diabetes mellitus, type 2"(MeSH Terms) OR "diabetes type 2"(MeSH Terms) OR "T2DM"(MeSH Terms) OR "diabetes type II"(MeSH Terms) OR "diabetes"(MeSH Terms) OR "glucose intolerance"(MeSH Terms) OR "insulin resistance"(MeSH Terms) OR "hyperglycemia"(MeSH Terms) OR "hyperglycaemia"(MeSH Terms) OR "hypoglycaemia"(MeSH Terms) OR "hypoglycaemia"(MeSH Terms) OR "high blood sugar"(MeSH Terms) OR "elevated blood sugar"(MeSH Terms) OR "raised blood sugar"(MeSH Terms) OR "high blood glucose"(MeSH Terms) OR "elevated blood glucose"(MeSH Terms) OR "raised blood glucose"(MeSH Terms) OR "diabetes mellitus" OR "diabetes mellitus, type 2" OR "diabetes type 2" OR "T2DM" OR "diabetes type II" OR "diabetes" OR "glucose intolerance" OR "insulin resistance" OR "hyperglycemia" OR "hyperglycaemia" OR "hypoglycemia" OR "hypoglycaemia" OR "high blood sugar" OR "elevated blood sugar" OR "raised blood sugar" OR "high blood glucose" OR "elevated blood glucose" OR "raised blood glucose")):ti,ab,kw (Word variations have been searched)



Otieno P, *et al. BMJ Open* 2023; 13:e073652. doi: 10.1136/bmjopen-2023-073652

**#3** (("stroke"(MeSH Terms) OR "transient ischemic attack"(MeSH Terms) OR "ischemic attack"(MeSH Terms) OR "angina"(MeSH Terms) OR "angina pectoris"(MeSH Terms) OR "heart attack"(MeSH Terms) OR "ischemic heart diseases "(MeSH Terms) OR "transient ischaemic attack"(MeSH Terms) OR "coronary heart disease"(MeSH Terms) OR "coronary disease"(MeSH Terms) OR "heart failure"(MeSH Terms) OR "peripheral vascular disease"(MeSH Terms) OR "Peripheral Vascular diseases"(MeSH Terms) OR "atrial fibrillation"(MeSH Terms) OR "cardiovascular disease"(MeSH Terms) OR "heart disease"(MeSH Terms) OR "stroke" OR "transient ischemic attack" OR "ischemic attack" OR "angina" OR "angina pectoris" OR "heart attack" OR "ischemic heart diseases " OR "transient ischaemic attack" OR "coronary heart disease" OR "coronary disease" OR "heart failure" OR "peripheral vascular disease" OR "Peripheral Vascular diseases" OR "atrial fibrillation" OR "cardiovascular disease" OR "heart disease")):ti,ab,kw (Word variations have been searched)

**#4** (("Hypercholesterolemia"(MeSH Terms) OR "Dyslipidemias"(MeSH Terms) OR "blood lipid"(MeSH Terms) OR "Cholesterol"(MeSH Terms) OR "high cholesterol"(MeSH Terms) OR "elevated cholesterol"(MeSH Terms) OR "raised cholesterol"(MeSH Terms) OR "low density lipoprotein "(MeSH Terms) OR "high density lipoprotein"(MeSH Terms) OR "Dyslipidemia"(MeSH Terms) OR "Dyslipidaemia"(MeSH Terms) OR "Hypercholesterolemia"(MeSH Terms) OR "hypercholesterolaemia "(MeSH Terms) OR "hypercholesterolimia "(MeSH Terms) OR "Hypertriglyceridemia"(MeSH Terms) OR "Hypertriglyceridemia"(MeSH Terms) OR "Hypertriglyceridaemia"(MeSH Terms) OR "Hyperlipidemia"(MeSH Terms) OR "Hyperlipidemias"(MeSH Terms) OR "Hyperlipidaemia"(MeSH Terms) OR "LDL"(MeSH Terms) OR "HDL"(MeSH Terms) OR "Hypercholesterolemia" OR "Dyslipidemias" OR "blood lipid" OR "Cholesterol" OR "high cholesterol" OR "elevated cholesterol" OR "raised cholesterol" OR "low density lipoprotein " OR "high density lipoprotein" OR "Dyslipidemia" OR "Dyslipidaemia" OR "Hypercholesterolemia" OR "hypercholesterolaemia " OR "hypercholesterolimia " OR "Hypertriglyceridemia" OR "Hypertriglyceridemia" OR "Hypertriglyceridaemia" OR "Hyperlipidemia" OR "Hyperlipidemias" OR "Hyperlipidaemia" OR "LDL" OR "HDL")):ti,ab,kw (Word variations have been searched)

**#5** (("Comorbid" OR "co-morbid" OR "co-morbidity" OR "Multimorbidity" OR "multi-morbid" OR "multi morbidity" OR "Multimorbidity" OR "Comorbidity" OR "multi-disease" OR "Multidisease" OR multi disease OR "multiple condition" OR "multi-condition" OR "multi condition" OR "multiple illness" OR "multi-illness" OR "multi illness" OR "multiple syndrome" OR "multi-syndrome" OR "multi syndrome" OR "concurrent condition" OR "concurrent illness" OR "concurrent disease" OR "co-existing disease" OR "coexisting disease" OR "co-existing illness" OR "coexisting illness" OR "co-existing syndrome" OR "coexisting syndrome" OR "co-existing condition" OR "coexisting condition" OR "co-occurring disease" OR "co-occuring disease" OR "co-occuring disease" OR "co-occurring illness" OR "co occurring illness" OR "cooccurring illness" OR "co-occurring syndrome" OR "co occurring syndrome" OR "cooccurring syndrome" OR "co-occurring condition" OR "co occurring condition" OR "cooccurring condition")):ti,ab,kw (Word variations have been searched)

**#6** (("Wagner model" OR "Wagner* model*" OR "Wagner* chronic care model*" OR "Wagner chronic care model" OR "chronic care model*" OR "model " OR "collaborative care" OR "chronic care framework" OR "chronic disease care" OR "chronic illness care" OR care model OR "model care" OR "Wagner* model*" OR "Wagner* chronic care model*" OR "Wagner chronic care model" OR "theory" OR "concept" OR "framework" OR "model" OR "programme" OR "approach" OR "clinical pathway" OR "care pathway" OR "critical path" OR "vertical integration" OR "virtual integration" OR "physician system integration" OR "provider system integration" OR "functional integration" OR "horizontal integration" OR "clinical integration" OR "case management" OR "delivery of health care, integrated" OR "disease management" OR "patient care management" OR "patient-centred care" OR "accountable care organisations" OR "continuity of patient care" OR "case management" OR "comprehensive health care" OR "delivery of health care, integrated" OR "managed care programmes" OR "patient-centred care" OR "care delivery" OR "integrated care" OR "comprehensive care" OR "care coordination" OR "managed care" OR "accountable care " OR "accountable care organisations" OR "accountable care organisation" OR "collaborative care" OR "disease management" OR "case-management" OR "case management" OR "shared care" OR "accountable care" OR "patient-centred" OR "patient centred" OR "person-centred" OR "person centred" OR "multidisciplinary care" OR "interdisciplinary care" OR "inter-disciplinary care" OR "cross disciplinary care" OR "cross-disciplinary care" OR "multiple interventions" OR "care chain" OR "care chains" OR "care continuity" OR

"care continuation" OR "care transition" OR "care transitions" OR "chain of care" OR "chains of care" OR "continuity of care" OR "cross sectoral care" OR "delivery of health care integrated" OR "integrated medicine" OR "integrated social network" OR "integrated social networks" OR "integration of care" OR "intersectoral care" OR "intrasectoral care" OR "linked care" OR "management model" OR "patient care management" OR "seamless care" OR "service network" OR "service networks" OR "transition of care" OR "transitional care" OR "transmural care" OR "whole system thinking" OR "holistic care")):ti,ab,kw (Word variations have been searched)

**#7** (("Angola " OR "Benin " OR "Botswana " OR "Burkina Faso" OR "Upper Volta" OR "Burundi " OR "Cameroon " OR "Cameroons " OR "Cape Verde" OR "Central African Republic" OR Chad OR "Comoros " OR "Comoro Islands" OR "Comores" OR "Mayotte " OR "Congo " OR "Zaire " OR "Cote d'Ivoire" OR "Ivory Coast" OR "Democratic Republic of the Congo" OR "Djibouti " OR "French Somaliland" OR "Eritrea " OR "Ethiopia " OR "Gabon " OR "Gabonese Republic" OR "Gambia " OR "Ghana " OR "Gold Coast" OR "Guinea " OR "Kenya " OR "Lesotho" OR "Basutoland" OR "Liberia " OR "Madagascar " OR "Malagasy Republic " OR "Malawi " OR "Nyasaland " OR "Mali " OR "Mauritania " OR "Mauritius " OR "Mozambique" OR "Namibia" OR "Niger" OR "Nigeria" OR "Rwanda" OR "Sao Tome" OR "Seychelles" OR "Senegal " OR "Sierra Leone" OR "Somalia " OR "South Africa" OR "Sudan " OR "Eswatini" OR "Tanzania" OR "Togo " OR "Togolese Republic" OR "Uganda" OR "Zambia" OR "Zimbabwe " OR "Rhodesia" OR "Sub-Saharan Africa" OR "Africa South of the Sahara")):ti,ab,kw (Word variations have been searched)

**#8** ((#1 OR #2 OR #3 OR #4 OR #5 ) AND #6 AND #7) AND Restricted (1999-2022, RCTs)

6. **Psycinfo via Ovid**

**#1** ("elevated blood pressure"/ OR "high blood pressure"/ OR "raised blood pressure"/ OR "diastolic blood pressure"/ OR "systolic blood pressure"/ OR "arterial blood pressure"/ OR "uncontrolled blood pressure"/ OR "uncontrolled hypertension" OR "elevated blood pressure" OR "high blood pressure" OR "raised blood pressure" OR "diastolic blood pressure" OR "systolic blood pressure" OR "arterial blood pressure" OR "uncontrolled blood pressure" OR "uncontrolled hypertension")

**#2** ("diabetes mellitus"/ OR "diabetes mellitus, type 2"/ OR "diabetes type 2"/ OR "T2DM"/ OR "diabetes type II"/ OR "diabetes"/ OR "glucose intolerance"/ OR "insulin resistance"/ OR "hyperglycemia"/ OR "hyperglycaemia"/ OR "hypoglycaemia"/ OR "hypoglycaemia"/ OR "high blood sugar"/ OR "elevated blood sugar"/ OR "raised blood sugar"/ OR "high blood glucose"/ OR "elevated blood glucose"/ OR "raised blood glucose"/ OR "diabetes mellitus" OR "diabetes mellitus, type 2" OR "diabetes type 2" OR "T2DM" OR "diabetes type II" OR "diabetes" OR "glucose intolerance" OR "insulin resistance" OR "hyperglycemia" OR "hyperglycaemia" OR "hypoglycemia" OR "hypoglycaemia" OR "high blood sugar" OR "elevated blood sugar" OR "raised blood sugar" OR "high blood glucose" OR "elevated blood glucose" OR "raised blood glucose")

**#3** ("stroke"/ OR "transient ischemic attack"/ OR "ischemic attack"/ OR "angina"/ OR "angina pectoris"/ OR "heart attack"/ OR "ischemic heart diseases "/ OR "transient ischaemic attack"/ OR "coronary heart disease"/ OR "coronary disease"/ OR "heart failure"/ OR "peripheral vascular disease"/ OR "Peripheral Vascular diseases"/ OR "atrial fibrillation"/ OR "cardiovascular disease"/ OR "heart disease"/ OR "stroke" OR "transient ischemic attack" OR "ischemic attack" OR "angina" OR "angina pectoris" OR "heart attack" OR "ischemic heart diseases " OR "transient ischaemic attack" OR "coronary heart disease" OR "coronary disease" OR "heart failure" OR "peripheral vascular disease" OR "Peripheral Vascular diseases" OR "atrial fibrillation" OR "cardiovascular disease" OR "heart disease")

**#4** ("Hypercholesterolemia"/ OR "Dyslipidemias"/ OR "blood lipid"/ OR "Cholesterol"/ OR "high cholesterol"/ OR "elevated cholesterol"/ OR "raised cholesterol"/ OR "low density lipoprotein "/ OR "high density lipoprotein"/ OR "Dyslipidemia"/ OR "Dyslipidaemia"/ OR "Hypercholesterolemia"/ OR "hypercholesterolaemia "/ OR "hypercholesterolimia "/ OR "Hypertriglyceridemia"/ OR "Hypertriglyceridemia"/ OR "Hypertriglyceridaemia"/ OR "Hyperlipidemia"/ OR "Hyperlipidemias"/ OR "Hyperlipidaemia"/ OR "LDL"/ OR "HDL"/ OR "Hypercholesterolemia" OR "Dyslipidemias" OR "blood lipid" OR "Cholesterol" OR "high cholesterol" OR "elevated cholesterol" OR "raised cholesterol" OR "low density lipoprotein " OR "high density lipoprotein" OR

"Dyslipidemia" OR "Dyslipidaemia" OR "Hypercholesterolemia" OR "hypercholesterolaemia " OR "hypercholesterolimia " OR "Hypertriglyceridemia" OR "Hypertriglyceridemia" OR "Hypertriglyceridaemia" OR "Hyperlipidemia" OR "Hyperlipidemias" OR "Hyperlipidaemia" OR "LDL" OR "HDL")

**#5** ("Comorbid" OR "co-morbid" OR "co-morbidity" OR "Multimorbidity" OR "multi-morbid" OR "multi morbidity" OR "Multimorbidity" OR "Comorbidity" OR "multi-disease" OR "Multidisease" OR multi disease OR "multiple condition" OR "multi-condition" OR "multi condition" OR "multiple illness" OR "multi-illness" OR "multi illness" OR "multiple syndrome" OR "multi-syndrome" OR "multi syndrome" OR "concurrent condition" OR "concurrent illness" OR "concurrent disease" OR "co-existing disease" OR "coexisting disease" OR "co-existing illness" OR "coexisting illness" OR "co-existing syndrome" OR "coexisting syndrome" OR "co-existing condition" OR "coexisting condition" OR "co-occurring disease" OR "co-occuring disease" OR "co-occuring disease" OR "co-occurring illness" OR "co occurring illness" OR "cooccurring illness" OR "co-occurring syndrome" OR "co occurring syndrome" OR "cooccurring syndrome" OR "co-occurring condition" OR "co occurring condition" OR "cooccurring condition")

**#6** ("Wagner model" OR "Wagner* model*" OR "Wagner* chronic care model*" OR "Wagner chronic care model" OR "chronic care model*" OR "model " OR "collaborative care" OR "chronic care framework" OR "chronic disease care" OR "chronic illness care" OR care model OR "model care" OR "Wagner* model*" OR "Wagner* chronic care model*" OR "Wagner chronic care model" OR "theory" OR "concept" OR "framework" OR "model" OR "programme" OR "approach" OR "clinical pathway" OR "care pathway" OR "critical path" OR "vertical integration" OR "virtual integration" OR "physician system integration" OR "provider system integration" OR "functional integration" OR "horizontal integration" OR "clinical integration" OR "case management" OR "delivery of health care, integrated" OR "disease management" OR "patient care management" OR "patient-centred care" OR "accountable care organisations" OR "continuity of patient care" OR "case management" OR "comprehensive health care" OR "delivery of health care, integrated" OR "managed care programmes" OR "patient-centred care" OR "care delivery" OR "integrated care" OR "comprehensive care" OR "care coordination" OR "managed care" OR "accountable care " OR "accountable care organisations" OR "accountable care organisation" OR "collaborative care" OR "disease management" OR "case-management" OR "case management" OR "shared care" OR "accountable care" OR "patient-centred" OR "patient centred" OR "person-centred" OR "person centred" OR "multidisciplinary care" OR "interdisciplinary care" OR "inter-disciplinary care" OR "cross disciplinary care" OR "cross-disciplinary care" OR "multiple interventions" OR "care chain" OR "care chains" OR "care continuity" OR "care continuation" OR "care transition" OR "care transitions" OR "chain of care" OR "chains of care" OR "continuity of care" OR "cross sectoral care" OR "delivery of health care integrated" OR "integrated medicine" OR "integrated social network" OR "integrated social networks" OR "integration of care" OR "intersectoral care" OR "intrasectoral care" OR "linked care" OR "management model" OR "patient care management" OR "seamless care" OR "service network" OR "service networks" OR "transition of care" OR "transitional care" OR "transmural care" OR "whole system thinking" OR "holistic care")

**#7** ("Angola " OR "Benin " OR "Botswana " OR "Burkina Faso" OR "Upper Volta" OR "Burundi " OR "Cameroon " OR "Cameroons " OR "Cape Verde" OR "Central African Republic" OR Chad OR "Comoros " OR "Comoro Islands" OR "Comores" OR "Mayotte " OR "Congo " OR "Zaire " OR "Cote d'Ivoire" OR "Ivory Coast" OR "Democratic Republic of the Congo" OR "Djibouti " OR "French Somaliland" OR "Eritrea " OR "Ethiopia " OR "Gabon " OR "Gabonese Republic" OR "Gambia " OR "Ghana " OR "Gold Coast" OR "Guinea " OR "Kenya " OR "Lesotho" OR "Basutoland" OR "Liberia " OR "Madagascar " OR "Malagasy Republic " OR "Malawi " OR "Nyasaland " OR "Mali " OR "Mauritania " OR "Mauritius " OR "Mozambique" OR "Namibia" OR "Niger" OR "Nigeria" OR "Rwanda" OR "Sao Tome" OR "Seychelles" OR "Senegal " OR "Sierra Leone" OR "Somalia " OR "South Africa" OR "Sudan " OR "Eswatini" OR "Tanzania" OR "Togo " OR "Togolese Republic" OR "Uganda" OR "Zambia" OR "Zimbabwe " OR "Rhodesia" OR "Sub-Saharan Africa" OR "Africa South of the Sahara")

8 ((#1 OR #2 OR #3 OR #4 OR #5 ) AND #6 AND #7)

9 limit 8 to ("0300 clinical trial" and yr="1999 - 2022")

7. **CINAHL**

**#1** ("elevated blood pressure" OR "high blood pressure" OR "raised blood pressure" OR "diastolic blood pressure" OR "systolic blood pressure" OR "arterial blood pressure" OR "uncontrolled blood pressure" OR "uncontrolled hypertension" OR "elevated blood pressure" OR "high blood pressure" OR "raised blood pressure" OR "diastolic blood pressure" OR "systolic blood pressure" OR "arterial blood pressure" OR "uncontrolled blood pressure" OR "uncontrolled hypertension")

**#2** ("diabetes mellitus" OR "diabetes mellitus, type 2" OR "diabetes type 2" OR "T2DM" OR "diabetes type II" OR "diabetes" OR "glucose intolerance" OR "insulin resistance" OR "hyperglycemia" OR "hyperglycaemia" OR "hypoglycaemia" OR "hypoglycaemia" OR "high blood sugar" OR "elevated blood sugar" OR "raised blood sugar" OR "high blood glucose" OR "elevated blood glucose" OR "raised blood glucose" OR "diabetes mellitus" OR "diabetes mellitus, type 2" OR "diabetes type 2" OR "T2DM" OR "diabetes type II" OR "diabetes" OR "glucose intolerance" OR "insulin resistance" OR "hyperglycemia" OR "hyperglycaemia" OR "hypoglycemia" OR "hypoglycaemia" OR "high blood sugar" OR "elevated blood sugar" OR "raised blood sugar" OR "high blood glucose" OR "elevated blood glucose" OR "raised blood glucose")

**#3** ("stroke" OR "transient ischemic attack" OR "ischemic attack" OR "angina" OR "angina pectoris" OR "heart attack" OR "ischemic heart diseases " OR "transient ischaemic attack" OR "coronary heart disease" OR "coronary disease" OR "heart failure" OR "peripheral vascular disease" OR "Peripheral Vascular diseases" OR "atrial fibrillation" OR "cardiovascular disease" OR "heart disease" OR "stroke" OR "transient ischemic attack" OR "ischemic attack" OR "angina" OR "angina pectoris" OR "heart attack" OR "ischemic heart diseases " OR "transient ischaemic attack" OR "coronary heart disease" OR "coronary disease" OR "heart failure" OR "peripheral vascular disease" OR "Peripheral Vascular diseases" OR "atrial fibrillation" OR "cardiovascular disease" OR "heart disease")

**#4** ("Hypercholesterolemia" OR "Dyslipidemias" OR "blood lipid" OR "Cholesterol" OR "high cholesterol" OR "elevated cholesterol" OR "raised cholesterol" OR "low density lipoprotein " OR "high density lipoprotein" OR "Dyslipidemia" OR "Dyslipidaemia" OR "Hypercholesterolemia" OR "hypercholesterolaemia " OR "hypercholesterolimia " OR "Hypertriglyceridemia" OR "Hypertriglyceridemia" OR "Hypertriglyceridaemia" OR "Hyperlipidemia" OR "Hyperlipidemias" OR "Hyperlipidaemia" OR "LDL" OR "HDL" OR "Hypercholesterolemia" OR "Dyslipidemias" OR "blood lipid" OR "Cholesterol" OR "high cholesterol" OR "elevated cholesterol" OR "raised cholesterol" OR "low density lipoprotein " OR "high density lipoprotein" OR "Dyslipidemia" OR "Dyslipidaemia" OR "Hypercholesterolemia" OR "hypercholesterolaemia " OR "hypercholesterolimia " OR "Hypertriglyceridemia" OR "Hypertriglyceridemia" OR "Hypertriglyceridaemia" OR "Hyperlipidemia" OR "Hyperlipidemias" OR "Hyperlipidaemia" OR "LDL" OR "HDL")

**#5** ("Comorbid" OR "co-morbid" OR "co-morbidity" OR "Multimorbidity" OR "multi-morbid" OR "multi morbidity" OR "Multimorbidity" OR "Comorbidity" OR "multi-disease" OR "Multidisease" OR multi disease OR "multiple condition" OR "multi-condition" OR "multi condition" OR "multiple illness" OR "multi-illness" OR "multi illness" OR "multiple syndrome" OR "multi-syndrome" OR "multi syndrome" OR "concurrent condition" OR "concurrent illness" OR "concurrent disease" OR "co-existing disease" OR "coexisting disease" OR "co-existing illness" OR "coexisting illness" OR "co-existing syndrome" OR "coexisting syndrome" OR "co-existing condition" OR "coexisting condition" OR "co-occurring disease" OR "co-occuring disease" OR "co-occuring disease" OR "co-occurring illness" OR "co occurring illness" OR "cooccurring illness" OR "co-occurring syndrome" OR "co occurring syndrome" OR "cooccurring syndrome" OR "co-occurring condition" OR "co occurring condition" OR "cooccurring condition")

**#6** ("Wagner model" OR "Wagner* model*" OR "Wagner* chronic care model*" OR "Wagner chronic care model" OR "chronic care model*" OR "model " OR "collaborative care" OR "chronic care framework" OR "chronic disease care" OR "chronic illness care" OR care model OR "model care" OR "Wagner* model*" OR "Wagner* chronic care model*" OR "Wagner chronic care model" OR "theory" OR "concept" OR "framework" OR "model" OR "programme" OR "approach" OR "clinical pathway" OR "care pathway" OR "critical path" OR "vertical integration" OR "virtual integration" OR "physician system integration" OR "provider system integration" OR "functional integration" OR "horizontal integration" OR "clinical integration" OR "case management" OR "delivery of health care, integrated" OR "disease management" OR "patient care management" OR "patient-centred care" OR "accountable care organisations" OR "continuity of patient care" OR "case management" OR "comprehensive health

care" OR "delivery of health care, integrated" OR "managed care programmes" OR "patient-centred care" OR "care delivery" OR "integrated care" OR "comprehensive care" OR "care coordination" OR "managed care" OR "accountable care " OR "accountable care organisations" OR "accountable care organisation" OR "collaborative care" OR "disease management" OR "case-management" OR "case management" OR "shared care" OR "accountable care" OR "patient-centred" OR "patient centred" OR "person-centred" OR "person centred" OR "multidisciplinary care" OR "interdisciplinary care" OR "inter-disciplinary care" OR "cross disciplinary care" OR "cross-disciplinary care" OR "multiple interventions" OR "care chain" OR "care chains" OR "care continuity" OR "care continuation" OR "care transition" OR "care transitions" OR "chain of care" OR "chains of care" OR "continuity of care" OR "cross sectoral care" OR "delivery of health care integrated" OR "integrated medicine" OR "integrated social network" OR "integrated social networks" OR "integration of care" OR "intersectoral care" OR "intrasectoral care" OR "linked care" OR "management model" OR "patient care management" OR "seamless care" OR "service network" OR "service networks" OR "transition of care" OR "transitional care" OR "transmural care" OR "whole system thinking" OR "holistic care")

**#7** ("Angola " OR "Benin " OR "Botswana " OR "Burkina Faso" OR "Upper Volta" OR "Burundi " OR "Cameroon " OR "Cameroons " OR "Cape Verde" OR "Central African Republic" OR Chad OR "Comoros " OR "Comoro Islands" OR "Comores" OR "Mayotte " OR "Congo " OR "Zaire " OR "Cote d'Ivoire" OR "Ivory Coast" OR "Democratic Republic of the Congo" OR "Djibouti " OR "French Somaliland" OR "Eritrea " OR "Ethiopia " OR "Gabon " OR "Gabonese Republic" OR "Gambia " OR "Ghana " OR "Gold Coast" OR "Guinea " OR "Kenya " OR "Lesotho" OR "Basutoland" OR "Liberia " OR "Madagascar " OR "Malagasy Republic " OR "Malawi " OR "Nyasaland " OR "Mali " OR "Mauritania " OR "Mauritius " OR "Mozambique" OR "Namibia" OR "Niger" OR "Nigeria" OR "Rwanda" OR "Sao Tome" OR "Seychelles" OR "Senegal " OR "Sierra Leone" OR "Somalia " OR "South Africa" OR "Sudan " OR "Eswatini" OR "Tanzania" OR "Togo " OR "Togolese Republic" OR "Uganda" OR "Zambia" OR "Zimbabwe " OR "Rhodesia" OR "Sub-Saharan Africa" OR "Africa South of the Sahara")

**#8** ((S1 OR S2 OR S3 OR S4 OR S5 ) AND S6 AND S7) & 1999-2022 & research article& peer-reviewed



Otieno P, *et al. BMJ Open* 2023; 13:e073652. doi: 10.1136/bmjopen-2023-073652

**Online supplementary file 3: Sensitivity analysis of RCTs and cluster RCTs**

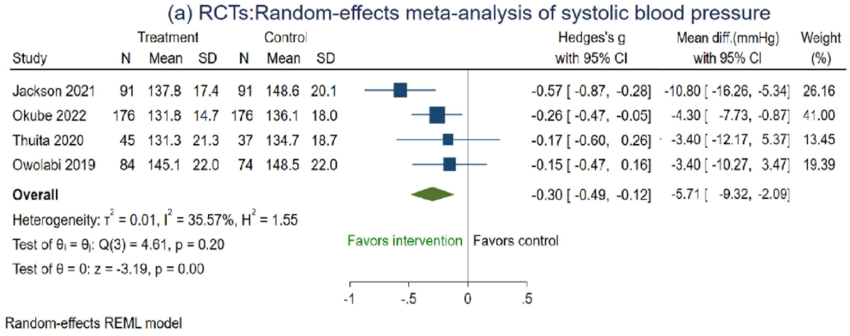


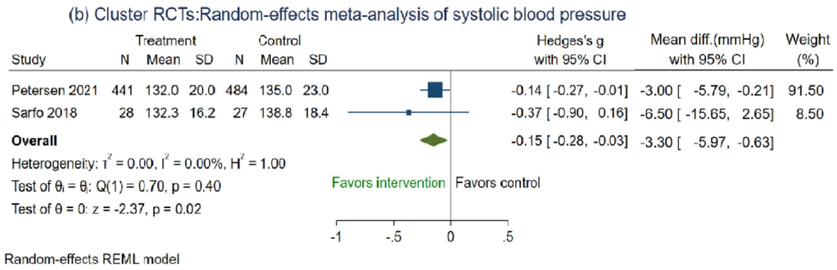




Otieno P, *et al. BMJ Open* 2023; 13:e073652. doi: 10.1136/bmjopen-2023-073652

**Online supplementary file 4: DOI plot of the random effects meta-analysis of SBP for integrated versus standard care for cardiometabolic multimorbidity in Sub-Saharan Africa**

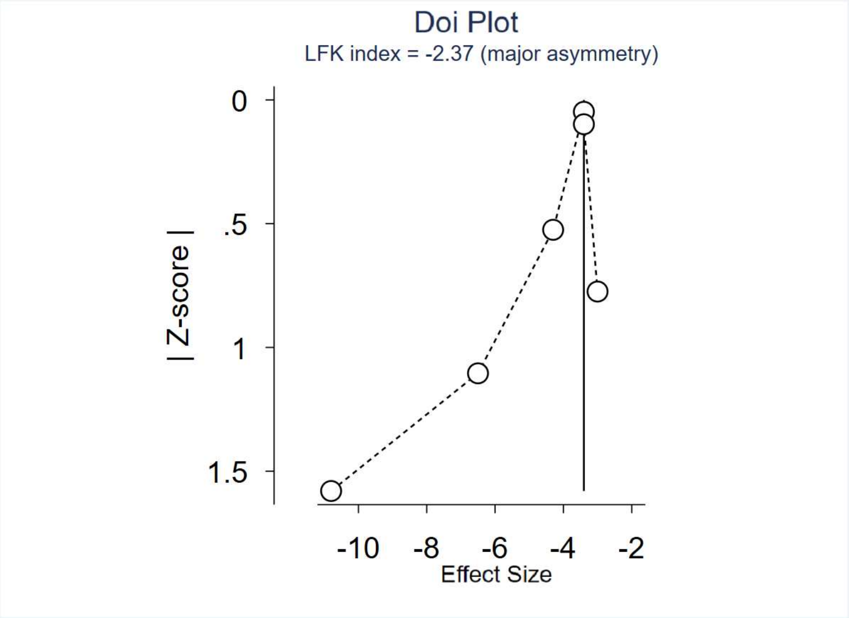




Otieno P, *et al. BMJ Open* 2023; 13:e073652. doi: 10.1136/bmjopen-2023-073652
