# Supplementary material for: Effectiveness of integrated chronic care models for cardiometabolic multimorbidity in sub-Saharan Africa: a systematic review and meta-analysis
Source: BMJ Open. 2023 Jun 27;13(6):e073652. doi: 10.1136/bmjopen-2023-073652 (PMC10410889; doi:10.1136/bmjopen-2023-073652)
Supplement: Author's manuscript [file bmjopen-2023-073652.draft_revisions.pdf]

BMJ Open is committed to open peer review. As part of this commitment we make the peer review history of every article we publish publicly available.

When an article is published we post the peer reviewers' comments and the authors' responses online. We also post the versions of the paper that were used during peer review. These are the versions that the peer review comments apply to.

The versions of the paper that follow are the versions that were submitted during the peer review process. They are not the versions of record or the final published versions. They should not be cited or distributed as the published version of this manuscript.

BMJ Open is an open access journal and the full, final, typeset and author-corrected version of record of the manuscript is available on our site with no access controls, subscription charges or pay-per-view fees (http://bmjopen.bmj.com).

If you have any questions on BMJ Open's open peer review process please email info.bmjopen@bmj.com

BMJ Open

Effectiveness of integrated chronic care models for cardiometabolic multimorbidity in Sub-Saharan Africa: A systematic review and meta-analysis

Journal:	*BMJ Open*
Manuscript ID	bmjopen-2023-073652
Article Type:	Original research
Date Submitted by the Author:	14-Mar-2023

Complete List of Authors:	Otieno, Peter; African Population and Health Research Center, Chronic Disease Management Uninit; Amsterdam UMC Locatie AMC, Department of Public & Occupational Health<br>Agyemang, Charles ; Amsterdam UMC Locatie AMC, Department of Public & Occupational Health<br>Wao, Hesborn; African Population and Health Research Center, Research and Related Capacity Strengthening<br>Wambiya, Elvis; The University of Sheffield, School of Health and Related Research<br>Ng'oda, Maurine; African Population and Health Research Center<br>Mwanga, Daniel; African Population and Health Research Center<br>Oguta, James; The University of Sheffield, School of Health and Related Research<br>Kibe , Peter M. ; African Population and Health Research Center<br>Asiki, G; African Population and Health Research Center, Chronic Disease Management Unit; Karolinska Institutet, Women's and Children's Health

Keywords:	Hypertension < CARDIOLOGY, Patient-Centered Care, Primary Health Care, Self Care

SCHOLARONE™
Manuscripts

I, the Submitting Author has the right to grant and does grant on behalf of all authors of the Work (as defined in the below author licence), an exclusive licence and/or a non-exclusive licence for contributions from authors who are: i) UK Crown employees; ii) where BMJ has agreed a CC-BY licence shall apply, and/or iii) in accordance with the terms applicable for US Federal Government officers or employees acting as part of their official duties; on a worldwide, perpetual, irrevocable, royalty-free basis to BMJ Publishing Group Ltd ("BMJ") its licensees and where the relevant Journal is co-owned by BMJ to the co-owners of the Journal, to publish the Work in this journal and any other BMJ products and to exploit all rights, as set out in our licence.

The Submitting Author accepts and understands that any supply made under these terms is made by BMJ to the Submitting Author unless you are acting as an employee on behalf of your employer or a postgraduate student of an affiliated institution which is paying any applicable article publishing charge ("APC") for Open Access articles. Where the Submitting Author wishes to make the Work available on an Open Access basis (and intends to pay the relevant APC), the terms of reuse of such Open Access shall be governed by a Creative Commons licence – details of these licences and which Creative Commons licence will apply to this Work are set out in our licence referred to above.

Other than as permitted in any relevant BMJ Author's Self Archiving Policies, I confirm this Work has not been accepted for publication elsewhere, is not being considered for publication elsewhere and does not duplicate material already published. I confirm all authors consent to publication of this Work and authorise the granting of this licence.

For peer review only

Effectiveness of integrated chronic care models for cardiometabolic multimorbidity in Sub-Saharan Africa: A systematic review and meta-analysis

Peter Otieno*[1,2,3]
Charles Agyemang[2]
Hesborn Wao[1]
Elvis Wambiya[5]
Maurine Ng'oda[1]
Daniel Mwanga[1]
James Oguta[5]
Peter Kibe[1]
Gershim Asiki[1,4]

1 African Population and Health Research Center P.O. Box: 10787-00100, Nairobi, Kenya.
2 Department of Public & Occupational Health, Amsterdam UMC, University of Amsterdam, Amsterdam Public Health Research Institute, Amsterdam, The Netherlands.
3 Amsterdam Institute for Global Health and Development (AIGHD), AHTC, Tower C4, Paasheuvelweg 25, 1105 BP Amsterdam, Netherlands.
4 Department of Women's and Children's Health, Karolinska Institutet, Stockholm, Sweden
5 School of Health and Related Research, University of Sheffield. Sheffield, S1 4DA, United Kingdom

* Corresponding author:
Peter Otieno
African Population and Health Research Center,
P.O. Box: 10787-00100, Nairobi, Kenya.
Email: pootienoh@gmail.com
Telephone: +254 (20) 400 1000

Word count: 3,997

For peer review only

For peer review only - http://bmjopen.bmj.com/site/about/guidelines.xhtml

Abstract

Objective: This review aimed at identifying the elements of integrated care models for cardiometabolic multimorbidity in Sub-Saharan Africa (SSA) and their effects on clinical or mental health outcomes including blood pressure, blood sugar, depression scores and other patient-reported outcomes such as quality of life, and medication adherence.

Design: We systematically searched PubMed, Embase, Scopus, Web of Science, Global Health CINAHL, African Journals Online, Informit, PsycINFO, clinicaltrials.gov, Pan African Clinical Trials Registry and grey literature from OpenSIGLE for studies published between 1999 and 2022. We included randomized controlled trial studies featuring integrated care models with two or more elements of Wagner's chronic care model.

Setting: We selected studies conducted in SSA among adults aged 18 years and over with cardiometabolic multimorbidity receiving care in primary or community care settings.

Outcomes: The outcome measures included systolic blood pressure (SBP), glycated haemoglobin (HbA1c), depression, medication adherence, and quality of life.

Results: In all, we included 10 randomized controlled trials from 11 publications with 4,864 participants from seven SSA countries (South Africa, Kenya, Nigeria, Eswatini, Ghana and Uganda). The overall quality of evidence based on GRADE criteria was moderate. A random-effects meta-analysis of six studies involving 1,754 participants shows that integrated compared to standard care conferred a significantly lower mean SBP (Mean Difference=-4.85mmHg, 95% CI: -7.37 to -2.34) for people with cardiometabolic multimorbidity; Hedges' g effect size (g=-0.25, (-0.39 to -0.11). However, integrated care compared with usual care showed mixed results for HbA1c, depression, medication adherence, and quality of life.

Conclusions: Integrated care improved SBP among patients living with cardiometabolic multimorbidity in SSA. More studies on integrated care are required to improve the evidence pool on chronic care models for multimorbidities. These include implementation studies, cost-effectiveness studies; and more trials with outcomes on mental health, medication adherence, and health-related quality of life.

Keywords: Integrated care, chronic care models, multimorbidity, cardiometabolic diseases, Sub-Saharan Africa

PROSPERO registration number: CRD42020187756

For peer review only

For peer review only - http://bmjopen.bmj.com/site/about/guidelines.xhtml

Article Summary:

Strengths and limitations of this study

- All the included studies in this review were randomized controlled trials. Hence, the findings are more unbiased than previous reviews on integrated care in low and middle-income countries that are mostly based on observational study designs.
- This study comprehensively extracted important constructs of Wagner's chronic care model, thus offering deeper insights into the effectiveness of its elements and delivering crucial evidence to researchers and health stakeholders for future improvement of integrated care models.
- The consideration of both communicable and non-communicable diseases in the review is also a strength due to the converging burdens of infectious and non-communicable diseases in Sub-Saharan Africa.
- The varying numbers and types of chronic care model components resulted in heterogeneity in the included studies. Furthermore, the absolute effect attributable to a particular element of the chronic care model remains unknown, as most included studies used multi-component interventions. However, the findings provide crucial insights for policymakers and health service providers on the effectiveness of integrated chronic care models for cardiometabolic multimorbidity in Sub-Saharan Africa.

For peer review only

For peer review only - http://bmjopen.bmj.com/site/about/guidelines.xhtml

Background

Cardiometabolic diseases, including type 2 diabetes and cardiovascular diseases (CVDs), are the leading cause of global mortality [1]. People living with cardiometabolic diseases often have multiple rather than a single condition, commonly known as multimorbidity [2]. Cardiometabolic multimorbidity occurs against the background of obesity and insulin resistance [3]. However, these conditions are also characterised by the simultaneous co-occurrence of other diseases with unrelated pathophysiology or care management profile such as HIV/AIDS, chronic respiratory diseases, cancers, and mental illnesses, a phenomenon referred to as discordant multimorbidity [4-6].

The burden of multimorbidity is highest in low- and middle-income countries (LMICs), which still bear a high burden of infectious diseases while also facing a new threat from non-communicable diseases [7]. However, the majority of the chronic care models in Sub-Saharan Africa (SSA) are still built around single disease frameworks [8]. Evidence shows that health services delivery based on isolated interventions may derail the value of treatment and preventive measures [9-12]. Vertical disease programs result in missed opportunities in the early diagnosis and management of multimorbidities [13]. Therefore, integrated chronic disease management is recommended to respond to this challenge [14]. Singer et al, (2011) [15], define *integrated care* as a set of patient-centred and multidisciplinary care activities coordinated by two or more collaborating service providers within or across the healthcare sector including community and social environments. This definition emphasizes the coordination of care activities and the active involvement of patients in managing their health.

The chronic care model, first identified by McColl Institute for Healthcare Innovation at Group Health Cooperative [11], has been proposed as a solution to improve the integrated management of chronic diseases. This model identifies six essential elements: community resources and policies, health care organization, self-management support, delivery system design, decision support and clinical information systems. However, what constitutes an integrated chronic care model and how it is implemented and delivered within healthcare services, has continued to evolve with the advent of new interventions. Most of the chronic care models have been developed in high-income countries with sophisticated health services [16], which may not appropriately apply to SSA.

The untapped capacity of chronic care models for HIV/AIDS care in SSA should be viewed as emerging models for other chronic diseases including cardiometabolic diseases [17]. Lessons learned from the implementation of care models for HIV/AIDS and tuberculosis in SSA, could be effectively applied to improve care models for cardiometabolic diseases [18]. A systematic review by Rohwer et al [19] examined

For peer review only - http://bmjopen.bmj.com/site/about/guidelines.xhtml

existing integrated care models for diabetes and hypertension in LMICs. However, the included studies did not focus on structured clinical care delivered to persons living with multimorbidity. Furthermore, the integrated care for hypertension and diabetes was based on a 'one-stop-shop' model where all healthcare services are provided under one roof [19]. This is just one way of describing integrated care. A comprehensive evaluation of the applicability of the elements of chronic care models for cardiometabolic multimorbidity has not been systematically evaluated in SSA. Therefore, the purpose of this systematic review was to identify elements of integrated chronic care models for cardiometabolic multimorbidity in SSA and their effects on clinical or mental health outcomes including blood pressure, blood sugar, depression scores and other patient-reported outcomes such as quality of life, and medication adherence.

Methods

Protocol registration

The study protocol was registered in the International Prospective Register for Systematic Reviews (PROSPERO) (CRD42020187756). The findings are reported according to the Preferred Reporting Items for Systematic Reviews (PRISMA) [20].

Eligibility criteria

Participants

We selected studies conducted in SSA among adult patients aged 18 years and over with cardiometabolic multimorbidity receiving care in a primary or community care setting. Cardiometabolic multimorbidity was defined as having two or more chronic conditions; at least one of which is a cardiometabolic disease such zas type 2 diabetes, hypertension, hypercholesterolemia, hypertriglyceridemia, dyslipidaemia or CVDs such as stroke. Others included common discordant conditions such as HIV/AIDS, chronic respiratory diseases, cancers and mental illnesses. These conditions constitute over three-quarters of the global burden of chronic diseases [21].

Intervention

We included interventions with integrated chronic care models for persons living with cardiometabolic multimorbidity. In line with previous studies [22-26], we classified the intervention components into six categories based on the elements of Wagner's chronic care model [11]. Thus, for the inclusion criterion, the models were considered "integrated care" if they applied at least two of the six chronic care model elements to manage more than one chronic disease [23]. This included self-management support, delivery system design, decision support, clinical information system, healthcare organization, and community linkages.

For peer review only - http://bmjopen.bmj.com/site/about/guidelines.xhtml

The classification of intervention features and components is shown in Supplementary file 1. Self-management support includes interventions that empower people living with chronic conditions to manage their health. Examples are self-help groups and technological aids for self-care. Delivery system design interventions focus on care delivery efficiency and effectiveness such as patient care planning, coordination and follow-up. Decision-support interventions promote clinical care that is consistent with scientific evidence and patient preferences. Clinical information systems is the organization of patient data to facilitate efficient and effective care. Healthcare organization interventions focus on creating an organizational culture and mechanisms that promote safe, high-quality care. Community linkages include interventions that mobilize community resources to meet the needs of people living with chronic diseases. Where the chronic care model had multiple components, we defined each element using the Wagner taxonomy [11] and highlighted the predominant components.

Control

The control or comparison group in the included studies received the usual or standard care comprising standalone services for cardiometabolic multimorbidity.

Outcome

We included studies that reported any objective measure of patient clinical or mental health outcomes, for example, blood pressure (mmHg), glycated haemoglobin ((HbA1c (%)), and depression scores. Others included patient-reported outcomes such as quality of life, and patient behaviour (medication adherence).

Types of studies

We only included randomised controlled trials (RCTs) meeting the quality criteria developed by the Cochrane Effective Practice and Organization of Care (EPOC) [27]. This is because RCTs provide more unbiased information than other study designs on the differential effects of chronic care models. Studies from SSA published in all languages between 1999 and December 2022 were included. We used 1999 because this was the formal year of inception of the Wagner's chronic care model [28].

Exclusion criteria

We excluded single-disease-focused studies and studies conducted among pregnant women. Interventions with no specified structured clinical care delivered to persons living with cardiometabolic multimorbidity were excluded. Studies with interventions for comorbid conditions where the intervention was targeted solely at one of the conditions rather than integrated care for the co-occurring conditions were also excluded. Studies that

For peer review only - http://bmjopen.bmj.com/site/about/guidelines.xhtml

assumed multimorbidity to be the norm based on individuals' age without screening and diagnosis by qualified healthcare providers were excluded as the interventions were not being targeted specifically at multimorbidity.

Search strategy

Two reviewers independently searched PubMed, Embase, Scopus, Web of Science, Cumulative Index to Nursing and Allied Health Literature (CINAHL), Cochrane library, African Journals Online, Informit, and PsycINFO without language restriction. The search was limited to articles indexed from 1999 up to 15th December 2022 to capture the beginning of the application of the elements of Wagner's chronic care model. The search results were limited by filters for randomized control trials and search concepts for chronic care models, cardiometabolic diseases and multimorbidity (See supplementary file 2). Additional articles were identified by scanning the reference list of relevant studies obtained through the search. Hand-searching of key journals was also conducted. Furthermore, we searched for registered trials in the clinicaltrials.gov, the Pan African Clinical Trials Registry and grey literature from OpenSIGLE.

Data collection and analysis

Selection of studies

The search results were uploaded into the Endnote version 12 reference manager (Clarivate, Philadelphia, USA) and screened for duplicates. Three review authors (EW, MN, and DM) independently performed the initial screening of titles and abstracts. The articles selected for full-text review were subsequently assessed for adequacy following the inclusion criteria for patient, intervention, comparator and outcome. Consensus meetings were held between the reviewers and the project coordinator (PO) to resolve disagreements. We used a modified PRISMA flow chart to describe the study selection (Figure 1).

Data extraction and management

Three review authors (EW, MN, and DM) abstracted data using a modified version of the EPOC data collection checklist [27]. Data quality disagreements were resolved by consensus between the review authors and the project coordinator (PO). Information extracted from the included studies included:

1. Participants: type of patients, age, nature of multimorbidity and how it was diagnosed.
2. Study design: randomization, unit of allocation, and follow-up.
3. Intervention: we extracted a full description of the intervention components and features. This included self-management support, delivery system design, decision support, clinical information system, healthcare organization, and community resources
4. Service providers: specialists, primary care providers, and family members
5. Study setting: hospital, primary care and community-based care

For peer review only - http://bmjopen.bmj.com/site/about/guidelines.xhtml

6. Patient clinical or mental health outcomes such as blood pressure, blood glucose control, and depression symptom scores. Others included patient-reported outcomes such as quality of life and medication adherence.

7. Results: We organized results into clinical outcomes, mental health outcomes and patient-reported outcomes.

Assessment of risk of bias

The risk of bias in the included studies was independently assessed by three review authors (EA, MN, and DM) using standard EPOC criteria. The assessment domains included: allocation (sequence generation and concealment); baseline characteristics; incomplete outcome data; contamination; blinding; selective outcome reporting; and other potential sources of bias. The criteria for the assessment were 'low risk', 'high risk' or 'unclear risk' (Figure 2). Loss to follow-up of more than 20% was considered a high risk of bias. We also considered loss to follow-up as a high risk of bias when it was unbalanced between the groups. Published protocols and trial registrations were tracked to assess selective reporting bias. We assessed incomplete reporting for studies that reported different results than the outcomes outlined in the methods sections of selected articles. Any disagreements between the review authors were resolved by consensus, or with the consultation of the project coordinator (PO).

Data analysis

We used a random-effects meta-analysis to estimate the pooled mean effect size of integrated care versus standard care on systolic blood pressure (SBP). The random-effects meta-analysis allows for statistical heterogeneity between studies by assuming that the true effects in the individual studies differ from each other. Six studies with similarities in terms of patient population, interventions, and outcome assessment, were included in the meta-analysis. Studies with significant heterogeneity were not included in the meta-analysis. Hence, we conducted a structured synthesis of the results. All analyses were performed using "*metan'*" command in Stata version 15 (StataCorp LLC).

We calculated the effect size following the methods described by Hedges [29]. The heterogeneity of the selected studies were evaluated using χ^2 and I^2 statistics [30]. Using the χ^2 test, heterogeneity between studies was considered significant if $p<0.10$ [31]. We used I^2 statistics, values of 25%-49%, 50%-74% and >75% to determine low, moderate and high heterogeneity respectively [32]. Publication bias was explored using a funnel plot and Egger's test [33].

For peer review only - http://bmjopen.bmj.com/site/about/guidelines.xhtml

Certainty of evidence

The certainty of the evidence of included studies was assessed according to the Grading of Recommendations Assessment, Development and Evaluation (GRADE) methodological guideline. According to GRADE guidance, RCTs start with high-certainty evidence [34]. Our judgements to downgrade the certainty of evidence were based on the assessment of the following five domains: study limitations, inconsistency, imprecision, indirectness and publication bias. For each outcome, we described the certainty of evidence as high, moderate, low or very low. The GRADE criteria also take into consideration the certainty of evidence and the size of the effect [35]. We considered a 5 mmHg reduction in SBP to be clinically significant [36]. A change of 0.5% for the outcome HbA1c, and 10% for depression scores, medication adherence and quality of life were considered clinically significant. For each outcome, we described the certainty of evidence as high, moderate, low or very low [37].

Patient and public involvement

Patients or the public were not involved in the design, conduct, reporting, or dissemination of this research.

Results

Search results

The PRISMA flow diagram of the article selection process is presented in figure 1. We identified 2,649 studies from our searches. Another 17 studies were included after manually searching the reference list and grey literature. After the removal of 806 duplicate records, we screened the titles and abstracts of 1,860 articles and identified 32 potentially eligible studies. We reviewed the full text of 32 studies and excluded 22 studies. Thus, 10 studies were included in this review (6 randomized controlled trials and 4 cluster randomized trials). Of these 10 studies, 6 were included in the meta-analyses and 4 studies were included in the structured synthesis of the results.

[Figure 1 approximately here]

Characteristics of included studies

The characteristics of the included studies is presented in table 1. We included 10 trials from 11 publications [38-48] with a total of 4,864 participants. Sample sizes ranged from 55 to 1,174 participants. Three studies were conducted in South Africa, two in Kenya and Nigeria and one in Eswatini and Ghana, respectively. There was one multi-country study conducted in Uganda and Kenya. Studies mostly took place in primary care settings (n=9) or both community and primary care settings (n=2). Most trials recruited participants

For peer review only - http://bmjopen.bmj.com/site/about/guidelines.xhtml

with HIV and cardiometabolic diseases (n=9), another two recruited patients with HIV, type 2 diabetes, hypertension, depression and substance use disorder. Eight studies lasted 12 months or more. In all the included studies, the control group received the usual standard care while the intervention group received structured integrated care with varying elements of chronic care models including self-management support, delivery system design, decision support, clinical information system, healthcare organization, and community resources.

For peer review only

For peer review only - http://bmjopen.bmj.com/site/about/guidelines.xhtml

Table 1: Characteristics of included studies

Study	Setting	Study design	Sample size	Age, years	Multimorbidity	Follow-up (months)	Outcome	Intervention
Havlir et al (2019) [38] Uganda and Kenya	Primary and community-based care	Cluster RCT	1,441 patients with hypertension & HIV	≥30	Hypertension and HIV	36	BP control	Multi-disease testing; integrated care for HIV and hypertension; structured follow-up, flexible hours of operation and reduced wait time at clinics; and patient incentives
Jackson et al (2021) [39] Nigeria	Primary care	RCT	182	18-69	Hypertension & HIV	12	SBP and medication adherence	Pharmaceutical care with structured education and counselling by a pharmacist after seeing a physician on their clinic visit. Education focused on self-monitoring of BP, lifestyle modification, reviewing the date of the next appointment and prescription
Jackson et al (2022) [40] Nigeria	Primary care	RCT	182	18-69	Hypertension and HIV	12	Health-related quality of life	Structured education and counselling on general self-care, medicines use storage, and lifestyle modifications
Myers et al (2022) [41] South Africa	Primary care	Cluster RCT	1,174	≥18	HIV, Type 2 diabetes, depression and substance use disorder	12	Depression scores	Psychological interventions comprising motivational interviewing and problem-solving therapy delivered by a trained facility-based community health worker.
Okube et al (2022) [42] Kenya	Community-based care	RCT	294	18-64	Metabolic syndrome	12	BP and blood sugar control	Community-based health education on lifestyle modification and face-to-face delivery of verbal and written individualized health recommendations on risk factors for CVDs
Owolabi et al 2019 [43] Nigeria	Primary care	RCT	158	≥18	Hypertension and stroke	12	SBP	A culturally appropriate, multipronged intervention comprising patient global risk factor control report card, personalized phone text-messaging, and educational video
Petersen et al (2021) [44] South Africa	Primary care	Cluster RCT	925	≥18	Hypertension and depression	12	Depression scores	Supplementary training of primary nurses and doctors on mental health and clinical communication skills. Collaborative care model for patients with hypertension and comorbid depressive symptoms including doctors, nurses, clinical psychologist and lay counsellors
Rabkin et al (2018) [45] Eswatini	Primary care	RCT	236	≥40	HIV, Hypertension, type 2 diabetes, and hyperlipidemia	6	SBP and HbA1c	CVD risk factors screening and structured referrals among patients living with HIV.
Roos et al 2014 [46] South Africa	Primary care	RCT	84	20-65	HIV and metabolic diseases	12	SBP and fasting blood sugar	Pedometer and a physical activity diary with education materials and self-monitoring documents. Structured regular clinic sessions for review of physical activity diary and risk factors for ischemic heart disease. Monthly SMS text motivational messages.
Sarfo et al (2018) [47] Ghana	Primary care	Cluster RCT	55	≥18	Hypertension and stroke	9	BP control and medication adherence	Blue-toothed BP device and smartphone for self-monitoring and reporting BP measurements and medication intake. Tailored motivational text messages delivered based on the levels of adherence to the medication intake protocol
Thuita et al 2020 Kenya [48]	Primary care	RCT	133	20–79	Metabolic syndrome	6	SBP and HbA1c	Integrated care with nutrition education and peer-to peer support

*The control group received standard/usual care

For peer review only - http://bmjopen.bmj.com/site/about/guidelines.xhtml

Risk of bias in included studies

The risk of bias summary is presented in figure 2. All the included studies were RCTs. Hence, all studies had low risk for randomization. Six studies were appraised as unclear risk due to insufficient information about protection against contamination [39, 42, 43, 45, 46, 48]. One study was rated as unclear risk due to lack of sufficient information about allocation concealment. Although it was not feasible to blind the participants and study personnel due to the nature of the interventions, the absence of blinding did not affect the objective outcomes. Hence, all studies were graded as low risk for blinding. Under the reliability of primary outcomes, five studies were rated as high risk due to small sample sizes [43, 46-48] and lack of baseline measurements [38]. For selective reporting, all the studies were assessed as low risk, except one that lacked clarity and was assessed as unclear risk [45]. Four studies were rated high risk for other biases, mainly short follow-up duration (less than one year) [43, 45, 47, 48]. Overall, the certainties of the evidence for SBP, HbA1c, depression, medication adherence and quality of life were downgraded to moderate. This was due to the high risk of bias, imprecision and clinically insignificant effect sizes in the included studies [38-48].

[Figure 2 approximately here]

Components of the chronic care model

Table 2 presents the components of the chronic care model in the included studies. All studies had to include at least two components of the Wagner chronic care model to be defined as "integrated care" for the management of at least two chronic diseases. The number of chronic care elements ranged from two to five. One study included five of the six elements of the chronic care model [38]. Three studies included three elements [43, 46, 47] and six enclosed two elements [41, 44, 45, 48-50].

All the studies included delivery system design except one. The components of self-delivery included multi-disease screening and treatment, task shifting, structured follow-up, and collaborative care. Seven studies included self-management support such as home-based self-monitoring, lifestyle counselling, post-clinic patient follow-up and patient support groups [38, 39, 43, 46-48, 50]. Four studies included decision support components such as supplementary training of healthcare workers and support supervision [38, 41, 44, 45]. ‘Three studies included clinical information system [43, 46, 47]. The elements enclosed comprise hospital registries, patient diaries, report cards and electronic platforms for BP monitoring. Two studies included community resource elements to enhance health campaigns [38, 50]. One study included the healthcare organization element, mainly quality improvement strategies to enhance access to medication and treatment services [38].

For peer review only - http://bmjopen.bmj.com/site/about/guidelines.xhtml

Table 2: Elements of the chronic care model included in the studies

Author (year)	Self-management support	Delivery system design	Decision support	Clinical information system	Healthcare organization	Community resources
Havlir et al (2019) [38]	Enhanced lifestyle modification counselling in primary care centers	Point-of-care multi-disease screening for HIV, hypertension, and diabetes with structured follow-up and care linkages	Telephone and in-person oversight from a senior physician on the services provided by general physicians and nurses.		Quality improvement strategies such as guaranteed access to medication, flexible hours of operations, and reduced wait time at clinics	Multi-disease testing community health campaigns using community resource persons
Jackson et al (2021) [39]	Education on self-monitoring of BP, lifestyle modification, self-care and appropriate use of medicines	Structured pharmaceutical care including prescription review and follow-up				
Myers et al (2022) [40]		Task shifting and empowerment of community health workers to provide basic psychological interventions	Trained community health workers on basic psychological intervention including motivational interviewing and problem-solving therapy			
Okube et al (2022) [42]	Individualized health recommendations on CVD risk factors					Community-based health education on lifestyle modification
Owolabi et al 2019 [43]	Post-clinic follow-up phone texts and waiting room educational video	Enhanced follow-up visits and pre-appointment phone texts		Hospital registry and patient report card as part of medical chart		
Petersen et al (2021) [44]		Collaborative care model for patients with hypertension and comorbid depressive symptoms including doctors, nurses, clinical psychologist and lay counsellors	Supplementary training of primary care nurses and doctors on mental health and clinical communication skills			
Rabkin et al (2018) [45]	-	One stop shop for CVD risk factors screening and structured referrals among patients living with HIV	Training of HIV clinic nurses and doctors to conduct CVD risk factors screening during routine clinic appointments of patients			
Roos et al (2014) [46]	A pedometer and a physical activity diary with education materials and self-monitoring documents. Monthly SMS text motivational messages	Structured regular clinic sessions for review of physical activity diary and risk factors for ischemic heart disease		Patient diary for self-monitoring of risk factors for ischemic heart disease		
Sarfo et al (2018) [47]	Self-monitoring of BP using a blue-toothed device. Tailored motivational text messages delivered based on the levels of adherence to medication	Structured follow-up for BP measurements and medication adherence		Digital platform for tracking BP measurements and medication adherence		
Thuita et al 2020 [48]	Nutrition counselling and peer support group	Monthly follow up visits and structured regular clinic sessions to review patient progress. Facility-based patient support groups.				

.

For peer review only - http://bmjopen.bmj.com/site/about/guidelines.xhtml

Effectiveness of integrated models of care compared with standard care

Blood pressure

Figure 3 presents the random effects meta-analysis of SBP. The pooled results of 6 studies [39, 42-44, 47, 48] involving 1,754 participants show that integrated versus standard care conferred a lower mean SBP (Mean Difference (MD)=-4.85mmHg, 95% CI (-7.37 to -2.34) with a small Hedges' g effect size (g=-0.25, (-0.39 to -0.11). However, the overall quality of evidence based on GRADE criteria was moderate. Moderate heterogeneity (I^2=39.57%) existed in the included studies (Figure 3). A funnel plot of the random effects meta-analysis of SBP for integrated versus standard care is shown in Supplementary file 3. The absence of publication bias is suggested by the symmetrical visual inspection of the funnel plot, confirmed by Egger's test (p<0.001).

[Figure 3 approximately here]

We did not conduct meta-analyses for the four of the included studies in this review [38, 41, 45, 46], as they were too heterogeneous in terms of, patient population, interventions, and outcome assessment. Hence, we conducted a structured synthesis of the results. Of the studies included in the structured synthesis of the results, the effect of integrated versus standard care on BP were reported in three studies [38, 45, 46]. However, the quality of evidence based on GRADE criteria was moderate. The SEARCH trial conducted in Kenya and Uganda found that hypertension control was 22% higher among patients on integrated care versus standard care (relative prevalence=1.22; (1.08 to 1.37)) [38]. In contrast, a study by Rabkin et al [45] conducted in Eswatini found no significant effect of integrated versus referred management of CVD risk factors on mean SBP. Another study by Roos et al (2014) also found no significant effect of education and home-based pedometer walking program on SBP MD=2.50 (-2.78, to 7.78).

Fasting blood glucose and HbA1c

Two studies included in the structured synthesis of the results assessed the effect of integrated versus standard care on FBS and HbA1c [42, 45]. A study by Okube et al [42] found that integrated care conferred a significant ($p < 0.05$) reduction in FBS by -0.5mmol/L versus +0.08 mmol/L among patients on standard care. In a study by Rabkin et al [45] the mean of HbA1c was significantly reduced by -0.7% (95%CI: -1.3 to -0.1) and -1.4% (-2.5, -0.2) in the integrated and standard care arms, respectively with no statistical difference between arms.

Depression

The effect of integrated versus standard care on depression was reported in two studies. A study by Petersen et al [44] found no significant difference in the depression scores in patients on integrated versus standard

For peer review only - http://bmjopen.bmj.com/site/about/guidelines.xhtml

care (adjusted risk difference = -0.04 (-0.19; 0.11)). By contrast, the results of the study by Myers et al [41] found significant differences in the depression scores in patients on integrated versus standard care (MD = 4.8(-7.2 to -2.4)).

Medication adherence and quality of life

Two studies assessed the effect of integrated care on hypertension medication adherence [40, 45]. One study reported the effect of integrated versus standard care HRQoL [40]. In a study by Rabkin et al [45], integrated care conferred a higher likelihood of hypertension medication adherence than standard care (relative risk=1.28 [1.10, 1.47])). A study by Jackson et al [40] found that integrated care led to significant improvements in hypertension medication adherence (Mean Difference in Difference (DiD)= 2.32; $p < 0.001$) and HRQoL (DiD= 6.5%, $P < 0.001$).

.

Discussions

This study presents the results of a systematic review of integrated care models for cardiometabolic multimorbidity and their effects on intermediate health outcomes in SSA. Our results show that, in comparison to usual care, integrated care featuring at least two elements of Wagner's chronic care model conferred significant improvements in SBP among patients living with cardiometabolic multimorbidity. The overall quality of evidence was moderate. Results of the systematic review suggest that integrated care compared with usual care have mixed results with regards to HbA1c, depression, medication adherence and quality of life, with some studies showing a significant effect and some no effect.

The findings of the current review based on RCTs are broadly consistent with reviews on the effectiveness of the integrated chronic care model from pervious observational studies [51, 52]. In contrast, one systematic review by Rohwer et al [19] on integrated care models for diabetes and hypertension in LMICs found no evidence of improved blood pressure or diabetes control. However, the evidence presented had very low certainty and the included studies that did not focus on care models for persons living with multimorbidity.

Our study used the Wagner's chronic care model, a framework for improving the integrated management of chronic diseases through the implementation of its six core elements: community resources and policies, health care organization, self-management support, delivery system design, decision support and clinical information systems [11]. A review by Goh et al [51] found greater improvements in HbA1c for care models with more elements over a single element. However, most of the studies included in the current review had two to three elements. This could partly explain the mixed results with regards to effect of care integration

For peer review only - http://bmjopen.bmj.com/site/about/guidelines.xhtml

on intermediate health outcomes such as HbA1c, depression, medication adherence and quality of life, with some studies showing a significant effect and some no effect.

In the current review, delivery system design and self-management support were the most predominant components of the integrated chronic care models while healthcare organization and community resource elements were less common. Nevertheless, the structured synthesis of the results shows no significant differences in health outcomes by the elements of care integration since a majority of studies included two to three elements. Previous studies have shown that intervention intensity rather than the number of intervention components may have an impact on the direction and magnitude of the health outcomes [52, 53].

Strengths and limitations

The current review has three main strengths. First, all the included studies were RCTs. Hence, the findings are more unbiased than previous reviews on integrated care in LMICs that are mostly based on observational study designs. Second, this study comprehensively extracted important constructs of Wagner's chronic care model, thus offering deeper insights into the effectiveness of its elements and delivering crucial evidence to researchers and health stakeholders for future improvement of integrated care models. The consideration of both communicable and non-communicable diseases in the review is also a strength due to the converging burdens of infectious and non-communicable diseases in SSA [7].

The findings of this review should be interpreted in light of a few limitations. First, the majority of the included studies did not classify the components of chronic care models in the interventions. Although the three reviewers independently classified the type of chronic care model elements in the studies using a standard guide, this could still lead to misclassification bias. Second, the varying numbers and types of chronic care model components added to the heterogeneity. Furthermore, the absolute effect attributable to a particular element of the chronic care model remains unknown, as most included studies used multi-component interventions. Third, it may not be possible to have a standard of care that is similar for all the studies included in this review. This resulted in a broad variety of usual care. Lastly, due to the substantial heterogeneity in the studies, in terms of multimorbidity, interventions, follow-up periods, and outcome assessment, six of the ten studies were included in the meta-analysis. The rest of the studies were included in the structured synthesis of the results.

For peer review only - http://bmjopen.bmj.com/site/about/guidelines.xhtml

Conclusions

We found that integrated care may lead to moderate improvements in SBP among patients living with cardiometabolic multimorbidity in SSA. This review highlights the paucity of research on care integration interventions for cardiometabolic multimorbidity in SSA since the selected RCTs were from only a few SSA countries. In addition, there were fewer studies with outcomes on health-related quality of life, mental health, and medication adherence. The small number of studies addressing healthcare organisation and community resource elements provide less certainty on the benefits associated with these components. More studies are needed on the implementation effectiveness of integrated care models and its impact on cardiometabolic multimorbidity. The relative effectiveness of the different elements of integrated chronic care models and the cost-effectiveness of these models for the management of cardiometabolic multimorbidity should be explored. Other elements that are not well explored such as healthcare organisation and community resource elements should be investigated in future research. Future studies investigating the effectiveness of integrated care should classify the elements in the interventions and standardize the description of each element.

Acknowledgements: Not applicable.

Author contributions: PO conceptualized the study, reviewed the literature, and analysed the data. All authors contributed to the development and review of the protocol. EW, MN, and DM screened titles and abstracts and participated in full-text screening; PO helped to resolve discrepancies. EW, MN, and DM extracted data and assessed the risk of bias. PO assessed the certainty of evidence with input from all the authors. CA, HW and GA provided overall methodological guidance. JO and PK critically read and revised the manuscript. All authors have approved the final version of the manuscript.

Funding: This systematic review was supported by funding from the Joep Lange Institute and in part by the Dutch Ministry of Foreign Affairs under the Joep Lange Chairs and Fellows Program. The funders had no role in the design of the study and collection, analysis, and interpretation of data and in writing the manuscript.

Disclaimer: The funder did not have any role in the review process.

Competing interests: None declared.

Patient consent for publication: Not required.

For peer review only

Ethics approval: This systematic review does not involve human participants. All data included are in the public domain and ethics approval was thus not sought.

Data availability statement: All data relevant to the study are included in the article or uploaded as online supplemental information.

References

1. Roth, G.A., et al., *Global burden of cardiovascular diseases and risk factors, 1990–2019: update from the GBD 2019 study.* Journal of the American College of Cardiology, 2020. **76**(25): p. 2982-3021.
2. Violan, C., et al., *Prevalence, determinants and patterns of multimorbidity in primary care: a systematic review of observational studies.* PloS one, 2014. **9**(7).
3. Arenillas, J.F., M.a.A. Moro, and A. Dávalos, *The metabolic syndrome and stroke: potential treatment approaches.* Stroke, 2007. **38**(7): p. 2196-2203.
4. Mannino, D.M., et al., *Prevalence and outcomes of diabetes, hypertension and cardiovascular disease in COPD.* European Respiratory Journal, 2008. **32**(4): p. 962-969.
5. Metra, M., et al., *Cardiovascular and noncardiovascular comorbidities in patients with chronic heart failure.* Journal of Cardiovascular Medicine, 2011. **12**(2): p. 76-84.
6. Scott, K.M., *Depression, anxiety and incident cardiometabolic diseases.* Current opinion in psychiatry, 2014. **27**(4): p. 289-293.
7. Coates, M.M., et al., *Burden of disease among the world's poorest billion people: An expert-informed secondary analysis of Global Burden of Disease estimates.* Plos one, 2021. **16**(8): p. e0253073.
8. Guthrie, B., et al., *Adapting clinical guidelines to take account of multimorbidity.* Bmj, 2012. **345**: p. e6341.
9. Nolte, E. and M. McKee, *Caring for people with chronic conditions: a health system perspective*. 2008: McGraw-Hill Education (UK).
10. Boult, C., et al., *Early effects of "Guided Care" on the quality of health care for multimorbid older persons: a cluster-randomized controlled trial.* J Gerontol A Biol Sci Med Sci, 2008. **63**(3): p. 321-327.
11. Wagner, E.H., *Chronic disease management: what will it take to improve care for chronic illness?* Effective clinical practice, 1998. **1**(1).
12. World Health Organization, *Towards people-centred health systems: An innovative approach for better health outcomes*, in *World Health Organization Regional Office for Europe, Division of Health Systems and Public Health. Geneva: Switzerland: WHO Press*. 2013.
13. Kamkuemah, M., B. Gausi, and T. Oni, *Missed opportunities for NCD multimorbidity prevention in adolescents and youth living with HIV in urban South Africa.* BMC Public Health, 2020. **20**(1): p. 821.
14. Barr, A.L., et al., *The need for an integrated approach for chronic disease research and care in Africa.* Global health, epidemiology and genomics, 2016. **1**.
15. Singer, S.J., et al., *Defining and measuring integrated patient care: promoting the next frontier in health care delivery.* Medical Care Research and Review, 2011. **68**(1): p. 112-127.
16. Battersby, M.W., *Health reform through coordinated care: SA HealthPlus.* Bmj, 2005. **330**(7492): p. 662-665.
17. Geldsetzer, P., K. Ortblad, and T. Bärnighausen, *The efficiency of chronic disease care in sub-*

For peer review only - http://bmjopen.bmj.com/site/about/guidelines.xhtml

Saharan Africa. BMC medicine, 2016. **14**(1): p. 127.
18. Harries, A.D., et al., *Adapting the DOTS framework for tuberculosis control to the management of non-communicable diseases in sub-Saharan Africa.* PLoS medicine, 2008. **5**(6).
19. Rohwer, A., et al., *Effects of integrated models of care for diabetes and hypertension in low-income and middle-income countries: a systematic review and meta-analysis.* BMJ open, 2021. **11**(7): p. e043705.
20. Shamseer, L., et al., *Preferred reporting items for systematic review and meta-analysis protocols (PRISMA-P) 2015: elaboration and explanation.* Bmj, 2015. **349**.
21. Naghavi, M., et al., *Global, regional, and national age-sex specific mortality for 264 causes of death, 1980–2016: a systematic analysis for the Global Burden of Disease Study 2016.* The lancet, 2017. **390**(10100): p. 1151-1210.
22. Busetto, L., et al., *Intervention types and outcomes of integrated care for diabetes mellitus type 2: a systematic review.* Journal of evaluation in clinical practice, 2016. **22**(3): p. 299-310.
23. Desmedt, M., et al., *Economic impact of integrated care models for patients with chronic diseases: a systematic review.* Value in Health, 2016. **19**(6): p. 892-902.
24. Drewes, H.W., et al., *The effectiveness of chronic care management for heart failure: meta-regression analyses to explain the heterogeneity in outcomes.* Health services research, 2012. **47**(5): p. 1926-1959.
25. Elissen, A.M., et al., *Meta-analysis of the effectiveness of chronic care management for diabetes: investigating heterogeneity in outcomes.* Journal of Evaluation in Clinical Practice, 2013. **19**(5): p. 753-762.
26. Meeuwissen, J.A., et al., *Meta-analysis and meta-regression analyses explaining heterogeneity in outcomes of chronic care management for depression: implications for person-centered mental healthcare.* International Journal of Person Centered Medicine, 2012. **2**(4): p. 716-758.
27. Effective Practice and Organisation of Care (EPOC), *What study designs should be included in an EPOC review? EPOC Resources for review authors*. 2013, Norwegian Knowledge Centre for the Health Services: Oslo.
28. Wagner, E.H., et al., *Improving chronic illness care: translating evidence into action.* Health affairs, 2001. **20**(6): p. 64-78.
29. Hedges, L.V. and I. Olkin, *Statistical methods for meta-analysis*. 2014: Academic press.
30. Deeks, J.J., et al., *Analysing data and undertaking meta-analyses.* Cochrane handbook for systematic reviews of interventions, 2019: p. 241-284.
31. Hoaglin, D.C., *Misunderstandings about Q and 'Cochran's Q test'in meta-analysis.* Statistics in medicine, 2016. **35**(4): p. 485-495.
32. Higgins, J.P., et al., *Measuring inconsistency in meta-analyses.* Bmj, 2003. **327**(7414): p. 557-560.
33. Egger, M., et al., *Bias in meta-analysis detected by a simple, graphical test.* Bmj, 1997. **315**(7109): p. 629-634.
34. Guyatt, G., et al., *GRADE guidelines: 1. Introduction—GRADE evidence profiles and summary of findings tables.* Journal of clinical epidemiology, 2011. **64**(4): p. 383-394.
35. Santesso, N., et al., *GRADE guidelines 26: informative statements to communicate the findings of systematic reviews of interventions.* Journal of clinical epidemiology, 2020. **119**: p. 126-135.
36. Rahimi, K., et al., *Pharmacological blood pressure lowering for primary and secondary prevention of cardiovascular disease across different levels of blood pressure: an individual participant-level data meta-analysis.* The Lancet, 2021. **397**(10285): p. 1625-1636.
37. Puhan, M.A., et al., *A GRADE Working Group approach for rating the quality of treatment effect estimates from network meta-analysis.* Bmj, 2014. **349**.
38. Havlir, D.V., et al., *HIV testing and treatment with the use of a community health approach in*

For peer review only - http://bmjopen.bmj.com/site/about/guidelines.xhtml

rural Africa. New England Journal of Medicine, 2019. **381**(3): p. 219-229.

39. Jackson, I.L. and C.V. Ukwe, *Clinical outcomes of pharmaceutical care intervention in HIV positive patients with hypertension: a randomized controlled study.* Journal of Clinical Pharmacy and Therapeutics, 2021. **46**(4): p. 1083-1094.
40. Jackson, I.L. and C.V. Ukwe, *Effects of pharmaceutical care interventions on humanistic outcomes in hypertensive people living with HIV: results of a randomized controlled trial.* International Journal of Pharmacy Practice, 2022. **30**(3): p. 261-267.
41. Myers, B., et al., *Comparing dedicated and designated approaches to integrating task-shared psychological interventions into chronic disease care in South Africa: a three-arm, cluster randomised, multicentre, open-label trial.* The Lancet, 2022. **400**(10360): p. 1321-1333.
42. Okube, O.T., S. Kimani, and W. Mirie, *Community-based lifestyle intervention improves metabolic syndrome and related markers among Kenyan adults.* Journal of Diabetes & Metabolic Disorders, 2022. **21**(1): p. 607-621.
43. Owolabi, M.O., et al., *Randomized trial of an intervention to improve blood pressure control in stroke survivors.* Circulation: Cardiovascular Quality and Outcomes, 2019. **12**(12): p. e005904.
44. Petersen, I., et al., *Effectiveness of a task-sharing collaborative care model for identification and management of depressive symptoms in patients with hypertension attending public sector primary care clinics in South Africa: pragmatic parallel cluster randomised controlled trial.* Journal of Affective Disorders, 2021. **282**: p. 112-121.
45. Rabkin, M., et al., *Integrated vs. referred management of CVD risk factors for HIV positive patients on antiretroviral therapy in Swaziland.* 2018.
46. Roos, R., et al., *Effects of an education and home-based pedometer walking program on ischemic heart disease risk factors in people infected with HIV: a randomized trial.* JAIDS Journal of Acquired Immune Deficiency Syndromes, 2014. **67**(3): p. 268-276.
47. Sarfo, F.S., et al., *Phone-based intervention for blood pressure control among Ghanaian stroke survivors: a pilot randomized controlled trial.* International Journal of Stroke, 2019. **14**(6): p. 630-638.
48. Thuita, A.W., et al., *Effect of a nutrition education programme on the metabolic syndrome in type 2 diabetes mellitus patients at a level 5 Hospital in Kenya:"a randomized controlled trial".* BMC nutrition, 2020. **6**: p. 1-14.
49. Jackson, I.L. and C.V. Ukwe, *Effects of pharmaceutical care interventions on humanistic outcomes in hypertensive people living with HIV: results of a randomized controlled trial.* International Journal of Pharmacy Practice, 2022.
50. Okube, O.T., S. Kimani, and W. Mirie, *Community-based lifestyle intervention improves metabolic syndrome and related markers among Kenyan adults.* Journal of Diabetes & Metabolic Disorders, 2022: p. 1-15.
51. Goh, L.H., et al., *Effectiveness of the chronic care model for adults with type 2 diabetes in primary care: a systematic review and meta-analysis.* Systematic Reviews, 2022. **11**(1): p. 1-23.
52. Si, D., R. Bailie, and T. Weeramanthri, *Effectiveness of chronic care model-oriented interventions to improve quality of diabetes care: a systematic review.* Primary Health Care Research & Development, 2008. **9**(1): p. 25-40.
53. Weingarten, S.R., et al., *Interventions used in disease management programmes for patients with chronic illness which ones work? Meta-analysis of published reports.* Bmj, 2002. **325**(7370): p. 925.

For peer review only - http://bmjopen.bmj.com/site/about/guidelines.xhtml

List of Figures

Figure 1: PRISMA flow diagram of the article selection process

Figure 2: Risk of bias in included studies

Figure 3: Forest plot of the random effects meta-analysis of the effect of integrated versus standard care on SBP for people with cardiometabolic multimorbidity in Sub-Saharan Africa

For peer review only

For peer review only - http://bmjopen.bmj.com/site/about/guidelines.xhtml

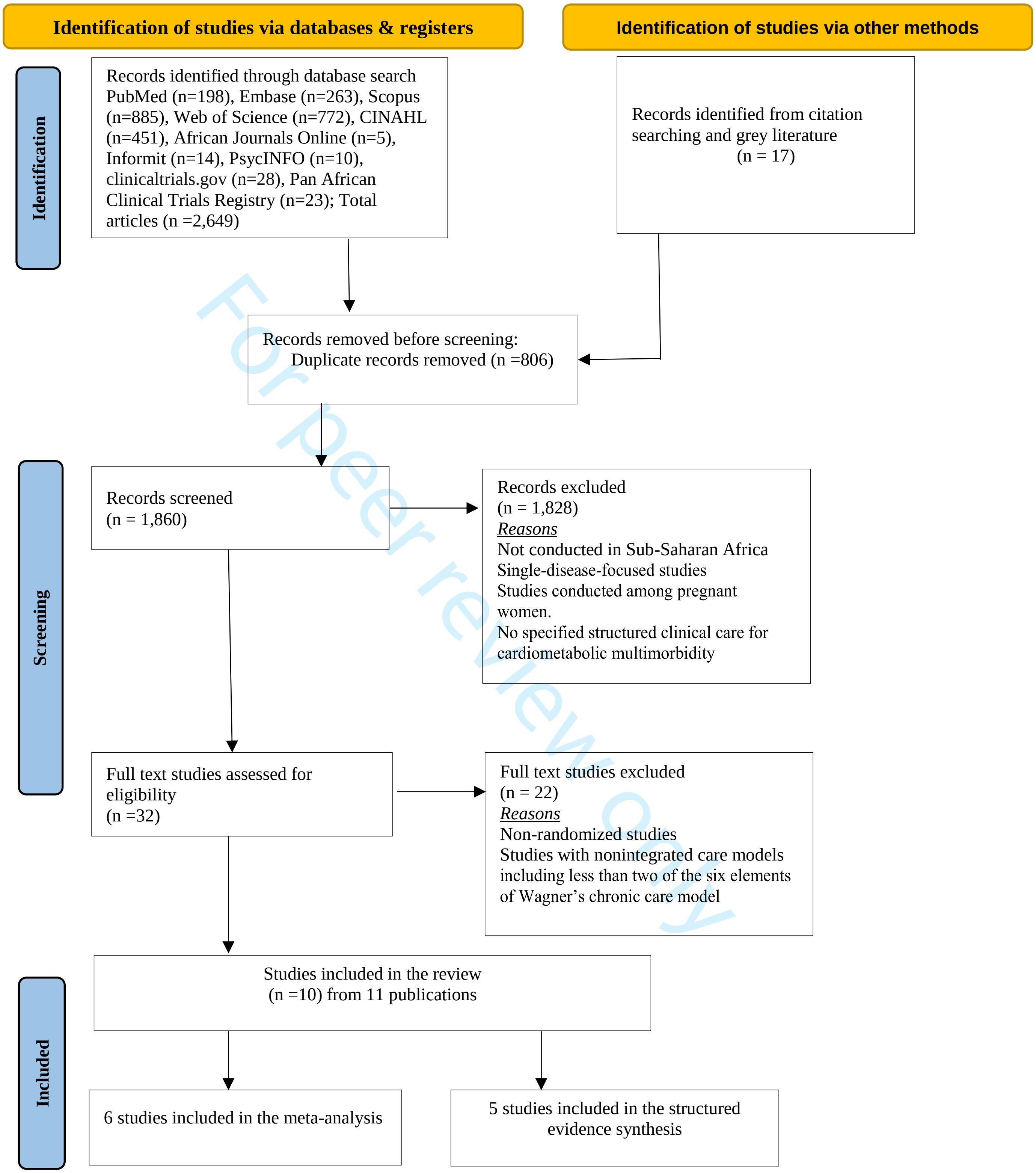

For peer review only

	Randomization process	Allocation concealment (selection bias)	Blinding (Performance and detection bias)	Selective reporting bias	Other bias	Protection against contamination	Reliable primary outcomes	Baseline measurements	Overall
Havlir et al (2019)	+	+	+	+	+	+	?	?	?
Jackson et al (2021)	+	+	+	+	+	?	+	+	+
Myers et al (2022)	+	+	+	+	+	+	+	+	+
Okube et al (2022)	+	+	+	+	+	?	+	+	+
Owolabi et al 2019	+	+	+	+	-	?	-	+	?
Petersen et al (2021)	+	+	+	+	+	+	+	+	+
Rabkin et al (2018)	+	?	+	+	-	?	+	+	?
Roos et al (2014)	+	+	+	+	+	?	-	+	?
Sarfo et al (2018)	+	+	+	?	-	+	-	+	?
Thuita et al (2020)	+	+	+	+	-	?	-	+	?

Low risk +
Some concerns ?
High risk -

ew only

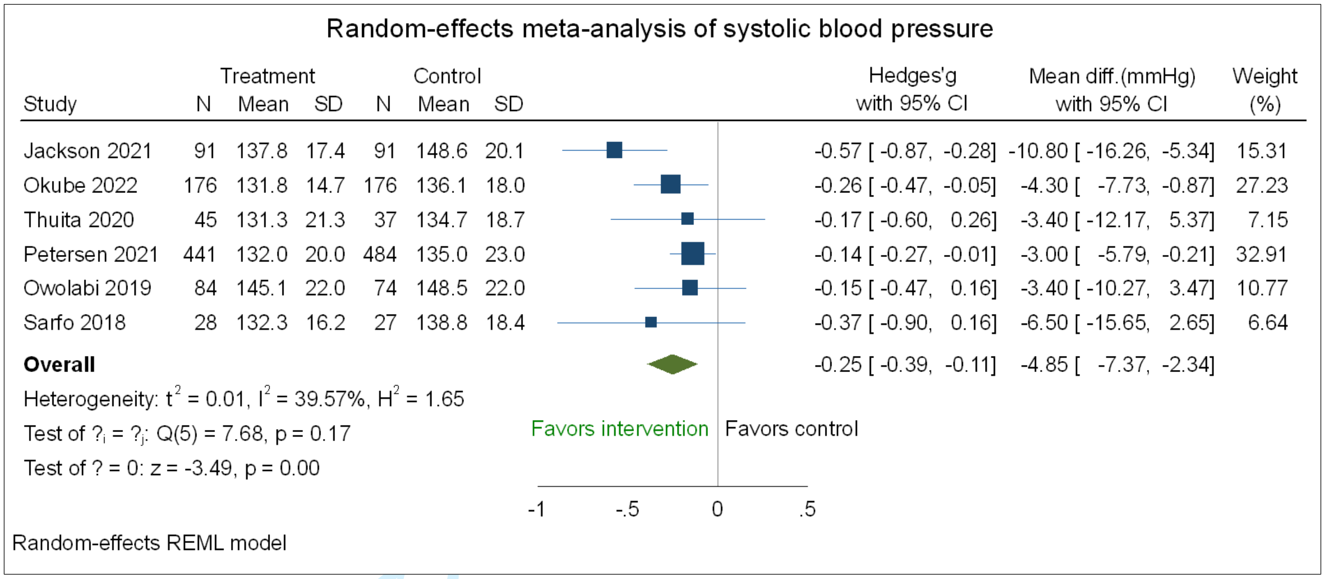

For peer review only

List of online supplementary files

Online supplementary file 1: Classification of interventions

Intervention component	Feature
Self-management support	• Self-care interventions based on technological aid<br>• Self-help groups<br>• Family-oriented supports<br>• Motivational support<br>• Behaviour therapy
Delivery system design	• Practice team functioning<br>• Patient care planning and follow-up<br>• Coordination between primary care and specialist services
Decision support	• Practice clinical guidelines<br>• Provider education<br>• Involvement of specialists in improving primary care
Clinical information system	• Disease registry<br>• Reminders to providers<br>• Feedback to providers
Healthcare organization	• Organizational goals and resources for chronic disease management<br>• Quality improvement strategies<br>• Incentives
Community linkages	• Linking patients to outside resources<br>• Activities with community-based organizations<br>• Professionals working out in the community

Supplementary file 2: Search strategies for electronic databases

1. **PubMED (Medline) Search strategy**

#1 ("elevated blood pressure"[MeSH Terms] OR "high blood pressure"[MeSH Terms] OR "raised blood pressure"[MeSH Terms] OR "diastolic blood pressure"[MeSH Terms] OR "systolic blood pressure"[MeSH Terms] OR "arterial blood pressure"[MeSH Terms] OR "uncontrolled blood pressure"[MeSH Terms] OR "uncontrolled hypertension"[All Fields] OR "elevated blood pressure"[All Fields] OR "high blood pressure"[All Fields] OR "raised blood pressure"[All Fields] OR "diastolic blood pressure"[All Fields] OR "systolic blood pressure"[All Fields] OR "arterial blood pressure"[All Fields] OR "uncontrolled blood pressure"[All Fields] OR "uncontrolled hypertension"[All Fields])

#2 ("diabetes mellitus"[MeSH Terms] OR "diabetes mellitus, type 2"[MeSH Terms] OR "diabetes type 2"[MeSH Terms] OR "T2DM"[MeSH Terms] OR "diabetes type II"[MeSH Terms] OR "diabetes"[MeSH Terms] OR "glucose intolerance"[MeSH Terms] OR "insulin resistance"[MeSH Terms] OR "hyperglycemia"[MeSH Terms] OR "hyperglycaemia"[MeSH Terms] OR "hypoglycaemia"[MeSH Terms] OR "hypoglycaemia"[MeSH Terms] OR "high blood sugar"[MeSH Terms] OR "elevated blood sugar"[MeSH Terms] OR "raised blood sugar"[MeSH Terms] OR "high blood glucose"[MeSH Terms] OR "elevated blood glucose"[MeSH Terms] OR "raised blood glucose"[MeSH Terms] OR "diabetes mellitus"[All Fields] OR "diabetes mellitus, type 2"[All Fields] OR "diabetes type 2"[All Fields] OR "T2DM"[All Fields] OR "diabetes type II"[All Fields] OR "diabetes"[All Fields] OR "glucose intolerance"[All Fields] OR "insulin resistance"[All Fields] OR "hyperglycemia"[All Fields] OR "hyperglycaemia"[All Fields] OR "hypoglycemia"[All Fields] OR "hypoglycaemia"[All Fields] OR "high blood sugar"[All Fields] OR "elevated blood sugar"[All Fields] OR "raised blood sugar"[All Fields] OR "high blood glucose"[All Fields] OR "elevated blood glucose"[All Fields] OR "raised blood glucose"[All Fields])

For peer review only - http://bmjopen.bmj.com/site/about/guidelines.xhtml

#3 ("stroke"[MeSH Terms] OR "transient ischemic attack"[MeSH Terms] OR "ischemic attack"[MeSH Terms] OR "angina"[MeSH Terms] OR "angina pectoris"[MeSH Terms] OR "heart attack"[MeSH Terms] OR "ischemic heart diseases "[MeSH Terms] OR "transient ischaemic attack"[MeSH Terms] OR "coronary heart disease"[MeSH Terms] OR "coronary disease"[MeSH Terms] OR "heart failure"[MeSH Terms] OR "peripheral vascular disease"[MeSH Terms] OR "Peripheral Vascular diseases"[MeSH Terms] OR "atrial fibrillation"[MeSH Terms] OR "cardiovascular disease"[MeSH Terms] OR "heart disease"[MeSH Terms] OR "stroke"[All Fields] OR "transient ischemic attack"[All Fields] OR "ischemic attack"[All Fields] OR "angina"[All Fields] OR "angina pectoris"[All Fields] OR "heart attack"[All Fields] OR "ischemic heart diseases "[All Fields] OR "transient ischaemic attack"[All Fields] OR "coronary heart disease"[All Fields] OR "coronary disease"[All Fields] OR "heart failure"[All Fields] OR "peripheral vascular disease"[All Fields] OR "Peripheral Vascular diseases"[All Fields] OR "atrial fibrillation"[All Fields] OR "cardiovascular disease"[All Fields] OR "heart disease"[All Fields])

#4 ("Hypercholesterolemia"[MeSH Terms] OR "Dyslipidemias"[MeSH Terms] OR "blood lipid"[MeSH Terms] OR "Cholesterol"[MeSH Terms] OR "high cholesterol"[MeSH Terms] OR "elevated cholesterol"[MeSH Terms] OR "raised cholesterol"[MeSH Terms] OR "low density lipoprotein "[MeSH Terms] OR "high density lipoprotein"[MeSH Terms] OR "Dyslipidemia"[MeSH Terms] OR "Dyslipidaemia"[MeSH Terms] OR "Hypercholesterolemia"[MeSH Terms] OR "hypercholesterolaemia "[MeSH Terms] OR "hypercholesterolimia "[MeSH Terms] OR "Hypertriglyceridemia"[MeSH Terms] OR "Hypertriglyceridemia"[MeSH Terms] OR "Hypertriglyceridaemia"[MeSH Terms] OR "Hyperlipidemia"[MeSH Terms] OR "Hyperlipidemias"[MeSH Terms] OR "Hyperlipidaemia"[MeSH Terms] OR "LDL"[MeSH Terms] OR "HDL"[MeSH Terms] OR "Hypercholesterolemia"[All Fields] OR "Dyslipidemias"[All Fields] OR "blood lipid"[All Fields] OR "Cholesterol"[All Fields] OR "high cholesterol"[All Fields] OR "elevated cholesterol"[All Fields] OR "raised cholesterol"[All Fields] OR "low density lipoprotein "[All Fields] OR "high density lipoprotein"[All Fields] OR "Dyslipidemia"[All Fields] OR "Dyslipidaemia"[All Fields] OR "Hypercholesterolemia"[All Fields] OR "hypercholesterolaemia "[All Fields] OR "hypercholesterolimia "[All Fields] OR "Hypertriglyceridemia"[All Fields] OR "Hypertriglyceridemia"[All Fields] OR "Hypertriglyceridaemia"[All Fields] OR "Hyperlipidemia"[All Fields] OR "Hyperlipidemias"[All Fields] OR "Hyperlipidaemia"[All Fields] OR "LDL"[All Fields] OR "HDL"[All Fields])

#5 ("Comorbid"[All Fields] OR "co-morbid"[All Fields] OR "co-morbidity"[All Fields] OR "Multimorbidity"[All Fields] OR "multi-morbid"[All Fields] OR "multi morbidity"[All Fields] OR "Multimorbidity"[All Fields] OR "Comorbidity"[All Fields] OR "multi-disease"[All Fields] OR "Multidisease"[All Fields] OR multi disease[All Fields] OR "multiple condition"[All Fields] OR "multi-condition"[All Fields] OR "multi condition"[All Fields] OR "multiple illness"[All Fields] OR "multi-illness"[All Fields] OR "multi illness"[All Fields] OR "multiple syndrome"[All Fields] OR "multi-syndrome"[All Fields] OR "multi syndrome"[All Fields] OR "concurrent condition"[All Fields] OR "concurrent illness"[All Fields] OR "concurrent disease"[All Fields] OR "co-existing disease"[All Fields] OR "coexisting disease"[All Fields] OR "co-existing illness"[All Fields] OR "coexisting illness"[All Fields] OR "co-existing syndrome"[All Fields] OR "coexisting syndrome"[All Fields] OR "co-existing condition"[All Fields] OR "coexisting condition"[All Fields] OR "co-occurring disease"[All Fields] OR "co-occuring disease"[All Fields] OR "co-occuring disease"[All Fields] OR "co-occurring illness"[All Fields] OR "co occurring illness"[All Fields] OR "cooccurring illness"[All Fields] OR "co-occurring syndrome"[All Fields] OR "co occurring syndrome"[All Fields] OR "cooccurring syndrome"[All Fields] OR "co-occurring condition"[All Fields] OR "co occurring condition"[All Fields] OR "cooccurring condition"[All Fields])

#6 ("Wagner model"[All Fields] OR "Wagner* model*"[All Fields] OR "Wagner* chronic care model*"[All Fields] OR "Wagner chronic care model"[All Fields] OR "chronic care model*"[All Fields] OR "model "[All Fields] OR "collaborative care"[All Fields] OR "chronic care framework"[All Fields] OR "chronic disease care"[All Fields] OR "chronic illness care"[All Fields] OR care model[All Fields] OR "model care"[All Fields] OR "Wagner* model*"[All Fields] OR "Wagner* chronic care model*"[All Fields] OR "Wagner chronic care model"[All Fields] OR "theory"[All Fields] OR "concept"[All Fields] OR "framework"[All Fields] OR "model"[All Fields] OR "programme"[All Fields] OR "approach"[All Fields] OR "clinical pathway"[All Fields] OR "care pathway"[All Fields] OR "critical path"[All Fields] OR "vertical integration"[All Fields] OR "virtual integration"[All Fields] OR "physician system integration"[All Fields] OR "provider system integration"[All Fields] OR "functional

For peer review only - http://bmjopen.bmj.com/site/about/guidelines.xhtml

integration"[All Fields] OR "horizontal integration"[All Fields] OR "clinical integration"[All Fields] OR "case management"[All Fields] OR "delivery of health care, integrated"[All Fields] OR "disease management"[All Fields] OR "patient care management"[All Fields] OR "patient-centred care"[All Fields] OR "accountable care organisations"[All Fields] OR "continuity of patient care"[All Fields] OR "case management"[All Fields] OR "comprehensive health care"[All Fields] OR "delivery of health care, integrated"[All Fields] OR "managed care programmes"[All Fields] OR "patient-centred care"[All Fields] OR "care delivery"[All Fields] OR "integrated care"[All Fields] OR "comprehensive care"[All Fields] OR "care coordination"[All Fields] OR "managed care"[All Fields] OR "accountable care "[All Fields] OR "accountable care organisations"[All Fields] OR "accountable care organisation"[All Fields] OR "collaborative care"[All Fields] OR "disease management"[All Fields] OR "case-management"[All Fields] OR "case management"[All Fields] OR "shared care"[All Fields] OR "accountable care"[All Fields] OR "patient-centred"[All Fields] OR "patient centred"[All Fields] OR "person-centred"[All Fields] OR "person centred"[All Fields] OR "multidisciplinary care"[All Fields] OR "interdisciplinary care"[All Fields] OR "inter-disciplinary care"[All Fields] OR "cross disciplinary care"[All Fields] OR "cross-disciplinary care"[All Fields] OR "multiple interventions"[All Fields] OR "care chain"[All Fields] OR "care chains"[All Fields] OR "care continuity"[All Fields] OR "care continuation"[All Fields] OR "care transition"[All Fields] OR "care transitions"[All Fields] OR "chain of care"[All Fields] OR "chains of care"[All Fields] OR "continuity of care"[All Fields] OR "cross sectoral care"[All Fields] OR "delivery of health care integrated"[All Fields] OR "integrated medicine"[All Fields] OR "integrated social network"[All Fields] OR "integrated social networks"[All Fields] OR "integration of care"[All Fields] OR "intersectoral care"[All Fields] OR "intrasectoral care"[All Fields] OR "linked care"[All Fields] OR "management model"[All Fields] OR "patient care management"[All Fields] OR "seamless care"[All Fields] OR "service network"[All Fields] OR "service networks"[All Fields] OR "transition of care"[All Fields] OR "transitional care"[All Fields] OR "transmural care"[All Fields] OR "whole system thinking"[All Fields] OR "holistic care"[All Fields])

#7 ("Angola "[All Fields] OR "Benin "[All Fields] OR "Botswana "[All Fields] OR "Burkina Faso"[All Fields] OR "Upper Volta"[All Fields] OR "Burundi "[All Fields] OR "Cameroon "[All Fields] OR "Cameroons "[All Fields] OR "Cape Verde"[All Fields] OR "Central African Republic"[All Fields] OR Chad[All Fields] OR "Comoros "[All Fields] OR "Comoro Islands"[All Fields] OR "Comores"[All Fields] OR "Mayotte "[All Fields] OR "Congo "[All Fields] OR "Zaire "[All Fields] OR "Cote d'Ivoire"[All Fields] OR "Ivory Coast"[All Fields] OR "Democratic Republic of the Congo"[All Fields] OR "Djibouti "[All Fields] OR "French Somaliland"[All Fields] OR "Eritrea "[All Fields] OR "Ethiopia "[All Fields] OR "Gabon "[All Fields] OR "Gabonese Republic"[All Fields] OR "Gambia "[All Fields] OR "Ghana "[All Fields] OR "Gold Coast"[All Fields] OR "Guinea "[All Fields] OR "Kenya "[All Fields] OR "Lesotho"[All Fields] OR "Basutoland"[All Fields] OR "Liberia "[All Fields] OR "Madagascar "[All Fields] OR "Malagasy Republic "[All Fields] OR "Malawi "[All Fields] OR "Nyasaland "[All Fields] OR "Mali "[All Fields] OR "Mauritania "[All Fields] OR "Mauritius "[All Fields] OR "Mozambique"[All Fields] OR "Namibia"[All Fields] OR "Niger"[All Fields] OR "Nigeria"[All Fields] OR "Rwanda"[All Fields] OR "Sao Tome"[All Fields] OR "Seychelles"[All Fields] OR "Senegal "[All Fields] OR "Sierra Leone"[All Fields] OR "Somalia "[All Fields] OR "South Africa"[All Fields] OR "Sudan "[All Fields] OR "Eswatini"[All Fields] OR "Tanzania"[All Fields] OR "Togo "[All Fields] OR "Togolese Republic"[All Fields] OR "Uganda"[All Fields] OR "Zambia"[All Fields] OR "Zimbabwe "[All Fields] OR "Rhodesia"[All Fields] OR "Sub-Saharan Africa"[All Fields] OR "Africa South of the Sahara"[All Fields])

#8 ((#1 OR #2 OR #3 OR #4 OR #5) AND #6 AND #7) AND Restricted (1999-2022, RCTs)

2. Web of Science Search Strategy

#1 elevated blood pressure OR high blood pressure OR raised blood pressure OR diastolic blood pressure OR systolic blood pressure OR arterial blood pressure OR uncontrolled blood pressure OR uncontrolled hypertension OR elevated blood pressure OR high blood pressure OR raised blood pressure OR diastolic blood pressure OR systolic blood pressure OR arterial blood pressure OR uncontrolled blood pressure OR uncontrolled hypertension

#2 diabetes mellitus OR diabetes mellitus, type 2 OR diabetes type 2 OR T2DM OR diabetes type II OR diabetes OR glucose intolerance OR insulin resistance OR hyperglycemia OR hyperglycaemia OR hypoglycaemia

For peer review only - http://bmjopen.bmj.com/site/about/guidelines.xhtml

OR hypoglycaemia OR high blood sugar OR elevated blood sugar OR raised blood sugar OR high blood glucose OR elevated blood glucose OR raised blood glucose OR diabetes mellitus OR diabetes mellitus, type 2 OR diabetes type 2 OR T2DM OR diabetes type II OR diabetes OR glucose intolerance OR insulin resistance OR hyperglycemia OR hyperglycaemia OR hypoglycemia OR hypoglycaemia OR high blood sugar OR elevated blood sugar OR raised blood sugar OR high blood glucose OR elevated blood glucose OR raised blood glucose

#3 stroke OR transient ischemic attack OR ischemic attack OR angina OR angina pectoris OR heart attack OR ischemic heart diseases OR transient ischaemic attack OR coronary heart disease OR coronary disease OR heart failure OR peripheral vascular disease OR Peripheral Vascular diseases OR atrial fibrillation OR cardiovascular disease OR heart disease OR stroke OR transient ischemic attack OR ischemic attack OR angina OR angina pectoris OR heart attack OR ischemic heart diseases OR transient ischaemic attack OR coronary heart disease OR coronary disease OR heart failure OR peripheral vascular disease OR Peripheral Vascular diseases OR atrial fibrillation OR cardiovascular disease OR heart disease

#4 Hypercholesterolemia OR Dyslipidemias OR blood lipid OR Cholesterol OR high cholesterol OR elevated cholesterol OR raised cholesterol OR low density lipoprotein OR high density lipoprotein OR Dyslipidemia OR Dyslipidaemia OR Hypercholesterolemia OR hypercholesterolaemia OR hypercholesterolimia OR Hypertriglyceridemia OR Hypertriglyceridemia OR Hypertriglyceridaemia OR Hyperlipidemia OR Hyperlipidemias OR Hyperlipidaemia OR LDL OR HDL OR Hypercholesterolemia OR Dyslipidemias OR blood lipid OR Cholesterol OR high cholesterol OR elevated cholesterol OR raised cholesterol OR low density lipoprotein OR high density lipoprotein OR Dyslipidemia OR Dyslipidaemia OR Hypercholesterolemia OR hypercholesterolaemia OR hypercholesterolimia OR Hypertriglyceridemia OR Hypertriglyceridemia OR Hypertriglyceridaemia OR Hyperlipidemia OR Hyperlipidemias OR Hyperlipidaemia OR LDL OR HDL

#5 Comorbid OR co-morbid OR co-morbidity OR Multimorbidity OR multi-morbid OR multi morbidity OR Multimorbidity OR Comorbidity OR multi-disease OR Multidisease OR multi disease OR multiple condition OR multi-condition OR multi condition OR multiple illness OR multi-illness OR multi illness OR multiple syndrome OR multi-syndrome OR multi syndrome OR concurrent condition OR concurrent illness OR concurrent disease OR co-existing disease OR coexisting disease OR co-existing illness OR coexisting illness OR co-existing syndrome OR coexisting syndrome OR co-existing condition OR coexisting condition OR co-occurring disease OR co-occuring disease OR co-occuring disease OR co-occurring illness OR co occurring illness OR cooccurring illness OR co-occurring syndrome OR co occurring syndrome OR cooccurring syndrome OR co-occurring condition OR co occurring condition OR cooccurring condition

#6 Wagner model OR Wagner* model* OR Wagner* chronic care model* OR Wagner chronic care model OR chronic care model* OR model OR collaborative care OR chronic care framework OR chronic disease care OR chronic illness care OR care model OR model care OR Wagner* model* OR Wagner* chronic care model* OR Wagner chronic care model OR theory OR concept OR framework OR model OR programme OR approach OR clinical pathway OR care pathway OR critical path OR vertical integration OR virtual integration OR physician system integration OR provider system integration OR functional integration OR horizontal integration OR clinical integration OR case management OR delivery of health care, integrated OR disease management OR patient care management OR patient-centred care OR accountable care organisations OR continuity of patient care OR case management OR comprehensive health care OR delivery of health care, integrated OR managed care programmes OR patient-centred care OR care delivery OR integrated care OR comprehensive care OR care coordination OR managed care OR accountable care OR accountable care organisations OR accountable care organisation OR collaborative care OR disease management OR case-management OR case management OR shared care OR accountable care OR patient-centred OR patient centred OR person-centred OR person centred OR multidisciplinary care OR interdisciplinary care OR inter-disciplinary care OR cross disciplinary care OR cross-disciplinary care OR multiple interventions OR care chain OR care chains OR care continuity OR care continuation OR care transition OR care transitions OR chain of care OR chains of care OR continuity of care OR cross sectoral care OR delivery of health care integrated OR integrated medicine OR integrated social network OR integrated social networks OR integration of care OR intersectoral care OR intrasectoral care OR linked care OR management model OR patient

For peer review only - http://bmjopen.bmj.com/site/about/guidelines.xhtml

care management OR seamless care OR service network OR service networks OR transition of care OR transitional care OR transmural care OR whole system thinking OR holistic care

#7 Angola OR Benin OR Botswana OR Burkina Faso OR Upper Volta OR Burundi OR Cameroon OR Cameroons OR Cape Verde OR Central African Republic OR Chad OR Comoros OR Comoro Islands OR Comores OR Mayotte OR Congo OR Zaire OR Cote d'Ivoire OR Ivory Coast OR Democratic Republic of the Congo OR Djibouti OR French Somaliland OR Eritrea OR Ethiopia OR Gabon OR Gabonese Republic OR Gambia OR Ghana OR Gold Coast OR Guinea OR Kenya OR Lesotho OR Basutoland OR Liberia OR Madagascar OR Malagasy Republic OR Malawi OR Nyasaland OR Mali OR Mauritania OR Mauritius OR Mozambique OR Namibia OR Niger OR Nigeria OR Rwanda OR Sao Tome OR Seychelles OR Senegal OR Sierra Leone OR Somalia OR South Africa OR Sudan OR Eswatini OR Tanzania OR Togo OR Togolese Republic OR Uganda OR Zambia OR Zimbabwe OR Rhodesia OR Sub-Saharan Africa OR Africa South of the Sahara

#8 trial* OR RCT

#9 ((#1 OR #2 OR #3 OR #4 OR #5) AND #6 AND #7 AND #8) and Clinical Trial (Document Types) and 1999 or 2000 or 2001 or 2002 or 2003 or 2004 or 2005 or 2006 or 2007 or 2008 or 2009 or 2011 or 2010 or 2012 or 2013 or 2014 or 2015 or 2016 or 2017 or 2018 or 2019 or 2020 or 2021 or 2022 (Publication Years)

3. SCOPUS

(TITLE-ABS-KEY (trial* OR rct)) AND (((TITLE-ABS-KEY ("wagner model" OR "wagner* model*" OR "wagner* chronic care model*" OR "wagner chronic care model" OR "chronic care model*" OR "model " OR "collaborative care" OR "chronic care framework" OR "chronic disease care" OR "chronic illness care" OR care AND model OR "model care" OR "wagner* model*" OR "wagner* chronic care model*" OR "wagner chronic care model" OR "theory" OR "concept" OR "framework" OR "model" OR "programme" OR "approach" OR "clinical pathway" OR "care pathway" OR "critical path" OR "vertical integration" OR "virtual integration" OR "physician system integration" OR "provider system integration" OR "functional integration" OR "horizontal integration" OR "clinical integration" OR "case management" OR "delivery of health care, integrated" OR "disease management" OR "patient care management" OR "patient-centred care")) OR (TITLE-ABS-KEY ("accountable care organisations" OR "continuity of patient care" OR "case management" OR "comprehensive health care" OR "delivery of health care, integrated" OR "managed care programmes" OR "patient-centred care" OR "care delivery" OR "integrated care" OR "comprehensive care" OR "care coordination" OR "managed care" OR "accountable care " OR "accountable care organisations" OR "accountable care organisation" OR "collaborative care" OR "disease management" OR "case-management" OR "case management" OR "shared care" OR "accountable care" OR "patient-centred" OR "patient centred" OR "person-centred" OR "person centred" OR "multidisciplinary care" OR "interdisciplinary care" OR "inter-disciplinary care" OR "cross disciplinary care" OR "cross-disciplinary care" OR "multiple interventions" OR "care chain" OR "care chains" OR "care continuity" OR "care continuation" OR "care transition")) OR (TITLE-ABS-KEY ("care transitions" OR "chain of care" OR "chains of care" OR "continuity of care" OR "cross sectoral care" OR "delivery of health care integrated" OR "integrated medicine" OR "integrated social network" OR "integrated social networks" OR "integration of care" OR "intersectoral care" OR "intrasectoral care" OR "linked care" OR "management model" OR "patient care management" OR "seamless care" OR "service network" OR "service networks" OR "transition of care" OR "transitional care" OR "transmural care" OR "whole system thinking" OR "holistic care"))) AND ((TITLE-ABS-KEY (("elevated blood pressure" OR "high blood pressure" OR "raised blood pressure" OR "diastolic blood pressure" OR "systolic blood pressure" OR "arterial blood pressure" OR "uncontrolled blood pressure" OR "uncontrolled hypertension" OR "elevated blood pressure" OR "high blood pressure" OR "raised blood pressure" OR "diastolic blood pressure" OR "systolic blood pressure" OR "arterial blood pressure" OR "uncontrolled blood pressure" OR "uncontrolled hypertension"))) OR (TITLE-ABS-KEY (("diabetes mellitus" OR "diabetes mellitus, type 2" OR "diabetes type 2" OR "t2dm" OR "diabetes type ii" OR "diabetes" OR "glucose intolerance" OR "insulin resistance" OR "hyperglycemia" OR "hyperglycaemia" OR "hypoglycaemia" OR "hypoglycaemia" OR "high blood sugar" OR "elevated blood sugar" OR "raised blood sugar" OR "high blood glucose" OR "elevated blood

For peer review only - http://bmjopen.bmj.com/site/about/guidelines.xhtml

glucose" OR "raised blood glucose" OR "diabetes mellitus" OR "diabetes mellitus, type 2" OR "diabetes type 2" OR "t2dm" OR "diabetes type ii" OR "diabetes" OR "glucose intolerance" OR "insulin resistance" OR "hyperglycemia" OR "hyperglycaemia" OR "hypoglycemia" OR "hypoglycaemia" OR "high blood sugar" OR "elevated blood sugar" OR "raised blood sugar" OR "high blood glucose" OR "elevated blood glucose" OR "raised blood glucose"))) OR (TITLE-ABS-KEY (("stroke" OR "transient ischemic attack" OR "ischemic attack" OR "angina" OR "angina pectoris" OR "heart attack" OR "ischemic heart diseases " OR "transient ischaemic attack" OR "coronary heart disease" OR "coronary disease" OR "heart failure" OR "peripheral vascular disease" OR "peripheral vascular diseases" OR "atrial fibrillation" OR "cardiovascular disease" OR "heart disease" OR "stroke" OR "transient ischemic attack" OR "ischemic attack" OR "angina" OR "angina pectoris" OR "heart attack" OR "ischemic heart diseases " OR "transient ischaemic attack" OR "coronary heart disease" OR "coronary disease" OR "heart failure" OR "peripheral vascular disease" OR "peripheral vascular diseases" OR "atrial fibrillation" OR "cardiovascular disease" OR "heart disease"))) OR (TITLE-ABS-KEY (("hypercholesterolemia" OR "dyslipidemias" OR "blood lipid" OR "cholesterol" OR "high cholesterol" OR "elevated cholesterol" OR "raised cholesterol" OR "low density lipoprotein " OR "high density lipoprotein" OR "dyslipidemia" OR "dyslipidaemia" OR "hypercholesterolemia" OR "hypercholesterolaemia " OR "hypercholesterolimia " OR "hypertriglyceridemia" OR "hypertriglyceridemia" OR "hypertriglyceridaemia" OR "hyperlipidemia" OR "hyperlipidemias" OR "hyperlipidaemia" OR "ldl" OR "hdl" OR "hypercholesterolemia" OR "dyslipidemias" OR "blood lipid" OR "cholesterol" OR "high cholesterol" OR "elevated cholesterol" OR "raised cholesterol" OR "low density lipoprotein " OR "high density lipoprotein" OR "dyslipidemia" OR "dyslipidaemia" OR "hypercholesterolemia" OR "hypercholesterolaemia " OR "hypercholesterolimia " OR "hypertriglyceridemia" OR "hypertriglyceridemia" OR "hypertriglyceridaemia" OR "hyperlipidemia" OR "hyperlipidemias" OR "hyperlipidaemia" OR "ldl" OR "hdl"))) OR (TITLE-ABS-KEY (("comorbid" OR "co-morbid" OR "co-morbidity" OR "multimorbidity" OR "multi-morbid" OR "multi morbidity" OR "multimorbidity" OR "comorbidity" OR "multi-disease" OR "multidisease" OR multi AND disease OR "multiple condition" OR "multi-condition" OR "multi condition" OR "multiple illness" OR "multi-illness" OR "multi illness" OR "multiple syndrome" OR "multi-syndrome" OR "multi syndrome" OR "concurrent condition" OR "concurrent illness" OR "concurrent disease" OR "co-existing disease" OR "coexisting disease" OR "co-existing illness" OR "coexisting illness" OR "co-existing syndrome" OR "coexisting syndrome" OR "co-existing condition" OR "coexisting condition" OR "co-occurring disease" OR "co-occuring disease" OR "co-occuring disease" OR "co-occurring illness" OR "co occurring illness" OR "cooccurring illness" OR "co-occurring syndrome" OR "co occurring syndrome" OR "cooccurring syndrome" OR "co-occurring condition" OR "co occurring condition" OR "cooccurring condition"))))) AND (TITLE-ABS-KEY (("angola " OR "benin " OR "botswana " OR "burkina faso" OR "upper volta" OR "burundi " OR "cameroon " OR "cameroons " OR "cape verde" OR "central african republic" OR chad OR "comoros " OR "comoro islands" OR "comores" OR "mayotte " OR "congo " OR "zaire " OR "cote d'ivoire" OR "ivory coast" OR "democratic republic of the congo" OR "djibouti " OR "french somaliland" OR "eritrea " OR "ethiopia " OR "gabon " OR "gabonese republic" OR "gambia " OR "ghana " OR "gold coast" OR "guinea " OR "kenya " OR "lesotho" OR "basutoland" OR "liberia " OR "madagascar " OR "malagasy republic " OR "malawi " OR "nyasaland " OR "mali " OR "mauritania " OR "mauritius " OR "mozambique" OR "namibia" OR "niger" OR "nigeria" OR "rwanda" OR "sao tome" OR "seychelles" OR "senegal " OR "sierra leone" OR "somalia " OR "south africa" OR "sudan " OR "Eswatini" OR "tanzania" OR "togo " OR "Togolese republic" OR "uganda" OR "zambia" OR "zimbabwe " OR "rhodesia" OR "sub-saharan africa" OR "africa south of the sahara"))))) AND (LIMIT-TO (PUBYEAR , 2022) OR LIMIT-TO (PUBYEAR , 2021) OR LIMIT-TO (PUBYEAR , 2020) OR LIMIT-TO (PUBYEAR , 2019) OR LIMIT-TO (PUBYEAR , 2018) OR LIMIT-TO (PUBYEAR , 2017) OR LIMIT-TO (PUBYEAR , 2016) OR LIMIT-TO (PUBYEAR , 2015) OR LIMIT-TO (PUBYEAR , 2014) OR LIMIT-TO (PUBYEAR , 2013) OR LIMIT-TO (PUBYEAR , 2012) OR LIMIT-TO (PUBYEAR , 2011) OR LIMIT-TO (PUBYEAR , 2010) OR LIMIT-TO (PUBYEAR , 2009) OR LIMIT-TO (PUBYEAR , 2008) OR LIMIT-TO (PUBYEAR , 2007) OR LIMIT-TO (PUBYEAR , 2006) OR LIMIT-TO (PUBYEAR , 2005) OR LIMIT-

For peer review only - http://bmjopen.bmj.com/site/about/guidelines.xhtml

TO (PUBYEAR , 2004) OR LIMIT-TO (PUBYEAR , 2003) OR LIMIT-TO (PUBYEAR , 2002) OR LIMIT-TO (PUBYEAR , 2001) OR LIMIT-TO (PUBYEAR , 2000) OR LIMIT-TO (PUBYEAR , 1999))

4. **Embase**

#1 ("elevated blood pressure"/ OR "high blood pressure"/ OR "raised blood pressure"/ OR "diastolic blood pressure"/ OR "systolic blood pressure"/ OR "arterial blood pressure"/ OR "uncontrolled blood pressure"/ OR "uncontrolled hypertension" OR "elevated blood pressure" OR "high blood pressure" OR "raised blood pressure" OR "diastolic blood pressure" OR "systolic blood pressure" OR "arterial blood pressure" OR "uncontrolled blood pressure" OR "uncontrolled hypertension")

#2 ("diabetes mellitus"/ OR "diabetes mellitus, type 2"/ OR "diabetes type 2"/ OR "T2DM"/ OR "diabetes type II"/ OR "diabetes"/ OR "glucose intolerance"/ OR "insulin resistance"/ OR "hyperglycemia"/ OR "hyperglycaemia"/ OR "hypoglycaemia"/ OR "hypoglycaemia"/ OR "high blood sugar"/ OR "elevated blood sugar"/ OR "raised blood sugar"/ OR "high blood glucose"/ OR "elevated blood glucose"/ OR "raised blood glucose"/ OR "diabetes mellitus" OR "diabetes mellitus, type 2" OR "diabetes type 2" OR "T2DM" OR "diabetes type II" OR "diabetes" OR "glucose intolerance" OR "insulin resistance" OR "hyperglycemia" OR "hyperglycaemia" OR "hypoglycemia" OR "hypoglycaemia" OR "high blood sugar" OR "elevated blood sugar" OR "raised blood sugar" OR "high blood glucose" OR "elevated blood glucose" OR "raised blood glucose")

#3 ("stroke"/ OR "transient ischemic attack"/ OR "ischemic attack"/ OR "angina"/ OR "angina pectoris"/ OR "heart attack"/ OR "ischemic heart diseases "/ OR "transient ischaemic attack"/ OR "coronary heart disease"/ OR "coronary disease"/ OR "heart failure"/ OR "peripheral vascular disease"/ OR "Peripheral Vascular diseases"/ OR "atrial fibrillation"/ OR "cardiovascular disease"/ OR "heart disease"/ OR "stroke" OR "transient ischemic attack" OR "ischemic attack" OR "angina" OR "angina pectoris" OR "heart attack" OR "ischemic heart diseases " OR "transient ischaemic attack" OR "coronary heart disease" OR "coronary disease" OR "heart failure" OR "peripheral vascular disease" OR "Peripheral Vascular diseases" OR "atrial fibrillation" OR "cardiovascular disease" OR "heart disease")

#4 ("Hypercholesterolemia"/ OR "Dyslipidemias"/ OR "blood lipid"/ OR "Cholesterol"/ OR "high cholesterol"/ OR "elevated cholesterol"/ OR "raised cholesterol"/ OR "low density lipoprotein "/ OR "high density lipoprotein"/ OR "Dyslipidemia"/ OR "Dyslipidaemia"/ OR "Hypercholesterolemia"/ OR "hypercholesterolaemia "/ OR "hypercholesterolimia "/ OR "Hypertriglyceridemia"/ OR "Hypertriglyceridemia"/ OR "Hypertriglyceridaemia"/ OR "Hyperlipidemia"/ OR "Hyperlipidemias"/ OR "Hyperlipidaemia"/ OR "LDL"/ OR "HDL"/ OR "Hypercholesterolemia" OR "Dyslipidemias" OR "blood lipid" OR "Cholesterol" OR "high cholesterol" OR "elevated cholesterol" OR "raised cholesterol" OR "low density lipoprotein " OR "high density lipoprotein" OR "Dyslipidemia" OR "Dyslipidaemia" OR "Hypercholesterolemia" OR "hypercholesterolaemia " OR "hypercholesterolimia " OR "Hypertriglyceridemia" OR "Hypertriglyceridemia" OR "Hypertriglyceridaemia" OR "Hyperlipidemia" OR "Hyperlipidemias" OR "Hyperlipidaemia" OR "LDL" OR "HDL")

#5 ("Comorbid" OR "co-morbid" OR "co-morbidity" OR "Multimorbidity" OR "multi-morbid" OR "multi morbidity" OR "Multimorbidity" OR "Comorbidity" OR "multi-disease" OR "Multidisease" OR multi disease OR "multiple condition" OR "multi-condition" OR "multi condition" OR "multiple illness" OR "multi-illness" OR "multi illness" OR "multiple syndrome" OR "multi-syndrome" OR "multi syndrome" OR "concurrent condition" OR "concurrent illness" OR "concurrent disease" OR "co-existing disease" OR "coexisting disease" OR "co-existing illness" OR "coexisting illness" OR "co-existing syndrome" OR "coexisting syndrome" OR "co-existing condition" OR "coexisting condition" OR "co-occurring disease" OR "co-occuring disease" OR "co-occuring disease" OR "co-occurring illness" OR "co occurring illness" OR "cooccurring illness" OR "co-occurring syndrome" OR "co occurring syndrome" OR "cooccurring syndrome" OR "co-occurring condition" OR "co occurring condition" OR "cooccurring condition")

#6 ("Wagner model" OR "Wagner* model*" OR "Wagner* chronic care model*" OR "Wagner chronic care model" OR "chronic care model*" OR "model " OR "collaborative care" OR "chronic care framework" OR "chronic disease care" OR "chronic illness care" OR care model OR "model care" OR "Wagner* model*" OR "Wagner* chronic care model*" OR "Wagner chronic care model" OR "theory" OR "concept" OR "framework" OR "model"

For peer review only - http://bmjopen.bmj.com/site/about/guidelines.xhtml

OR "programme" OR "approach" OR "clinical pathway" OR "care pathway" OR "critical path" OR "vertical integration" OR "virtual integration" OR "physician system integration" OR "provider system integration" OR "functional integration" OR "horizontal integration" OR "clinical integration" OR "case management" OR "delivery of health care, integrated" OR "disease management" OR "patient care management" OR "patient-centred care" OR "accountable care organisations" OR "continuity of patient care" OR "case management" OR "comprehensive health care" OR "delivery of health care, integrated" OR "managed care programmes" OR "patient-centred care" OR "care delivery" OR "integrated care" OR "comprehensive care" OR "care coordination" OR "managed care" OR "accountable care " OR "accountable care organisations" OR "accountable care organisation" OR "collaborative care" OR "disease management" OR "case-management" OR "case management" OR "shared care" OR "accountable care" OR "patient-centred" OR "patient centred" OR "person-centred" OR "person centred" OR "multidisciplinary care" OR "interdisciplinary care" OR "inter-disciplinary care" OR "cross disciplinary care" OR "cross-disciplinary care" OR "multiple interventions" OR "care chain" OR "care chains" OR "care continuity" OR "care continuation" OR "care transition" OR "care transitions" OR "chain of care" OR "chains of care" OR "continuity of care" OR "cross sectoral care" OR "delivery of health care integrated" OR "integrated medicine" OR "integrated social network" OR "integrated social networks" OR "integration of care" OR "intersectoral care" OR "intrasectoral care" OR "linked care" OR "management model" OR "patient care management" OR "seamless care" OR "service network" OR "service networks" OR "transition of care" OR "transitional care" OR "transmural care" OR "whole system thinking" OR "holistic care")

#7 ("Angola " OR "Benin " OR "Botswana " OR "Burkina Faso" OR "Upper Volta" OR "Burundi " OR "Cameroon " OR "Cameroons " OR "Cape Verde" OR "Central African Republic" OR Chad OR "Comoros " OR "Comoro Islands" OR "Comores" OR "Mayotte " OR "Congo " OR "Zaire " OR "Cote d'Ivoire" OR "Ivory Coast" OR "Democratic Republic of the Congo" OR "Djibouti " OR "French Somaliland" OR "Eritrea " OR "Ethiopia " OR "Gabon " OR "Gabonese Republic" OR "Gambia " OR "Ghana " OR "Gold Coast" OR "Guinea " OR "Kenya " OR "Lesotho" OR "Basutoland" OR "Liberia " OR "Madagascar " OR "Malagasy Republic " OR "Malawi " OR "Nyasaland " OR "Mali " OR "Mauritania " OR "Mauritius " OR "Mozambique" OR "Namibia" OR "Niger" OR "Nigeria" OR "Rwanda" OR "Sao Tome" OR "Seychelles" OR "Senegal " OR "Sierra Leone" OR "Somalia " OR "South Africa" OR "Sudan " OR "Eswatini" OR "Tanzania" OR "Togo " OR "Togolese Republic" OR "Uganda" OR "Zambia" OR "Zimbabwe " OR "Rhodesia" OR "Sub-Saharan Africa" OR "Africa South of the Sahara")

#8 ((#1 OR #2 OR #3 OR #4 OR #5) AND #6 AND #7) & 1999 - 2022 & RCTs

5. **Cochrane Library**

#1 (("elevated blood pressure"(MeSH Terms) OR "high blood pressure"(MeSH Terms) OR "raised blood pressure"(MeSH Terms) OR "diastolic blood pressure"(MeSH Terms) OR "systolic blood pressure"(MeSH Terms) OR "arterial blood pressure"(MeSH Terms) OR "uncontrolled blood pressure"(MeSH Terms) OR "uncontrolled hypertension" OR "elevated blood pressure" OR "high blood pressure" OR "raised blood pressure" OR "diastolic blood pressure" OR "systolic blood pressure" OR "arterial blood pressure" OR "uncontrolled blood pressure" OR "uncontrolled hypertension")):ti,ab,kw (Word variations have been searched)

#2 (("diabetes mellitus"(MeSH Terms) OR "diabetes mellitus, type 2"(MeSH Terms) OR "diabetes type 2"(MeSH Terms) OR "T2DM"(MeSH Terms) OR "diabetes type II"(MeSH Terms) OR "diabetes"(MeSH Terms) OR "glucose intolerance"(MeSH Terms) OR "insulin resistance"(MeSH Terms) OR "hyperglycemia"(MeSH Terms) OR "hyperglycaemia"(MeSH Terms) OR "hypoglycaemia"(MeSH Terms) OR "hypoglycaemia"(MeSH Terms) OR "high blood sugar"(MeSH Terms) OR "elevated blood sugar"(MeSH Terms) OR "raised blood sugar"(MeSH Terms) OR "high blood glucose"(MeSH Terms) OR "elevated blood glucose"(MeSH Terms) OR "raised blood glucose"(MeSH Terms) OR "diabetes mellitus" OR "diabetes mellitus, type 2" OR "diabetes type 2" OR "T2DM" OR "diabetes type II" OR "diabetes" OR "glucose intolerance" OR "insulin resistance" OR "hyperglycemia" OR "hyperglycaemia" OR "hypoglycemia" OR "hypoglycaemia" OR "high blood sugar" OR "elevated blood sugar" OR "raised blood sugar" OR "high blood glucose" OR "elevated blood glucose" OR "raised blood glucose")):ti,ab,kw (Word variations have been searched)

For peer review only - http://bmjopen.bmj.com/site/about/guidelines.xhtml

#3 (("stroke"(MeSH Terms) OR "transient ischemic attack"(MeSH Terms) OR "ischemic attack"(MeSH Terms) OR "angina"(MeSH Terms) OR "angina pectoris"(MeSH Terms) OR "heart attack"(MeSH Terms) OR "ischemic heart diseases "(MeSH Terms) OR "transient ischaemic attack"(MeSH Terms) OR "coronary heart disease"(MeSH Terms) OR "coronary disease"(MeSH Terms) OR "heart failure"(MeSH Terms) OR "peripheral vascular disease"(MeSH Terms) OR "Peripheral Vascular diseases"(MeSH Terms) OR "atrial fibrillation"(MeSH Terms) OR "cardiovascular disease"(MeSH Terms) OR "heart disease"(MeSH Terms) OR "stroke" OR "transient ischemic attack" OR "ischemic attack" OR "angina" OR "angina pectoris" OR "heart attack" OR "ischemic heart diseases " OR "transient ischaemic attack" OR "coronary heart disease" OR "coronary disease" OR "heart failure" OR "peripheral vascular disease" OR "Peripheral Vascular diseases" OR "atrial fibrillation" OR "cardiovascular disease" OR "heart disease")):ti,ab,kw (Word variations have been searched)

#4 (("Hypercholesterolemia"(MeSH Terms) OR "Dyslipidemias"(MeSH Terms) OR "blood lipid"(MeSH Terms) OR "Cholesterol"(MeSH Terms) OR "high cholesterol"(MeSH Terms) OR "elevated cholesterol"(MeSH Terms) OR "raised cholesterol"(MeSH Terms) OR "low density lipoprotein "(MeSH Terms) OR "high density lipoprotein"(MeSH Terms) OR "Dyslipidemia"(MeSH Terms) OR "Dyslipidaemia"(MeSH Terms) OR "Hypercholesterolemia"(MeSH Terms) OR "hypercholesterolaemia "(MeSH Terms) OR "hypercholesterolimia "(MeSH Terms) OR "Hypertriglyceridemia"(MeSH Terms) OR "Hypertriglyceridemia"(MeSH Terms) OR "Hypertriglyceridaemia"(MeSH Terms) OR "Hyperlipidemia"(MeSH Terms) OR "Hyperlipidemias"(MeSH Terms) OR "Hyperlipidaemia"(MeSH Terms) OR "LDL"(MeSH Terms) OR "HDL"(MeSH Terms) OR "Hypercholesterolemia" OR "Dyslipidemias" OR "blood lipid" OR "Cholesterol" OR "high cholesterol" OR "elevated cholesterol" OR "raised cholesterol" OR "low density lipoprotein " OR "high density lipoprotein" OR "Dyslipidemia" OR "Dyslipidaemia" OR "Hypercholesterolemia" OR "hypercholesterolaemia " OR "hypercholesterolimia " OR "Hypertriglyceridemia" OR "Hypertriglyceridemia" OR "Hypertriglyceridaemia" OR "Hyperlipidemia" OR "Hyperlipidemias" OR "Hyperlipidaemia" OR "LDL" OR "HDL")):ti,ab,kw (Word variations have been searched)

#5 (("Comorbid" OR "co-morbid" OR "co-morbidity" OR "Multimorbidity" OR "multi-morbid" OR "multi morbidity" OR "Multimorbidity" OR "Comorbidity" OR "multi-disease" OR "Multidisease" OR multi disease OR "multiple condition" OR "multi-condition" OR "multi condition" OR "multiple illness" OR "multi-illness" OR "multi illness" OR "multiple syndrome" OR "multi-syndrome" OR "multi syndrome" OR "concurrent condition" OR "concurrent illness" OR "concurrent disease" OR "co-existing disease" OR "coexisting disease" OR "co-existing illness" OR "coexisting illness" OR "co-existing syndrome" OR "coexisting syndrome" OR "co-existing condition" OR "coexisting condition" OR "co-occurring disease" OR "co-occuring disease" OR "co-occuring disease" OR "co-occurring illness" OR "co occurring illness" OR "cooccurring illness" OR "co-occurring syndrome" OR "co occurring syndrome" OR "cooccurring syndrome" OR "co-occurring condition" OR "co occurring condition" OR "cooccurring condition")):ti,ab,kw (Word variations have been searched)

#6 (("Wagner model" OR "Wagner* model*" OR "Wagner* chronic care model*" OR "Wagner chronic care model" OR "chronic care model*" OR "model " OR "collaborative care" OR "chronic care framework" OR "chronic disease care" OR "chronic illness care" OR care model OR "model care" OR "Wagner* model*" OR "Wagner* chronic care model*" OR "Wagner chronic care model" OR "theory" OR "concept" OR "framework" OR "model" OR "programme" OR "approach" OR "clinical pathway" OR "care pathway" OR "critical path" OR "vertical integration" OR "virtual integration" OR "physician system integration" OR "provider system integration" OR "functional integration" OR "horizontal integration" OR "clinical integration" OR "case management" OR "delivery of health care, integrated" OR "disease management" OR "patient care management" OR "patient-centred care" OR "accountable care organisations" OR "continuity of patient care" OR "case management" OR "comprehensive health care" OR "delivery of health care, integrated" OR "managed care programmes" OR "patient-centred care" OR "care delivery" OR "integrated care" OR "comprehensive care" OR "care coordination" OR "managed care" OR "accountable care " OR "accountable care organisations" OR "accountable care organisation" OR "collaborative care" OR "disease management" OR "case-management" OR "case management" OR "shared care" OR "accountable care" OR "patient-centred" OR "patient centred" OR "person-centred" OR "person centred" OR "multidisciplinary care" OR "interdisciplinary care" OR "inter-disciplinary care" OR "cross disciplinary care" OR "cross-disciplinary care" OR "multiple interventions" OR "care chain" OR "care chains" OR "care continuity" OR

For peer review only - http://bmjopen.bmj.com/site/about/guidelines.xhtml

"care continuation" OR "care transition" OR "care transitions" OR "chain of care" OR "chains of care" OR "continuity of care" OR "cross sectoral care" OR "delivery of health care integrated" OR "integrated medicine" OR "integrated social network" OR "integrated social networks" OR "integration of care" OR "intersectoral care" OR "intrasectoral care" OR "linked care" OR "management model" OR "patient care management" OR "seamless care" OR "service network" OR "service networks" OR "transition of care" OR "transitional care" OR "transmural care" OR "whole system thinking" OR "holistic care")):ti,ab,kw (Word variations have been searched)

#7 (("Angola " OR "Benin " OR "Botswana " OR "Burkina Faso" OR "Upper Volta" OR "Burundi " OR "Cameroon " OR "Cameroons " OR "Cape Verde" OR "Central African Republic" OR Chad OR "Comoros " OR "Comoro Islands" OR "Comores" OR "Mayotte " OR "Congo " OR "Zaire " OR "Cote d'Ivoire" OR "Ivory Coast" OR "Democratic Republic of the Congo" OR "Djibouti " OR "French Somaliland" OR "Eritrea " OR "Ethiopia " OR "Gabon " OR "Gabonese Republic" OR "Gambia " OR "Ghana " OR "Gold Coast" OR "Guinea " OR "Kenya " OR "Lesotho" OR "Basutoland" OR "Liberia " OR "Madagascar " OR "Malagasy Republic " OR "Malawi " OR "Nyasaland " OR "Mali " OR "Mauritania " OR "Mauritius " OR "Mozambique" OR "Namibia" OR "Niger" OR "Nigeria" OR "Rwanda" OR "Sao Tome" OR "Seychelles" OR "Senegal " OR "Sierra Leone" OR "Somalia " OR "South Africa" OR "Sudan " OR "Eswatini" OR "Tanzania" OR "Togo " OR "Togolese Republic" OR "Uganda" OR "Zambia" OR "Zimbabwe " OR "Rhodesia" OR "Sub-Saharan Africa" OR "Africa South of the Sahara")):ti,ab,kw (Word variations have been searched)

#8 ((#1 OR #2 OR #3 OR #4 OR #5) AND #6 AND #7) AND Restricted (1999-2022, RCTs)

6. Psycinfo via Ovid

#1 ("elevated blood pressure"/ OR "high blood pressure"/ OR "raised blood pressure"/ OR "diastolic blood pressure"/ OR "systolic blood pressure"/ OR "arterial blood pressure"/ OR "uncontrolled blood pressure"/ OR "uncontrolled hypertension" OR "elevated blood pressure" OR "high blood pressure" OR "raised blood pressure" OR "diastolic blood pressure" OR "systolic blood pressure" OR "arterial blood pressure" OR "uncontrolled blood pressure" OR "uncontrolled hypertension")

#2 ("diabetes mellitus"/ OR "diabetes mellitus, type 2"/ OR "diabetes type 2"/ OR "T2DM"/ OR "diabetes type II"/ OR "diabetes"/ OR "glucose intolerance"/ OR "insulin resistance"/ OR "hyperglycemia"/ OR "hyperglycaemia"/ OR "hypoglycaemia"/ OR "hypoglycaemia"/ OR "high blood sugar"/ OR "elevated blood sugar"/ OR "raised blood sugar"/ OR "high blood glucose"/ OR "elevated blood glucose"/ OR "raised blood glucose"/ OR "diabetes mellitus" OR "diabetes mellitus, type 2" OR "diabetes type 2" OR "T2DM" OR "diabetes type II" OR "diabetes" OR "glucose intolerance" OR "insulin resistance" OR "hyperglycemia" OR "hyperglycaemia" OR "hypoglycemia" OR "hypoglycaemia" OR "high blood sugar" OR "elevated blood sugar" OR "raised blood sugar" OR "high blood glucose" OR "elevated blood glucose" OR "raised blood glucose")

#3 ("stroke"/ OR "transient ischemic attack"/ OR "ischemic attack"/ OR "angina"/ OR "angina pectoris"/ OR "heart attack"/ OR "ischemic heart diseases "/ OR "transient ischaemic attack"/ OR "coronary heart disease"/ OR "coronary disease"/ OR "heart failure"/ OR "peripheral vascular disease"/ OR "Peripheral Vascular diseases"/ OR "atrial fibrillation"/ OR "cardiovascular disease"/ OR "heart disease"/ OR "stroke" OR "transient ischemic attack" OR "ischemic attack" OR "angina" OR "angina pectoris" OR "heart attack" OR "ischemic heart diseases " OR "transient ischaemic attack" OR "coronary heart disease" OR "coronary disease" OR "heart failure" OR "peripheral vascular disease" OR "Peripheral Vascular diseases" OR "atrial fibrillation" OR "cardiovascular disease" OR "heart disease")

#4 ("Hypercholesterolemia"/ OR "Dyslipidemias"/ OR "blood lipid"/ OR "Cholesterol"/ OR "high cholesterol"/ OR "elevated cholesterol"/ OR "raised cholesterol"/ OR "low density lipoprotein "/ OR "high density lipoprotein"/ OR "Dyslipidemia"/ OR "Dyslipidaemia"/ OR "Hypercholesterolemia"/ OR "hypercholesterolaemia "/ OR "hypercholesterolimia "/ OR "Hypertriglyceridemia"/ OR "Hypertriglyceridemia"/ OR "Hypertriglyceridaemia"/ OR "Hyperlipidemia"/ OR "Hyperlipidemias"/ OR "Hyperlipidaemia"/ OR "LDL"/ OR "HDL"/ OR "Hypercholesterolemia" OR "Dyslipidemias" OR "blood lipid" OR "Cholesterol" OR "high cholesterol" OR "elevated cholesterol" OR "raised cholesterol" OR "low density lipoprotein " OR "high density lipoprotein" OR

For peer review only - http://bmjopen.bmj.com/site/about/guidelines.xhtml

"Dyslipidemia" OR "Dyslipidaemia" OR "Hypercholesterolemia" OR "hypercholesterolaemia " OR "hypercholesterolimia " OR "Hypertriglyceridemia" OR "Hypertriglyceridemia" OR "Hypertriglyceridaemia" OR "Hyperlipidemia" OR "Hyperlipidemias" OR "Hyperlipidaemia" OR "LDL" OR "HDL")

#5 ("Comorbid" OR "co-morbid" OR "co-morbidity" OR "Multimorbidity" OR "multi-morbid" OR "multi morbidity" OR "Multimorbidity" OR "Comorbidity" OR "multi-disease" OR "Multidisease" OR multi disease OR "multiple condition" OR "multi-condition" OR "multi condition" OR "multiple illness" OR "multi-illness" OR "multi illness" OR "multiple syndrome" OR "multi-syndrome" OR "multi syndrome" OR "concurrent condition" OR "concurrent illness" OR "concurrent disease" OR "co-existing disease" OR "coexisting disease" OR "co-existing illness" OR "coexisting illness" OR "co-existing syndrome" OR "coexisting syndrome" OR "co-existing condition" OR "coexisting condition" OR "co-occurring disease" OR "co-occuring disease" OR "co-occuring disease" OR "co-occurring illness" OR "co occurring illness" OR "cooccurring illness" OR "co-occurring syndrome" OR "co occurring syndrome" OR "cooccurring syndrome" OR "co-occurring condition" OR "co occurring condition" OR "cooccurring condition")

#6 ("Wagner model" OR "Wagner* model*" OR "Wagner* chronic care model*" OR "Wagner chronic care model" OR "chronic care model*" OR "model " OR "collaborative care" OR "chronic care framework" OR "chronic disease care" OR "chronic illness care" OR care model OR "model care" OR "Wagner* model*" OR "Wagner* chronic care model*" OR "Wagner chronic care model" OR "theory" OR "concept" OR "framework" OR "model" OR "programme" OR "approach" OR "clinical pathway" OR "care pathway" OR "critical path" OR "vertical integration" OR "virtual integration" OR "physician system integration" OR "provider system integration" OR "functional integration" OR "horizontal integration" OR "clinical integration" OR "case management" OR "delivery of health care, integrated" OR "disease management" OR "patient care management" OR "patient-centred care" OR "accountable care organisations" OR "continuity of patient care" OR "case management" OR "comprehensive health care" OR "delivery of health care, integrated" OR "managed care programmes" OR "patient-centred care" OR "care delivery" OR "integrated care" OR "comprehensive care" OR "care coordination" OR "managed care" OR "accountable care " OR "accountable care organisations" OR "accountable care organisation" OR "collaborative care" OR "disease management" OR "case-management" OR "case management" OR "shared care" OR "accountable care" OR "patient-centred" OR "patient centred" OR "person-centred" OR "person centred" OR "multidisciplinary care" OR "interdisciplinary care" OR "inter-disciplinary care" OR "cross disciplinary care" OR "cross-disciplinary care" OR "multiple interventions" OR "care chain" OR "care chains" OR "care continuity" OR "care continuation" OR "care transition" OR "care transitions" OR "chain of care" OR "chains of care" OR "continuity of care" OR "cross sectoral care" OR "delivery of health care integrated" OR "integrated medicine" OR "integrated social network" OR "integrated social networks" OR "integration of care" OR "intersectoral care" OR "intrasectoral care" OR "linked care" OR "management model" OR "patient care management" OR "seamless care" OR "service network" OR "service networks" OR "transition of care" OR "transitional care" OR "transmural care" OR "whole system thinking" OR "holistic care")

#7 ("Angola " OR "Benin " OR "Botswana " OR "Burkina Faso" OR "Upper Volta" OR "Burundi " OR "Cameroon " OR "Cameroons " OR "Cape Verde" OR "Central African Republic" OR Chad OR "Comoros " OR "Comoro Islands" OR "Comores" OR "Mayotte " OR "Congo " OR "Zaire " OR "Cote d'Ivoire" OR "Ivory Coast" OR "Democratic Republic of the Congo" OR "Djibouti " OR "French Somaliland" OR "Eritrea " OR "Ethiopia " OR "Gabon " OR "Gabonese Republic" OR "Gambia " OR "Ghana " OR "Gold Coast" OR "Guinea " OR "Kenya " OR "Lesotho" OR "Basutoland" OR "Liberia " OR "Madagascar " OR "Malagasy Republic " OR "Malawi " OR "Nyasaland " OR "Mali " OR "Mauritania " OR "Mauritius " OR "Mozambique" OR "Namibia" OR "Niger" OR "Nigeria" OR "Rwanda" OR "Sao Tome" OR "Seychelles" OR "Senegal " OR "Sierra Leone" OR "Somalia " OR "South Africa" OR "Sudan " OR "Eswatini" OR "Tanzania" OR "Togo " OR "Togolese Republic" OR "Uganda" OR "Zambia" OR "Zimbabwe " OR "Rhodesia" OR "Sub-Saharan Africa" OR "Africa South of the Sahara")

8 ((#1 OR #2 OR #3 OR #4 OR #5) AND #6 AND #7)

9 limit 8 to ("0300 clinical trial" and yr="1999 - 2022")

7. CINAHL

For peer review only - http://bmjopen.bmj.com/site/about/guidelines.xhtml

#1 ("elevated blood pressure" OR "high blood pressure" OR "raised blood pressure" OR "diastolic blood pressure" OR "systolic blood pressure" OR "arterial blood pressure" OR "uncontrolled blood pressure" OR "uncontrolled hypertension" OR "elevated blood pressure" OR "high blood pressure" OR "raised blood pressure" OR "diastolic blood pressure" OR "systolic blood pressure" OR "arterial blood pressure" OR "uncontrolled blood pressure" OR "uncontrolled hypertension")

#2 ("diabetes mellitus" OR "diabetes mellitus, type 2" OR "diabetes type 2" OR "T2DM" OR "diabetes type II" OR "diabetes" OR "glucose intolerance" OR "insulin resistance" OR "hyperglycemia" OR "hyperglycaemia" OR "hypoglycaemia" OR "hypoglycaemia" OR "high blood sugar" OR "elevated blood sugar" OR "raised blood sugar" OR "high blood glucose" OR "elevated blood glucose" OR "raised blood glucose" OR "diabetes mellitus" OR "diabetes mellitus, type 2" OR "diabetes type 2" OR "T2DM" OR "diabetes type II" OR "diabetes" OR "glucose intolerance" OR "insulin resistance" OR "hyperglycemia" OR "hyperglycaemia" OR "hypoglycemia" OR "hypoglycaemia" OR "high blood sugar" OR "elevated blood sugar" OR "raised blood sugar" OR "high blood glucose" OR "elevated blood glucose" OR "raised blood glucose")

#3 ("stroke" OR "transient ischemic attack" OR "ischemic attack" OR "angina" OR "angina pectoris" OR "heart attack" OR "ischemic heart diseases " OR "transient ischaemic attack" OR "coronary heart disease" OR "coronary disease" OR "heart failure" OR "peripheral vascular disease" OR "Peripheral Vascular diseases" OR "atrial fibrillation" OR "cardiovascular disease" OR "heart disease" OR "stroke" OR "transient ischemic attack" OR "ischemic attack" OR "angina" OR "angina pectoris" OR "heart attack" OR "ischemic heart diseases " OR "transient ischaemic attack" OR "coronary heart disease" OR "coronary disease" OR "heart failure" OR "peripheral vascular disease" OR "Peripheral Vascular diseases" OR "atrial fibrillation" OR "cardiovascular disease" OR "heart disease")

#4 ("Hypercholesterolemia" OR "Dyslipidemias" OR "blood lipid" OR "Cholesterol" OR "high cholesterol" OR "elevated cholesterol" OR "raised cholesterol" OR "low density lipoprotein " OR "high density lipoprotein" OR "Dyslipidemia" OR "Dyslipidaemia" OR "Hypercholesterolemia" OR "hypercholesterolaemia " OR "hypercholesterolimia " OR "Hypertriglyceridemia" OR "Hypertriglyceridemia" OR "Hypertriglyceridaemia" OR "Hyperlipidemia" OR "Hyperlipidemias" OR "Hyperlipidaemia" OR "LDL" OR "HDL" OR "Hypercholesterolemia" OR "Dyslipidemias" OR "blood lipid" OR "Cholesterol" OR "high cholesterol" OR "elevated cholesterol" OR "raised cholesterol" OR "low density lipoprotein " OR "high density lipoprotein" OR "Dyslipidemia" OR "Dyslipidaemia" OR "Hypercholesterolemia" OR "hypercholesterolaemia " OR "hypercholesterolimia " OR "Hypertriglyceridemia" OR "Hypertriglyceridemia" OR "Hypertriglyceridaemia" OR "Hyperlipidemia" OR "Hyperlipidemias" OR "Hyperlipidaemia" OR "LDL" OR "HDL")

#5 ("Comorbid" OR "co-morbid" OR "co-morbidity" OR "Multimorbidity" OR "multi-morbid" OR "multi morbidity" OR "Multimorbidity" OR "Comorbidity" OR "multi-disease" OR "Multidisease" OR multi disease OR "multiple condition" OR "multi-condition" OR "multi condition" OR "multiple illness" OR "multi-illness" OR "multi illness" OR "multiple syndrome" OR "multi-syndrome" OR "multi syndrome" OR "concurrent condition" OR "concurrent illness" OR "concurrent disease" OR "co-existing disease" OR "coexisting disease" OR "co-existing illness" OR "coexisting illness" OR "co-existing syndrome" OR "coexisting syndrome" OR "co-existing condition" OR "coexisting condition" OR "co-occurring disease" OR "co-occuring disease" OR "co-occuring disease" OR "co-occurring illness" OR "co occurring illness" OR "cooccurring illness" OR "co-occurring syndrome" OR "co occurring syndrome" OR "cooccurring syndrome" OR "co-occurring condition" OR "co occurring condition" OR "cooccurring condition")

#6 ("Wagner model" OR "Wagner* model*" OR "Wagner* chronic care model*" OR "Wagner chronic care model" OR "chronic care model*" OR "model " OR "collaborative care" OR "chronic care framework" OR "chronic disease care" OR "chronic illness care" OR care model OR "model care" OR "Wagner* model*" OR "Wagner* chronic care model*" OR "Wagner chronic care model" OR "theory" OR "concept" OR "framework" OR "model" OR "programme" OR "approach" OR "clinical pathway" OR "care pathway" OR "critical path" OR "vertical integration" OR "virtual integration" OR "physician system integration" OR "provider system integration" OR "functional integration" OR "horizontal integration" OR "clinical integration" OR "case management" OR "delivery of health care, integrated" OR "disease management" OR "patient care management" OR "patient-centred care" OR "accountable care organisations" OR "continuity of patient care" OR "case management" OR "comprehensive health

For peer review only - http://bmjopen.bmj.com/site/about/guidelines.xhtml

care" OR "delivery of health care, integrated" OR "managed care programmes" OR "patient-centred care" OR "care delivery" OR "integrated care" OR "comprehensive care" OR "care coordination" OR "managed care" OR "accountable care " OR "accountable care organisations" OR "accountable care organisation" OR "collaborative care" OR "disease management" OR "case-management" OR "case management" OR "shared care" OR "accountable care" OR "patient-centred" OR "patient centred" OR "person-centred" OR "person centred" OR "multidisciplinary care" OR "interdisciplinary care" OR "inter-disciplinary care" OR "cross disciplinary care" OR "cross-disciplinary care" OR "multiple interventions" OR "care chain" OR "care chains" OR "care continuity" OR "care continuation" OR "care transition" OR "care transitions" OR "chain of care" OR "chains of care" OR "continuity of care" OR "cross sectoral care" OR "delivery of health care integrated" OR "integrated medicine" OR "integrated social network" OR "integrated social networks" OR "integration of care" OR "intersectoral care" OR "intrasectoral care" OR "linked care" OR "management model" OR "patient care management" OR "seamless care" OR "service network" OR "service networks" OR "transition of care" OR "transitional care" OR "transmural care" OR "whole system thinking" OR "holistic care")

#7 ("Angola " OR "Benin " OR "Botswana " OR "Burkina Faso" OR "Upper Volta" OR "Burundi " OR "Cameroon " OR "Cameroons " OR "Cape Verde" OR "Central African Republic" OR Chad OR "Comoros " OR "Comoro Islands" OR "Comores" OR "Mayotte " OR "Congo " OR "Zaire " OR "Cote d'Ivoire" OR "Ivory Coast" OR "Democratic Republic of the Congo" OR "Djibouti " OR "French Somaliland" OR "Eritrea " OR "Ethiopia " OR "Gabon " OR "Gabonese Republic" OR "Gambia " OR "Ghana " OR "Gold Coast" OR "Guinea " OR "Kenya " OR "Lesotho" OR "Basutoland" OR "Liberia " OR "Madagascar " OR "Malagasy Republic " OR "Malawi " OR "Nyasaland " OR "Mali " OR "Mauritania " OR "Mauritius " OR "Mozambique" OR "Namibia" OR "Niger" OR "Nigeria" OR "Rwanda" OR "Sao Tome" OR "Seychelles" OR "Senegal " OR "Sierra Leone" OR "Somalia " OR "South Africa" OR "Sudan " OR "Eswatini" OR "Tanzania" OR "Togo " OR "Togolese Republic" OR "Uganda" OR "Zambia" OR "Zimbabwe " OR "Rhodesia" OR "Sub-Saharan Africa" OR "Africa South of the Sahara")

#8 ((S1 OR S2 OR S3 OR S4 OR S5) AND S6 AND S7) & 1999-2022 & research article& peer-reviewed

Online supplementary file 3: Funnel plot of the random effects meta-analysis of SBP for integrated versus standard care for cardiometabolic multimorbidity in Sub-Saharan Africa

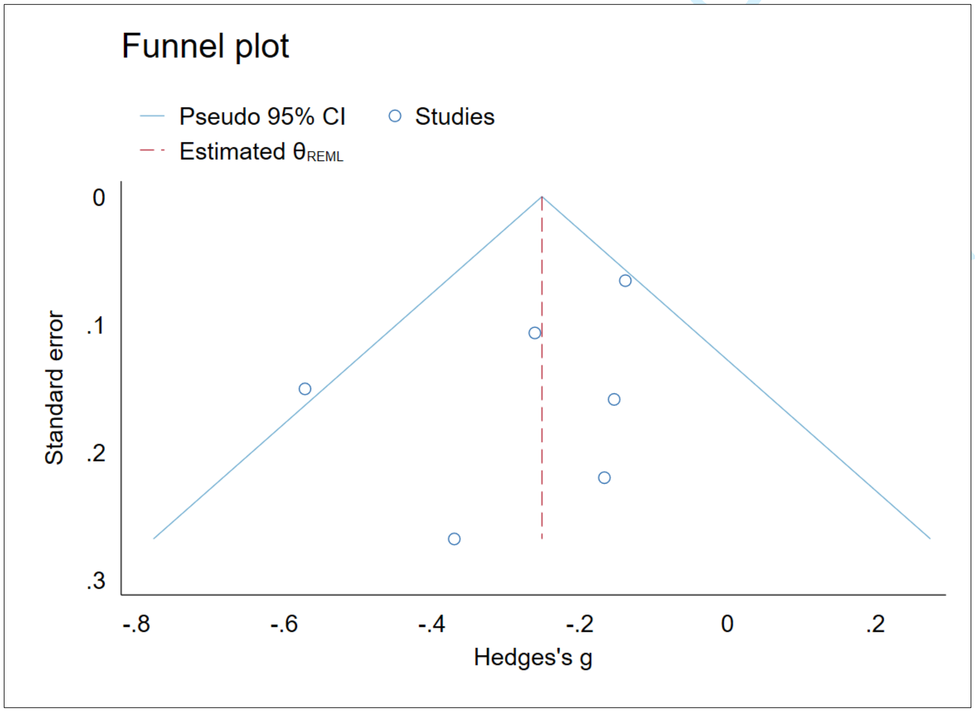

For peer review only - http://bmjopen.bmj.com/site/about/guidelines.xhtml

PRISMA 2020 Checklist

Section and Topic	Item #	Checklist item	Location (Page) where item is reported
**TITLE : Effectiveness of integrated chronic care models for cardiometabolic multimorbidity in Sub-Saharan Africa: A systematic review and meta-analysis**			
Title	1	Identify the report as a systematic review.	1
**ABSTRACT**			
Abstract	2	See the PRISMA 2020 for Abstracts checklist.	2
**INTRODUCTION**			
Rationale	3	Describe the rationale for the review in the context of existing knowledge.	4
Objectives	4	Provide an explicit statement of the objective(s) or question(s) the review addresses.	5
**METHODS**			
Eligibility criteria	5	Specify the inclusion and exclusion criteria for the review and how studies were grouped for the syntheses.	7
Information sources	6	Specify all databases, registers, websites, organisations, reference lists and other sources searched or consulted to identify studies. Specify the date when each source was last searched or consulted.	7
Search strategy	7	Present the full search strategies for all databases, registers and websites, including any filters and limits used.	7
Selection process	8	Specify the methods used to decide whether a study met the inclusion criteria of the review, including how many reviewers screened each record and each report retrieved, whether they worked independently, and if applicable, details of automation tools used in the process.	8
Data collection process	9	Specify the methods used to collect data from reports, including how many reviewers collected data from each report, whether they worked independently, any processes for obtaining or confirming data from study investigators, and if applicable, details of automation tools used in the process.	8
Data items	10a	List and define all outcomes for which data were sought. Specify whether all results that were compatible with each outcome domain in each study were sought (e.g. for all measures, time points, analyses), and if not, the methods used to decide which results to collect.	7
	10b	List and define all other variables for which data were sought (e.g. participant and intervention characteristics, funding sources). Describe any assumptions made about any missing or unclear information.	7
Study risk of bias assessment	11	Specify the methods used to assess risk of bias in the included studies, including details of the tool(s) used, how many reviewers assessed each study and whether they worked independently, and if applicable, details of automation tools used in the process.	8
Effect measures	12	Specify for each outcome the effect measure(s) (e.g. risk ratio, mean difference) used in the synthesis or presentation of results.	9
Synthesis methods	13a	Describe the processes used to decide which studies were eligible for each synthesis (e.g. tabulating the study intervention characteristics and comparing against the planned groups for each synthesis (item #5)).	9
	13b	Describe any methods required to prepare the data for presentation or synthesis, such as handling of missing summary statistics, or data conversions.	9
	13c	Describe any methods used to tabulate or visually display results of individual studies and syntheses.	9
	13d	Describe any methods used to synthesize results and provide a rationale for the choice(s). If meta-analysis was performed, describe the model(s), method(s) to identify the presence and extent of statistical heterogeneity, and software package(s) used.	9
	13e	Describe any methods used to explore possible causes of heterogeneity among study results (e.g. subgroup analysis, meta-regression).	N/A
	13f	Describe any sensitivity analyses conducted to assess robustness of the synthesized results.	9
Reporting bias	14	Describe any methods used to assess risk of bias due to missing results in a synthesis (arising from reporting biases).	9

For peer review only

PRISMA 2020 Checklist

Section and Topic	Item #	Checklist item	Location (Page) where item is reported
assessment			
Certainty assessment	15	Describe any methods used to assess certainty (or confidence) in the body of evidence for an outcome.	9
**RESULTS**			
Study selection	16a	Describe the results of the search and selection process, from the number of records identified in the search to the number of studies included in the review, ideally using a flow diagram.	10
	16b	Cite studies that might appear to meet the inclusion criteria, but which were excluded, and explain why they were excluded.	10
Study characteristics	17	Cite each included study and present its characteristics.	10
Risk of bias in studies	18	Present assessments of risk of bias for each included study.	12
Results of individual studies	19	For all outcomes, present, for each study: (a) summary statistics for each group (where appropriate) and (b) an effect estimate and its precision (e.g. confidence/credible interval), ideally using structured tables or plots.	14
Results of syntheses	20a	For each synthesis, briefly summarise the characteristics and risk of bias among contributing studies.	12
	20b	Present results of all statistical syntheses conducted. If meta-analysis was done, present for each the summary estimate and its precision (e.g. confidence/credible interval) and measures of statistical heterogeneity. If comparing groups, describe the direction of the effect.	14
	20c	Present results of all investigations of possible causes of heterogeneity among study results.	14
	20d	Present results of all sensitivity analyses conducted to assess the robustness of the synthesized results.	14
Reporting biases	21	Present assessments of risk of bias due to missing results (arising from reporting biases) for each synthesis assessed.	14
Certainty of evidence	22	Present assessments of certainty (or confidence) in the body of evidence for each outcome assessed.	14
**DISCUSSION**			
Discussion	23a	Provide a general interpretation of the results in the context of other evidence.	15-16
	23b	Discuss any limitations of the evidence included in the review.	16
	23c	Discuss any limitations of the review processes used.	16
	23d	Discuss implications of the results for practice, policy, and future research.	17
**OTHER INFORMATION**			
Registration and protocol	24a	Provide registration information for the review, including register name and registration number, or state that the review was not registered.	5
	24b	Indicate where the review protocol can be accessed, or state that a protocol was not prepared.	The protocol was not published
	24c	Describe and explain any amendments to information provided at registration or in the protocol.	N/A
Support	25	Describe sources of financial or non-financial support for the review, and the role of the funders or sponsors in the review.	17
Competing interests	26	Declare any competing interests of review authors.	17-18

For peer review only

For peer review only - http://bmjopen.bmj.com/site/about/guidelines.xhtml

PRISMA 2020 Checklist

Section and Topic	Item #	Checklist item	Location (Page) where item is reported
Availability of data, code and other materials	27	Report which of the following are publicly available and where they can be found: template data collection forms; data extracted from included studies; data used for all analyses; analytic code; any other materials used in the review.	17-18

From: Page MJ, McKenzie JE, Bossuyt PM, Boutron I, Hoffmann TC, Mulrow CD, et al. The PRISMA 2020 statement: an updated guideline for reporting systematic reviews. BMJ 2021;372:n71. doi: 10.1136/bmj.n71

For more information, visit: http://www.prisma-statement.org/

For peer review only

BMJ Open

Effectiveness of integrated chronic care models for cardiometabolic multimorbidity in Sub-Saharan Africa: A systematic review and meta-analysis

Journal:	*BMJ Open*
Manuscript ID	bmjopen-2023-073652.R1
Article Type:	Original research
Date Submitted by the Author:	26-May-2023

Complete List of Authors:	Otieno, Peter; African Population and Health Research Center, Chronic Disease Management Uninit; Amsterdam UMC Locatie AMC, Department of Public & Occupational Health<br>Agyemang, Charles ; Amsterdam UMC Locatie AMC, Department of Public & Occupational Health<br>Wao, Hesborn; African Population and Health Research Center, Research and Related Capacity Strengthening<br>Wambiya, Elvis; The University of Sheffield, School of Health and Related Research<br>Ng'oda, Maurine; African Population and Health Research Center<br>Mwanga, Daniel; African Population and Health Research Center<br>Oguta, James; The University of Sheffield, School of Health and Related Research<br>Kibe , Peter M. ; African Population and Health Research Center<br>Asiki, G; African Population and Health Research Center, Chronic Disease Management Unit; Karolinska Institutet, Women's and Children's Health

**Primary Subject Heading**:	Cardiovascular medicine
Secondary Subject Heading:	Cardiovascular medicine
Keywords:	Hypertension < CARDIOLOGY, Patient-Centered Care, Primary Health Care, Self Care

SCHOLARONE™
Manuscripts

I, the Submitting Author has the right to grant and does grant on behalf of all authors of the Work (as defined in the below author licence), an exclusive licence and/or a non-exclusive licence for contributions from authors who are: i) UK Crown employees; ii) where BMJ has agreed a CC-BY licence shall apply, and/or iii) in accordance with the terms applicable for US Federal Government officers or employees acting as part of their official duties; on a worldwide, perpetual, irrevocable, royalty-free basis to BMJ Publishing Group Ltd ("BMJ") its licensees and where the relevant Journal is co-owned by BMJ to the co-owners of the Journal, to publish the Work in this journal and any other BMJ products and to exploit all rights, as set out in our licence.

The Submitting Author accepts and understands that any supply made under these terms is made by BMJ to the Submitting Author unless you are acting as an employee on behalf of your employer or a postgraduate student of an affiliated institution which is paying any applicable article publishing charge ("APC") for Open Access articles. Where the Submitting Author wishes to make the Work available on an Open Access basis (and intends to pay the relevant APC), the terms of reuse of such Open Access shall be governed by a Creative Commons licence – details of these licences and which Creative Commons licence will apply to this Work are set out in our licence referred to above.

Other than as permitted in any relevant BMJ Author's Self Archiving Policies, I confirm this Work has not been accepted for publication elsewhere, is not being considered for publication elsewhere and does not duplicate material already published. I confirm all authors consent to publication of this Work and authorise the granting of this licence.

For peer review only

Effectiveness of integrated chronic care models for cardiometabolic multimorbidity in Sub-Saharan Africa: A systematic review and meta-analysis

Peter Otieno*[1,2,3,6]
Charles Agyemang[2]
Hesborn Wao[1]
Elvis Wambiya[5 6]
Maurine Ng'oda[1]
Daniel Mwanga[1]
James Oguta[5 6]
Peter Kibe[1. 6]
Gershim Asiki[1,4]

1 African Population and Health Research Center P.O. Box: 10787-00100, Nairobi, Kenya.
2 Department of Public & Occupational Health, Amsterdam UMC, University of Amsterdam, Amsterdam Public Health Research Institute, Amsterdam, The Netherlands.
3 Amsterdam Institute for Global Health and Development (AIGHD), AHTC, Tower C4, Paasheuvelweg 25, 1105 BP Amsterdam, Netherlands.
4 Department of Women's and Children's Health, Karolinska Institutet, Stockholm, Sweden
5 School of Health and Related Research, University of Sheffield. Sheffield, S1 4DA, United Kingdom
6 African Network of Research Scientists, Nairobi, Kenya

* Corresponding author:
Peter Otieno
African Population and Health Research Center,
P.O. Box: 10787-00100, Nairobi, Kenya.
Email: pootienoh@gmail.com
Telephone: +254 (20) 400 1000

Word count: 4, 633

For peer review only

Abstract

Objective: This review aimed at identifying the elements of integrated care models for cardiometabolic multimorbidity in Sub-Saharan Africa (SSA) and their effects on clinical or mental health outcomes including systolic blood pressure (SBP), blood sugar, depression scores and other patient-reported outcomes such as quality of life, and medication adherence.

Design: We systematically searched PubMed, Embase, Scopus, Web of Science, Global Health CINAHL, African Journals Online, Informit, PsycINFO, clinicaltrials.gov, Pan African Clinical Trials Registry and grey literature from OpenSIGLE for studies published between 1999 and 2022. We included randomized controlled trial studies featuring integrated care models with two or more elements of Wagner's chronic care model.

Setting: We selected studies conducted in SSA among adults aged 18 years and over with cardiometabolic multimorbidity receiving care in primary or community care settings.

Outcomes: The outcome measures included SBP, glycated haemoglobin (HbA1c), depression, medication adherence, and quality of life.

Results: In all, we included 10 randomized controlled trials from 11 publications with 4,864 participants from seven SSA countries (South Africa, Kenya, Nigeria, Eswatini, Ghana and Uganda). The overall quality of evidence based on GRADE criteria was moderate. A random-effects meta-analysis of six studies involving 1,754 participants shows that integrated compared to standard care conferred a moderately lower mean SBP (Mean Difference=-4.85mmHg, 95% CI: -7.37 to -2.34) for people with cardiometabolic multimorbidity; Hedges' g effect size (g=-0.25, (-0.39 to -0.11). However, integrated care compared with usual care showed mixed results for HbA1c, depression, medication adherence, and quality of life.

Conclusions: Integrated care improved SBP among patients living with cardiometabolic multimorbidity in SSA. More studies on integrated care are required to improve the evidence pool on chronic care models for multimorbidities. These include implementation studies, cost-effectiveness studies; and more trials with outcomes on mental health, medication adherence, and health-related quality of life.

Keywords: Integrated care, chronic care models, multimorbidity, cardiometabolic diseases, Sub-Saharan Africa

PROSPERO registration number: CRD42020187756

For peer review only - http://bmjopen.bmj.com/site/about/guidelines.xhtml

Article Summary:

Strengths and limitations of this study

- The inclusion of randomized controlled trials provides more unbiased findings than previous reviews that are mostly based on observational study designs.
- The extraction of important constructs of Wagner's chronic care model from the integrated care models offers deeper insights into the effectiveness of integrated care model elements.
- The review considered both communicable and non-communicable diseases which reflects their converging burdens in Sub-Saharan Africa.
- The varying numbers and types of chronic care model components resulted in heterogeneity in the included studies.
- Since most included studies used multi-component interventions, the absolute effect attributable to a particular element of the chronic care model remains unknown.

For peer review only

For peer review only - http://bmjopen.bmj.com/site/about/guidelines.xhtml

Background

Cardiometabolic diseases, including type 2 diabetes and cardiovascular diseases (CVDs), are the leading cause of global mortality [1]. People living with cardiometabolic diseases often have multiple rather than a single condition, commonly known as multimorbidity [2]. Cardiometabolic multimorbidity occurs against the background of obesity and insulin resistance [3]. However, these conditions are also characterised by the simultaneous co-occurrence of other diseases with unrelated pathophysiology or care management profile such as HIV/AIDS, chronic respiratory diseases, cancers, and mental illnesses, a phenomenon referred to as discordant multimorbidity [4-6].

The burden of multimorbidity is highest in low- and middle-income countries (LMICs), which still bear a high burden of infectious diseases while also facing a new threat from non-communicable diseases [7]. However, the majority of the chronic care models in Sub-Saharan Africa (SSA) are still built around single disease frameworks [8]. Evidence shows that health services delivery based on isolated interventions may derail the value of treatment and preventive measures [9-12]. Vertical disease programs result in missed opportunities for early diagnosis and management of multimorbidities [13]. Therefore, integrated chronic disease management is recommended to respond to this challenge [14]. Singer et al, (2011) [15], define *integrated care* as a set of patient-centred and multidisciplinary care activities coordinated by two or more collaborating service providers within or across the healthcare sector including community and social environments. This definition emphasizes the coordination of care activities and the active involvement of patients in managing their health.

The chronic care model, first identified by McColl Institute for Healthcare Innovation at Group Health Cooperative [11], has been proposed as a solution to improve the integrated management of chronic diseases. This model identifies six essential elements: community resources and policies, health care organization, self-management support, delivery system design, decision support and clinical information systems. However, what constitutes an integrated chronic care model and how it is implemented and delivered within healthcare services, has continued to evolve with the advent of new interventions. Most of the chronic care models have been developed in high-income countries with sophisticated health services [16], which may not appropriately apply to SSA.

The untapped capacity of chronic care models for HIV/AIDS care in SSA should be viewed as emerging models for other chronic diseases including cardiometabolic diseases [17]. Lessons learned from the implementation of care models for HIV/AIDS and tuberculosis in SSA could be effectively applied to improve care models for cardiometabolic diseases [18]. A systematic review by Rohwer et al [19] examined

For peer review only

existing integrated care models for diabetes and hypertension in LMICs. However, the included studies did not focus on structured clinical care delivered to persons living with multimorbidity. Furthermore, the integrated care for hypertension and diabetes was based on a 'one-stop-shop' model where all healthcare services are provided under one roof [19]. This is just one way of describing integrated care. A comprehensive evaluation of the applicability of the elements of chronic care models for cardiometabolic multimorbidity has not been systematically evaluated in SSA. Therefore, the purpose of this systematic review was to identify elements of integrated chronic care models for cardiometabolic multimorbidity in SSA and their effects on clinical or mental health outcomes including systolic blood pressure (SBP), blood sugar, depression scores and other patient-reported outcomes such as quality of life, and medication adherence.

Methods

Protocol registration

The study protocol was registered in the International Prospective Register for Systematic Reviews (PROSPERO) (CRD42020187756). The findings are reported according to the Preferred Reporting Items for Systematic Reviews (PRISMA) [20].

Eligibility criteria

Participants

We selected studies conducted in SSA among adult patients aged 18 years and over with cardiometabolic multimorbidity receiving care in a primary or community care setting. Cardiometabolic multimorbidity was defined as having two or more chronic conditions; at least one of which is a cardiometabolic disease such as type 2 diabetes, hypertension, hypercholesterolemia, hypertriglyceridemia, dyslipidaemia or CVDs such as stroke. Others included common discordant conditions such as HIV/AIDS, chronic respiratory diseases, cancers and mental illnesses. These conditions constitute over three-quarters of the global burden of chronic diseases [21].

Intervention

We included interventions with integrated chronic care models for persons living with cardiometabolic multimorbidity. In line with previous studies [22-26], we classified the intervention components into six categories based on the elements of Wagner's chronic care model [11]. Thus, for the inclusion criterion, the models were considered "integrated care" if they applied at least two of the six chronic care model elements to

For peer review only - http://bmjopen.bmj.com/site/about/guidelines.xhtml

manage more than one chronic disease [23]. This included self-management support, delivery system design, decision support, clinical information system, healthcare organization, and community linkages.

The classification of intervention features and components is shown in Supplementary File 1. Self-management support includes interventions that empower people living with chronic conditions to manage their health. Examples are self-help groups and technological aids for self-care. Delivery system design interventions focus on care delivery efficiency and effectiveness such as patient care planning, coordination, and follow-up. Decision-support interventions promote clinical care that is consistent with scientific evidence and patient preferences. Clinical information systems is the organization of patient data to facilitate efficient and effective care. Healthcare organization interventions focus on creating an organizational culture and mechanisms that promote safe, high-quality care. Community linkages include interventions that mobilize community resources to meet the needs of people living with chronic diseases. Where the chronic care model had multiple components, we defined each element using the Wagner taxonomy [11] and highlighted the predominant components.

Control

The control or comparison group in the included studies received the usual or standard care comprising standalone services for cardiometabolic multimorbidity.

Outcome

We included studies that reported any objective measure of patient clinical or mental health outcomes. For example, SBP (mmHg), glycated haemoglobin ((HbA1c (%)), and depression scores. Others included patient-reported outcomes such as quality of life, and patient behaviour (medication adherence).

Types of studies

We included randomised controlled trials (RCTs) meeting the quality criteria developed by the Cochrane Effective Practice and Organization of Care (EPOC) [27]. This is because RCTs provide more unbiased information than other study designs on the differential effects of chronic care models. Studies from SSA published in all languages between 1999 and December 2022 were included. We used 1999 because this was the formal year of inception of the Wagner's chronic care model [28].

For peer review only - http://bmjopen.bmj.com/site/about/guidelines.xhtml

Exclusion criteria

We excluded single-disease-focused studies and studies conducted among pregnant women. Interventions with no specified structured clinical care delivered to persons living with cardiometabolic multimorbidity were excluded. Studies with interventions for comorbid conditions where the intervention was targeted solely at one of the conditions rather than integrated care for the co-occurring conditions were also excluded. Studies that assumed multimorbidity to be the norm based on individuals' age without screening and diagnosis by qualified healthcare providers were excluded as the interventions were not being targeted specifically at multimorbidity.

Search strategy

Two reviewers independently searched PubMed, Embase, Scopus, Web of Science, Cumulative Index to Nursing and Allied Health Literature (CINAHL), Cochrane library, African Journals Online, Informit, and PsycINFO without language restriction. The search was limited to articles indexed from 1999 up to 15th December 2022 to capture the beginning of the application of the elements of Wagner's chronic care model. The search results were limited by filters for randomized control trials and search concepts for chronic care models, cardiometabolic diseases and multimorbidity (See supplementary file 2). Additional articles were identified by scanning the reference list of relevant studies obtained through the search. Hand-searching of key journals was also conducted. Furthermore, we searched for registered trials in the clinicaltrials.gov, the Pan African Clinical Trials Registry and grey literature from OpenSIGLE.

Data collection and analysis

Selection of studies

The search results were uploaded into the Endnote version 12 reference manager (Clarivate, Philadelphia, USA) and screened for duplicates. Four review authors (EW, MN, PK and DM) independently performed the initial screening of titles and abstracts. Results of screening were recorded against the citation in Excel spreadsheet. The articles selected for full-text review were subsequently assessed for adequacy following the inclusion criteria for patient, intervention, comparator and outcome. Consensus meetings were held between the reviewers and the project coordinator (PO) to resolve disagreements. We used a modified PRISMA flow chart to describe the study selection (Figure 1).

Data extraction and management

Four review authors (EW, MN, PK and DM) abstracted data using a modified version of the EPOC data collection checklist [27]. Data were entered in Excel spreadsheets and exported to Stata version 15 (StataCorp LLC). Data quality disagreements were resolved by consensus between the review authors and the project coordinator (PO). Information extracted from the included studies comprised:

1. Participants: type of patients, age, nature of multimorbidity and how it was diagnosed.

For peer review only - http://bmjopen.bmj.com/site/about/guidelines.xhtml

2. Study design: randomization, the unit of allocation, and follow-up.
3. Intervention: we extracted a full description of the intervention components and features. This included self-management support, delivery system design, decision support, clinical information system, healthcare organization, and community resources
4. Service providers: specialists, primary care providers, and family members
5. Study setting: primary care and community-based care
6. Patient clinical or mental health outcomes such as SBP, blood glucose control, and depression symptom scores. Others included patient-reported outcomes such as quality of life and medication adherence.
7. Results: We organized results into clinical outcomes, mental health outcomes and patient-reported outcomes.

Assessment of risk of bias

Four review authors (EA, MN, PK and DM) independently assessed the risk of bias in the included studies using standard EPOC criteria [29]. The assessment domains included: allocation (sequence generation and concealment); baseline characteristics; incomplete outcome data; contamination; blinding; selective outcome reporting; and other potential sources of bias. The criteria for the assessment were 'low risk', 'high risk' or 'unclear risk' (Figure 2). Loss to follow-up of more than 20% was considered a high risk of bias. We also considered loss to follow-up as a high risk of bias when it was unbalanced between the groups. Published protocols and trial registrations were tracked to assess selective reporting bias. We assessed incomplete reporting for studies that reported different results than the outcomes outlined in the methods sections of selected articles. Any disagreements between the review authors were resolved by consensus, or with the consultation of the project coordinator (PO).

Data analysis

We used a random-effects meta-analysis to estimate the pooled mean effect size of integrated care versus standard care on SBP. The random-effects meta-analysis allows for statistical heterogeneity between studies by assuming that the true effects in the individual studies differ from each other. Six studies with similarities in terms of the patient population, interventions, and outcome assessment, were included in the meta-analysis. Studies with significant heterogeneity were not included in the meta-analysis. Hence, we conducted a structured synthesis of the results. All analyses were performed using *"metan"* command in Stata version 15 (StataCorp LLC).

For peer review only - http://bmjopen.bmj.com/site/about/guidelines.xhtml

We calculated the effect size following the methods described by Hedges [30]. For each of the included studies, we calculated results in terms of absolute and standardized mean differences with a 95% Confidence Interval (CI). The heterogeneity of the selected studies were evaluated using χ^2 and I^2 statistics [31]. Using the χ^2 test, heterogeneity between studies was considered significant if $p<0.10$ [32]. We used I^2 statistics, values of 25%-49%, 50%-74% and >75% to determine low, moderate and high heterogeneity respectively [33]. Publication bias was assessed using the Doi plot and Luis Furuya Kanamori (LFK) index [34]. The LFK index outside the interval −1 and 1 was considered consistent with asymmetry [35]. As Higgins et al. [36] posit, it is justifiable to perform a pooled meta-analysis involving cluster RCTs and individual RCTs. However, sensitivity analyses should be performed to test the robustness of the pooled estimates. [36, 37]. We performed sensitivity analyses to assess the robustness of our results by comparing the pooled effect estimates from separate analyses involving RCTs (four studies) and cluster RCTs (two studies).

Certainty of evidence

The certainty of the evidence of included studies was assessed using the Grading of Recommendations Assessment, Development and Evaluation (GRADE) methodological guideline. According to GRADE guidance, RCTs start with high-certainty evidence [38]. Our judgements to downgrade the certainty of evidence were based on the assessment of the following five domains: study limitations, inconsistency, imprecision, indirectness and publication bias. For each outcome, we described the certainty of evidence as high, moderate, low or very low. The GRADE criteria also take into consideration the certainty of evidence and the size of the effect [39]. We considered a 5 mmHg reduction in SBP to be clinically significant [40]. A change of 0.5% for the outcome HbA1c, and 10% for depression scores, medication adherence and quality of life were considered clinically significant [41]. For each outcome, we described the certainty of evidence as high, moderate, low or very low [42].

Patient and public involvement

Patients or the public were not involved in the design, conduct, reporting, or dissemination of this research.

For peer review only - http://bmjopen.bmj.com/site/about/guidelines.xhtml

Results

Search results

The PRISMA flow diagram of the article selection process is presented in Figure 1. We identified 2,649 studies from our searches. Another 17 studies were included after manually searching the reference list and grey literature. After the removal of 806 duplicate records, we screened the titles and abstracts of 1,860 articles and identified 32 potentially eligible studies. We reviewed the full text of 32 studies and excluded 22 studies. Thus, 10 studies from 11 publications were included in this review (6 randomized controlled trials and 4 cluster randomized trials). Of these 10 studies, 6 were included in the meta-analyses and 4 studies from 5 publications were included in the structured synthesis of the results.

[Figure 1 approximately here]

Characteristics of included studies

The characteristics of the included studies are presented in Table 1. We included 10 trials from 11 publications [43-53] with a total of 4,864 participants. Sample sizes ranged from 55 to 1,174 participants. Three studies were conducted in South Africa, two in Kenya and Nigeria and one in Eswatini and Ghana, respectively. There was one multi-country study conducted in Uganda and Kenya. Studies mostly took place in primary care settings (n=9) or both community and primary care settings (n=2). Most trials recruited participants with HIV and cardiometabolic diseases (n=9), another two recruited patients with HIV, type 2 diabetes, hypertension, depression and substance use disorder. Eight studies lasted 12 months or more. In all the included studies, the control group received the usual standard care while the intervention group received structured integrated care with varying elements of chronic care models including self-management support, delivery system design, decision support, clinical information system, healthcare organization, and community resources.

For peer review only

Table 1: Characteristics of included studies

Study	Setting	Study design	Sample size	Age, years	Multimorbidity	Follow-up (months)	Outcome	Intervention
Havlir et al (2019) [43] Uganda and Kenya	Primary and community-based care	Cluster RCT	1,441 patients with hypertension & HIV	≥30	Hypertension and HIV	36	BP control	Multi-disease testing; integrated care for HIV and hypertension; structured follow-up, flexible hours of operation and reduced wait time at clinics; and patient incentives.
Jackson et al (2021) [44] Nigeria	Primary care	RCT	182	18-69	Hypertension & HIV	12	SBP and medication adherence	Pharmaceutical care with structured education and counselling by a pharmacist after seeing a physician on their clinic visit. Education focused on self-monitoring of BP, lifestyle modification, reviewing the date of the next appointment and prescription.
Jackson et al (2022) [45] Nigeria	Primary care	RCT	182	18-69	Hypertension and HIV	12	Health-related quality of life	Structured education and counselling on general self-care, medicines use storage and lifestyle modifications.
Myers et al (2022) [46] South Africa	Primary care	Cluster RCT	1,174	≥18	HIV, Type 2 diabetes, depression and substance use disorder	12	Depression scores	Psychological interventions comprising motivational interviewing and problem-solving therapy delivered by a trained facility-based community health worker.
Okube et al (2022) [47] Kenya	Community-based care	RCT	294	18-64	Metabolic syndrome	12	BP and blood sugar control	Community-based health education on lifestyle modification and face-to-face delivery of verbal and written individualized health recommendations on risk factors for CVDs.
Owolabi et al 2019 [48] Nigeria	Primary care	RCT	158	≥18	Hypertension and stroke	12	SBP	A culturally appropriate, multipronged intervention comprising patient global risk factor control report card, personalized phone text-messaging, and educational video.
Petersen et al (2021) [49] South Africa	Primary care	Cluster RCT	925	≥18	Hypertension and depression	12	Depression scores	Supplementary training of primary nurses and doctors on mental health and clinical communication skills. Collaborative care model for patients with hypertension and comorbid depressive symptoms including doctors, nurses, clinical psychologists and lay counsellors.
Rabkin et al (2018) [50] Eswatini	Primary care	RCT	236	≥40	HIV, Hypertension, type 2 diabetes, and hyperlipidemia	6	SBP and HbA1c	CVD risk factors screening and structured referrals among patients living with HIV.
Roos et al 2014 [51] South Africa	Primary care	RCT	84	20-65	HIV and metabolic diseases	12	SBP and fasting blood sugar	Pedometer and a physical activity diary with education materials and self-monitoring documents. Structured regular clinic sessions for review of physical activity diary and risk factors for ischemic heart disease. Monthly SMS text motivational messages.
Sarfo et al (2018) [52] Ghana	Primary care	Cluster RCT	55	≥18	Hypertension and stroke	9	BP control and medication adherence	Blue-toothed BP device and smartphone for self-monitoring and reporting BP measurements and medication intake. Tailored motivational text messages delivered based on the levels of adherence to the medication intake protocol.
Thuita et al 2020 Kenya [53]	Primary care	RCT	133	20–79	Metabolic syndrome	6	SBP and HbA1c	Integrated care with nutrition education and peer-to-peer support

*The control group received standard/usual care

For peer review only - http://bmjopen.bmj.com/site/about/guidelines.xhtml

Risk of bias in included studies

The risk of bias summary is presented in Figure 2. All the included studies were RCTs. Hence, all studies had low risk for randomization. Four of the 11 publications had a cluster design that ensured no contamination of control participants [43, 46, 49, 52]. The four cluster-randomized controlled trials accounted for clustering effects in their analysis so there was no unit of analysis errors [43, 46, 49, 52]. Six studies were appraised as unclear risk due to insufficient information about protection against contamination [44, 47, 48, 50, 51, 53]. One study was rated as unclear risk due to lack of sufficient information about allocation concealment. Although it was not feasible to blind the participants and study personnel due to the nature of the interventions, the absence of blinding did not affect the objective outcomes. Hence, all studies were graded as low risk for blinding. Under the reliability of primary outcomes, five studies were rated as high risk due to small sample sizes [48, 51-53] and lack of baseline measurements [43]. For selective reporting, all the studies were assessed as low risk, except one that lacked clarity and was assessed as unclear risk [50]. Four studies were rated high risk for other biases, mainly short follow-up duration (less than one year) [48, 50, 52, 53]. Overall, the certainties of the evidence for SBP, HbA1c, depression, medication adherence and quality of life were downgraded to moderate. This was due to the high risk of bias, imprecision and clinically insignificant effect sizes in the included studies [43-53].

[Figure 2 approximately here]

Components of the chronic care model

Table 2 presents the components of the chronic care model in the included studies. All studies had to include at least two components of the Wagner chronic care model to be defined as "integrated care" for the management of at least two chronic diseases. The number of chronic care elements ranged from two to five. One study included five of the six elements of the chronic care model [43]. Three studies included three elements [48, 51, 52] and six enclosed two elements [46, 49, 50, 53-55]. All the studies included delivery system design except one. The components of self-delivery included multi-disease screening and treatment, task shifting, structured follow-up, and collaborative care. Seven studies included self-management support such as home-based self-monitoring, lifestyle counselling, post-clinic patient follow-up and patient support groups [43, 44, 48, 51-53, 55]. Four studies included decision support components such as supplementary training of healthcare workers and support supervision [43, 46, 49, 50]. Three studies included clinical information systems [48, 51, 52]. The elements enclosed comprised hospital registries, patient diaries, report cards and electronic platforms for BP monitoring. Two studies included community resource elements to enhance health campaigns [43, 55]. One study included the healthcare organization element, mainly quality improvement strategies to enhance access to medication and treatment services [43].

For peer review only - http://bmjopen.bmj.com/site/about/guidelines.xhtml

Table 2: Elements of the chronic care model included in the studies

Author (year)	Self-management support	Delivery system design	Decision support	Clinical information system	Healthcare organization	Community resources
Havlir et al (2019) [43]	Enhanced lifestyle modification counselling in primary care centers	Point-of-care multi-disease screening for HIV, hypertension, and diabetes with structured follow-up and care linkages	Telephone and in-person oversight from a senior physician on the services provided by general physicians and nurses.		Quality improvement strategies such as guaranteed access to medication, flexible hours of operations, and reduced wait time at clinics	Multi-disease testing community health campaigns using community resource persons
Jackson et al (2021) [44]	Education on self-monitoring of BP, lifestyle modification, self-care and appropriate use of medicines	Structured pharmaceutical care including prescription review and follow-up				
Myers et al (2022) [45]		Task shifting and empowerment of community health workers to provide basic psychological interventions	Trained community health workers on basic psychological intervention including motivational interviewing and problem-solving therapy			
Okube et al (2022) [47]	Individualized health recommendations on CVD risk factors					Community-based health education on lifestyle modification
Owolabi et al 2019 [48]	Post-clinic follow-up phone texts and waiting room educational video	Enhanced follow-up visits and pre-appointment phone texts		Hospital registry and patient report card as part of medical chart		
Petersen et al (2021) [49]		Collaborative care model for patients with hypertension and comorbid depressive symptoms including doctors, nurses, clinical psychologist and lay counsellors	Supplementary training of primary care nurses and doctors on mental health and clinical communication skills			
Rabkin et al (2018) [50]	-	One stop shop for CVD risk factors screening and structured referrals among patients living with HIV	Training of HIV clinic nurses and doctors to conduct CVD risk factors screening during routine clinic appointments of patients			
Roos et al (2014) [51]	A pedometer and a physical activity diary with education materials and self-monitoring documents. Monthly SMS text motivational messages	Structured regular clinic sessions for review of physical activity diary and risk factors for ischemic heart disease		Patient diary for self-monitoring of risk factors for ischemic heart disease		
Sarfo et al (2018) [52]	Self-monitoring of BP using a blue-toothed device. Tailored motivational text messages delivered based on the levels of adherence to medication	Structured follow-up for BP measurements and medication adherence		Digital platform for tracking BP measurements and medication adherence		
Thuita et al 2020 [53]	Nutrition counselling and peer support group	Monthly follow up visits and structured regular clinic sessions to review patient progress. Facility-based patient support groups.				

For peer review only

Effectiveness of integrated models of care compared with standard care

Systolic blood pressure

Figure 3 presents the random effects meta-analysis of SBP. The pooled results of 6 studies [44, 47-49, 52, 53] involving 1,754 participants show that integrated versus standard care conferred a lower mean SBP (Mean Difference (MD) =-4.85mmHg, 95% CI (-7.37 to -2.34) with a small Hedges' g effect size (g=-0.25, (-0.39 to -0.11). However, the overall quality of evidence based on GRADE criteria was moderate. Moderate heterogeneity (I^2=39.57%) existed in the included studies (Figure 3). The results of the sensitivity analyses separating RCTs from cluster RCTs are consistent with those of the pooled analyses (Supplementary File 3). Integrated care reduced systolic blood pressure in RCTs by (MD=-5.71mmHg, 95% CI -9.32 to -2.09) and by (MD=-3.30mmHg, 95% CI -5.97 to –0.63) for the cluster RCTs. A Doi plot of the random effects meta-analysis of SBP for integrated versus standard care is shown in Supplementary File 4. The presence of publication bias is suggested by the asymmetrical visual inspection of the Doi plot, confirmed by LFK index=-2.37.

[Figure 3 approximately here]

We did not conduct meta-analyses for the four of the included studies in this review [43, 46, 50, 51], as they were too heterogeneous in terms of patient population, interventions, and outcome assessment. Hence, we conducted a structured synthesis of their results. Of the studies included in the structured synthesis of results, the effect of integrated versus standard care on BP were reported in three studies [43, 50, 51]. However, the quality of evidence based on GRADE criteria was moderate. The SEARCH trial conducted in Kenya and Uganda found that hypertension control was 22% higher among patients on integrated care versus standard care (relative prevalence=1.22; (95%CI 1.08 to 1.37)) [43]. In contrast, a study by Rabkin et al [50] conducted in Eswatini found no significant effect of integrated versus referred management of CVD risk factors on mean SBP. Another study by Roos et al (2014) also found no significant effect of education and a home-based pedometer walking program on SBP MD=2.50 mmHg, 95% CI (-2.78 to 7.78).

Fasting blood glucose and HbA1c

Two studies included in the structured synthesis of the results assessed the effect of integrated versus standard care on FBS and HbA1c [47, 50]. A study by Okube et al [47] found that integrated care conferred a significant ($p < 0.05$) reduction in FBS by -0.5mmol/L versus +0.08 mmol/L among patients on standard care. In a study by Rabkin et al [50], the mean of HbA1c was significantly reduced by -0.7% (95%CI: -1.3 to -0.1) and -1.4% (-2.5 to -0.2) in the integrated and standard care arms, respectively with no statistical

For peer review only - http://bmjopen.bmj.com/site/about/guidelines.xhtml

difference between arms 0.7% (95%CI -0.4 to 1.8).

Depression

The effect of integrated versus standard care on depression was reported in two studies. A study by Petersen et al [49] found no significant difference in the depression scores in patients on integrated versus standard care (adjusted risk difference = -0.04 (95%CI, -0.19 to 0.11)). By contrast, the results of the study by Myers et al [46] found significant differences in the depression scores in patients on integrated versus standard care (MD = 4.8(95%CI -7.2 to -2.4)).

Medication adherence and quality of life

Two studies assessed the effect of integrated care on hypertension medication adherence [45, 50]. One study reported the effect of integrated versus standard care HRQoL [45]. In a study by Rabkin et al [50], integrated care conferred a higher likelihood of hypertension medication adherence than standard care (relative risk=1.28 (95%CI 1.10 to 1.47)). A study by Jackson et al [45] found that integrated care led to significant improvements in hypertension medication adherence (Mean Difference in Difference (DiD)= 2.32; $p < 0.001$) and HRQoL (DiD= 6.5%, $P < 0.001$). Two studies assessed the effect of integrated care on hypertension medication adherence [45, 50]. One study reported the effect of integrated versus standard care on HRQoL [45]. In a study by Rabkin et al [50], integrated care conferred a higher likelihood of hypertension medication adherence than standard care (relative risk=1.28 (95%CI 1.10 to 1.47)). A study by Jackson et al [45] found that integrated care led to significant improvements in hypertension medication adherence (Mean Difference in Difference (DiD)= 2.32; $p < 0.001$) and HRQoL (DiD= 6.5%, $P < 0.001$).

.

Discussions

This paper presents the results of a systematic review of integrated care models for cardiometabolic multimorbidity and their effects on intermediate health outcomes in SSA. Our results show that, in comparison to usual care, integrated care featuring at least two elements of Wagner's chronic care model conferred moderate improvements in SBP among patients living with cardiometabolic multimorbidity. The overall quality of evidence was moderate. Results of the systematic review suggest that integrated care compared with usual care has mixed results with regards to HbA1c, depression, medication adherence and quality of life, with some studies showing a significant effect and some no effect.

The findings of the current review based on RCTs are broadly consistent with reviews on the effectiveness of the integrated chronic care model from previous observational studies [56, 57]. In contrast, one systematic review by Rohwer et al [19] on integrated care models for diabetes and hypertension in LMICs found no evidence of improved blood pressure or diabetes control. However, the evidence presented had

For peer review only - http://bmjopen.bmj.com/site/about/guidelines.xhtml

very low certainty and the included studies did not focus on care models for persons living with multimorbidity.

Our study used Wagner's chronic care model, a framework for improving the integrated management of chronic diseases through the implementation of its six core elements: community resources and policies, health care organization, self-management support, delivery system design, decision support and clinical information systems [11]. A review by Goh et al [56] found greater improvements in HbA1c for care models with more elements over a single element. In the same vein, another systematic review by Ellisen et al [56] reported variations in glycemic control with an increase in the number of elements of the chronic care model. However, most studies included in the current review had two to three CCM elements which could partly explain the mixed results with regard to the effect of care integration on intermediate health outcomes such as HbA1c, depression, medication adherence and quality of life, with some studies showing a significant effect and some no effect.

In the current review, delivery system design and self-management support were the most predominant components of the integrated chronic care models while healthcare organization and community resource elements were less common. Nevertheless, the structured synthesis of the results shows no significant differences in health outcomes by the elements of care integration since a majority of studies included two to three elements. Previous studies have shown that intervention intensity including length of implementation and frequency of monitoring, rather than the number of intervention components may have an impact on the direction and magnitude of the health outcomes [57, 58].

Overall, our results have three main implications. First, our findings have great public health significance given that a 5 mmHg reduction of SBP could reduce the risk of major cardiovascular events by about 13% [59]. Thus, incorporating integrated care into the implementation of Wagner's chronic care model may partly address the needs of people living with cardiometabolic multimorbidity. Second, this review provides crucial evidence on the applicability of chronic care models in SSA and unearths the components of chronic care models for people living with cardiometabolic multimorbidity. Lastly, most integrated care models sought to support self-management and delivery system design. However, a few included healthcare organizations and community resource elements. More interventions should seek to incorporate these elements to support integrated care.

For peer review only - http://bmjopen.bmj.com/site/about/guidelines.xhtml

Strengths and limitations

The current review has three main strengths. First, all the included studies were RCTs. Hence, the findings are more unbiased than previous reviews on integrated care in LMICs that are mostly based on observational study designs [19, 60-62]. Second, this study comprehensively extracted important constructs of Wagner's chronic care model, thus offering deeper insights into the effectiveness of its elements and delivering crucial evidence to researchers and health stakeholders for future improvement of integrated care models. The consideration of both communicable and non-communicable diseases in the review is also a strength due to the converging burdens of infectious and non-communicable diseases in SSA [7].

The findings of this review should be interpreted cautiously due to a few limitations. First, the majority of the included studies did not classify the components of chronic care models in the interventions. Although the three reviewers independently classified the type of chronic care model elements in the studies using a standard guide, there is potential for misclassification bias. Second, the varying numbers and types of chronic care model components added to the heterogeneity. Furthermore, the absolute effect attributable to a particular element of the chronic care model remains unknown, as most included studies used multi-component interventions. Third, it may not be possible to have a standard of care that is similar for all the studies included in this review. This resulted in a broad variety of usual care. Lastly, due to the substantial heterogeneity in the studies, in terms of multimorbidity, interventions, follow-up periods, and outcome assessment, only six of the ten studies were included in the meta-analysis, which may have a bearing on the power of the meta-analysis findings. The rest of the studies were included in the structured synthesis of the results.

Conclusions

We found that integrated care may lead to moderate improvements in SBP among patients living with cardiometabolic multimorbidity in SSA. This review highlights the paucity of research on care integration interventions for cardiometabolic multimorbidity in SSA since the selected RCTs were from only a few SSA countries. In addition, there were fewer studies with outcomes on health-related quality of life, mental health, and medication adherence. The small number of studies addressing healthcare organisation and community resource elements provide less certainty on the benefits associated with these components. More studies are needed on the implementation effectiveness of integrated care models and its impact on cardiometabolic multimorbidity. The relative effectiveness of the different elements of integrated chronic care models and the cost-effectiveness of these models for the management of cardiometabolic multimorbidity should be explored. Other elements that are not well explored such as healthcare organisation and community resource elements should be investigated in future research.

For peer review only - http://bmjopen.bmj.com/site/about/guidelines.xhtml

For peer review only

Future studies investigating the effectiveness of integrated care should classify the elements in the interventions and standardize the description of each element.

Acknowledgements: Not applicable.

Author contributions: PO conceptualized the study, reviewed the literature, and analysed the data. All authors contributed to the development and review of the protocol. EW, MN, and DM screened titles and abstracts and participated in full-text screening; PO helped to resolve discrepancies. EW, MN, and DM extracted data and assessed the risk of bias. PO assessed the certainty of evidence with input from all the authors. PO, JO and EW performed the meta-analyses. CA, HW and GA provided overall methodological guidance. JO and PK critically read and revised the first draft of the manuscript. PO, EW, PK and JO responded to reviewers' comments and made subsequent revisions. All authors have approved the final version of the manuscript.

Funding: This systematic review was supported by funding from the Joep Lange Institute and in part by the Dutch Ministry of Foreign Affairs under the Joep Lange Chairs and Fellows Program. JO and EW are funded by Wellcome Trust [218462/Z/19/Z] doctoral training grant to the University of Sheffield. The funders had no role in the design of the study and collection, analysis, and interpretation of data and in writing the manuscript.

Disclaimer: The funder did not have any role in the review process.

Competing interests: None declared.

Patient consent for publication: Not required.

Ethics approval: This systematic review does not involve human participants. All data included are in the public domain and ethics approval was thus not sought.

Data availability statement: All data relevant to the study are included in the article or uploaded as online supplemental information.

For peer review only - http://bmjopen.bmj.com/site/about/guidelines.xhtml

References

1. Roth, G.A., et al., *Global burden of cardiovascular diseases and risk factors, 1990–2019: update from the GBD 2019 study.* Journal of the American College of Cardiology, 2020. **76**(25): p. 2982-3021.
2. Violan, C., et al., *Prevalence, determinants and patterns of multimorbidity in primary care: a systematic review of observational studies.* PloS one, 2014. **9**(7).
3. Arenillas, J.F., M.a.A. Moro, and A. Dávalos, *The metabolic syndrome and stroke: potential treatment approaches.* Stroke, 2007. **38**(7): p. 2196-2203.
4. Mannino, D.M., et al., *Prevalence and outcomes of diabetes, hypertension and cardiovascular disease in COPD.* European Respiratory Journal, 2008. **32**(4): p. 962-969.
5. Metra, M., et al., *Cardiovascular and noncardiovascular comorbidities in patients with chronic heart failure.* Journal of Cardiovascular Medicine, 2011. **12**(2): p. 76-84.
6. Scott, K.M., *Depression, anxiety and incident cardiometabolic diseases.* Current opinion in psychiatry, 2014. **27**(4): p. 289-293.
7. Coates, M.M., et al., *Burden of disease among the world's poorest billion people: An expert-informed secondary analysis of Global Burden of Disease estimates.* Plos one, 2021. **16**(8): p. e0253073.
8. Guthrie, B., et al., *Adapting clinical guidelines to take account of multimorbidity.* Bmj, 2012. **345**: p. e6341.
9. Nolte, E. and M. McKee, *Caring for people with chronic conditions: a health system perspective*. 2008: McGraw-Hill Education (UK).
10. Boult, C., et al., *Early effects of "Guided Care" on the quality of health care for multimorbid older persons: a cluster-randomized controlled trial.* J Gerontol A Biol Sci Med Sci, 2008. **63**(3): p. 321-327.
11. Wagner, E.H., *Chronic disease management: what will it take to improve care for chronic illness?* Effective clinical practice, 1998. **1**(1).
12. World Health Organization, *Towards people-centred health systems: An innovative approach for better health outcomes*, in *World Health Organization Regional Office for Europe, Division of Health Systems and Public Health. Geneva: Switzerland: WHO Press*. 2013.
13. Kamkuemah, M., B. Gausi, and T. Oni, *Missed opportunities for NCD multimorbidity prevention in adolescents and youth living with HIV in urban South Africa.* BMC Public Health, 2020. **20**(1): p. 821.
14. Barr, A.L., et al., *The need for an integrated approach for chronic disease research and care in Africa.* Global health, epidemiology and genomics, 2016. **1**.
15. Singer, S.J., et al., *Defining and measuring integrated patient care: promoting the next frontier in health care delivery.* Medical Care Research and Review, 2011. **68**(1): p. 112-127.
16. Battersby, M.W., *Health reform through coordinated care: SA HealthPlus.* Bmj, 2005. **330**(7492): p. 662-665.
17. Geldsetzer, P., K. Ortblad, and T. Bärnighausen, *The efficiency of chronic disease care in sub-Saharan Africa.* BMC medicine, 2016. **14**(1): p. 127.
18. Harries, A.D., et al., *Adapting the DOTS framework for tuberculosis control to the management of non-communicable diseases in sub-Saharan Africa.* PLoS medicine, 2008. **5**(6).
19. Rohwer, A., et al., *Effects of integrated models of care for diabetes and hypertension in low-income and middle-income countries: a systematic review and meta-analysis.* BMJ open, 2021. **11**(7): p. e043705.
20. Shamseer, L., et al., *Preferred reporting items for systematic review and meta-analysis protocols (PRISMA-P) 2015: elaboration and explanation.* Bmj, 2015. **349**.
21. Naghavi, M., et al., *Global, regional, and national age-sex specific mortality for 264 causes of death, 1980–2016: a systematic analysis for the Global Burden of Disease Study 2016.* The

For peer review only

For peer review only - http://bmjopen.bmj.com/site/about/guidelines.xhtml

lancet, 2017. **390**(10100): p. 1151-1210.
22. Busetto, L., et al., *Intervention types and outcomes of integrated care for diabetes mellitus type 2: a systematic review.* Journal of evaluation in clinical practice, 2016. **22**(3): p. 299-310.
23. Desmedt, M., et al., *Economic impact of integrated care models for patients with chronic diseases: a systematic review.* Value in Health, 2016. **19**(6): p. 892-902.
24. Drewes, H.W., et al., *The effectiveness of chronic care management for heart failure: meta-regression analyses to explain the heterogeneity in outcomes.* Health services research, 2012. **47**(5): p. 1926-1959.
25. Elissen, A.M., et al., *Meta-analysis of the effectiveness of chronic care management for diabetes: investigating heterogeneity in outcomes.* Journal of Evaluation in Clinical Practice, 2013. **19**(5): p. 753-762.
26. Meeuwissen, J.A., et al., *Meta-analysis and meta-regression analyses explaining heterogeneity in outcomes of chronic care management for depression: implications for person-centered mental healthcare.* International Journal of Person Centered Medicine, 2012. **2**(4): p. 716-758.
27. Effective Practice and Organisation of Care (EPOC), *What study designs should be included in an EPOC review? EPOC Resources for review authors*. 2013, Norwegian Knowledge Centre for the Health Services: Oslo.
28. Wagner, E.H., et al., *Improving chronic illness care: translating evidence into action.* Health affairs, 2001. **20**(6): p. 64-78.
29. Practice, C.E. and O.o. Care, *Suggested risk of bias criteria for EPOC reviews*, in *EPOC Resources for review authors*. 2017.
30. Hedges, L.V. and I. Olkin, *Statistical methods for meta-analysis*. 2014: Academic press.
31. Deeks, J.J., et al., *Analysing data and undertaking meta-analyses.* Cochrane handbook for systematic reviews of interventions, 2019: p. 241-284.
32. Hoaglin, D.C., *Misunderstandings about Q and 'Cochran's Q test'in meta-analysis.* Statistics in medicine, 2016. **35**(4): p. 485-495.
33. Higgins, J.P., et al., *Measuring inconsistency in meta-analyses.* Bmj, 2003. **327**(7414): p. 557-560.
34. Furuya-Kanamori, L., J.J. Barendregt, and S.A. Doi, *A new improved graphical and quantitative method for detecting bias in meta-analysis.* JBI Evidence Implementation, 2018. **16**(4): p. 195-203.
35. Xu, C. and L. Furuya-Kanamori, *DOIPLOT: Stata module for visualization of asymmetry and heterogeneity in meta-analysis.* 2021.
36. Higgins, J.P., S. Eldridge, and T. Li, *Including variants on randomized trials.* Cochrane handbook for systematic reviews of interventions, 2019: p. 569-593.
37. Bolland, M.J., A. Avenell, and A. Grey, *Analysis of cluster randomised trials as if they were individually randomised.* The Lancet Diabetes & Endocrinology, 2023. **11**(2): p. 75.
38. Guyatt, G., et al., *GRADE guidelines: 1. Introduction—GRADE evidence profiles and summary of findings tables.* Journal of clinical epidemiology, 2011. **64**(4): p. 383-394.
39. Santesso, N., et al., *GRADE guidelines 26: informative statements to communicate the findings of systematic reviews of interventions.* Journal of clinical epidemiology, 2020. **119**: p. 126-135.
40. Rahimi, K., et al., *Pharmacological blood pressure lowering for primary and secondary prevention of cardiovascular disease across different levels of blood pressure: an individual participant-level data meta-analysis.* The Lancet, 2021. **397**(10285): p. 1625-1636.
41. Kaiafa, G., et al., *Is HbA1c an ideal biomarker of well-controlled diabetes?* Postgraduate Medical Journal, 2021. **97**(1148): p. 380-383.
42. Puhan, M.A., et al., *A GRADE Working Group approach for rating the quality of treatment effect estimates from network meta-analysis.* Bmj, 2014. **349**.

For peer review only

For peer review only - http://bmjopen.bmj.com/site/about/guidelines.xhtml

43. Havlir, D.V., et al., *HIV testing and treatment with the use of a community health approach in rural Africa.* New England Journal of Medicine, 2019. **381**(3): p. 219-229.
44. Jackson, I.L. and C.V. Ukwe, *Clinical outcomes of pharmaceutical care intervention in HIV positive patients with hypertension: a randomized controlled study.* Journal of Clinical Pharmacy and Therapeutics, 2021. **46**(4): p. 1083-1094.
45. Jackson, I.L. and C.V. Ukwe, *Effects of pharmaceutical care interventions on humanistic outcomes in hypertensive people living with HIV: results of a randomized controlled trial.* International Journal of Pharmacy Practice, 2022. **30**(3): p. 261-267.
46. Myers, B., et al., *Comparing dedicated and designated approaches to integrating task-shared psychological interventions into chronic disease care in South Africa: a three-arm, cluster randomised, multicentre, open-label trial.* The Lancet, 2022. **400**(10360): p. 1321-1333.
47. Okube, O.T., S. Kimani, and W. Mirie, *Community-based lifestyle intervention improves metabolic syndrome and related markers among Kenyan adults.* Journal of Diabetes & Metabolic Disorders, 2022. **21**(1): p. 607-621.
48. Owolabi, M.O., et al., *Randomized trial of an intervention to improve blood pressure control in stroke survivors.* Circulation: Cardiovascular Quality and Outcomes, 2019. **12**(12): p. e005904.
49. Petersen, I., et al., *Effectiveness of a task-sharing collaborative care model for identification and management of depressive symptoms in patients with hypertension attending public sector primary care clinics in South Africa: pragmatic parallel cluster randomised controlled trial.* Journal of Affective Disorders, 2021. **282**: p. 112-121.
50. Rabkin, M., et al., *Integrated vs. referred management of CVD risk factors for HIV positive patients on antiretroviral therapy in Swaziland.* 2018.
51. Roos, R., et al., *Effects of an education and home-based pedometer walking program on ischemic heart disease risk factors in people infected with HIV: a randomized trial.* JAIDS Journal of Acquired Immune Deficiency Syndromes, 2014. **67**(3): p. 268-276.
52. Sarfo, F.S., et al., *Phone-based intervention for blood pressure control among Ghanaian stroke survivors: a pilot randomized controlled trial.* International Journal of Stroke, 2019. **14**(6): p. 630-638.
53. Thuita, A.W., et al., *Effect of a nutrition education programme on the metabolic syndrome in type 2 diabetes mellitus patients at a level 5 Hospital in Kenya:"a randomized controlled trial".* BMC nutrition, 2020. **6**: p. 1-14.
54. Jackson, I.L. and C.V. Ukwe, *Effects of pharmaceutical care interventions on humanistic outcomes in hypertensive people living with HIV: results of a randomized controlled trial.* International Journal of Pharmacy Practice, 2022.
55. Okube, O.T., S. Kimani, and W. Mirie, *Community-based lifestyle intervention improves metabolic syndrome and related markers among Kenyan adults.* Journal of Diabetes & Metabolic Disorders, 2022: p. 1-15.
56. Goh, L.H., et al., *Effectiveness of the chronic care model for adults with type 2 diabetes in primary care: a systematic review and meta-analysis.* Systematic Reviews, 2022. **11**(1): p. 1-23.
57. Si, D., R. Bailie, and T. Weeramanthri, *Effectiveness of chronic care model-oriented interventions to improve quality of diabetes care: a systematic review.* Primary Health Care Research & Development, 2008. **9**(1): p. 25-40.
58. Weingarten, S.R., et al., *Interventions used in disease management programmes for patients with chronic illnesswhich ones work? Meta-analysis of published reports.* Bmj, 2002. **325**(7370): p. 925.
59. Reboldi, G., et al., *Effects of intensive blood pressure reduction on myocardial infarction and stroke in diabetes: a meta-analysis in 73 913 patients.* Journal of hypertension, 2011. **29**(7): p. 1253-1269.

For peer review only - http://bmjopen.bmj.com/site/about/guidelines.xhtml

60. Joshi, R., et al., *Task shifting for non-communicable disease management in low and middle income countries–a systematic review.* PloS one, 2014. **9**(8): p. e103754.
61. Kemp, C.G., et al., *Implementation science for integration of HIV and non-communicable disease services in sub-Saharan Africa: a systematic review.* Aids, 2018. **32**: p. S93-S105.
62. Thornicroft, G., et al., *Integrated care for people with long-term mental and physical health conditions in low-income and middle-income countries.* The Lancet Psychiatry, 2019. **6**(2): p. 174-186.

List of Figures

Figure 1: PRISMA flow diagram of the article selection process

Figure 2: Risk of bias in included studies

Figure 3: Forest plot of the random effects meta-analysis of the effect of integrated versus standard care on SBP for people with cardiometabolic multimorbidity in Sub-Saharan Africa

For peer review only

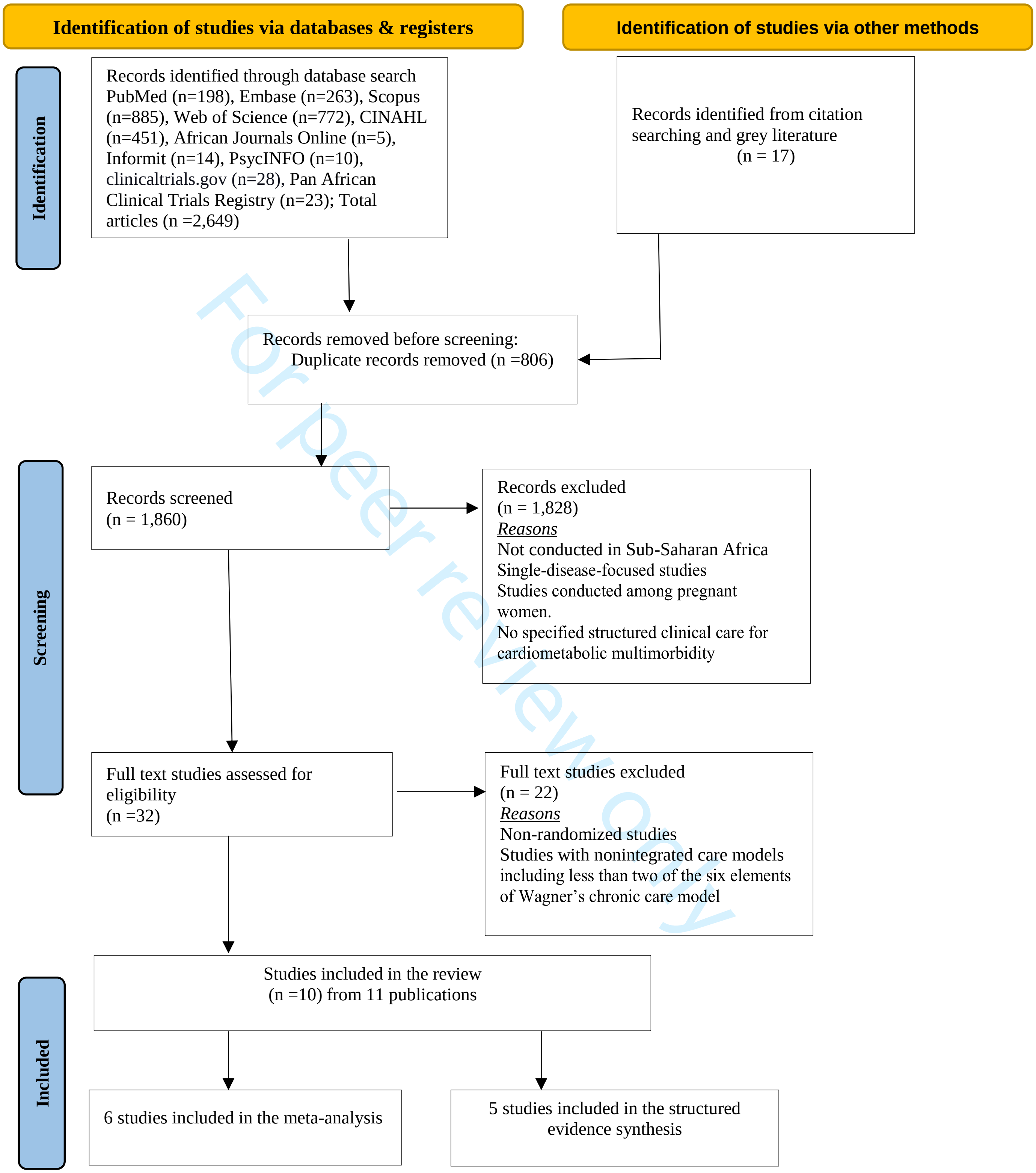

For peer review only

	Randomization process	Allocation concealment (selection bias)	Blinding (Performance and detection bias)	Selective reporting bias	Other bias	Protection against contamination	Reliable primary outcomes	Baseline measurements	Overall
Havlir et al (2019)	+	+	+	+	+	+	?	?	?
Jackson et al (2021)	+	+	+	+	+	?	+	+	+
Myers et al (2022)	+	+	+	+	+	+	+	+	+
Okube et al (2022)	+	+	+	+	+	?	+	+	+
Owolabi et al 2019	+	+	+	+	-	?	-	+	?
Petersen et al (2021)	+	+	+	+	+	+	+	+	+
Rabkin et al (2018)	+	?	+	+	-	?	+	+	?
Roos et al (2014)	+	+	+	+	+	?	-	+	?
Sarfo et al (2018)	+	+	+	?	-	+	-	+	?
Thuita et al (2020)	+	+	+	+	-	?	-	+	?

Low risk +
Some concerns ?
High risk -

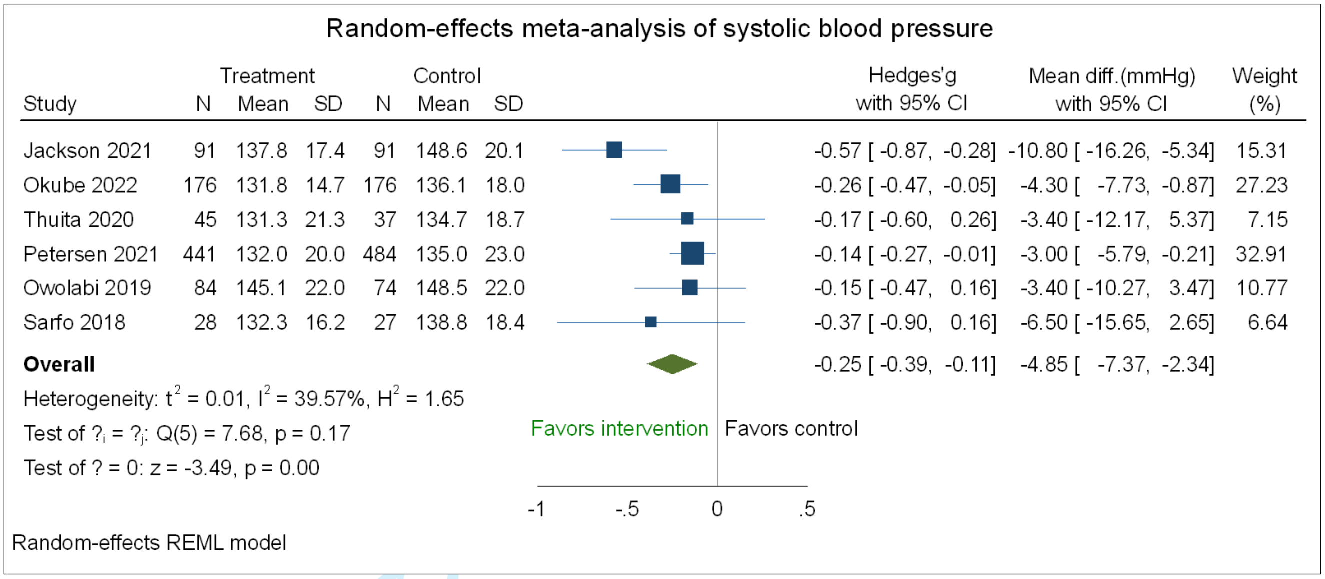

For peer review only

List of online supplementary files

Online supplementary file 1: Classification of interventions

Intervention component	Feature
Self-management support	• Self-care interventions based on technological aid<br>• Self-help groups<br>• Family-oriented supports<br>• Motivational support<br>• Behaviour therapy
Delivery system design	• Practice team functioning<br>• Patient care planning and follow-up<br>• Coordination between primary care and specialist services
Decision support	• Practice clinical guidelines<br>• Provider education<br>• Involvement of specialists in improving primary care
Clinical information system	• Disease registry<br>• Reminders to providers<br>• Feedback to providers
Healthcare organization	• Organizational goals and resources for chronic disease management<br>• Quality improvement strategies<br>• Incentives
Community linkages	• Linking patients to outside resources<br>• Activities with community-based organizations<br>• Professionals working out in the community

Supplementary file 2: Search strategies for electronic databases

1. **PubMED (Medline) Search strategy**

#1 ("elevated blood pressure"[MeSH Terms] OR "high blood pressure"[MeSH Terms] OR "raised blood pressure"[MeSH Terms] OR "diastolic blood pressure"[MeSH Terms] OR "systolic blood pressure"[MeSH Terms] OR "arterial blood pressure"[MeSH Terms] OR "uncontrolled blood pressure"[MeSH Terms] OR "uncontrolled hypertension"[All Fields] OR "elevated blood pressure"[All Fields] OR "high blood pressure"[All Fields] OR "raised blood pressure"[All Fields] OR "diastolic blood pressure"[All Fields] OR "systolic blood pressure"[All Fields] OR "arterial blood pressure"[All Fields] OR "uncontrolled blood pressure"[All Fields] OR "uncontrolled hypertension"[All Fields])

#2 ("diabetes mellitus"[MeSH Terms] OR "diabetes mellitus, type 2"[MeSH Terms] OR "diabetes type 2"[MeSH Terms] OR "T2DM"[MeSH Terms] OR "diabetes type II"[MeSH Terms] OR "diabetes"[MeSH Terms] OR "glucose intolerance"[MeSH Terms] OR "insulin resistance"[MeSH Terms] OR "hyperglycemia"[MeSH Terms] OR "hyperglycaemia"[MeSH Terms] OR "hypoglycaemia"[MeSH Terms] OR "hypoglycaemia"[MeSH Terms] OR "high blood sugar"[MeSH Terms] OR "elevated blood sugar"[MeSH Terms] OR "raised blood sugar"[MeSH Terms] OR "high blood glucose"[MeSH Terms] OR "elevated blood glucose"[MeSH Terms] OR "raised blood glucose"[MeSH Terms] OR "diabetes mellitus"[All Fields] OR "diabetes mellitus, type 2"[All Fields] OR "diabetes type 2"[All Fields] OR "T2DM"[All Fields] OR "diabetes type II"[All Fields] OR "diabetes"[All Fields] OR "glucose intolerance"[All Fields] OR "insulin resistance"[All Fields] OR "hyperglycemia"[All Fields] OR "hyperglycaemia"[All Fields] OR "hypoglycemia"[All Fields] OR "hypoglycaemia"[All Fields] OR "high blood sugar"[All Fields] OR "elevated blood sugar"[All Fields] OR "raised blood sugar"[All Fields] OR "high blood glucose"[All Fields] OR "elevated blood glucose"[All Fields] OR "raised blood glucose"[All Fields])

For peer review only - http://bmjopen.bmj.com/site/about/guidelines.xhtml

#3 ("stroke"[MeSH Terms] OR "transient ischemic attack"[MeSH Terms] OR "ischemic attack"[MeSH Terms] OR "angina"[MeSH Terms] OR "angina pectoris"[MeSH Terms] OR "heart attack"[MeSH Terms] OR "ischemic heart diseases "[MeSH Terms] OR "transient ischaemic attack"[MeSH Terms] OR "coronary heart disease"[MeSH Terms] OR "coronary disease"[MeSH Terms] OR "heart failure"[MeSH Terms] OR "peripheral vascular disease"[MeSH Terms] OR "Peripheral Vascular diseases"[MeSH Terms] OR "atrial fibrillation"[MeSH Terms] OR "cardiovascular disease"[MeSH Terms] OR "heart disease"[MeSH Terms] OR "stroke"[All Fields] OR "transient ischemic attack"[All Fields] OR "ischemic attack"[All Fields] OR "angina"[All Fields] OR "angina pectoris"[All Fields] OR "heart attack"[All Fields] OR "ischemic heart diseases "[All Fields] OR "transient ischaemic attack"[All Fields] OR "coronary heart disease"[All Fields] OR "coronary disease"[All Fields] OR "heart failure"[All Fields] OR "peripheral vascular disease"[All Fields] OR "Peripheral Vascular diseases"[All Fields] OR "atrial fibrillation"[All Fields] OR "cardiovascular disease"[All Fields] OR "heart disease"[All Fields])

#4 ("Hypercholesterolemia"[MeSH Terms] OR "Dyslipidemias"[MeSH Terms] OR "blood lipid"[MeSH Terms] OR "Cholesterol"[MeSH Terms] OR "high cholesterol"[MeSH Terms] OR "elevated cholesterol"[MeSH Terms] OR "raised cholesterol"[MeSH Terms] OR "low density lipoprotein "[MeSH Terms] OR "high density lipoprotein"[MeSH Terms] OR "Dyslipidemia"[MeSH Terms] OR "Dyslipidaemia"[MeSH Terms] OR "Hypercholesterolemia"[MeSH Terms] OR "hypercholesterolaemia "[MeSH Terms] OR "hypercholesterolimia "[MeSH Terms] OR "Hypertriglyceridemia"[MeSH Terms] OR "Hypertriglyceridemia"[MeSH Terms] OR "Hypertriglyceridaemia"[MeSH Terms] OR "Hyperlipidemia"[MeSH Terms] OR "Hyperlipidemias"[MeSH Terms] OR "Hyperlipidaemia"[MeSH Terms] OR "LDL"[MeSH Terms] OR "HDL"[MeSH Terms] OR "Hypercholesterolemia"[All Fields] OR "Dyslipidemias"[All Fields] OR "blood lipid"[All Fields] OR "Cholesterol"[All Fields] OR "high cholesterol"[All Fields] OR "elevated cholesterol"[All Fields] OR "raised cholesterol"[All Fields] OR "low density lipoprotein "[All Fields] OR "high density lipoprotein"[All Fields] OR "Dyslipidemia"[All Fields] OR "Dyslipidaemia"[All Fields] OR "Hypercholesterolemia"[All Fields] OR "hypercholesterolaemia "[All Fields] OR "hypercholesterolimia "[All Fields] OR "Hypertriglyceridemia"[All Fields] OR "Hypertriglyceridemia"[All Fields] OR "Hypertriglyceridaemia"[All Fields] OR "Hyperlipidemia"[All Fields] OR "Hyperlipidemias"[All Fields] OR "Hyperlipidaemia"[All Fields] OR "LDL"[All Fields] OR "HDL"[All Fields])

#5 ("Comorbid"[All Fields] OR "co-morbid"[All Fields] OR "co-morbidity"[All Fields] OR "Multimorbidity"[All Fields] OR "multi-morbid"[All Fields] OR "multi morbidity"[All Fields] OR "Multimorbidity"[All Fields] OR "Comorbidity"[All Fields] OR "multi-disease"[All Fields] OR "Multidisease"[All Fields] OR multi disease[All Fields] OR "multiple condition"[All Fields] OR "multi-condition"[All Fields] OR "multi condition"[All Fields] OR "multiple illness"[All Fields] OR "multi-illness"[All Fields] OR "multi illness"[All Fields] OR "multiple syndrome"[All Fields] OR "multi-syndrome"[All Fields] OR "multi syndrome"[All Fields] OR "concurrent condition"[All Fields] OR "concurrent illness"[All Fields] OR "concurrent disease"[All Fields] OR "co-existing disease"[All Fields] OR "coexisting disease"[All Fields] OR "co-existing illness"[All Fields] OR "coexisting illness"[All Fields] OR "co-existing syndrome"[All Fields] OR "coexisting syndrome"[All Fields] OR "co-existing condition"[All Fields] OR "coexisting condition"[All Fields] OR "co-occurring disease"[All Fields] OR "co-occuring disease"[All Fields] OR "co-occuring disease"[All Fields] OR "co-occurring illness"[All Fields] OR "co occurring illness"[All Fields] OR "cooccurring illness"[All Fields] OR "co-occurring syndrome"[All Fields] OR "co occurring syndrome"[All Fields] OR "cooccurring syndrome"[All Fields] OR "co-occurring condition"[All Fields] OR "co occurring condition"[All Fields] OR "cooccurring condition"[All Fields])

#6 ("Wagner model"[All Fields] OR "Wagner* model*"[All Fields] OR "Wagner* chronic care model*"[All Fields] OR "Wagner chronic care model"[All Fields] OR "chronic care model*"[All Fields] OR "model "[All Fields] OR "collaborative care"[All Fields] OR "chronic care framework"[All Fields] OR "chronic disease care"[All Fields] OR "chronic illness care"[All Fields] OR care model[All Fields] OR "model care"[All Fields] OR "Wagner* model*"[All Fields] OR "Wagner* chronic care model*"[All Fields] OR "Wagner chronic care model"[All Fields] OR "theory"[All Fields] OR "concept"[All Fields] OR "framework"[All Fields] OR "model"[All Fields] OR "programme"[All Fields] OR "approach"[All Fields] OR "clinical pathway"[All Fields] OR "care pathway"[All Fields] OR "critical path"[All Fields] OR "vertical integration"[All Fields] OR "virtual integration"[All Fields] OR "physician system integration"[All Fields] OR "provider system integration"[All Fields] OR "functional

For peer review only - http://bmjopen.bmj.com/site/about/guidelines.xhtml

integration"[All Fields] OR "horizontal integration"[All Fields] OR "clinical integration"[All Fields] OR "case management"[All Fields] OR "delivery of health care, integrated"[All Fields] OR "disease management"[All Fields] OR "patient care management"[All Fields] OR "patient-centred care"[All Fields] OR "accountable care organisations"[All Fields] OR "continuity of patient care"[All Fields] OR "case management"[All Fields] OR "comprehensive health care"[All Fields] OR "delivery of health care, integrated"[All Fields] OR "managed care programmes"[All Fields] OR "patient-centred care"[All Fields] OR "care delivery"[All Fields] OR "integrated care"[All Fields] OR "comprehensive care"[All Fields] OR "care coordination"[All Fields] OR "managed care"[All Fields] OR "accountable care "[All Fields] OR "accountable care organisations"[All Fields] OR "accountable care organisation"[All Fields] OR "collaborative care"[All Fields] OR "disease management"[All Fields] OR "case-management"[All Fields] OR "case management"[All Fields] OR "shared care"[All Fields] OR "accountable care"[All Fields] OR "patient-centred"[All Fields] OR "patient centred"[All Fields] OR "person-centred"[All Fields] OR "person centred"[All Fields] OR "multidisciplinary care"[All Fields] OR "interdisciplinary care"[All Fields] OR "inter-disciplinary care"[All Fields] OR "cross disciplinary care"[All Fields] OR "cross-disciplinary care"[All Fields] OR "multiple interventions"[All Fields] OR "care chain"[All Fields] OR "care chains"[All Fields] OR "care continuity"[All Fields] OR "care continuation"[All Fields] OR "care transition"[All Fields] OR "care transitions"[All Fields] OR "chain of care"[All Fields] OR "chains of care"[All Fields] OR "continuity of care"[All Fields] OR "cross sectoral care"[All Fields] OR "delivery of health care integrated"[All Fields] OR "integrated medicine"[All Fields] OR "integrated social network"[All Fields] OR "integrated social networks"[All Fields] OR "integration of care"[All Fields] OR "intersectoral care"[All Fields] OR "intrasectoral care"[All Fields] OR "linked care"[All Fields] OR "management model"[All Fields] OR "patient care management"[All Fields] OR "seamless care"[All Fields] OR "service network"[All Fields] OR "service networks"[All Fields] OR "transition of care"[All Fields] OR "transitional care"[All Fields] OR "transmural care"[All Fields] OR "whole system thinking"[All Fields] OR "holistic care"[All Fields])

#7 ("Angola "[All Fields] OR "Benin "[All Fields] OR "Botswana "[All Fields] OR "Burkina Faso"[All Fields] OR "Upper Volta"[All Fields] OR "Burundi "[All Fields] OR "Cameroon "[All Fields] OR "Cameroons "[All Fields] OR "Cape Verde"[All Fields] OR "Central African Republic"[All Fields] OR Chad[All Fields] OR "Comoros "[All Fields] OR "Comoro Islands"[All Fields] OR "Comores"[All Fields] OR "Mayotte "[All Fields] OR "Congo "[All Fields] OR "Zaire "[All Fields] OR "Cote d'Ivoire"[All Fields] OR "Ivory Coast"[All Fields] OR "Democratic Republic of the Congo"[All Fields] OR "Djibouti "[All Fields] OR "French Somaliland"[All Fields] OR "Eritrea "[All Fields] OR "Ethiopia "[All Fields] OR "Gabon "[All Fields] OR "Gabonese Republic"[All Fields] OR "Gambia "[All Fields] OR "Ghana "[All Fields] OR "Gold Coast"[All Fields] OR "Guinea "[All Fields] OR "Kenya "[All Fields] OR "Lesotho"[All Fields] OR "Basutoland"[All Fields] OR "Liberia "[All Fields] OR "Madagascar "[All Fields] OR "Malagasy Republic "[All Fields] OR "Malawi "[All Fields] OR "Nyasaland "[All Fields] OR "Mali "[All Fields] OR "Mauritania "[All Fields] OR "Mauritius "[All Fields] OR "Mozambique"[All Fields] OR "Namibia"[All Fields] OR "Niger"[All Fields] OR "Nigeria"[All Fields] OR "Rwanda"[All Fields] OR "Sao Tome"[All Fields] OR "Seychelles"[All Fields] OR "Senegal "[All Fields] OR "Sierra Leone"[All Fields] OR "Somalia "[All Fields] OR "South Africa"[All Fields] OR "Sudan "[All Fields] OR "Eswatini"[All Fields] OR "Tanzania"[All Fields] OR "Togo "[All Fields] OR "Togolese Republic"[All Fields] OR "Uganda"[All Fields] OR "Zambia"[All Fields] OR "Zimbabwe "[All Fields] OR "Rhodesia"[All Fields] OR "Sub-Saharan Africa"[All Fields] OR "Africa South of the Sahara"[All Fields])

#8 ((#1 OR #2 OR #3 OR #4 OR #5) AND #6 AND #7) AND Restricted (1999-2022, RCTs)

2. Web of Science Search Strategy

#1 elevated blood pressure OR high blood pressure OR raised blood pressure OR diastolic blood pressure OR systolic blood pressure OR arterial blood pressure OR uncontrolled blood pressure OR uncontrolled hypertension OR elevated blood pressure OR high blood pressure OR raised blood pressure OR diastolic blood pressure OR systolic blood pressure OR arterial blood pressure OR uncontrolled blood pressure OR uncontrolled hypertension

#2 diabetes mellitus OR diabetes mellitus, type 2 OR diabetes type 2 OR T2DM OR diabetes type II OR diabetes OR glucose intolerance OR insulin resistance OR hyperglycemia OR hyperglycaemia OR hypoglycaemia

For peer review only - http://bmjopen.bmj.com/site/about/guidelines.xhtml

OR hypoglycaemia OR high blood sugar OR elevated blood sugar OR raised blood sugar OR high blood glucose OR elevated blood glucose OR raised blood glucose OR diabetes mellitus OR diabetes mellitus, type 2 OR diabetes type 2 OR T2DM OR diabetes type II OR diabetes OR glucose intolerance OR insulin resistance OR hyperglycemia OR hyperglycaemia OR hypoglycemia OR hypoglycaemia OR high blood sugar OR elevated blood sugar OR raised blood sugar OR high blood glucose OR elevated blood glucose OR raised blood glucose

#3 stroke OR transient ischemic attack OR ischemic attack OR angina OR angina pectoris OR heart attack OR ischemic heart diseases OR transient ischaemic attack OR coronary heart disease OR coronary disease OR heart failure OR peripheral vascular disease OR Peripheral Vascular diseases OR atrial fibrillation OR cardiovascular disease OR heart disease OR stroke OR transient ischemic attack OR ischemic attack OR angina OR angina pectoris OR heart attack OR ischemic heart diseases OR transient ischaemic attack OR coronary heart disease OR coronary disease OR heart failure OR peripheral vascular disease OR Peripheral Vascular diseases OR atrial fibrillation OR cardiovascular disease OR heart disease

#4 Hypercholesterolemia OR Dyslipidemias OR blood lipid OR Cholesterol OR high cholesterol OR elevated cholesterol OR raised cholesterol OR low density lipoprotein OR high density lipoprotein OR Dyslipidemia OR Dyslipidaemia OR Hypercholesterolemia OR hypercholesterolaemia OR hypercholesterolimia OR Hypertriglyceridemia OR Hypertriglyceridemia OR Hypertriglyceridaemia OR Hyperlipidemia OR Hyperlipidemias OR Hyperlipidaemia OR LDL OR HDL OR Hypercholesterolemia OR Dyslipidemias OR blood lipid OR Cholesterol OR high cholesterol OR elevated cholesterol OR raised cholesterol OR low density lipoprotein OR high density lipoprotein OR Dyslipidemia OR Dyslipidaemia OR Hypercholesterolemia OR hypercholesterolaemia OR hypercholesterolimia OR Hypertriglyceridemia OR Hypertriglyceridemia OR Hypertriglyceridaemia OR Hyperlipidemia OR Hyperlipidemias OR Hyperlipidaemia OR LDL OR HDL

#5 Comorbid OR co-morbid OR co-morbidity OR Multimorbidity OR multi-morbid OR multi morbidity OR Multimorbidity OR Comorbidity OR multi-disease OR Multidisease OR multi disease OR multiple condition OR multi-condition OR multi condition OR multiple illness OR multi-illness OR multi illness OR multiple syndrome OR multi-syndrome OR multi syndrome OR concurrent condition OR concurrent illness OR concurrent disease OR co-existing disease OR coexisting disease OR co-existing illness OR coexisting illness OR co-existing syndrome OR coexisting syndrome OR co-existing condition OR coexisting condition OR co-occurring disease OR co-occuring disease OR co-occuring disease OR co-occurring illness OR co occurring illness OR cooccurring illness OR co-occurring syndrome OR co occurring syndrome OR cooccurring syndrome OR co-occurring condition OR co occurring condition OR cooccurring condition

#6 Wagner model OR Wagner* model* OR Wagner* chronic care model* OR Wagner chronic care model OR chronic care model* OR model OR collaborative care OR chronic care framework OR chronic disease care OR chronic illness care OR care model OR model care OR Wagner* model* OR Wagner* chronic care model* OR Wagner chronic care model OR theory OR concept OR framework OR model OR programme OR approach OR clinical pathway OR care pathway OR critical path OR vertical integration OR virtual integration OR physician system integration OR provider system integration OR functional integration OR horizontal integration OR clinical integration OR case management OR delivery of health care, integrated OR disease management OR patient care management OR patient-centred care OR accountable care organisations OR continuity of patient care OR case management OR comprehensive health care OR delivery of health care, integrated OR managed care programmes OR patient-centred care OR care delivery OR integrated care OR comprehensive care OR care coordination OR managed care OR accountable care OR accountable care organisations OR accountable care organisation OR collaborative care OR disease management OR case-management OR case management OR shared care OR accountable care OR patient-centred OR patient centred OR person-centred OR person centred OR multidisciplinary care OR interdisciplinary care OR inter-disciplinary care OR cross disciplinary care OR cross-disciplinary care OR multiple interventions OR care chain OR care chains OR care continuity OR care continuation OR care transition OR care transitions OR chain of care OR chains of care OR continuity of care OR cross sectoral care OR delivery of health care integrated OR integrated medicine OR integrated social network OR integrated social networks OR integration of care OR intersectoral care OR intrasectoral care OR linked care OR management model OR patient

For peer review only - http://bmjopen.bmj.com/site/about/guidelines.xhtml

care management OR seamless care OR service network OR service networks OR transition of care OR transitional care OR transmural care OR whole system thinking OR holistic care

#7 Angola OR Benin OR Botswana OR Burkina Faso OR Upper Volta OR Burundi OR Cameroon OR Cameroons OR Cape Verde OR Central African Republic OR Chad OR Comoros OR Comoro Islands OR Comores OR Mayotte OR Congo OR Zaire OR Cote d'Ivoire OR Ivory Coast OR Democratic Republic of the Congo OR Djibouti OR French Somaliland OR Eritrea OR Ethiopia OR Gabon OR Gabonese Republic OR Gambia OR Ghana OR Gold Coast OR Guinea OR Kenya OR Lesotho OR Basutoland OR Liberia OR Madagascar OR Malagasy Republic OR Malawi OR Nyasaland OR Mali OR Mauritania OR Mauritius OR Mozambique OR Namibia OR Niger OR Nigeria OR Rwanda OR Sao Tome OR Seychelles OR Senegal OR Sierra Leone OR Somalia OR South Africa OR Sudan OR Eswatini OR Tanzania OR Togo OR Togolese Republic OR Uganda OR Zambia OR Zimbabwe OR Rhodesia OR Sub-Saharan Africa OR Africa South of the Sahara

#8 trial* OR RCT

#9 ((#1 OR #2 OR #3 OR #4 OR #5) AND #6 AND #7 AND #8) and Clinical Trial (Document Types) and 1999 or 2000 or 2001 or 2002 or 2003 or 2004 or 2005 or 2006 or 2007 or 2008 or 2009 or 2011 or 2010 or 2012 or 2013 or 2014 or 2015 or 2016 or 2017 or 2018 or 2019 or 2020 or 2021 or 2022 (Publication Years)

3. SCOPUS

(TITLE-ABS-KEY (trial* OR rct)) AND (((TITLE-ABS-KEY ("wagner model" OR "wagner* model*" OR "wagner* chronic care model*" OR "wagner chronic care model" OR "chronic care model*" OR "model " OR "collaborative care" OR "chronic care framework" OR "chronic disease care" OR "chronic illness care" OR care AND model OR "model care" OR "wagner* model*" OR "wagner* chronic care model*" OR "wagner chronic care model" OR "theory" OR "concept" OR "framework" OR "model" OR "programme" OR "approach" OR "clinical pathway" OR "care pathway" OR "critical path" OR "vertical integration" OR "virtual integration" OR "physician system integration" OR "provider system integration" OR "functional integration" OR "horizontal integration" OR "clinical integration" OR "case management" OR "delivery of health care, integrated" OR "disease management" OR "patient care management" OR "patient-centred care")) OR (TITLE-ABS-KEY ("accountable care organisations" OR "continuity of patient care" OR "case management" OR "comprehensive health care" OR "delivery of health care, integrated" OR "managed care programmes" OR "patient-centred care" OR "care delivery" OR "integrated care" OR "comprehensive care" OR "care coordination" OR "managed care" OR "accountable care " OR "accountable care organisations" OR "accountable care organisation" OR "collaborative care" OR "disease management" OR "case-management" OR "case management" OR "shared care" OR "accountable care" OR "patient-centred" OR "patient centred" OR "person-centred" OR "person centred" OR "multidisciplinary care" OR "interdisciplinary care" OR "inter-disciplinary care" OR "cross disciplinary care" OR "cross-disciplinary care" OR "multiple interventions" OR "care chain" OR "care chains" OR "care continuity" OR "care continuation" OR "care transition")) OR (TITLE-ABS-KEY ("care transitions" OR "chain of care" OR "chains of care" OR "continuity of care" OR "cross sectoral care" OR "delivery of health care integrated" OR "integrated medicine" OR "integrated social network" OR "integrated social networks" OR "integration of care" OR "intersectoral care" OR "intrasectoral care" OR "linked care" OR "management model" OR "patient care management" OR "seamless care" OR "service network" OR "service networks" OR "transition of care" OR "transitional care" OR "transmural care" OR "whole system thinking" OR "holistic care"))) AND ((TITLE-ABS-KEY (("elevated blood pressure" OR "high blood pressure" OR "raised blood pressure" OR "diastolic blood pressure" OR "systolic blood pressure" OR "arterial blood pressure" OR "uncontrolled blood pressure" OR "uncontrolled hypertension" OR "elevated blood pressure" OR "high blood pressure" OR "raised blood pressure" OR "diastolic blood pressure" OR "systolic blood pressure" OR "arterial blood pressure" OR "uncontrolled blood pressure" OR "uncontrolled hypertension"))) OR (TITLE-ABS-KEY (("diabetes mellitus" OR "diabetes mellitus, type 2" OR "diabetes type 2" OR "t2dm" OR "diabetes type ii" OR "diabetes" OR "glucose intolerance" OR "insulin resistance" OR "hyperglycemia" OR "hyperglycaemia" OR "hypoglycaemia" OR "hypoglycaemia" OR "high blood sugar" OR "elevated blood sugar" OR "raised blood sugar" OR "high blood glucose" OR "elevated blood

For peer review only - http://bmjopen.bmj.com/site/about/guidelines.xhtml

glucose" OR "raised blood glucose" OR "diabetes mellitus" OR "diabetes mellitus, type 2" OR "diabetes type 2" OR "t2dm" OR "diabetes type ii" OR "diabetes" OR "glucose intolerance" OR "insulin resistance" OR "hyperglycemia" OR "hyperglycaemia" OR "hypoglycemia" OR "hypoglycaemia" OR "high blood sugar" OR "elevated blood sugar" OR "raised blood sugar" OR "high blood glucose" OR "elevated blood glucose" OR "raised blood glucose"))) OR (TITLE-ABS-KEY (("stroke" OR "transient ischemic attack" OR "ischemic attack" OR "angina" OR "angina pectoris" OR "heart attack" OR "ischemic heart diseases " OR "transient ischaemic attack" OR "coronary heart disease" OR "coronary disease" OR "heart failure" OR "peripheral vascular disease" OR "peripheral vascular diseases" OR "atrial fibrillation" OR "cardiovascular disease" OR "heart disease" OR "stroke" OR "transient ischemic attack" OR "ischemic attack" OR "angina" OR "angina pectoris" OR "heart attack" OR "ischemic heart diseases " OR "transient ischaemic attack" OR "coronary heart disease" OR "coronary disease" OR "heart failure" OR "peripheral vascular disease" OR "peripheral vascular diseases" OR "atrial fibrillation" OR "cardiovascular disease" OR "heart disease"))) OR (TITLE-ABS-KEY (("hypercholesterolemia" OR "dyslipidemias" OR "blood lipid" OR "cholesterol" OR "high cholesterol" OR "elevated cholesterol" OR "raised cholesterol" OR "low density lipoprotein " OR "high density lipoprotein" OR "dyslipidemia" OR "dyslipidaemia" OR "hypercholesterolemia" OR "hypercholesterolaemia " OR "hypercholesterolimia " OR "hypertriglyceridemia" OR "hypertriglyceridemia" OR "hypertriglyceridaemia" OR "hyperlipidemia" OR "hyperlipidemias" OR "hyperlipidaemia" OR "ldl" OR "hdl" OR "hypercholesterolemia" OR "dyslipidemias" OR "blood lipid" OR "cholesterol" OR "high cholesterol" OR "elevated cholesterol" OR "raised cholesterol" OR "low density lipoprotein " OR "high density lipoprotein" OR "dyslipidemia" OR "dyslipidaemia" OR "hypercholesterolemia" OR "hypercholesterolaemia " OR "hypercholesterolimia " OR "hypertriglyceridemia" OR "hypertriglyceridemia" OR "hypertriglyceridaemia" OR "hyperlipidemia" OR "hyperlipidemias" OR "hyperlipidaemia" OR "ldl" OR "hdl"))) OR (TITLE-ABS-KEY (("comorbid" OR "co-morbid" OR "co-morbidity" OR "multimorbidity" OR "multi-morbid" OR "multi morbidity" OR "multimorbidity" OR "comorbidity" OR "multi-disease" OR "multidisease" OR multi AND disease OR "multiple condition" OR "multi-condition" OR "multi condition" OR "multiple illness" OR "multi-illness" OR "multi illness" OR "multiple syndrome" OR "multi-syndrome" OR "multi syndrome" OR "concurrent condition" OR "concurrent illness" OR "concurrent disease" OR "co-existing disease" OR "coexisting disease" OR "co-existing illness" OR "coexisting illness" OR "co-existing syndrome" OR "coexisting syndrome" OR "co-existing condition" OR "coexisting condition" OR "co-occurring disease" OR "co-occuring disease" OR "co-occuring disease" OR "co-occurring illness" OR "co occurring illness" OR "cooccurring illness" OR "co-occurring syndrome" OR "co occurring syndrome" OR "cooccurring syndrome" OR "co-occurring condition" OR "co occurring condition" OR "cooccurring condition")))) AND (TITLE-ABS-KEY (("angola " OR "benin " OR "botswana " OR "burkina faso" OR "upper volta" OR "burundi " OR "cameroon " OR "cameroons " OR "cape verde" OR "central african republic" OR chad OR "comoros " OR "comoro islands" OR "comores" OR "mayotte " OR "congo " OR "zaire " OR "cote d'ivoire" OR "ivory coast" OR "democratic republic of the congo" OR "djibouti " OR "french somaliland" OR "eritrea " OR "ethiopia " OR "gabon " OR "gabonese republic" OR "gambia " OR "ghana " OR "gold coast" OR "guinea " OR "kenya " OR "lesotho" OR "basutoland" OR "liberia " OR "madagascar " OR "malagasy republic " OR "malawi " OR "nyasaland " OR "mali " OR "mauritania " OR "mauritius " OR "mozambique" OR "namibia" OR "niger" OR "nigeria" OR "rwanda" OR "sao tome" OR "seychelles" OR "senegal " OR "sierra leone" OR "somalia " OR "south africa" OR "sudan " OR "Eswatini" OR "tanzania" OR "togo " OR "Togolese republic" OR "uganda" OR "zambia" OR "zimbabwe " OR "rhodesia" OR "sub-saharan africa" OR "africa south of the sahara")))) AND (LIMIT-TO (PUBYEAR , 2022) OR LIMIT-TO (PUBYEAR , 2021) OR LIMIT-TO (PUBYEAR , 2020) OR LIMIT-TO (PUBYEAR , 2019) OR LIMIT-TO (PUBYEAR , 2018) OR LIMIT-TO (PUBYEAR , 2017) OR LIMIT-TO (PUBYEAR , 2016) OR LIMIT-TO (PUBYEAR , 2015) OR LIMIT-TO (PUBYEAR , 2014) OR LIMIT-TO (PUBYEAR , 2013) OR LIMIT-TO (PUBYEAR , 2012) OR LIMIT-TO (PUBYEAR , 2011) OR LIMIT-TO (PUBYEAR , 2010) OR LIMIT-TO (PUBYEAR , 2009) OR LIMIT-TO (PUBYEAR , 2008) OR LIMIT-TO (PUBYEAR , 2007) OR LIMIT-TO (PUBYEAR , 2006) OR LIMIT-TO (PUBYEAR , 2005) OR LIMIT-

For peer review only - http://bmjopen.bmj.com/site/about/guidelines.xhtml

TO (PUBYEAR , 2004) OR LIMIT-TO (PUBYEAR , 2003) OR LIMIT-TO (PUBYEAR , 2002) OR LIMIT-TO (PUBYEAR , 2001) OR LIMIT-TO (PUBYEAR , 2000) OR LIMIT-TO (PUBYEAR , 1999))

4. Embase

#1 ("elevated blood pressure"/ OR "high blood pressure"/ OR "raised blood pressure"/ OR "diastolic blood pressure"/ OR "systolic blood pressure"/ OR "arterial blood pressure"/ OR "uncontrolled blood pressure"/ OR "uncontrolled hypertension" OR "elevated blood pressure" OR "high blood pressure" OR "raised blood pressure" OR "diastolic blood pressure" OR "systolic blood pressure" OR "arterial blood pressure" OR "uncontrolled blood pressure" OR "uncontrolled hypertension")

#2 ("diabetes mellitus"/ OR "diabetes mellitus, type 2"/ OR "diabetes type 2"/ OR "T2DM"/ OR "diabetes type II"/ OR "diabetes"/ OR "glucose intolerance"/ OR "insulin resistance"/ OR "hyperglycemia"/ OR "hyperglycaemia"/ OR "hypoglycaemia"/ OR "hypoglycaemia"/ OR "high blood sugar"/ OR "elevated blood sugar"/ OR "raised blood sugar"/ OR "high blood glucose"/ OR "elevated blood glucose"/ OR "raised blood glucose"/ OR "diabetes mellitus" OR "diabetes mellitus, type 2" OR "diabetes type 2" OR "T2DM" OR "diabetes type II" OR "diabetes" OR "glucose intolerance" OR "insulin resistance" OR "hyperglycemia" OR "hyperglycaemia" OR "hypoglycemia" OR "hypoglycaemia" OR "high blood sugar" OR "elevated blood sugar" OR "raised blood sugar" OR "high blood glucose" OR "elevated blood glucose" OR "raised blood glucose")

#3 ("stroke"/ OR "transient ischemic attack"/ OR "ischemic attack"/ OR "angina"/ OR "angina pectoris"/ OR "heart attack"/ OR "ischemic heart diseases "/ OR "transient ischaemic attack"/ OR "coronary heart disease"/ OR "coronary disease"/ OR "heart failure"/ OR "peripheral vascular disease"/ OR "Peripheral Vascular diseases"/ OR "atrial fibrillation"/ OR "cardiovascular disease"/ OR "heart disease"/ OR "stroke" OR "transient ischemic attack" OR "ischemic attack" OR "angina" OR "angina pectoris" OR "heart attack" OR "ischemic heart diseases " OR "transient ischaemic attack" OR "coronary heart disease" OR "coronary disease" OR "heart failure" OR "peripheral vascular disease" OR "Peripheral Vascular diseases" OR "atrial fibrillation" OR "cardiovascular disease" OR "heart disease")

#4 ("Hypercholesterolemia"/ OR "Dyslipidemias"/ OR "blood lipid"/ OR "Cholesterol"/ OR "high cholesterol"/ OR "elevated cholesterol"/ OR "raised cholesterol"/ OR "low density lipoprotein "/ OR "high density lipoprotein"/ OR "Dyslipidemia"/ OR "Dyslipidaemia"/ OR "Hypercholesterolemia"/ OR "hypercholesterolaemia "/ OR "hypercholesterolimia "/ OR "Hypertriglyceridemia"/ OR "Hypertriglyceridemia"/ OR "Hypertriglyceridaemia"/ OR "Hyperlipidemia"/ OR "Hyperlipidemias"/ OR "Hyperlipidaemia"/ OR "LDL"/ OR "HDL"/ OR "Hypercholesterolemia" OR "Dyslipidemias" OR "blood lipid" OR "Cholesterol" OR "high cholesterol" OR "elevated cholesterol" OR "raised cholesterol" OR "low density lipoprotein " OR "high density lipoprotein" OR "Dyslipidemia" OR "Dyslipidaemia" OR "Hypercholesterolemia" OR "hypercholesterolaemia " OR "hypercholesterolimia " OR "Hypertriglyceridemia" OR "Hypertriglyceridemia" OR "Hypertriglyceridaemia" OR "Hyperlipidemia" OR "Hyperlipidemias" OR "Hyperlipidaemia" OR "LDL" OR "HDL")

#5 ("Comorbid" OR "co-morbid" OR "co-morbidity" OR "Multimorbidity" OR "multi-morbid" OR "multi morbidity" OR "Multimorbidity" OR "Comorbidity" OR "multi-disease" OR "Multidisease" OR multi disease OR "multiple condition" OR "multi-condition" OR "multi condition" OR "multiple illness" OR "multi-illness" OR "multi illness" OR "multiple syndrome" OR "multi-syndrome" OR "multi syndrome" OR "concurrent condition" OR "concurrent illness" OR "concurrent disease" OR "co-existing disease" OR "coexisting disease" OR "co-existing illness" OR "coexisting illness" OR "co-existing syndrome" OR "coexisting syndrome" OR "co-existing condition" OR "coexisting condition" OR "co-occurring disease" OR "co-occuring disease" OR "co-occuring disease" OR "co-occurring illness" OR "co occurring illness" OR "cooccurring illness" OR "co-occurring syndrome" OR "co occurring syndrome" OR "cooccurring syndrome" OR "co-occurring condition" OR "co occurring condition" OR "cooccurring condition")

#6 ("Wagner model" OR "Wagner* model*" OR "Wagner* chronic care model*" OR "Wagner chronic care model" OR "chronic care model*" OR "model " OR "collaborative care" OR "chronic care framework" OR "chronic disease care" OR "chronic illness care" OR care model OR "model care" OR "Wagner* model*" OR "Wagner* chronic care model*" OR "Wagner chronic care model" OR "theory" OR "concept" OR "framework" OR "model"

For peer review only - http://bmjopen.bmj.com/site/about/guidelines.xhtml

OR "programme" OR "approach" OR "clinical pathway" OR "care pathway" OR "critical path" OR "vertical integration" OR "virtual integration" OR "physician system integration" OR "provider system integration" OR "functional integration" OR "horizontal integration" OR "clinical integration" OR "case management" OR "delivery of health care, integrated" OR "disease management" OR "patient care management" OR "patient-centred care" OR "accountable care organisations" OR "continuity of patient care" OR "case management" OR "comprehensive health care" OR "delivery of health care, integrated" OR "managed care programmes" OR "patient-centred care" OR "care delivery" OR "integrated care" OR "comprehensive care" OR "care coordination" OR "managed care" OR "accountable care " OR "accountable care organisations" OR "accountable care organisation" OR "collaborative care" OR "disease management" OR "case-management" OR "case management" OR "shared care" OR "accountable care" OR "patient-centred" OR "patient centred" OR "person-centred" OR "person centred" OR "multidisciplinary care" OR "interdisciplinary care" OR "inter-disciplinary care" OR "cross disciplinary care" OR "cross-disciplinary care" OR "multiple interventions" OR "care chain" OR "care chains" OR "care continuity" OR "care continuation" OR "care transition" OR "care transitions" OR "chain of care" OR "chains of care" OR "continuity of care" OR "cross sectoral care" OR "delivery of health care integrated" OR "integrated medicine" OR "integrated social network" OR "integrated social networks" OR "integration of care" OR "intersectoral care" OR "intrasectoral care" OR "linked care" OR "management model" OR "patient care management" OR "seamless care" OR "service network" OR "service networks" OR "transition of care" OR "transitional care" OR "transmural care" OR "whole system thinking" OR "holistic care")

#7 ("Angola " OR "Benin " OR "Botswana " OR "Burkina Faso" OR "Upper Volta" OR "Burundi " OR "Cameroon " OR "Cameroons " OR "Cape Verde" OR "Central African Republic" OR Chad OR "Comoros " OR "Comoro Islands" OR "Comores" OR "Mayotte " OR "Congo " OR "Zaire " OR "Cote d'Ivoire" OR "Ivory Coast" OR "Democratic Republic of the Congo" OR "Djibouti " OR "French Somaliland" OR "Eritrea " OR "Ethiopia " OR "Gabon " OR "Gabonese Republic" OR "Gambia " OR "Ghana " OR "Gold Coast" OR "Guinea " OR "Kenya " OR "Lesotho" OR "Basutoland" OR "Liberia " OR "Madagascar " OR "Malagasy Republic " OR "Malawi " OR "Nyasaland " OR "Mali " OR "Mauritania " OR "Mauritius " OR "Mozambique" OR "Namibia" OR "Niger" OR "Nigeria" OR "Rwanda" OR "Sao Tome" OR "Seychelles" OR "Senegal " OR "Sierra Leone" OR "Somalia " OR "South Africa" OR "Sudan " OR "Eswatini" OR "Tanzania" OR "Togo " OR "Togolese Republic" OR "Uganda" OR "Zambia" OR "Zimbabwe " OR "Rhodesia" OR "Sub-Saharan Africa" OR "Africa South of the Sahara")

#8 ((#1 OR #2 OR #3 OR #4 OR #5) AND #6 AND #7) & 1999 - 2022 & RCTs

5. **Cochrane Library**

#1 (("elevated blood pressure"(MeSH Terms) OR "high blood pressure"(MeSH Terms) OR "raised blood pressure"(MeSH Terms) OR "diastolic blood pressure"(MeSH Terms) OR "systolic blood pressure"(MeSH Terms) OR "arterial blood pressure"(MeSH Terms) OR "uncontrolled blood pressure"(MeSH Terms) OR "uncontrolled hypertension" OR "elevated blood pressure" OR "high blood pressure" OR "raised blood pressure" OR "diastolic blood pressure" OR "systolic blood pressure" OR "arterial blood pressure" OR "uncontrolled blood pressure" OR "uncontrolled hypertension")):ti,ab,kw (Word variations have been searched)

#2 (("diabetes mellitus"(MeSH Terms) OR "diabetes mellitus, type 2"(MeSH Terms) OR "diabetes type 2"(MeSH Terms) OR "T2DM"(MeSH Terms) OR "diabetes type II"(MeSH Terms) OR "diabetes"(MeSH Terms) OR "glucose intolerance"(MeSH Terms) OR "insulin resistance"(MeSH Terms) OR "hyperglycemia"(MeSH Terms) OR "hyperglycaemia"(MeSH Terms) OR "hypoglycaemia"(MeSH Terms) OR "hypoglycaemia"(MeSH Terms) OR "high blood sugar"(MeSH Terms) OR "elevated blood sugar"(MeSH Terms) OR "raised blood sugar"(MeSH Terms) OR "high blood glucose"(MeSH Terms) OR "elevated blood glucose"(MeSH Terms) OR "raised blood glucose"(MeSH Terms) OR "diabetes mellitus" OR "diabetes mellitus, type 2" OR "diabetes type 2" OR "T2DM" OR "diabetes type II" OR "diabetes" OR "glucose intolerance" OR "insulin resistance" OR "hyperglycemia" OR "hyperglycaemia" OR "hypoglycemia" OR "hypoglycaemia" OR "high blood sugar" OR "elevated blood sugar" OR "raised blood sugar" OR "high blood glucose" OR "elevated blood glucose" OR "raised blood glucose")):ti,ab,kw (Word variations have been searched)

For peer review only - http://bmjopen.bmj.com/site/about/guidelines.xhtml

#3 (("stroke"(MeSH Terms) OR "transient ischemic attack"(MeSH Terms) OR "ischemic attack"(MeSH Terms) OR "angina"(MeSH Terms) OR "angina pectoris"(MeSH Terms) OR "heart attack"(MeSH Terms) OR "ischemic heart diseases "(MeSH Terms) OR "transient ischaemic attack"(MeSH Terms) OR "coronary heart disease"(MeSH Terms) OR "coronary disease"(MeSH Terms) OR "heart failure"(MeSH Terms) OR "peripheral vascular disease"(MeSH Terms) OR "Peripheral Vascular diseases"(MeSH Terms) OR "atrial fibrillation"(MeSH Terms) OR "cardiovascular disease"(MeSH Terms) OR "heart disease"(MeSH Terms) OR "stroke" OR "transient ischemic attack" OR "ischemic attack" OR "angina" OR "angina pectoris" OR "heart attack" OR "ischemic heart diseases " OR "transient ischaemic attack" OR "coronary heart disease" OR "coronary disease" OR "heart failure" OR "peripheral vascular disease" OR "Peripheral Vascular diseases" OR "atrial fibrillation" OR "cardiovascular disease" OR "heart disease")):ti,ab,kw (Word variations have been searched)

#4 (("Hypercholesterolemia"(MeSH Terms) OR "Dyslipidemias"(MeSH Terms) OR "blood lipid"(MeSH Terms) OR "Cholesterol"(MeSH Terms) OR "high cholesterol"(MeSH Terms) OR "elevated cholesterol"(MeSH Terms) OR "raised cholesterol"(MeSH Terms) OR "low density lipoprotein "(MeSH Terms) OR "high density lipoprotein"(MeSH Terms) OR "Dyslipidemia"(MeSH Terms) OR "Dyslipidaemia"(MeSH Terms) OR "Hypercholesterolemia"(MeSH Terms) OR "hypercholesterolaemia "(MeSH Terms) OR "hypercholesterolimia "(MeSH Terms) OR "Hypertriglyceridemia"(MeSH Terms) OR "Hypertriglyceridemia"(MeSH Terms) OR "Hypertriglyceridaemia"(MeSH Terms) OR "Hyperlipidemia"(MeSH Terms) OR "Hyperlipidemias"(MeSH Terms) OR "Hyperlipidaemia"(MeSH Terms) OR "LDL"(MeSH Terms) OR "HDL"(MeSH Terms) OR "Hypercholesterolemia" OR "Dyslipidemias" OR "blood lipid" OR "Cholesterol" OR "high cholesterol" OR "elevated cholesterol" OR "raised cholesterol" OR "low density lipoprotein " OR "high density lipoprotein" OR "Dyslipidemia" OR "Dyslipidaemia" OR "Hypercholesterolemia" OR "hypercholesterolaemia " OR "hypercholesterolimia " OR "Hypertriglyceridemia" OR "Hypertriglyceridemia" OR "Hypertriglyceridaemia" OR "Hyperlipidemia" OR "Hyperlipidemias" OR "Hyperlipidaemia" OR "LDL" OR "HDL")):ti,ab,kw (Word variations have been searched)

#5 (("Comorbid" OR "co-morbid" OR "co-morbidity" OR "Multimorbidity" OR "multi-morbid" OR "multi morbidity" OR "Multimorbidity" OR "Comorbidity" OR "multi-disease" OR "Multidisease" OR multi disease OR "multiple condition" OR "multi-condition" OR "multi condition" OR "multiple illness" OR "multi-illness" OR "multi illness" OR "multiple syndrome" OR "multi-syndrome" OR "multi syndrome" OR "concurrent condition" OR "concurrent illness" OR "concurrent disease" OR "co-existing disease" OR "coexisting disease" OR "co-existing illness" OR "coexisting illness" OR "co-existing syndrome" OR "coexisting syndrome" OR "co-existing condition" OR "coexisting condition" OR "co-occurring disease" OR "co-occuring disease" OR "co-occuring disease" OR "co-occurring illness" OR "co occurring illness" OR "cooccurring illness" OR "co-occurring syndrome" OR "co occurring syndrome" OR "cooccurring syndrome" OR "co-occurring condition" OR "co occurring condition" OR "cooccurring condition")):ti,ab,kw (Word variations have been searched)

#6 (("Wagner model" OR "Wagner* model*" OR "Wagner* chronic care model*" OR "Wagner chronic care model" OR "chronic care model*" OR "model " OR "collaborative care" OR "chronic care framework" OR "chronic disease care" OR "chronic illness care" OR care model OR "model care" OR "Wagner* model*" OR "Wagner* chronic care model*" OR "Wagner chronic care model" OR "theory" OR "concept" OR "framework" OR "model" OR "programme" OR "approach" OR "clinical pathway" OR "care pathway" OR "critical path" OR "vertical integration" OR "virtual integration" OR "physician system integration" OR "provider system integration" OR "functional integration" OR "horizontal integration" OR "clinical integration" OR "case management" OR "delivery of health care, integrated" OR "disease management" OR "patient care management" OR "patient-centred care" OR "accountable care organisations" OR "continuity of patient care" OR "case management" OR "comprehensive health care" OR "delivery of health care, integrated" OR "managed care programmes" OR "patient-centred care" OR "care delivery" OR "integrated care" OR "comprehensive care" OR "care coordination" OR "managed care" OR "accountable care " OR "accountable care organisations" OR "accountable care organisation" OR "collaborative care" OR "disease management" OR "case-management" OR "case management" OR "shared care" OR "accountable care" OR "patient-centred" OR "patient centred" OR "person-centred" OR "person centred" OR "multidisciplinary care" OR "interdisciplinary care" OR "inter-disciplinary care" OR "cross disciplinary care" OR "cross-disciplinary care" OR "multiple interventions" OR "care chain" OR "care chains" OR "care continuity" OR

For peer review only - http://bmjopen.bmj.com/site/about/guidelines.xhtml

"care continuation" OR "care transition" OR "care transitions" OR "chain of care" OR "chains of care" OR "continuity of care" OR "cross sectoral care" OR "delivery of health care integrated" OR "integrated medicine" OR "integrated social network" OR "integrated social networks" OR "integration of care" OR "intersectoral care" OR "intrasectoral care" OR "linked care" OR "management model" OR "patient care management" OR "seamless care" OR "service network" OR "service networks" OR "transition of care" OR "transitional care" OR "transmural care" OR "whole system thinking" OR "holistic care")):ti,ab,kw (Word variations have been searched)

#7 (("Angola " OR "Benin " OR "Botswana " OR "Burkina Faso" OR "Upper Volta" OR "Burundi " OR "Cameroon " OR "Cameroons " OR "Cape Verde" OR "Central African Republic" OR Chad OR "Comoros " OR "Comoro Islands" OR "Comores" OR "Mayotte " OR "Congo " OR "Zaire " OR "Cote d'Ivoire" OR "Ivory Coast" OR "Democratic Republic of the Congo" OR "Djibouti " OR "French Somaliland" OR "Eritrea " OR "Ethiopia " OR "Gabon " OR "Gabonese Republic" OR "Gambia " OR "Ghana " OR "Gold Coast" OR "Guinea " OR "Kenya " OR "Lesotho" OR "Basutoland" OR "Liberia " OR "Madagascar " OR "Malagasy Republic " OR "Malawi " OR "Nyasaland " OR "Mali " OR "Mauritania " OR "Mauritius " OR "Mozambique" OR "Namibia" OR "Niger" OR "Nigeria" OR "Rwanda" OR "Sao Tome" OR "Seychelles" OR "Senegal " OR "Sierra Leone" OR "Somalia " OR "South Africa" OR "Sudan " OR "Eswatini" OR "Tanzania" OR "Togo " OR "Togolese Republic" OR "Uganda" OR "Zambia" OR "Zimbabwe " OR "Rhodesia" OR "Sub-Saharan Africa" OR "Africa South of the Sahara")):ti,ab,kw (Word variations have been searched)

#8 ((#1 OR #2 OR #3 OR #4 OR #5) AND #6 AND #7) AND Restricted (1999-2022, RCTs)

6. Psycinfo via Ovid

#1 ("elevated blood pressure"/ OR "high blood pressure"/ OR "raised blood pressure"/ OR "diastolic blood pressure"/ OR "systolic blood pressure"/ OR "arterial blood pressure"/ OR "uncontrolled blood pressure"/ OR "uncontrolled hypertension" OR "elevated blood pressure" OR "high blood pressure" OR "raised blood pressure" OR "diastolic blood pressure" OR "systolic blood pressure" OR "arterial blood pressure" OR "uncontrolled blood pressure" OR "uncontrolled hypertension")

#2 ("diabetes mellitus"/ OR "diabetes mellitus, type 2"/ OR "diabetes type 2"/ OR "T2DM"/ OR "diabetes type II"/ OR "diabetes"/ OR "glucose intolerance"/ OR "insulin resistance"/ OR "hyperglycemia"/ OR "hyperglycaemia"/ OR "hypoglycaemia"/ OR "hypoglycaemia"/ OR "high blood sugar"/ OR "elevated blood sugar"/ OR "raised blood sugar"/ OR "high blood glucose"/ OR "elevated blood glucose"/ OR "raised blood glucose"/ OR "diabetes mellitus" OR "diabetes mellitus, type 2" OR "diabetes type 2" OR "T2DM" OR "diabetes type II" OR "diabetes" OR "glucose intolerance" OR "insulin resistance" OR "hyperglycemia" OR "hyperglycaemia" OR "hypoglycemia" OR "hypoglycaemia" OR "high blood sugar" OR "elevated blood sugar" OR "raised blood sugar" OR "high blood glucose" OR "elevated blood glucose" OR "raised blood glucose")

#3 ("stroke"/ OR "transient ischemic attack"/ OR "ischemic attack"/ OR "angina"/ OR "angina pectoris"/ OR "heart attack"/ OR "ischemic heart diseases "/ OR "transient ischaemic attack"/ OR "coronary heart disease"/ OR "coronary disease"/ OR "heart failure"/ OR "peripheral vascular disease"/ OR "Peripheral Vascular diseases"/ OR "atrial fibrillation"/ OR "cardiovascular disease"/ OR "heart disease"/ OR "stroke" OR "transient ischemic attack" OR "ischemic attack" OR "angina" OR "angina pectoris" OR "heart attack" OR "ischemic heart diseases " OR "transient ischaemic attack" OR "coronary heart disease" OR "coronary disease" OR "heart failure" OR "peripheral vascular disease" OR "Peripheral Vascular diseases" OR "atrial fibrillation" OR "cardiovascular disease" OR "heart disease")

#4 ("Hypercholesterolemia"/ OR "Dyslipidemias"/ OR "blood lipid"/ OR "Cholesterol"/ OR "high cholesterol"/ OR "elevated cholesterol"/ OR "raised cholesterol"/ OR "low density lipoprotein "/ OR "high density lipoprotein"/ OR "Dyslipidemia"/ OR "Dyslipidaemia"/ OR "Hypercholesterolemia"/ OR "hypercholesterolaemia "/ OR "hypercholesterolimia "/ OR "Hypertriglyceridemia"/ OR "Hypertriglyceridemia"/ OR "Hypertriglyceridaemia"/ OR "Hyperlipidemia"/ OR "Hyperlipidemias"/ OR "Hyperlipidaemia"/ OR "LDL"/ OR "HDL"/ OR "Hypercholesterolemia" OR "Dyslipidemias" OR "blood lipid" OR "Cholesterol" OR "high cholesterol" OR "elevated cholesterol" OR "raised cholesterol" OR "low density lipoprotein " OR "high density lipoprotein" OR

For peer review only - http://bmjopen.bmj.com/site/about/guidelines.xhtml

"Dyslipidemia" OR "Dyslipidaemia" OR "Hypercholesterolemia" OR "hypercholesterolaemia " OR "hypercholesterolimia " OR "Hypertriglyceridemia" OR "Hypertriglyceridemia" OR "Hypertriglyceridaemia" OR "Hyperlipidemia" OR "Hyperlipidemias" OR "Hyperlipidaemia" OR "LDL" OR "HDL")

#5 ("Comorbid" OR "co-morbid" OR "co-morbidity" OR "Multimorbidity" OR "multi-morbid" OR "multi morbidity" OR "Multimorbidity" OR "Comorbidity" OR "multi-disease" OR "Multidisease" OR multi disease OR "multiple condition" OR "multi-condition" OR "multi condition" OR "multiple illness" OR "multi-illness" OR "multi illness" OR "multiple syndrome" OR "multi-syndrome" OR "multi syndrome" OR "concurrent condition" OR "concurrent illness" OR "concurrent disease" OR "co-existing disease" OR "coexisting disease" OR "co-existing illness" OR "coexisting illness" OR "co-existing syndrome" OR "coexisting syndrome" OR "co-existing condition" OR "coexisting condition" OR "co-occurring disease" OR "co-occuring disease" OR "co-occuring disease" OR "co-occurring illness" OR "co occurring illness" OR "cooccurring illness" OR "co-occurring syndrome" OR "co occurring syndrome" OR "cooccurring syndrome" OR "co-occurring condition" OR "co occurring condition" OR "cooccurring condition")

#6 ("Wagner model" OR "Wagner* model*" OR "Wagner* chronic care model*" OR "Wagner chronic care model" OR "chronic care model*" OR "model " OR "collaborative care" OR "chronic care framework" OR "chronic disease care" OR "chronic illness care" OR care model OR "model care" OR "Wagner* model*" OR "Wagner* chronic care model*" OR "Wagner chronic care model" OR "theory" OR "concept" OR "framework" OR "model" OR "programme" OR "approach" OR "clinical pathway" OR "care pathway" OR "critical path" OR "vertical integration" OR "virtual integration" OR "physician system integration" OR "provider system integration" OR "functional integration" OR "horizontal integration" OR "clinical integration" OR "case management" OR "delivery of health care, integrated" OR "disease management" OR "patient care management" OR "patient-centred care" OR "accountable care organisations" OR "continuity of patient care" OR "case management" OR "comprehensive health care" OR "delivery of health care, integrated" OR "managed care programmes" OR "patient-centred care" OR "care delivery" OR "integrated care" OR "comprehensive care" OR "care coordination" OR "managed care" OR "accountable care " OR "accountable care organisations" OR "accountable care organisation" OR "collaborative care" OR "disease management" OR "case-management" OR "case management" OR "shared care" OR "accountable care" OR "patient-centred" OR "patient centred" OR "person-centred" OR "person centred" OR "multidisciplinary care" OR "interdisciplinary care" OR "inter-disciplinary care" OR "cross disciplinary care" OR "cross-disciplinary care" OR "multiple interventions" OR "care chain" OR "care chains" OR "care continuity" OR "care continuation" OR "care transition" OR "care transitions" OR "chain of care" OR "chains of care" OR "continuity of care" OR "cross sectoral care" OR "delivery of health care integrated" OR "integrated medicine" OR "integrated social network" OR "integrated social networks" OR "integration of care" OR "intersectoral care" OR "intrasectoral care" OR "linked care" OR "management model" OR "patient care management" OR "seamless care" OR "service network" OR "service networks" OR "transition of care" OR "transitional care" OR "transmural care" OR "whole system thinking" OR "holistic care")

#7 ("Angola " OR "Benin " OR "Botswana " OR "Burkina Faso" OR "Upper Volta" OR "Burundi " OR "Cameroon " OR "Cameroons " OR "Cape Verde" OR "Central African Republic" OR Chad OR "Comoros " OR "Comoro Islands" OR "Comores" OR "Mayotte " OR "Congo " OR "Zaire " OR "Cote d'Ivoire" OR "Ivory Coast" OR "Democratic Republic of the Congo" OR "Djibouti " OR "French Somaliland" OR "Eritrea " OR "Ethiopia " OR "Gabon " OR "Gabonese Republic" OR "Gambia " OR "Ghana " OR "Gold Coast" OR "Guinea " OR "Kenya " OR "Lesotho" OR "Basutoland" OR "Liberia " OR "Madagascar " OR "Malagasy Republic " OR "Malawi " OR "Nyasaland " OR "Mali " OR "Mauritania " OR "Mauritius " OR "Mozambique" OR "Namibia" OR "Niger" OR "Nigeria" OR "Rwanda" OR "Sao Tome" OR "Seychelles" OR "Senegal " OR "Sierra Leone" OR "Somalia " OR "South Africa" OR "Sudan " OR "Eswatini" OR "Tanzania" OR "Togo " OR "Togolese Republic" OR "Uganda" OR "Zambia" OR "Zimbabwe " OR "Rhodesia" OR "Sub-Saharan Africa" OR "Africa South of the Sahara")

8 ((#1 OR #2 OR #3 OR #4 OR #5) AND #6 AND #7)

9 limit 8 to ("0300 clinical trial" and yr="1999 - 2022")

7. CINAHL

For peer review only - http://bmjopen.bmj.com/site/about/guidelines.xhtml

#1 ("elevated blood pressure" OR "high blood pressure" OR "raised blood pressure" OR "diastolic blood pressure" OR "systolic blood pressure" OR "arterial blood pressure" OR "uncontrolled blood pressure" OR "uncontrolled hypertension" OR "elevated blood pressure" OR "high blood pressure" OR "raised blood pressure" OR "diastolic blood pressure" OR "systolic blood pressure" OR "arterial blood pressure" OR "uncontrolled blood pressure" OR "uncontrolled hypertension")

#2 ("diabetes mellitus" OR "diabetes mellitus, type 2" OR "diabetes type 2" OR "T2DM" OR "diabetes type II" OR "diabetes" OR "glucose intolerance" OR "insulin resistance" OR "hyperglycemia" OR "hyperglycaemia" OR "hypoglycaemia" OR "hypoglycaemia" OR "high blood sugar" OR "elevated blood sugar" OR "raised blood sugar" OR "high blood glucose" OR "elevated blood glucose" OR "raised blood glucose" OR "diabetes mellitus" OR "diabetes mellitus, type 2" OR "diabetes type 2" OR "T2DM" OR "diabetes type II" OR "diabetes" OR "glucose intolerance" OR "insulin resistance" OR "hyperglycemia" OR "hyperglycaemia" OR "hypoglycemia" OR "hypoglycaemia" OR "high blood sugar" OR "elevated blood sugar" OR "raised blood sugar" OR "high blood glucose" OR "elevated blood glucose" OR "raised blood glucose")

#3 ("stroke" OR "transient ischemic attack" OR "ischemic attack" OR "angina" OR "angina pectoris" OR "heart attack" OR "ischemic heart diseases " OR "transient ischaemic attack" OR "coronary heart disease" OR "coronary disease" OR "heart failure" OR "peripheral vascular disease" OR "Peripheral Vascular diseases" OR "atrial fibrillation" OR "cardiovascular disease" OR "heart disease" OR "stroke" OR "transient ischemic attack" OR "ischemic attack" OR "angina" OR "angina pectoris" OR "heart attack" OR "ischemic heart diseases " OR "transient ischaemic attack" OR "coronary heart disease" OR "coronary disease" OR "heart failure" OR "peripheral vascular disease" OR "Peripheral Vascular diseases" OR "atrial fibrillation" OR "cardiovascular disease" OR "heart disease")

#4 ("Hypercholesterolemia" OR "Dyslipidemias" OR "blood lipid" OR "Cholesterol" OR "high cholesterol" OR "elevated cholesterol" OR "raised cholesterol" OR "low density lipoprotein " OR "high density lipoprotein" OR "Dyslipidemia" OR "Dyslipidaemia" OR "Hypercholesterolemia" OR "hypercholesterolaemia " OR "hypercholesterolimia " OR "Hypertriglyceridemia" OR "Hypertriglyceridemia" OR "Hypertriglyceridaemia" OR "Hyperlipidemia" OR "Hyperlipidemias" OR "Hyperlipidaemia" OR "LDL" OR "HDL" OR "Hypercholesterolemia" OR "Dyslipidemias" OR "blood lipid" OR "Cholesterol" OR "high cholesterol" OR "elevated cholesterol" OR "raised cholesterol" OR "low density lipoprotein " OR "high density lipoprotein" OR "Dyslipidemia" OR "Dyslipidaemia" OR "Hypercholesterolemia" OR "hypercholesterolaemia " OR "hypercholesterolimia " OR "Hypertriglyceridemia" OR "Hypertriglyceridemia" OR "Hypertriglyceridaemia" OR "Hyperlipidemia" OR "Hyperlipidemias" OR "Hyperlipidaemia" OR "LDL" OR "HDL")

#5 ("Comorbid" OR "co-morbid" OR "co-morbidity" OR "Multimorbidity" OR "multi-morbid" OR "multi morbidity" OR "Multimorbidity" OR "Comorbidity" OR "multi-disease" OR "Multidisease" OR multi disease OR "multiple condition" OR "multi-condition" OR "multi condition" OR "multiple illness" OR "multi-illness" OR "multi illness" OR "multiple syndrome" OR "multi-syndrome" OR "multi syndrome" OR "concurrent condition" OR "concurrent illness" OR "concurrent disease" OR "co-existing disease" OR "coexisting disease" OR "co-existing illness" OR "coexisting illness" OR "co-existing syndrome" OR "coexisting syndrome" OR "co-existing condition" OR "coexisting condition" OR "co-occurring disease" OR "co-occuring disease" OR "co-occuring disease" OR "co-occurring illness" OR "co occurring illness" OR "cooccurring illness" OR "co-occurring syndrome" OR "co occurring syndrome" OR "cooccurring syndrome" OR "co-occurring condition" OR "co occurring condition" OR "cooccurring condition")

#6 ("Wagner model" OR "Wagner* model*" OR "Wagner* chronic care model*" OR "Wagner chronic care model" OR "chronic care model*" OR "model " OR "collaborative care" OR "chronic care framework" OR "chronic disease care" OR "chronic illness care" OR care model OR "model care" OR "Wagner* model*" OR "Wagner* chronic care model*" OR "Wagner chronic care model" OR "theory" OR "concept" OR "framework" OR "model" OR "programme" OR "approach" OR "clinical pathway" OR "care pathway" OR "critical path" OR "vertical integration" OR "virtual integration" OR "physician system integration" OR "provider system integration" OR "functional integration" OR "horizontal integration" OR "clinical integration" OR "case management" OR "delivery of health care, integrated" OR "disease management" OR "patient care management" OR "patient-centred care" OR "accountable care organisations" OR "continuity of patient care" OR "case management" OR "comprehensive health

For peer review only - http://bmjopen.bmj.com/site/about/guidelines.xhtml

care" OR "delivery of health care, integrated" OR "managed care programmes" OR "patient-centred care" OR "care delivery" OR "integrated care" OR "comprehensive care" OR "care coordination" OR "managed care" OR "accountable care " OR "accountable care organisations" OR "accountable care organisation" OR "collaborative care" OR "disease management" OR "case-management" OR "case management" OR "shared care" OR "accountable care" OR "patient-centred" OR "patient centred" OR "person-centred" OR "person centred" OR "multidisciplinary care" OR "interdisciplinary care" OR "inter-disciplinary care" OR "cross disciplinary care" OR "cross-disciplinary care" OR "multiple interventions" OR "care chain" OR "care chains" OR "care continuity" OR "care continuation" OR "care transition" OR "care transitions" OR "chain of care" OR "chains of care" OR "continuity of care" OR "cross sectoral care" OR "delivery of health care integrated" OR "integrated medicine" OR "integrated social network" OR "integrated social networks" OR "integration of care" OR "intersectoral care" OR "intrasectoral care" OR "linked care" OR "management model" OR "patient care management" OR "seamless care" OR "service network" OR "service networks" OR "transition of care" OR "transitional care" OR "transmural care" OR "whole system thinking" OR "holistic care")

#7 ("Angola " OR "Benin " OR "Botswana " OR "Burkina Faso" OR "Upper Volta" OR "Burundi " OR "Cameroon " OR "Cameroons " OR "Cape Verde" OR "Central African Republic" OR Chad OR "Comoros " OR "Comoro Islands" OR "Comores" OR "Mayotte " OR "Congo " OR "Zaire " OR "Cote d'Ivoire" OR "Ivory Coast" OR "Democratic Republic of the Congo" OR "Djibouti " OR "French Somaliland" OR "Eritrea " OR "Ethiopia " OR "Gabon " OR "Gabonese Republic" OR "Gambia " OR "Ghana " OR "Gold Coast" OR "Guinea " OR "Kenya " OR "Lesotho" OR "Basutoland" OR "Liberia " OR "Madagascar " OR "Malagasy Republic " OR "Malawi " OR "Nyasaland " OR "Mali " OR "Mauritania " OR "Mauritius " OR "Mozambique" OR "Namibia" OR "Niger" OR "Nigeria" OR "Rwanda" OR "Sao Tome" OR "Seychelles" OR "Senegal " OR "Sierra Leone" OR "Somalia " OR "South Africa" OR "Sudan " OR "Eswatini" OR "Tanzania" OR "Togo " OR "Togolese Republic" OR "Uganda" OR "Zambia" OR "Zimbabwe " OR "Rhodesia" OR "Sub-Saharan Africa" OR "Africa South of the Sahara")

#8 ((S1 OR S2 OR S3 OR S4 OR S5) AND S6 AND S7) & 1999-2022 & research article& peer-reviewed

For peer review only - http://bmjopen.bmj.com/site/about/guidelines.xhtml

Online supplementary file 3: Sensitivity analysis of RCTs and cluster RCTs

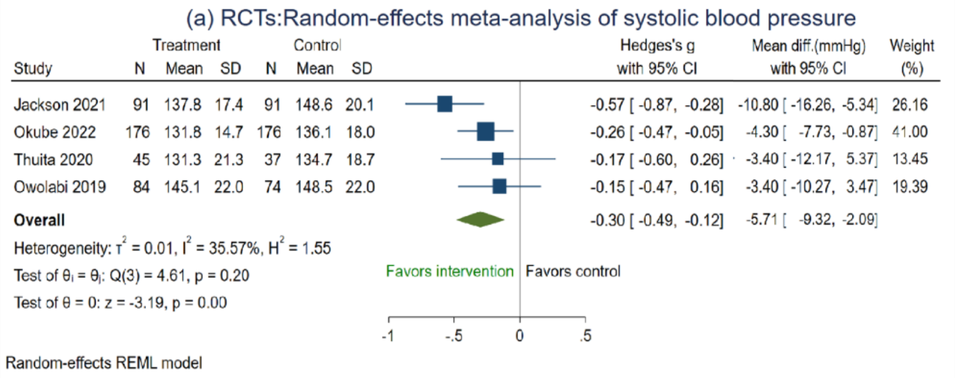

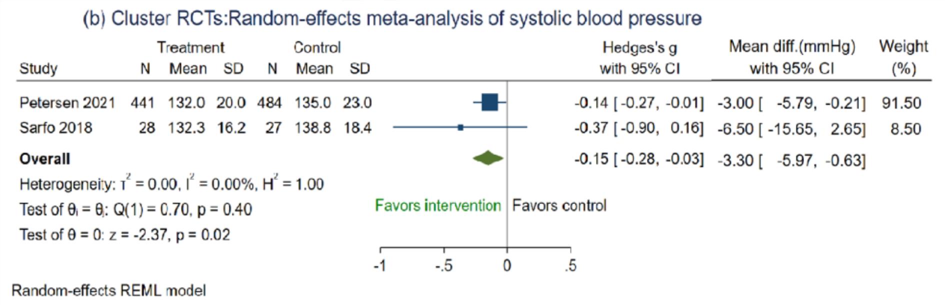

For peer review only - http://bmjopen.bmj.com/site/about/guidelines.xhtml

Online supplementary file 4: DOI plot of the random effects meta-analysis of SBP for integrated versus standard care for cardiometabolic multimorbidity in Sub-Saharan Africa

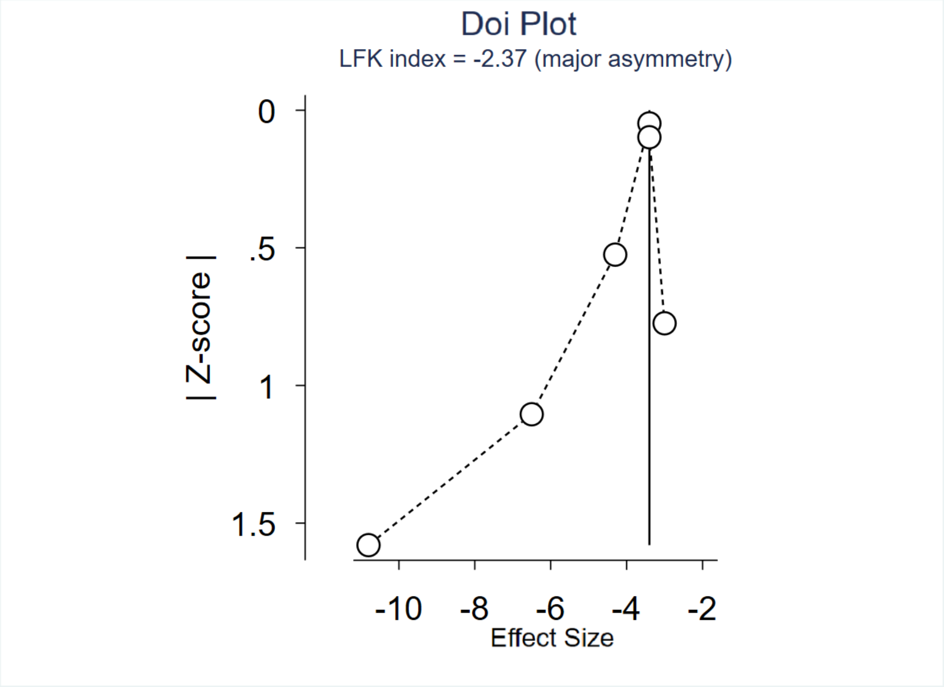

review only

For peer review only - http://bmjopen.bmj.com/site/about/guidelines.xhtml

PRISMA 2020 Checklist

Section and Topic	Item #	Checklist item	Location (Page) where item is reported
**TITLE : Effectiveness of integrated chronic care models for cardiometabolic multimorbidity in Sub-Saharan Africa: A systematic review and meta-analysis**			
Title	1	Identify the report as a systematic review.	1
**ABSTRACT**			
Abstract	2	See the PRISMA 2020 for Abstracts checklist.	2
**INTRODUCTION**			
Rationale	3	Describe the rationale for the review in the context of existing knowledge.	4
Objectives	4	Provide an explicit statement of the objective(s) or question(s) the review addresses.	5
**METHODS**			
Eligibility criteria	5	Specify the inclusion and exclusion criteria for the review and how studies were grouped for the syntheses.	7
Information sources	6	Specify all databases, registers, websites, organisations, reference lists and other sources searched or consulted to identify studies. Specify the date when each source was last searched or consulted.	7
Search strategy	7	Present the full search strategies for all databases, registers and websites, including any filters and limits used.	7
Selection process	8	Specify the methods used to decide whether a study met the inclusion criteria of the review, including how many reviewers screened each record and each report retrieved, whether they worked independently, and if applicable, details of automation tools used in the process.	8
Data collection process	9	Specify the methods used to collect data from reports, including how many reviewers collected data from each report, whether they worked independently, any processes for obtaining or confirming data from study investigators, and if applicable, details of automation tools used in the process.	8
Data items	10a	List and define all outcomes for which data were sought. Specify whether all results that were compatible with each outcome domain in each study were sought (e.g. for all measures, time points, analyses), and if not, the methods used to decide which results to collect.	7
	10b	List and define all other variables for which data were sought (e.g. participant and intervention characteristics, funding sources). Describe any assumptions made about any missing or unclear information.	7
Study risk of bias assessment	11	Specify the methods used to assess risk of bias in the included studies, including details of the tool(s) used, how many reviewers assessed each study and whether they worked independently, and if applicable, details of automation tools used in the process.	8
Effect measures	12	Specify for each outcome the effect measure(s) (e.g. risk ratio, mean difference) used in the synthesis or presentation of results.	9
Synthesis methods	13a	Describe the processes used to decide which studies were eligible for each synthesis (e.g. tabulating the study intervention characteristics and comparing against the planned groups for each synthesis (item #5)).	9
	13b	Describe any methods required to prepare the data for presentation or synthesis, such as handling of missing summary statistics, or data conversions.	9
	13c	Describe any methods used to tabulate or visually display results of individual studies and syntheses.	9
	13d	Describe any methods used to synthesize results and provide a rationale for the choice(s). If meta-analysis was performed, describe the model(s), method(s) to identify the presence and extent of statistical heterogeneity, and software package(s) used.	9
	13e	Describe any methods used to explore possible causes of heterogeneity among study results (e.g. subgroup analysis, meta-regression).	N/A
	13f	Describe any sensitivity analyses conducted to assess robustness of the synthesized results.	9
Reporting bias	14	Describe any methods used to assess risk of bias due to missing results in a synthesis (arising from reporting biases).	9

For peer review only - http://bmjopen.bmj.com/site/about/guidelines.xhtml

PRISMA 2020 Checklist

Section and Topic	Item #	Checklist item	Location (Page) where item is reported
assessment			
Certainty assessment	15	Describe any methods used to assess certainty (or confidence) in the body of evidence for an outcome.	9
**RESULTS**			
Study selection	16a	Describe the results of the search and selection process, from the number of records identified in the search to the number of studies included in the review, ideally using a flow diagram.	10
	16b	Cite studies that might appear to meet the inclusion criteria, but which were excluded, and explain why they were excluded.	10
Study characteristics	17	Cite each included study and present its characteristics.	10
Risk of bias in studies	18	Present assessments of risk of bias for each included study.	12
Results of individual studies	19	For all outcomes, present, for each study: (a) summary statistics for each group (where appropriate) and (b) an effect estimate and its precision (e.g. confidence/credible interval), ideally using structured tables or plots.	14
Results of syntheses	20a	For each synthesis, briefly summarise the characteristics and risk of bias among contributing studies.	12
	20b	Present results of all statistical syntheses conducted. If meta-analysis was done, present for each the summary estimate and its precision (e.g. confidence/credible interval) and measures of statistical heterogeneity. If comparing groups, describe the direction of the effect.	14
	20c	Present results of all investigations of possible causes of heterogeneity among study results.	14
	20d	Present results of all sensitivity analyses conducted to assess the robustness of the synthesized results.	14
Reporting biases	21	Present assessments of risk of bias due to missing results (arising from reporting biases) for each synthesis assessed.	14
Certainty of evidence	22	Present assessments of certainty (or confidence) in the body of evidence for each outcome assessed.	14
**DISCUSSION**			
Discussion	23a	Provide a general interpretation of the results in the context of other evidence.	15-16
	23b	Discuss any limitations of the evidence included in the review.	16
	23c	Discuss any limitations of the review processes used.	16
	23d	Discuss implications of the results for practice, policy, and future research.	17
**OTHER INFORMATION**			
Registration and protocol	24a	Provide registration information for the review, including register name and registration number, or state that the review was not registered.	5
	24b	Indicate where the review protocol can be accessed, or state that a protocol was not prepared.	The protocol was not published
	24c	Describe and explain any amendments to information provided at registration or in the protocol.	N/A
Support	25	Describe sources of financial or non-financial support for the review, and the role of the funders or sponsors in the review.	17
Competing interests	26	Declare any competing interests of review authors.	17-18

For peer review only - http://bmjopen.bmj.com/site/about/guidelines.xhtml

PRISMA 2020 Checklist

Section and Topic	Item #	Checklist item	Location (Page) where item is reported
Availability of data, code and other materials	27	Report which of the following are publicly available and where they can be found: template data collection forms; data extracted from included studies; data used for all analyses; analytic code; any other materials used in the review.	17-18

From: Page MJ, McKenzie JE, Bossuyt PM, Boutron I, Hoffmann TC, Mulrow CD, et al. The PRISMA 2020 statement: an updated guideline for reporting systematic reviews. BMJ 2021;372:n71. doi: 10.1136/bmj.n71

For more information, visit: http://www.prisma-statement.org/

For peer review only

BMJ Open

Effectiveness of integrated chronic care models for cardiometabolic multimorbidity in Sub-Saharan Africa: A systematic review and meta-analysis

Journal:	*BMJ Open*
Manuscript ID	bmjopen-2023-073652.R2
Article Type:	Original research
Date Submitted by the Author:	08-Jun-2023

Complete List of Authors:	Otieno, Peter; African Population and Health Research Center, Chronic Disease Management Uninit; Amsterdam UMC Locatie AMC, Department of Public & Occupational Health<br>Agyemang, Charles ; Amsterdam UMC Locatie AMC, Department of Public & Occupational Health<br>Wao, Hesborn; African Population and Health Research Center, Research and Related Capacity Strengthening<br>Wambiya, Elvis; The University of Sheffield, School of Health and Related Research<br>Ng’oda, Maurine; African Population and Health Research Center<br>Mwanga, Daniel; African Population and Health Research Center<br>Oguta, James; The University of Sheffield, School of Health and Related Research<br>Kibe , Peter M. ; African Population and Health Research Center<br>Asiki, G; African Population and Health Research Center, Chronic Disease Management Unit; Karolinska Institutet, Women's and Children's Health

**Primary Subject Heading**:	Cardiovascular medicine
Secondary Subject Heading:	Cardiovascular medicine
Keywords:	Hypertension < CARDIOLOGY, Patient-Centered Care, Primary Health Care, Self Care

SCHOLARONE™
Manuscripts

I, the Submitting Author has the right to grant and does grant on behalf of all authors of the Work (as defined in the below author licence), an exclusive licence and/or a non-exclusive licence for contributions from authors who are: i) UK Crown employees; ii) where BMJ has agreed a CC-BY licence shall apply, and/or iii) in accordance with the terms applicable for US Federal Government officers or employees acting as part of their official duties; on a worldwide, perpetual, irrevocable, royalty-free basis to BMJ Publishing Group Ltd ("BMJ") its licensees and where the relevant Journal is co-owned by BMJ to the co-owners of the Journal, to publish the Work in this journal and any other BMJ products and to exploit all rights, as set out in our licence.

The Submitting Author accepts and understands that any supply made under these terms is made by BMJ to the Submitting Author unless you are acting as an employee on behalf of your employer or a postgraduate student of an affiliated institution which is paying any applicable article publishing charge ("APC") for Open Access articles. Where the Submitting Author wishes to make the Work available on an Open Access basis (and intends to pay the relevant APC), the terms of reuse of such Open Access shall be governed by a Creative Commons licence – details of these licences and which Creative Commons licence will apply to this Work are set out in our licence referred to above.

Other than as permitted in any relevant BMJ Author's Self Archiving Policies, I confirm this Work has not been accepted for publication elsewhere, is not being considered for publication elsewhere and does not duplicate material already published. I confirm all authors consent to publication of this Work and authorise the granting of this licence.

For peer review only

Effectiveness of integrated chronic care models for cardiometabolic multimorbidity in Sub-Saharan Africa: A systematic review and meta-analysis

Peter Otieno*[1,2,3,6]
Charles Agyemang[2]
Hesborn Wao[1]
Elvis Wambiya[5 6]
Maurine Ng'oda[1]
Daniel Mwanga[1]
James Oguta[5 6]
Peter Kibe[1. 6]
Gershim Asiki[1,4]

1 African Population and Health Research Center P.O. Box: 10787-00100, Nairobi, Kenya.
2 Department of Public & Occupational Health, Amsterdam UMC, University of Amsterdam, Amsterdam Public Health Research Institute, Amsterdam, The Netherlands.
3 Amsterdam Institute for Global Health and Development (AIGHD), AHTC, Tower C4, Paasheuvelweg 25, 1105 BP Amsterdam, Netherlands.
4 Department of Women's and Children's Health, Karolinska Institutet, Stockholm, Sweden
5 School of Health and Related Research, University of Sheffield. Sheffield, S1 4DA, United Kingdom
6 African Network of Research Scientists, Nairobi, Kenya

* Corresponding author:
Peter Otieno
African Population and Health Research Center,
P.O. Box: 10787-00100, Nairobi, Kenya.
Email: pootienoh@gmail.com
Telephone: +254 (20) 400 1000

Word count: 4, 633

For peer review only

Abstract

Objectives: This review aimed at identifying the elements of integrated care models for cardiometabolic multimorbidity in Sub-Saharan Africa (SSA) and their effects on clinical or mental health outcomes including systolic blood pressure (SBP), blood sugar, depression scores and other patient-reported outcomes such as quality of life, and medication adherence.

Design: Systematic review and meta-analysis using the Grading of Recommendation, Assessment, Development, and Evaluation (GRADE) approach.

Data sources: We systematically searched PubMed, Embase, Scopus, Web of Science, Global Health CINAHL, African Journals Online, Informit, PsycINFO, clinicaltrials.gov, Pan African Clinical Trials Registry and grey literature from OpenSIGLE for studies published between 1999 and 2022.

Eligibility criteria for selecting studies: We included randomized controlled trial studies featuring integrated care models with two or more elements of Wagner's chronic care model.

Data extraction and synthesis: Two independent reviewers used standardised methods to search and screen included studies. Publication bias was assessed using the Doi plot and LFK index. Meta-analysis was conducted using random effects models.

Results: In all, we included 10 randomized controlled trials from 11 publications with 4,864 participants from seven SSA countries (South Africa, Kenya, Nigeria, Eswatini, Ghana and Uganda). The overall quality of evidence based on GRADE criteria was moderate. A random-effects meta-analysis of six studies involving 1,754 participants shows that integrated compared to standard care conferred a moderately lower mean SBP (Mean Difference=-4.85mmHg, 95% CI: -7.37 to -2.34) for people with cardiometabolic multimorbidity; Hedges' g effect size (g=-0.25, (-0.39 to -0.11). However, integrated care compared with usual care showed mixed results for HbA1c, depression, medication adherence, and quality of life.

Conclusion: Integrated care improved SBP among patients living with cardiometabolic multimorbidity in SSA. More studies on integrated care are required to improve the evidence pool on chronic care models for multimorbidity in SSA. These include implementation studies and cost-effectiveness studies.

Keywords: Integrated care, chronic care models, multimorbidity, cardiometabolic diseases, Sub-Saharan Africa

PROSPERO registration number: CRD42020187756

For peer review only - http://bmjopen.bmj.com/site/about/guidelines.xhtml

Article Summary:

Strengths and limitations of this study

- The inclusion of randomized controlled trials provides more unbiased findings than previous reviews that are mostly based on observational study designs.
- The extraction of important constructs of Wagner's chronic care model from the integrated care models offers deeper insights into the effectiveness of integrated care model elements.
- The review considered both communicable and non-communicable diseases which reflects their converging burdens in Sub-Saharan Africa.
- The varying numbers and types of chronic care model components resulted in heterogeneity in the included studies.
- Since most included studies used multi-component interventions, the absolute effect attributable to a particular element of the chronic care model remains unknown.

For peer review only - http://bmjopen.bmj.com/site/about/guidelines.xhtml
For peer review only

Background

Cardiometabolic diseases, including type 2 diabetes and cardiovascular diseases (CVDs), are the leading cause of global mortality (1). People living with cardiometabolic diseases often have multiple rather than a single condition, commonly known as multimorbidity (2). Cardiometabolic multimorbidity occurs against the background of obesity and insulin resistance (3). However, these conditions are also characterised by the simultaneous co-occurrence of other diseases with unrelated pathophysiology or care management profile such as HIV/AIDS, chronic respiratory diseases, cancers, and mental illnesses, a phenomenon referred to as discordant multimorbidity (4-6).

The burden of multimorbidity is highest in low- and middle-income countries (LMICs), which still bear a high burden of infectious diseases while also facing a new threat from non-communicable diseases (7). However, the majority of the chronic care models in Sub-Saharan Africa (SSA) are still built around single disease frameworks (8). Evidence shows that health services delivery based on isolated interventions may derail the value of treatment and preventive measures (9-12). Vertical disease programs result in missed opportunities for early diagnosis and management of multimorbidities (13). Therefore, integrated chronic disease management is recommended to respond to this challenge (14). Singer et al, (2011) (15), define *integrated care* as a set of patient-centred and multidisciplinary care activities coordinated by two or more collaborating service providers within or across the healthcare sector including community and social environments. This definition emphasizes the coordination of care activities and the active involvement of patients in managing their health.

The chronic care model, first identified by McColl Institute for Healthcare Innovation at Group Health Cooperative (11), has been proposed as a solution to improve the integrated management of chronic diseases. This model identifies six essential elements: community resources and policies, health care organization, self-management support, delivery system design, decision support and clinical information systems. However, what constitutes an integrated chronic care model and how it is implemented and delivered within healthcare services, has continued to evolve with the advent of new interventions. Most of the chronic care models have been developed in high-income countries with sophisticated health services (16), which may not appropriately apply to SSA.

The untapped capacity of chronic care models for HIV/AIDS care in SSA should be viewed as emerging models for other chronic diseases including cardiometabolic diseases (17). Lessons learned from the implementation of care models for HIV/AIDS and tuberculosis in SSA could be effectively applied to improve care models for cardiometabolic diseases (18). A systematic review by Rohwer et al (19) examined

For peer review only - http://bmjopen.bmj.com/site/about/guidelines.xhtml

existing integrated care models for diabetes and hypertension in LMICs. However, the included studies did not focus on structured clinical care delivered to persons living with multimorbidity. Furthermore, the integrated care for hypertension and diabetes was based on a 'one-stop-shop' model where all healthcare services are provided under one roof (19). This is just one way of describing integrated care. A comprehensive evaluation of the applicability of the elements of chronic care models for cardiometabolic multimorbidity has not been systematically evaluated in SSA. Therefore, the purpose of this systematic review was to identify elements of integrated chronic care models for cardiometabolic multimorbidity in SSA and their effects on clinical or mental health outcomes including systolic blood pressure (SBP), blood sugar, depression scores and other patient-reported outcomes such as quality of life, and medication adherence.

Methods

Protocol registration

The study protocol was registered in the International Prospective Register for Systematic Reviews (PROSPERO) (CRD42020187756). The findings are reported according to the Preferred Reporting Items for Systematic Reviews (PRISMA) (20).

Eligibility criteria

Participants

We selected studies conducted in SSA among adult patients aged 18 years and over with cardiometabolic multimorbidity receiving care in a primary or community care setting. Cardiometabolic multimorbidity was defined as having two or more chronic conditions; at least one of which is a cardiometabolic disease such as type 2 diabetes, hypertension, hypercholesterolemia, hypertriglyceridemia, dyslipidaemia or CVDs such as stroke. Others included common discordant conditions such as HIV/AIDS, chronic respiratory diseases, cancers and mental illnesses. These conditions constitute over three-quarters of the global burden of chronic diseases (21).

Intervention

We included interventions with integrated chronic care models for persons living with cardiometabolic multimorbidity. In line with previous studies (22-26), we classified the intervention components into six categories based on the elements of Wagner's chronic care model (11). Thus, for the inclusion criterion, the models were considered "integrated care" if they applied at least two of the six chronic care model elements to

For peer review only

manage more than one chronic disease (23). This included self-management support, delivery system design, decision support, clinical information system, healthcare organization, and community linkages.

The classification of intervention features and components is shown in Supplementary File 1. Self-management support includes interventions that empower people living with chronic conditions to manage their health. Examples are self-help groups and technological aids for self-care. Delivery system design interventions focus on care delivery efficiency and effectiveness such as patient care planning, coordination, and follow-up. Decision-support interventions promote clinical care that is consistent with scientific evidence and patient preferences. Clinical information systems is the organization of patient data to facilitate efficient and effective care. Healthcare organization interventions focus on creating an organizational culture and mechanisms that promote safe, high-quality care. Community linkages include interventions that mobilize community resources to meet the needs of people living with chronic diseases. Where the chronic care model had multiple components, we defined each element using the Wagner taxonomy (11) and highlighted the predominant components.

Control

The control or comparison group in the included studies received the usual or standard care comprising standalone services for cardiometabolic multimorbidity.

Outcome

We included studies that reported any objective measure of patient clinical or mental health outcomes. For example, SBP (mmHg), glycated haemoglobin ((HbA1c (%)), and depression scores. Others included patient-reported outcomes such as quality of life, and patient behaviour (medication adherence).

Types of studies

We included randomised controlled trials (RCTs) meeting the quality criteria developed by the Cochrane Effective Practice and Organization of Care (EPOC) (27). This is because RCTs provide more unbiased information than other study designs on the differential effects of chronic care models. Studies from SSA published in all lang.uages between 1999 and December 2022 were included. We used 1999 because this was the formal year of inception of the Wagner's chronic care model (28).

For peer review only - http://bmjopen.bmj.com/site/about/guidelines.xhtml

Exclusion criteria

We excluded single-disease-focused studies and studies conducted among pregnant women. Interventions with no specified structured clinical care delivered to persons living with cardiometabolic multimorbidity were excluded. Studies with interventions for comorbid conditions where the intervention was targeted solely at one of the conditions rather than integrated care for the co-occurring conditions were also excluded. Studies that assumed multimorbidity to be the norm based on individuals' age without screening and diagnosis by qualified healthcare providers were excluded as the interventions were not being targeted specifically at multimorbidity.

Search strategy

Two reviewers independently searched PubMed, Embase, Scopus, Web of Science, Cumulative Index to Nursing and Allied Health Literature (CINAHL), Cochrane library, African Journals Online, Informit, and PsycINFO without language restriction. The search was limited to articles indexed from 1999 up to 15th December 2022 to capture the beginning of the application of the elements of Wagner's chronic care model. The search results were limited by filters for randomized control trials and search concepts for chronic care models, cardiometabolic diseases and multimorbidity (See supplementary file 2). Additional articles were identified by scanning the reference list of relevant studies obtained through the search. Hand-searching of key journals was also conducted. Furthermore, we searched for registered trials in the clinicaltrials.gov, the Pan African Clinical Trials Registry and grey literature from OpenSIGLE.

Data collection and analysis

Selection of studies

The search results were uploaded into the Endnote version 12 reference manager (Clarivate, Philadelphia, USA) and screened for duplicates. Four review authors (EW, MN, PK and DM) independently performed the initial screening of titles and abstracts. Results of screening were recorded against the citation in Excel spreadsheet. The articles selected for full-text review were subsequently assessed for adequacy following the inclusion criteria for patient, intervention, comparator and outcome. Consensus meetings were held between the reviewers and the project coordinator (PO) to resolve disagreements. We used a modified PRISMA flow chart to describe the study selection (Figure 1).

Data extraction and management

Four review authors (EW, MN, PK and DM) abstracted data using a modified version of the EPOC data collection checklist (27). Data were entered in Excel spreadsheets and exported to Stata version 15 (StataCorp LLC). Data quality disagreements were resolved by consensus between the review authors and the project coordinator (PO). Information extracted from the included studies comprised:

1. Participants: type of patients, age, nature of multimorbidity and how it was diagnosed.

For peer review only - http://bmjopen.bmj.com/site/about/guidelines.xhtml

203 2. Study design: randomization, the unit of allocation, and follow-up.

204 3. Intervention: we extracted a full description of the intervention components and features. This
205 included self-management support, delivery system design, decision support, clinical information
206 system, healthcare organization, and community resources

207 4. Service providers: specialists, primary care providers, and family members

208 5. Study setting: primary care and community-based care

209 6. Patient clinical or mental health outcomes such as SBP, blood glucose control, and depression
210 symptom scores. Others included patient-reported outcomes such as quality of life and medication
211 adherence.

212 7. Results: We organized results into clinical outcomes, mental health outcomes and patient-reported
213 outcomes.

214 **Assessment of risk of bias**

215 Four review authors (EA, MN, PK and DM) independently assessed the risk of bias in the included studies
216 using standard EPOC criteria (29). The assessment domains included: allocation (sequence generation and
217 concealment); baseline characteristics; incomplete outcome data; contamination; blinding; selective
218 outcome reporting; and other potential sources of bias. The criteria for the assessment were 'low risk', 'high
219 risk' or 'unclear risk' (Figure 2). Loss to follow-up of more than 20% was considered a high risk of bias.
220 We also considered loss to follow-up as a high risk of bias when it was unbalanced between the groups.
221 Published protocols and trial registrations were tracked to assess selective reporting bias. We assessed
222 incomplete reporting for studies that reported different results than the outcomes outlined in the methods
223 sections of selected articles. Any disagreements between the review authors were resolved by consensus, or
224 with the consultation of the project coordinator (PO).

225 **Data analysis**

226 We used a random-effects meta-analysis to estimate the pooled mean effect size of integrated care versus
227 standard care on SBP. The random-effects meta-analysis allows for statistical heterogeneity between studies
228 by assuming that the true effects in the individual studies differ from each other. Six studies with similarities
229 in terms of the patient population, interventions, and outcome assessment, were included in the meta-
230 analysis. Studies with significant heterogeneity were not included in the meta-analysis. Hence, we
231 conducted a structured synthesis of the results. All analyses were performed using *"metan"* command in
232 Stata version 15 (StataCorp LLC).

233

For peer review only - http://bmjopen.bmj.com/site/about/guidelines.xhtml

We calculated the effect size following the methods described by Hedges (30). For each of the included studies, we calculated results in terms of absolute and standardized mean differences with a 95% Confidence Interval (CI). The heterogeneity of the selected studies were evaluated using χ^2 and I^2 statistics (31). Using the χ^2 test, heterogeneity between studies was considered significant if $p<0.10$ (32). We used I^2 statistics, values of 25%-49%, 50%-74% and >75% to determine low, moderate and high heterogeneity respectively (33). Publication bias was assessed using the Doi plot and Luis Furuya Kanamori (LFK) index (34). The LFK index outside the interval −1 and 1 was considered consistent with asymmetry (35). As Higgins et al. (36) posit, it is justifiable to perform a pooled meta-analysis involving cluster RCTs and individual RCTs. However, sensitivity analyses should be performed to test the robustness of the pooled estimates. (36, 37). We performed sensitivity analyses to assess the robustness of our results by comparing the pooled effect estimates from separate analyses involving RCTs (four studies) and cluster RCTs (two studies).

Certainty of evidence

The certainty of the evidence of included studies was assessed using the Grading of Recommendations Assessment, Development and Evaluation (GRADE) methodological guideline. According to GRADE guidance, RCTs start with high-certainty evidence (38). Our judgements to downgrade the certainty of evidence were based on the assessment of the following five domains: study limitations, inconsistency, imprecision, indirectness and publication bias. For each outcome, we described the certainty of evidence as high, moderate, low or very low. The GRADE criteria also take into consideration the certainty of evidence and the size of the effect (39). We considered a 5 mmHg reduction in SBP to be clinically significant (40). A change of 0.5% for the outcome HbA1c, and 10% for depression scores, medication adherence and quality of life were considered clinically significant (41). For each outcome, we described the certainty of evidence as high, moderate, low or very low (42).

Patient and public involvement

Patients or the public were not involved in the design, conduct, reporting, or dissemination of this research.

For peer review only - http://bmjopen.bmj.com/site/about/guidelines.xhtml

Results

Search results

The PRISMA flow diagram of the article selection process is presented in Figure 1. We identified 2,649 studies from our searches. Another 17 studies were included after manually searching the reference list and grey literature. After the removal of 806 duplicate records, we screened the titles and abstracts of 1,860 articles and identified 32 potentially eligible studies. We reviewed the full text of 32 studies and excluded 22 studies. Thus, 10 studies from 11 publications were included in this review (6 randomized controlled trials and 4 cluster randomized trials). Of these 10 studies, 6 were included in the meta-analyses and 4 studies from 5 publications were included in the structured synthesis of the results.

[Figure 1 approximately here]

Characteristics of included studies

The characteristics of the included studies are presented in Table 1. We included 10 trials from 11 publications (43-53) with a total of 4,864 participants. Sample sizes ranged from 55 to 1,174 participants. Three studies were conducted in South Africa, two in Kenya and Nigeria and one in Eswatini and Ghana, respectively. There was one multi-country study conducted in Uganda and Kenya. Studies mostly took place in primary care settings (n=9) or both community and primary care settings (n=2). Most trials recruited participants with HIV and cardiometabolic diseases (n=9), another two recruited patients with HIV, type 2 diabetes, hypertension, depression and substance use disorder. Eight studies lasted 12 months or more. In all the included studies, the control group received the usual standard care while the intervention group received structured integrated care with varying elements of chronic care models including self-management support, delivery system design, decision support, clinical information system, healthcare organization, and community resources.

For peer review only - http://bmjopen.bmj.com/site/about/guidelines.xhtml

For peer review only

Table 1: Characteristics of included studies

Study	Setting	Study design	Sample size	Age, years	Multimorbidity	Follow-up (months)	Outcome	Intervention
Havlir et al (2019) (43) Uganda and Kenya	Primary and community-based care	Cluster RCT	1,441 patients with hypertension & HIV	≥30	Hypertension and HIV	36	BP control	Multi-disease testing; integrated care for HIV and hypertension; structured follow-up, flexible hours of operation and reduced wait time at clinics; and patient incentives.
Jackson et al (2021) (44) Nigeria	Primary care	RCT	182	18-69	Hypertension & HIV	12	SBP and medication adherence	Pharmaceutical care with structured education and counselling by a pharmacist after seeing a physician on their clinic visit. Education focused on self-monitoring of BP, lifestyle modification, reviewing the date of the next appointment and prescription.
Jackson et al (2022) (45) Nigeria	Primary care	RCT	182	18-69	Hypertension and HIV	12	Health-related quality of life	Structured education and counselling on general self-care, medicines use storage and lifestyle modifications.
Myers et al (2022) (46) South Africa	Primary care	Cluster RCT	1,174	≥18	HIV, Type 2 diabetes, depression and substance use disorder	12	Depression scores	Psychological interventions comprising motivational interviewing and problem-solving therapy delivered by a trained facility-based community health worker.
Okube et al (2022) (47) Kenya	Community-based care	RCT	294	18-64	Metabolic syndrome	12	BP and blood sugar control	Community-based health education on lifestyle modification and face-to-face delivery of verbal and written individualized health recommendations on risk factors for CVDs.
Owolabi et al (2019) (48) Nigeria	Primary care	RCT	158	≥18	Hypertension and stroke	12	SBP	A culturally appropriate, multipronged intervention comprising patient global risk factor control report card, personalized phone text-messaging, and educational video.
Petersen et al (2021) (49) South Africa	Primary care	Cluster RCT	925	≥18	Hypertension and depression	12	Depression scores	Supplementary training of primary nurses and doctors on mental health and clinical communication skills. Collaborative care model for patients with hypertension and comorbid depressive symptoms including doctors, nurses, clinical psychologists and lay counsellors.
Rabkin et al (2018) (50) Eswatini	Primary care	RCT	236	≥40	HIV, Hypertension, type 2 diabetes, and hyperlipidemia	6	SBP and HbA1c	CVD risk factors screening and structured referrals among patients living with HIV.
Roos et al (2014) (51) South Africa	Primary care	RCT	84	20-65	HIV and metabolic diseases	12	SBP and fasting blood sugar	Pedometer and a physical activity diary with education materials and self-monitoring documents. Structured regular clinic sessions for review of physical activity diary and risk factors for ischemic heart disease. Monthly SMS text motivational messages.
Sarfo et al (2018) (52) Ghana	Primary care	Cluster RCT	55	≥18	Hypertension and stroke	9	BP control and medication adherence	Blue-toothed BP device and smartphone for self-monitoring and reporting BP measurements and medication intake. Tailored motivational text messages delivered based on the levels of adherence to the medication intake protocol.
Thuita et al (2020) Kenya (53)	Primary care	RCT	133	20–79	Metabolic syndrome	6	SBP and HbA1c	Integrated care with nutrition education and peer-to-peer support

*The control group received standard/usual care

For peer review only - http://bmjopen.bmj.com/site/about/guidelines.xhtml

Risk of bias in included studies

The risk of bias summary is presented in Figure 2. All the included studies were RCTs. Hence, all studies had low risk for randomization. Four of the 11 publications had a cluster design that ensured no contamination of control participants (43, 46, 49, 52). The four cluster-randomized controlled trials accounted for clustering effects in their analysis so there was no unit of analysis errors (43, 46, 49, 52). Six studies were appraised as unclear risk due to insufficient information about protection against contamination (44, 47, 48, 50, 51, 53). One study was rated as unclear risk due to lack of sufficient information about allocation concealment. Although it was not feasible to blind the participants and study personnel due to the nature of the interventions, the absence of blinding did not affect the objective outcomes. Hence, all studies were graded as low risk for blinding. Under the reliability of primary outcomes, five studies were rated as high risk due to small sample sizes (48, 51-53) and lack of baseline measurements (43). For selective reporting, all the studies were assessed as low risk, except one that lacked clarity and was assessed as unclear risk (50). Four studies were rated high risk for other biases, mainly short follow-up duration (less than one year) (48, 50, 52, 53). Overall, the certainties of the evidence for SBP, HbA1c, depression, medication adherence and quality of life were downgraded to moderate. This was due to the high risk of bias, imprecision and clinically insignificant effect sizes in the included studies (43-53).

[Figure 2 approximately here]

Components of the chronic care model

Table 2 presents the components of the chronic care model in the included studies. All studies had to include at least two components of the Wagner chronic care model to be defined as "integrated care" for the management of at least two chronic diseases. The number of chronic care elements ranged from two to five. One study included five of the six elements of the chronic care model (43). Three studies included three elements (48, 51, 52) and six enclosed two elements (46, 49, 50, 53-55). All the studies included delivery system design except one. The components of self-delivery included multi-disease screening and treatment, task shifting, structured follow-up, and collaborative care. Seven studies included self-management support such as home-based self-monitoring, lifestyle counselling, post-clinic patient follow-up and patient support groups (43, 44, 48, 51-53, 55). Four studies included decision support components such as supplementary training of healthcare workers and support supervision (43, 46, 49, 50). Three studies included clinical information systems (48, 51, 52). The elements enclosed comprised hospital registries, patient diaries, report cards and electronic platforms for BP monitoring. Two studies included community resource elements to enhance health campaigns (43, 55). One study included the healthcare organization element, mainly quality improvement strategies to enhance access to medication and treatment services (43).

For peer review only - http://bmjopen.bmj.com/site/about/guidelines.xhtml

Table 2: Elements of the chronic care model included in the studies

Author (year)	Self-management support	Delivery system design	Decision support	Clinical information system	Healthcare organization	Community resources
Havlir et al (2019) (43)	Enhanced lifestyle modification counselling in primary care centers	Point-of-care multi-disease screening for HIV, hypertension, and diabetes with structured follow-up and care linkages	Telephone and in-person oversight from a senior physician on the services provided by general physicians and nurses.		Quality improvement strategies such as guaranteed access to medication, flexible hours of operations, and reduced wait time at clinics	Multi-disease testing community health campaigns using community resource persons
Jackson et al (2021) (44)	Education on self-monitoring of BP, lifestyle modification, self-care and appropriate use of medicines	Structured pharmaceutical care including prescription review and follow-up				
Myers et al (2022) (45)		Task shifting and empowerment of community health workers to provide basic psychological interventions	Trained community health workers on basic psychological intervention including motivational interviewing and problem-solving therapy			
Okube et al (2022) (47)	Individualized health recommendations on CVD risk factors					Community-based health education on lifestyle modification
Owolabi et al 2019 (48)	Post-clinic follow-up phone texts and waiting room educational video	Enhanced follow-up visits and pre-appointment phone texts		Hospital registry and patient report card as part of medical chart		
Petersen et al (2021) (49)		Collaborative care model for patients with hypertension and comorbid depressive symptoms including doctors, nurses, clinical psychologist and lay counsellors	Supplementary training of primary care nurses and doctors on mental health and clinical communication skills			
Rabkin et al (2018) (50)	-	One stop shop for CVD risk factors screening and structured referrals among patients living with HIV	Training of HIV clinic nurses and doctors to conduct CVD risk factors screening during routine clinic appointments of patients			
Roos et al (2014) (51)	A pedometer and a physical activity diary with education materials and self-monitoring documents. Monthly SMS text motivational messages	Structured regular clinic sessions for review of physical activity diary and risk factors for ischemic heart disease		Patient diary for self-monitoring of risk factors for ischemic heart disease		
Sarfo et al (2018) (52)	Self-monitoring of BP using a blue-toothed device. Tailored motivational text messages delivered based on the levels of adherence to medication	Structured follow-up for BP measurements and medication adherence		Digital platform for tracking BP measurements and medication adherence		
Thuita et al 2020 (53)	Nutrition counselling and peer support group	Monthly follow up visits and structured regular clinic sessions to review patient progress. Facility-based patient support groups.				

.

For peer review only - http://bmjopen.bmj.com/site/about/guidelines.xhtml

Effectiveness of integrated models of care compared with standard care

Systolic blood pressure

Figure 3 presents the random effects meta-analysis of SBP. The pooled results of 6 studies (44, 47-49, 52, 53) involving 1,754 participants show that integrated versus standard care conferred a lower mean SBP (Mean Difference (MD) =-4.85mmHg, 95% CI (-7.37 to -2.34) with a small Hedges' g effect size (g=-0.25, (-0.39 to -0.11). However, the overall quality of evidence based on GRADE criteria was moderate. Moderate heterogeneity (I^2=39.57%) existed in the included studies (Figure 3). The results of the sensitivity analyses separating RCTs from cluster RCTs are consistent with those of the pooled analyses (Supplementary File 3). Integrated care reduced systolic blood pressure in RCTs by (MD=-5.71mmHg, 95% CI -9.32 to -2.09) and by (MD=-3.30mmHg, 95% CI -5.97 to –0.63) for the cluster RCTs. A Doi plot of the random effects meta-analysis of SBP for integrated versus standard care is shown in Supplementary File 4. The presence of publication bias is suggested by the asymmetrical visual inspection of the Doi plot, confirmed by LFK index=-2.37.

[Figure 3 approximately here]

We did not conduct meta-analyses for the four of the included studies in this review (43, 46, 50, 51), as they were too heterogeneous in terms of patient population, interventions, and outcome assessment. Hence, we conducted a structured synthesis of their results. Of the studies included in the structured synthesis of results, the effect of integrated versus standard care on BP were reported in three studies (43, 50, 51). However, the quality of evidence based on GRADE criteria was moderate. The SEARCH trial conducted in Kenya and Uganda found that hypertension control was 22% higher among patients on integrated care versus standard care (relative prevalence=1.22; (95%CI 1.08 to 1.37)) (43). In contrast, a study by Rabkin et al (50) conducted in Eswatini found no significant effect of integrated versus referred management of CVD risk factors on mean SBP. Another study by Roos et al (2014) also found no significant effect of education and a home-based pedometer walking program on SBP MD=2.50 mmHg, 95% CI (-2.78 to 7.78).

Fasting blood glucose and HbA1c

Two studies included in the structured synthesis of the results assessed the effect of integrated versus standard care on FBS and HbA1c (47, 50). A study by Okube et al (47) found that integrated care conferred a significant ($p < 0.05$) reduction in FBS by -0.5mmol/L versus +0.08 mmol/L among patients on standard care. In a study by Rabkin et al (50), the mean of HbA1c was significantly reduced by -0.7% (95%CI: -1.3 to -0.1) and -1.4% (-2.5 to -0.2) in the integrated and standard care arms, respectively with no statistical

For peer review only - http://bmjopen.bmj.com/site/about/guidelines.xhtml

difference between arms 0.7% (95%CI -0.4 to 1.8).

Depression

The effect of integrated versus standard care on depression was reported in two studies. A study by Petersen et al (49) found no significant difference in the depression scores in patients on integrated versus standard care (adjusted risk difference = -0.04 (95%CI, -0.19 to 0.11)). By contrast, the results of the study by Myers et al (46) found significant differences in the depression scores in patients on integrated versus standard care (MD = 4.8(95%CI -7.2 to -2.4)).

Medication adherence and quality of life

Two studies assessed the effect of integrated care on hypertension medication adherence (45, 50). One study reported the effect of integrated versus standard care HRQoL (45). In a study by Rabkin et al (50), integrated care conferred a higher likelihood of hypertension medication adherence than standard care (relative risk=1.28 (95%CI 1.10 to 1.47)). A study by Jackson et al (45) found that integrated care led to significant improvements in hypertension medication adherence (Mean Difference in Difference (DiD)= 2.32; $p < 0.001$) and HRQoL (DiD= 6.5%, $P < 0.001$). Two studies assessed the effect of integrated care on hypertension medication adherence (45, 50). One study reported the effect of integrated versus standard care on HRQoL (45). In a study by Rabkin et al (50), integrated care conferred a higher likelihood of hypertension medication adherence than standard care (relative risk=1.28 (95%CI 1.10 to 1.47)). A study by Jackson et al (45) found that integrated care led to significant improvements in hypertension medication adherence (Mean Difference in Difference (DiD)= 2.32; $p < 0.001$) and HRQoL (DiD= 6.5%, $P < 0.001$).

.

Discussions

This paper presents the results of a systematic review of integrated care models for cardiometabolic multimorbidity and their effects on intermediate health outcomes in SSA. Our results show that, in comparison to usual care, integrated care featuring at least two elements of Wagner's chronic care model conferred moderate improvements in SBP among patients living with cardiometabolic multimorbidity. The overall quality of evidence was moderate. Results of the systematic review suggest that integrated care compared with usual care has mixed results with regards to HbA1c, depression, medication adherence and quality of life, with some studies showing a significant effect and some no effect.

The findings of the current review based on RCTs are broadly consistent with reviews on the effectiveness of the integrated chronic care model from previous observational studies (56, 57). In contrast, one systematic review by Rohwer et al (19) on integrated care models for diabetes and hypertension in LMICs found no evidence of improved blood pressure or diabetes control. However, the evidence presented had

For peer review only - http://bmjopen.bmj.com/site/about/guidelines.xhtml

very low certainty and the included studies did not focus on care models for persons living with multimorbidity.

Our study used Wagner's chronic care model, a framework for improving the integrated management of chronic diseases through the implementation of its six core elements: community resources and policies, health care organization, self-management support, delivery system design, decision support and clinical information systems (11). A review by Goh et al (56) found greater improvements in HbA1c for care models with more elements over a single element. In the same vein, another systematic review by Ellisen et al [56] reported variations in glycemic control with an increase in the number of elements of the chronic care model. However, most studies included in the current review had two to three CCM elements which could partly explain the mixed results with regard to the effect of care integration on intermediate health outcomes such as HbA1c, depression, medication adherence and quality of life, with some studies showing a significant effect and some no effect.

In the current review, delivery system design and self-management support were the most predominant components of the integrated chronic care models while healthcare organization and community resource elements were less common. Nevertheless, the structured synthesis of the results shows no significant differences in health outcomes by the elements of care integration since a majority of studies included two to three elements. Previous studies have shown that intervention intensity including length of implementation and frequency of monitoring, rather than the number of intervention components may have an impact on the direction and magnitude of the health outcomes (57, 58).

Overall, our results have three main implications. First, our findings have great public health significance given that a 5 mmHg reduction of SBP could reduce the risk of major cardiovascular events by about 13% (59). Thus, incorporating integrated care into the implementation of Wagner's chronic care model may partly address the needs of people living with cardiometabolic multimorbidity. Second, this review provides crucial evidence on the applicability of chronic care models in SSA and unearths the components of chronic care models for people living with cardiometabolic multimorbidity. Lastly, most integrated care models sought to support self-management and delivery system design. However, a few included healthcare organizations and community resource elements. More interventions should seek to incorporate these elements to support integrated care.

For peer review only - http://bmjopen.bmj.com/site/about/guidelines.xhtml

Strengths and limitations

The current review has three main strengths. First, all the included studies were RCTs. Hence, the findings are more unbiased than previous reviews on integrated care in LMICs that are mostly based on observational study designs (19, 60-62). Second, this study comprehensively extracted important constructs of Wagner's chronic care model, thus offering deeper insights into the effectiveness of its elements and delivering crucial evidence to researchers and health stakeholders for future improvement of integrated care models. The consideration of both communicable and non-communicable diseases in the review is also a strength due to the converging burdens of infectious and non-communicable diseases in SSA (7).

The findings of this review should be interpreted cautiously due to a few limitations. First, the majority of the included studies did not classify the components of chronic care models in the interventions. Although the three reviewers independently classified the type of chronic care model elements in the studies using a standard guide, there is potential for misclassification bias. Second, the varying numbers and types of chronic care model components added to the heterogeneity. Furthermore, the absolute effect attributable to a particular element of the chronic care model remains unknown, as most included studies used multi-component interventions. Third, it may not be possible to have a standard of care that is similar for all the studies included in this review. This resulted in a broad variety of usual care. Lastly, due to the substantial heterogeneity in the studies, in terms of multimorbidity, interventions, follow-up periods, and outcome assessment, only six of the ten studies were included in the meta-analysis, which may have a bearing on the power of the meta-analysis findings. The rest of the studies were included in the structured synthesis of the results.

Conclusions

We found that integrated care may lead to moderate improvements in SBP among patients living with cardiometabolic multimorbidity in SSA. This review highlights the paucity of research on care integration interventions for cardiometabolic multimorbidity in SSA since the selected RCTs were from only a few SSA countries. In addition, there were fewer studies with outcomes on health-related quality of life, mental health, and medication adherence. The small number of studies addressing healthcare organisation and community resource elements provide less certainty on the benefits associated with these components. More studies are needed on the implementation effectiveness of integrated care models and its impact on cardiometabolic multimorbidity. The relative effectiveness of the different elements of integrated chronic care models and the cost-effectiveness of these models for the management of cardiometabolic multimorbidity should be explored. Other elements that are not well explored such as healthcare organisation and community resource elements should be investigated in future research.

For peer review only - http://bmjopen.bmj.com/site/about/guidelines.xhtml

Future studies investigating the effectiveness of integrated care should classify the elements in the interventions and standardize the description of each element.

Acknowledgements: Not applicable.

Author contributions: PO conceptualized the study, reviewed the literature, and analysed the data. All authors contributed to the development and review of the protocol. EW, MN, and DM screened titles and abstracts and participated in full-text screening; PO helped to resolve discrepancies. EW, MN, and DM extracted data and assessed the risk of bias. PO assessed the certainty of evidence with input from all the authors. PO, JO and EW performed the meta-analyses. CA, HW and GA provided overall methodological guidance. JO and PK critically read and revised the first draft of the manuscript. PO, EW, PK and JO responded to reviewers' comments and made subsequent revisions. All authors have approved the final version of the manuscript.

Funding: This systematic review was supported by funding from the Joep Lange Institute and in part by the Dutch Ministry of Foreign Affairs under the Joep Lange Chairs and Fellows Program. JO and EW are funded by Wellcome Trust [218462/Z/19/Z] doctoral training grant to the University of Sheffield. The funders had no role in the design of the study and collection, analysis, and interpretation of data and in writing the manuscript.

Disclaimer: The funder did not have any role in the review process.

Competing interests: None declared.

Patient consent for publication: Not required.

Ethics approval: This systematic review does not involve human participants. All data included are in the public domain and ethics approval was thus not sought.

Data availability statement: All data relevant to the study are included in the article or uploaded as online supplemental information.

For peer review only - http://bmjopen.bmj.com/site/about/guidelines.xhtml

References

1. Roth GA, Mensah GA, Johnson CO, Addolorato G, Ammirati E, Baddour LM, et al. Global burden of cardiovascular diseases and risk factors, 1990–2019: update from the GBD 2019 study. Journal of the American College of Cardiology. 2020;76(25):2982-3021.
2. Violan C, Foguet-Boreu Q, Flores-Mateo G, Salisbury C, Blom J, Freitag M, et al. Prevalence, determinants and patterns of multimorbidity in primary care: a systematic review of observational studies. PloS one. 2014;9(7).
3. Arenillas JF, Moro MaA, Dávalos A. The metabolic syndrome and stroke: potential treatment approaches. Stroke. 2007;38(7):2196-203.
4. Mannino DM, Thorn D, Swensen A, Holguin F. Prevalence and outcomes of diabetes, hypertension and cardiovascular disease in COPD. European Respiratory Journal. 2008;32(4):962-9.
5. Metra M, Zaca V, Parati G, Agostoni P, Bonadies M, Ciccone M, et al. Cardiovascular and noncardiovascular comorbidities in patients with chronic heart failure. Journal of Cardiovascular Medicine. 2011;12(2):76-84.
6. Scott KM. Depression, anxiety and incident cardiometabolic diseases. Current opinion in psychiatry. 2014;27(4):289-93.
7. Coates MM, Ezzati M, Robles Aguilar G, Kwan GF, Vigo D, Mocumbi AO, et al. Burden of disease among the world's poorest billion people: An expert-informed secondary analysis of Global Burden of Disease estimates. PLoS One. 2021;16(8):e0253073.
8. Guthrie B, Payne K, Alderson P, McMurdo ME, Mercer SW. Adapting clinical guidelines to take account of multimorbidity. Bmj. 2012;345:e6341.
9. Nolte E, McKee M. Caring for people with chronic conditions: a health system perspective: McGraw-Hill Education (UK); 2008.
10. Boult C, Reider L, Frey K, Leff B, Boyd CM, Wolff JL, et al. Early effects of "Guided Care" on the quality of health care for multimorbid older persons: a cluster-randomized controlled trial. J Gerontol A Biol Sci Med Sci. 2008;63(3):321-7.
11. Wagner EH. Chronic disease management: what will it take to improve care for chronic illness? Effective clinical practice. 1998;1(1).
12. World Health Organization. Towards people-centred health systems: An innovative approach for better health outcomes. 2013.
13. Kamkuemah M, Gausi B, Oni T. Missed opportunities for NCD multimorbidity prevention in adolescents and youth living with HIV in urban South Africa. BMC Public Health. 2020;20(1):821.
14. Barr AL, Young E, Smeeth L, Newton R, Seeley J, Ripullone K, et al. The need for an integrated approach for chronic disease research and care in Africa. Glob Health Epidemiol Genom. 2016;1.
15. Singer SJ, Burgers J, Friedberg M, Rosenthal MB, Leape L, Schneider E. Defining and measuring integrated patient care: promoting the next frontier in health care delivery. Medical Care Research and Review. 2011;68(1):112-27.
16. Battersby MW. Health reform through coordinated care: SA HealthPlus. Bmj. 2005;330(7492):662-5.
17. Geldsetzer P, Ortblad K, Bärnighausen T. The efficiency of chronic disease care in sub-Saharan Africa. BMC medicine. 2016;14(1):127.
18. Harries AD, Jahn A, Zachariah R, Enarson D. Adapting the DOTS framework for tuberculosis control to the management of non-communicable diseases in sub-Saharan Africa. PLoS medicine. 2008;5(6).
19. Rohwer A, Nicol JU, Toews I, Young T, Bavuma CM, Meerpohl J. Effects of integrated models of care for diabetes and hypertension in low-income and middle-income countries: a systematic review and meta-analysis. BMJ open. 2021;11(7):e043705.

For peer review only - http://bmjopen.bmj.com/site/about/guidelines.xhtml

20. Shamseer L, Moher D, Clarke M, Ghersi D, Liberati A, Petticrew M, et al. Preferred reporting items for systematic review and meta-analysis protocols (PRISMA-P) 2015: elaboration and explanation. Bmj. 2015;349.
21. Naghavi M, Abajobir AA, Abbafati C, Abbas KM, Abd-Allah F, Abera SF, et al. Global, regional, and national age-sex specific mortality for 264 causes of death, 1980–2016: a systematic analysis for the Global Burden of Disease Study 2016. The lancet. 2017;390(10100):1151-210.
22. Busetto L, Luijkx KG, Elissen AMJ, Vrijhoef HJM. Intervention types and outcomes of integrated care for diabetes mellitus type 2: a systematic review. Journal of evaluation in clinical practice. 2016;22(3):299-310.
23. Desmedt M, Vertriest S, Hellings J, Bergs J, Dessers E, Vankrunkelsven P, et al. Economic impact of integrated care models for patients with chronic diseases: a systematic review. Value in Health. 2016;19(6):892-902.
24. Drewes HW, Steuten LM, Lemmens LC, Baan CA, Boshuizen HC, Elissen AM, et al. The effectiveness of chronic care management for heart failure: meta-regression analyses to explain the heterogeneity in outcomes. Health services research. 2012;47(5):1926-59.
25. Elissen AM, Steuten LM, Lemmens LC, Drewes HW, Lemmens KM, Meeuwissen JA, et al. Meta-analysis of the effectiveness of chronic care management for diabetes: investigating heterogeneity in outcomes. Journal of Evaluation in Clinical Practice. 2013;19(5):753-62.
26. Meeuwissen JA, Lemmens LC, Drewes HW, Lemmens KM, Steuten LM, Elissen AM, et al. Meta-analysis and meta-regression analyses explaining heterogeneity in outcomes of chronic care management for depression: implications for person-centered mental healthcare. International Journal of Person Centered Medicine. 2012;2(4):716-58.
27. What study designs should be included in an EPOC review? EPOC Resources for review authors [Internet]. Norwegian Knowledge Centre for the Health Services. 2013. Available from: http://epoc.cochrane.org/epoc-specificresources-review-authors.
28. Wagner EH, Austin BT, Davis C, Hindmarsh M, Schaefer J, Bonomi A. Improving chronic illness care: translating evidence into action. Health affairs. 2001;20(6):64-78.
29. Practice CE, Care Oo. Suggested risk of bias criteria for EPOC reviews. 2017.
30. Hedges LV, Olkin I. Statistical methods for meta-analysis: Academic press; 2014.
31. Deeks JJ, Higgins JP, Altman DG, Group CSM. Analysing data and undertaking meta-analyses. Cochrane handbook for systematic reviews of interventions. 2019:241-84.
32. Hoaglin DC. Misunderstandings about Q and 'Cochran's Q test'in meta-analysis. Statistics in medicine. 2016;35(4):485-95.
33. Higgins JP, Thompson SG, Deeks JJ, Altman DG. Measuring inconsistency in meta-analyses. Bmj. 2003;327(7414):557-60.
34. Furuya-Kanamori L, Barendregt JJ, Doi SA. A new improved graphical and quantitative method for detecting bias in meta-analysis. JBI Evidence Implementation. 2018;16(4):195-203.
35. Xu C, Furuya-Kanamori L. DOIPLOT: Stata module for visualization of asymmetry and heterogeneity in meta-analysis. 2021.
36. Higgins JP, Eldridge S, Li T. Including variants on randomized trials. Cochrane handbook for systematic reviews of interventions. 2019:569-93.
37. Bolland MJ, Avenell A, Grey A. Analysis of cluster randomised trials as if they were individually randomised. The Lancet Diabetes & Endocrinology. 2023;11(2):75.
38. Guyatt G, Oxman AD, Akl EA, Kunz R, Vist G, Brozek J, et al. GRADE guidelines: 1. Introduction—GRADE evidence profiles and summary of findings tables. Journal of clinical epidemiology. 2011;64(4):383-94.
39. Santesso N, Glenton C, Dahm P, Garner P, Akl EA, Alper B, et al. GRADE guidelines 26: informative statements to communicate the findings of systematic reviews of interventions. Journal of clinical epidemiology. 2020;119:126-35.
40. Rahimi K, Bidel Z, Nazarzadeh M, Copland E, Canoy D, Ramakrishnan R, et al.

For peer review only

For peer review only - http://bmjopen.bmj.com/site/about/guidelines.xhtml

Pharmacological blood pressure lowering for primary and secondary prevention of cardiovascular disease across different levels of blood pressure: an individual participant-level data meta-analysis. The Lancet. 2021;397(10285):1625-36.
41. Kaiafa G, Veneti S, Polychronopoulos G, Pilalas D, Daios S, Kanellos I, et al. Is HbA1c an ideal biomarker of well-controlled diabetes? Postgraduate Medical Journal. 2021;97(1148):380-3.
42. Puhan MA, Schünemann HJ, Murad MH, Li T, Brignardello-Petersen R, Singh JA, et al. A GRADE Working Group approach for rating the quality of treatment effect estimates from network meta-analysis. Bmj. 2014;349.
43. Havlir DV, Balzer LB, Charlebois ED, Clark TD, Kwarisiima D, Ayieko J, et al. HIV testing and treatment with the use of a community health approach in rural Africa. New England Journal of Medicine. 2019;381(3):219-29.
44. Jackson IL, Ukwe CV. Clinical outcomes of pharmaceutical care intervention in HIV positive patients with hypertension: a randomized controlled study. Journal of Clinical Pharmacy and Therapeutics. 2021;46(4):1083-94.
45. Jackson IL, Ukwe CV. Effects of pharmaceutical care interventions on humanistic outcomes in hypertensive people living with HIV: results of a randomized controlled trial. International Journal of Pharmacy Practice. 2022;30(3):261-7.
46. Myers B, Lombard CJ, Lund C, Joska JA, Levitt N, Naledi T, et al. Comparing dedicated and designated approaches to integrating task-shared psychological interventions into chronic disease care in South Africa: a three-arm, cluster randomised, multicentre, open-label trial. The Lancet. 2022;400(10360):1321-33.
47. Okube OT, Kimani S, Mirie W. Community-based lifestyle intervention improves metabolic syndrome and related markers among Kenyan adults. Journal of Diabetes & Metabolic Disorders. 2022;21(1):607-21.
48. Owolabi MO, Gebregziabher M, Akinyemi RO, Akinyemi JO, Akpa O, Olaniyan O, et al. Randomized trial of an intervention to improve blood pressure control in stroke survivors. Circulation: Cardiovascular Quality and Outcomes. 2019;12(12):e005904.
49. Petersen I, Fairall L, Zani B, Bhana A, Lombard C, Folb N, et al. Effectiveness of a task-sharing collaborative care model for identification and management of depressive symptoms in patients with hypertension attending public sector primary care clinics in South Africa: pragmatic parallel cluster randomised controlled trial. Journal of Affective Disorders. 2021;282:112-21.
50. Rabkin M, Palma AM, McNairy ML, Simelane S, Gachuhi AB, Bitchong R, et al. Integrated vs. referred management of CVD risk factors for HIV positive patients on antiretroviral therapy in Swaziland. 2018.
51. Roos R, Myezwa H, van Aswegen H, Musenge E. Effects of an education and home-based pedometer walking program on ischemic heart disease risk factors in people infected with HIV: a randomized trial. JAIDS Journal of Acquired Immune Deficiency Syndromes. 2014;67(3):268-76.
52. Sarfo FS, Treiber F, Gebregziabher M, Adamu S, Nichols M, Singh A, et al. Phone-based intervention for blood pressure control among Ghanaian stroke survivors: a pilot randomized controlled trial. International Journal of Stroke. 2019;14(6):630-8.
53. Thuita AW, Kiage BN, Onyango AN, Makokha AO. Effect of a nutrition education programme on the metabolic syndrome in type 2 diabetes mellitus patients at a level 5 Hospital in Kenya:"a randomized controlled trial". BMC nutrition. 2020;6:1-14.
54. Jackson IL, Ukwe CV. Effects of pharmaceutical care interventions on humanistic outcomes in hypertensive people living with HIV: results of a randomized controlled trial. International Journal of Pharmacy Practice. 2022.
55. Okube OT, Kimani S, Mirie W. Community-based lifestyle intervention improves metabolic syndrome and related markers among Kenyan adults. Journal of Diabetes & Metabolic Disorders. 2022:1-15.
56. Goh LH, Siah CJR, Tam WWS, Tai ES, Young DYL. Effectiveness of the chronic care model for

For peer review only - http://bmjopen.bmj.com/site/about/guidelines.xhtml

adults with type 2 diabetes in primary care: a systematic review and meta-analysis. Systematic Reviews. 2022;11(1):1-23.
57. Si D, Bailie R, Weeramanthri T. Effectiveness of chronic care model-oriented interventions to improve quality of diabetes care: a systematic review. Primary Health Care Research & Development. 2008;9(1):25-40.
58. Weingarten SR, Henning JM, Badamgarav E, Knight K, Hasselblad V, Gano Jr A, et al. Interventions used in disease management programmes for patients with chronic illnesswhich ones work? Meta-analysis of published reports. Bmj. 2002;325(7370):925.
59. Reboldi G, Gentile G, Angeli F, Ambrosio G, Mancia G, Verdecchia P. Effects of intensive blood pressure reduction on myocardial infarction and stroke in diabetes: a meta-analysis in 73 913 patients. Journal of hypertension. 2011;29(7):1253-69.
60. Joshi R, Alim M, Kengne AP, Jan S, Maulik PK, Peiris D, et al. Task shifting for non-communicable disease management in low and middle income countries–a systematic review. PLoS One. 2014;9(8):e103754.
61. Kemp CG, Weiner BJ, Sherr KH, Kupfer LE, Cherutich PK, Wilson D, et al. Implementation science for integration of HIV and non-communicable disease services in sub-Saharan Africa: a systematic review. Aids. 2018;32:S93-S105.
62. Thornicroft G, Ahuja S, Barber S, Chisholm D, Collins PY, Docrat S, et al. Integrated care for people with long-term mental and physical health conditions in low-income and middle-income countries. The Lancet Psychiatry. 2019;6(2):174-86.

List of Figures

Figure 1: PRISMA flow diagram of the article selection process

Figure 2: Risk of bias in included studies

Figure 3: Forest plot of the random effects meta-analysis of the effect of integrated versus standard care on SBP for people with cardiometabolic multimorbidity in Sub-Saharan Africa

For peer review only

For peer review only - http://bmjopen.bmj.com/site/about/guidelines.xhtml

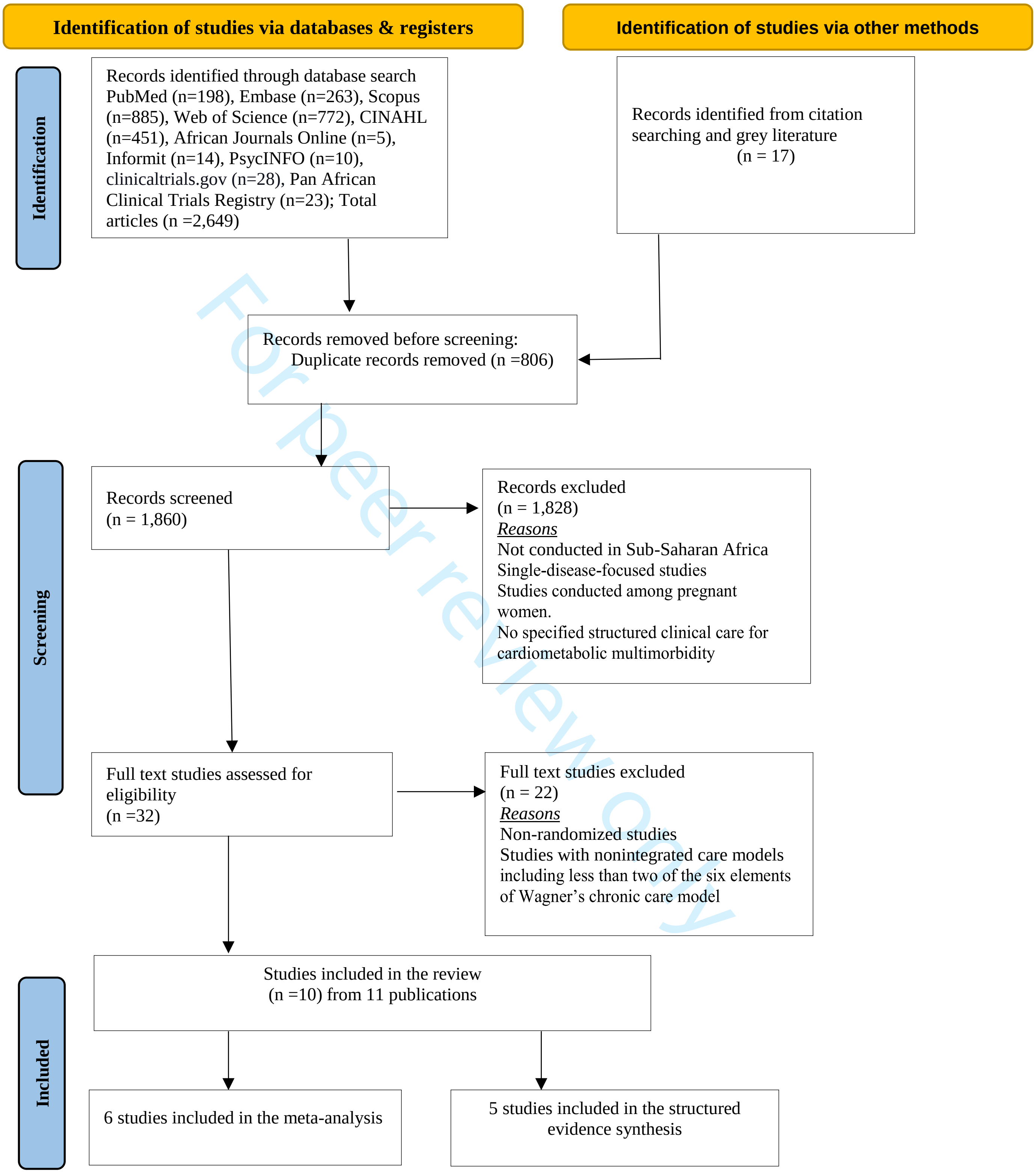

For peer review only

	Randomization process	Allocation concealment (selection bias)	Blinding (Performance and detection bias)	Selective reporting bias	Other bias	Protection against contamination	Reliable primary outcomes	Baseline measurements	Overall
Havlir et al (2019)	+	+	+	+	+	+	?	?	?
Jackson et al (2021)	+	+	+	+	+	?	+	+	+
Myers et al (2022)	+	+	+	+	+	+	+	+	+
Okube et al (2022)	+	+	+	+	+	?	+	+	+
Owolabi et al 2019	+	+	+	+	-	?	-	+	?
Petersen et al (2021)	+	+	+	+	+	+	+	+	+
Rabkin et al (2018)	+	?	+	+	-	?	+	+	?
Roos et al (2014)	+	+	+	+	+	?	-	+	?
Sarfo et al (2018)	+	+	+	?	-	+	-	+	?
Thuita et al (2020)	+	+	+	+	-	?	-	+	?

Low risk +
Some concerns ?
High risk -

ew only

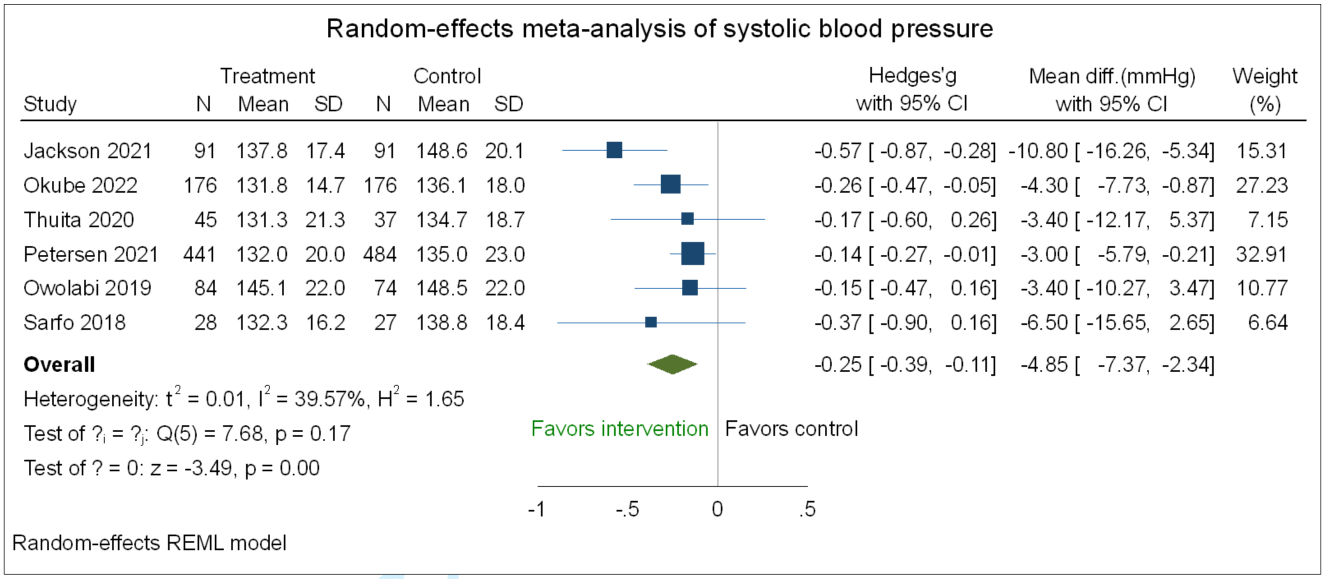

For peer review only

List of online supplementary files

Online supplementary file 1: Classification of interventions

Intervention component	Feature
Self-management support	• Self-care interventions based on technological aid<br>• Self-help groups<br>• Family-oriented supports<br>• Motivational support<br>• Behaviour therapy
Delivery system design	• Practice team functioning<br>• Patient care planning and follow-up<br>• Coordination between primary care and specialist services
Decision support	• Practice clinical guidelines<br>• Provider education<br>• Involvement of specialists in improving primary care
Clinical information system	• Disease registry<br>• Reminders to providers<br>• Feedback to providers
Healthcare organization	• Organizational goals and resources for chronic disease management<br>• Quality improvement strategies<br>• Incentives
Community linkages	• Linking patients to outside resources<br>• Activities with community-based organizations<br>• Professionals working out in the community

Supplementary file 2: Search strategies for electronic databases

1. **PubMED (Medline) Search strategy**

#1 ("elevated blood pressure"[MeSH Terms] OR "high blood pressure"[MeSH Terms] OR "raised blood pressure"[MeSH Terms] OR "diastolic blood pressure"[MeSH Terms] OR "systolic blood pressure"[MeSH Terms] OR "arterial blood pressure"[MeSH Terms] OR "uncontrolled blood pressure"[MeSH Terms] OR "uncontrolled hypertension"[All Fields] OR "elevated blood pressure"[All Fields] OR "high blood pressure"[All Fields] OR "raised blood pressure"[All Fields] OR "diastolic blood pressure"[All Fields] OR "systolic blood pressure"[All Fields] OR "arterial blood pressure"[All Fields] OR "uncontrolled blood pressure"[All Fields] OR "uncontrolled hypertension"[All Fields])

#2 ("diabetes mellitus"[MeSH Terms] OR "diabetes mellitus, type 2"[MeSH Terms] OR "diabetes type 2"[MeSH Terms] OR "T2DM"[MeSH Terms] OR "diabetes type II"[MeSH Terms] OR "diabetes"[MeSH Terms] OR "glucose intolerance"[MeSH Terms] OR "insulin resistance"[MeSH Terms] OR "hyperglycemia"[MeSH Terms] OR "hyperglycaemia"[MeSH Terms] OR "hypoglycaemia"[MeSH Terms] OR "hypoglycaemia"[MeSH Terms] OR "high blood sugar"[MeSH Terms] OR "elevated blood sugar"[MeSH Terms] OR "raised blood sugar"[MeSH Terms] OR "high blood glucose"[MeSH Terms] OR "elevated blood glucose"[MeSH Terms] OR "raised blood glucose"[MeSH Terms] OR "diabetes mellitus"[All Fields] OR "diabetes mellitus, type 2"[All Fields] OR "diabetes type 2"[All Fields] OR "T2DM"[All Fields] OR "diabetes type II"[All Fields] OR "diabetes"[All Fields] OR "glucose intolerance"[All Fields] OR "insulin resistance"[All Fields] OR "hyperglycemia"[All Fields] OR "hyperglycaemia"[All Fields] OR "hypoglycemia"[All Fields] OR "hypoglycaemia"[All Fields] OR "high blood sugar"[All Fields] OR "elevated blood sugar"[All Fields] OR "raised blood sugar"[All Fields] OR "high blood glucose"[All Fields] OR "elevated blood glucose"[All Fields] OR "raised blood glucose"[All Fields])

For peer review only - http://bmjopen.bmj.com/site/about/guidelines.xhtml

#3 ("stroke"[MeSH Terms] OR "transient ischemic attack"[MeSH Terms] OR "ischemic attack"[MeSH Terms] OR "angina"[MeSH Terms] OR "angina pectoris"[MeSH Terms] OR "heart attack"[MeSH Terms] OR "ischemic heart diseases "[MeSH Terms] OR "transient ischaemic attack"[MeSH Terms] OR "coronary heart disease"[MeSH Terms] OR "coronary disease"[MeSH Terms] OR "heart failure"[MeSH Terms] OR "peripheral vascular disease"[MeSH Terms] OR "Peripheral Vascular diseases"[MeSH Terms] OR "atrial fibrillation"[MeSH Terms] OR "cardiovascular disease"[MeSH Terms] OR "heart disease"[MeSH Terms] OR "stroke"[All Fields] OR "transient ischemic attack"[All Fields] OR "ischemic attack"[All Fields] OR "angina"[All Fields] OR "angina pectoris"[All Fields] OR "heart attack"[All Fields] OR "ischemic heart diseases "[All Fields] OR "transient ischaemic attack"[All Fields] OR "coronary heart disease"[All Fields] OR "coronary disease"[All Fields] OR "heart failure"[All Fields] OR "peripheral vascular disease"[All Fields] OR "Peripheral Vascular diseases"[All Fields] OR "atrial fibrillation"[All Fields] OR "cardiovascular disease"[All Fields] OR "heart disease"[All Fields])

#4 ("Hypercholesterolemia"[MeSH Terms] OR "Dyslipidemias"[MeSH Terms] OR "blood lipid"[MeSH Terms] OR "Cholesterol"[MeSH Terms] OR "high cholesterol"[MeSH Terms] OR "elevated cholesterol"[MeSH Terms] OR "raised cholesterol"[MeSH Terms] OR "low density lipoprotein "[MeSH Terms] OR "high density lipoprotein"[MeSH Terms] OR "Dyslipidemia"[MeSH Terms] OR "Dyslipidaemia"[MeSH Terms] OR "Hypercholesterolemia"[MeSH Terms] OR "hypercholesterolaemia "[MeSH Terms] OR "hypercholesterolimia "[MeSH Terms] OR "Hypertriglyceridemia"[MeSH Terms] OR "Hypertriglyceridemia"[MeSH Terms] OR "Hypertriglyceridaemia"[MeSH Terms] OR "Hyperlipidemia"[MeSH Terms] OR "Hyperlipidemias"[MeSH Terms] OR "Hyperlipidaemia"[MeSH Terms] OR "LDL"[MeSH Terms] OR "HDL"[MeSH Terms] OR "Hypercholesterolemia"[All Fields] OR "Dyslipidemias"[All Fields] OR "blood lipid"[All Fields] OR "Cholesterol"[All Fields] OR "high cholesterol"[All Fields] OR "elevated cholesterol"[All Fields] OR "raised cholesterol"[All Fields] OR "low density lipoprotein "[All Fields] OR "high density lipoprotein"[All Fields] OR "Dyslipidemia"[All Fields] OR "Dyslipidaemia"[All Fields] OR "Hypercholesterolemia"[All Fields] OR "hypercholesterolaemia "[All Fields] OR "hypercholesterolimia "[All Fields] OR "Hypertriglyceridemia"[All Fields] OR "Hypertriglyceridemia"[All Fields] OR "Hypertriglyceridaemia"[All Fields] OR "Hyperlipidemia"[All Fields] OR "Hyperlipidemias"[All Fields] OR "Hyperlipidaemia"[All Fields] OR "LDL"[All Fields] OR "HDL"[All Fields])

#5 ("Comorbid"[All Fields] OR "co-morbid"[All Fields] OR "co-morbidity"[All Fields] OR "Multimorbidity"[All Fields] OR "multi-morbid"[All Fields] OR "multi morbidity"[All Fields] OR "Multimorbidity"[All Fields] OR "Comorbidity"[All Fields] OR "multi-disease"[All Fields] OR "Multidisease"[All Fields] OR multi disease[All Fields] OR "multiple condition"[All Fields] OR "multi-condition"[All Fields] OR "multi condition"[All Fields] OR "multiple illness"[All Fields] OR "multi-illness"[All Fields] OR "multi illness"[All Fields] OR "multiple syndrome"[All Fields] OR "multi-syndrome"[All Fields] OR "multi syndrome"[All Fields] OR "concurrent condition"[All Fields] OR "concurrent illness"[All Fields] OR "concurrent disease"[All Fields] OR "co-existing disease"[All Fields] OR "coexisting disease"[All Fields] OR "co-existing illness"[All Fields] OR "coexisting illness"[All Fields] OR "co-existing syndrome"[All Fields] OR "coexisting syndrome"[All Fields] OR "co-existing condition"[All Fields] OR "coexisting condition"[All Fields] OR "co-occurring disease"[All Fields] OR "co-occuring disease"[All Fields] OR "co-occuring disease"[All Fields] OR "co-occurring illness"[All Fields] OR "co occurring illness"[All Fields] OR "cooccurring illness"[All Fields] OR "co-occurring syndrome"[All Fields] OR "co occurring syndrome"[All Fields] OR "cooccurring syndrome"[All Fields] OR "co-occurring condition"[All Fields] OR "co occurring condition"[All Fields] OR "cooccurring condition"[All Fields])

#6 ("Wagner model"[All Fields] OR "Wagner* model*"[All Fields] OR "Wagner* chronic care model*"[All Fields] OR "Wagner chronic care model"[All Fields] OR "chronic care model*"[All Fields] OR "model "[All Fields] OR "collaborative care"[All Fields] OR "chronic care framework"[All Fields] OR "chronic disease care"[All Fields] OR "chronic illness care"[All Fields] OR care model[All Fields] OR "model care"[All Fields] OR "Wagner* model*"[All Fields] OR "Wagner* chronic care model*"[All Fields] OR "Wagner chronic care model"[All Fields] OR "theory"[All Fields] OR "concept"[All Fields] OR "framework"[All Fields] OR "model"[All Fields] OR "programme"[All Fields] OR "approach"[All Fields] OR "clinical pathway"[All Fields] OR "care pathway"[All Fields] OR "critical path"[All Fields] OR "vertical integration"[All Fields] OR "virtual integration"[All Fields] OR "physician system integration"[All Fields] OR "provider system integration"[All Fields] OR "functional

For peer review only - http://bmjopen.bmj.com/site/about/guidelines.xhtml

integration"[All Fields] OR "horizontal integration"[All Fields] OR "clinical integration"[All Fields] OR "case management"[All Fields] OR "delivery of health care, integrated"[All Fields] OR "disease management"[All Fields] OR "patient care management"[All Fields] OR "patient-centred care"[All Fields] OR "accountable care organisations"[All Fields] OR "continuity of patient care"[All Fields] OR "case management"[All Fields] OR "comprehensive health care"[All Fields] OR "delivery of health care, integrated"[All Fields] OR "managed care programmes"[All Fields] OR "patient-centred care"[All Fields] OR "care delivery"[All Fields] OR "integrated care"[All Fields] OR "comprehensive care"[All Fields] OR "care coordination"[All Fields] OR "managed care"[All Fields] OR "accountable care "[All Fields] OR "accountable care organisations"[All Fields] OR "accountable care organisation"[All Fields] OR "collaborative care"[All Fields] OR "disease management"[All Fields] OR "case-management"[All Fields] OR "case management"[All Fields] OR "shared care"[All Fields] OR "accountable care"[All Fields] OR "patient-centred"[All Fields] OR "patient centred"[All Fields] OR "person-centred"[All Fields] OR "person centred"[All Fields] OR "multidisciplinary care"[All Fields] OR "interdisciplinary care"[All Fields] OR "inter-disciplinary care"[All Fields] OR "cross disciplinary care"[All Fields] OR "cross-disciplinary care"[All Fields] OR "multiple interventions"[All Fields] OR "care chain"[All Fields] OR "care chains"[All Fields] OR "care continuity"[All Fields] OR "care continuation"[All Fields] OR "care transition"[All Fields] OR "care transitions"[All Fields] OR "chain of care"[All Fields] OR "chains of care"[All Fields] OR "continuity of care"[All Fields] OR "cross sectoral care"[All Fields] OR "delivery of health care integrated"[All Fields] OR "integrated medicine"[All Fields] OR "integrated social network"[All Fields] OR "integrated social networks"[All Fields] OR "integration of care"[All Fields] OR "intersectoral care"[All Fields] OR "intrasectoral care"[All Fields] OR "linked care"[All Fields] OR "management model"[All Fields] OR "patient care management"[All Fields] OR "seamless care"[All Fields] OR "service network"[All Fields] OR "service networks"[All Fields] OR "transition of care"[All Fields] OR "transitional care"[All Fields] OR "transmural care"[All Fields] OR "whole system thinking"[All Fields] OR "holistic care"[All Fields])

#7 ("Angola "[All Fields] OR "Benin "[All Fields] OR "Botswana "[All Fields] OR "Burkina Faso"[All Fields] OR "Upper Volta"[All Fields] OR "Burundi "[All Fields] OR "Cameroon "[All Fields] OR "Cameroons "[All Fields] OR "Cape Verde"[All Fields] OR "Central African Republic"[All Fields] OR Chad[All Fields] OR "Comoros "[All Fields] OR "Comoro Islands"[All Fields] OR "Comores"[All Fields] OR "Mayotte "[All Fields] OR "Congo "[All Fields] OR "Zaire "[All Fields] OR "Cote d'Ivoire"[All Fields] OR "Ivory Coast"[All Fields] OR "Democratic Republic of the Congo"[All Fields] OR "Djibouti "[All Fields] OR "French Somaliland"[All Fields] OR "Eritrea "[All Fields] OR "Ethiopia "[All Fields] OR "Gabon "[All Fields] OR "Gabonese Republic"[All Fields] OR "Gambia "[All Fields] OR "Ghana "[All Fields] OR "Gold Coast"[All Fields] OR "Guinea "[All Fields] OR "Kenya "[All Fields] OR "Lesotho"[All Fields] OR "Basutoland"[All Fields] OR "Liberia "[All Fields] OR "Madagascar "[All Fields] OR "Malagasy Republic "[All Fields] OR "Malawi "[All Fields] OR "Nyasaland "[All Fields] OR "Mali "[All Fields] OR "Mauritania "[All Fields] OR "Mauritius "[All Fields] OR "Mozambique"[All Fields] OR "Namibia"[All Fields] OR "Niger"[All Fields] OR "Nigeria"[All Fields] OR "Rwanda"[All Fields] OR "Sao Tome"[All Fields] OR "Seychelles"[All Fields] OR "Senegal "[All Fields] OR "Sierra Leone"[All Fields] OR "Somalia "[All Fields] OR "South Africa"[All Fields] OR "Sudan "[All Fields] OR "Eswatini"[All Fields] OR "Tanzania"[All Fields] OR "Togo "[All Fields] OR "Togolese Republic"[All Fields] OR "Uganda"[All Fields] OR "Zambia"[All Fields] OR "Zimbabwe "[All Fields] OR "Rhodesia"[All Fields] OR "Sub-Saharan Africa"[All Fields] OR "Africa South of the Sahara"[All Fields])

#8 ((#1 OR #2 OR #3 OR #4 OR #5) AND #6 AND #7) AND Restricted (1999-2022, RCTs)

2. Web of Science Search Strategy

#1 elevated blood pressure OR high blood pressure OR raised blood pressure OR diastolic blood pressure OR systolic blood pressure OR arterial blood pressure OR uncontrolled blood pressure OR uncontrolled hypertension OR elevated blood pressure OR high blood pressure OR raised blood pressure OR diastolic blood pressure OR systolic blood pressure OR arterial blood pressure OR uncontrolled blood pressure OR uncontrolled hypertension

#2 diabetes mellitus OR diabetes mellitus, type 2 OR diabetes type 2 OR T2DM OR diabetes type II OR diabetes OR glucose intolerance OR insulin resistance OR hyperglycemia OR hyperglycaemia OR hypoglycaemia

For peer review only - http://bmjopen.bmj.com/site/about/guidelines.xhtml

OR hypoglycaemia OR high blood sugar OR elevated blood sugar OR raised blood sugar OR high blood glucose OR elevated blood glucose OR raised blood glucose OR diabetes mellitus OR diabetes mellitus, type 2 OR diabetes type 2 OR T2DM OR diabetes type II OR diabetes OR glucose intolerance OR insulin resistance OR hyperglycemia OR hyperglycaemia OR hypoglycemia OR hypoglycaemia OR high blood sugar OR elevated blood sugar OR raised blood sugar OR high blood glucose OR elevated blood glucose OR raised blood glucose

#3 stroke OR transient ischemic attack OR ischemic attack OR angina OR angina pectoris OR heart attack OR ischemic heart diseases OR transient ischaemic attack OR coronary heart disease OR coronary disease OR heart failure OR peripheral vascular disease OR Peripheral Vascular diseases OR atrial fibrillation OR cardiovascular disease OR heart disease OR stroke OR transient ischemic attack OR ischemic attack OR angina OR angina pectoris OR heart attack OR ischemic heart diseases OR transient ischaemic attack OR coronary heart disease OR coronary disease OR heart failure OR peripheral vascular disease OR Peripheral Vascular diseases OR atrial fibrillation OR cardiovascular disease OR heart disease

#4 Hypercholesterolemia OR Dyslipidemias OR blood lipid OR Cholesterol OR high cholesterol OR elevated cholesterol OR raised cholesterol OR low density lipoprotein OR high density lipoprotein OR Dyslipidemia OR Dyslipidaemia OR Hypercholesterolemia OR hypercholesterolaemia OR hypercholesterolimia OR Hypertriglyceridemia OR Hypertriglyceridemia OR Hypertriglyceridaemia OR Hyperlipidemia OR Hyperlipidemias OR Hyperlipidaemia OR LDL OR HDL OR Hypercholesterolemia OR Dyslipidemias OR blood lipid OR Cholesterol OR high cholesterol OR elevated cholesterol OR raised cholesterol OR low density lipoprotein OR high density lipoprotein OR Dyslipidemia OR Dyslipidaemia OR Hypercholesterolemia OR hypercholesterolaemia OR hypercholesterolimia OR Hypertriglyceridemia OR Hypertriglyceridemia OR Hypertriglyceridaemia OR Hyperlipidemia OR Hyperlipidemias OR Hyperlipidaemia OR LDL OR HDL

#5 Comorbid OR co-morbid OR co-morbidity OR Multimorbidity OR multi-morbid OR multi morbidity OR Multimorbidity OR Comorbidity OR multi-disease OR Multidisease OR multi disease OR multiple condition OR multi-condition OR multi condition OR multiple illness OR multi-illness OR multi illness OR multiple syndrome OR multi-syndrome OR multi syndrome OR concurrent condition OR concurrent illness OR concurrent disease OR co-existing disease OR coexisting disease OR co-existing illness OR coexisting illness OR co-existing syndrome OR coexisting syndrome OR co-existing condition OR coexisting condition OR co-occurring disease OR co-occuring disease OR co-occuring disease OR co-occurring illness OR co occurring illness OR cooccurring illness OR co-occurring syndrome OR co occurring syndrome OR cooccurring syndrome OR co-occurring condition OR co occurring condition OR cooccurring condition

#6 Wagner model OR Wagner* model* OR Wagner* chronic care model* OR Wagner chronic care model OR chronic care model* OR model OR collaborative care OR chronic care framework OR chronic disease care OR chronic illness care OR care model OR model care OR Wagner* model* OR Wagner* chronic care model* OR Wagner chronic care model OR theory OR concept OR framework OR model OR programme OR approach OR clinical pathway OR care pathway OR critical path OR vertical integration OR virtual integration OR physician system integration OR provider system integration OR functional integration OR horizontal integration OR clinical integration OR case management OR delivery of health care, integrated OR disease management OR patient care management OR patient-centred care OR accountable care organisations OR continuity of patient care OR case management OR comprehensive health care OR delivery of health care, integrated OR managed care programmes OR patient-centred care OR care delivery OR integrated care OR comprehensive care OR care coordination OR managed care OR accountable care OR accountable care organisations OR accountable care organisation OR collaborative care OR disease management OR case-management OR case management OR shared care OR accountable care OR patient-centred OR patient centred OR person-centred OR person centred OR multidisciplinary care OR interdisciplinary care OR inter-disciplinary care OR cross disciplinary care OR cross-disciplinary care OR multiple interventions OR care chain OR care chains OR care continuity OR care continuation OR care transition OR care transitions OR chain of care OR chains of care OR continuity of care OR cross sectoral care OR delivery of health care integrated OR integrated medicine OR integrated social network OR integrated social networks OR integration of care OR intersectoral care OR intrasectoral care OR linked care OR management model OR patient

For peer review only - http://bmjopen.bmj.com/site/about/guidelines.xhtml

care management OR seamless care OR service network OR service networks OR transition of care OR transitional care OR transmural care OR whole system thinking OR holistic care

#7 Angola OR Benin OR Botswana OR Burkina Faso OR Upper Volta OR Burundi OR Cameroon OR Cameroons OR Cape Verde OR Central African Republic OR Chad OR Comoros OR Comoro Islands OR Comores OR Mayotte OR Congo OR Zaire OR Cote d'Ivoire OR Ivory Coast OR Democratic Republic of the Congo OR Djibouti OR French Somaliland OR Eritrea OR Ethiopia OR Gabon OR Gabonese Republic OR Gambia OR Ghana OR Gold Coast OR Guinea OR Kenya OR Lesotho OR Basutoland OR Liberia OR Madagascar OR Malagasy Republic OR Malawi OR Nyasaland OR Mali OR Mauritania OR Mauritius OR Mozambique OR Namibia OR Niger OR Nigeria OR Rwanda OR Sao Tome OR Seychelles OR Senegal OR Sierra Leone OR Somalia OR South Africa OR Sudan OR Eswatini OR Tanzania OR Togo OR Togolese Republic OR Uganda OR Zambia OR Zimbabwe OR Rhodesia OR Sub-Saharan Africa OR Africa South of the Sahara

#8 trial* OR RCT

#9 ((#1 OR #2 OR #3 OR #4 OR #5) AND #6 AND #7 AND #8) and Clinical Trial (Document Types) and 1999 or 2000 or 2001 or 2002 or 2003 or 2004 or 2005 or 2006 or 2007 or 2008 or 2009 or 2011 or 2010 or 2012 or 2013 or 2014 or 2015 or 2016 or 2017 or 2018 or 2019 or 2020 or 2021 or 2022 (Publication Years)

3. SCOPUS

(TITLE-ABS-KEY (trial* OR rct)) AND (((TITLE-ABS-KEY ("wagner model" OR "wagner* model*" OR "wagner* chronic care model*" OR "wagner chronic care model" OR "chronic care model*" OR "model " OR "collaborative care" OR "chronic care framework" OR "chronic disease care" OR "chronic illness care" OR care AND model OR "model care" OR "wagner* model*" OR "wagner* chronic care model*" OR "wagner chronic care model" OR "theory" OR "concept" OR "framework" OR "model" OR "programme" OR "approach" OR "clinical pathway" OR "care pathway" OR "critical path" OR "vertical integration" OR "virtual integration" OR "physician system integration" OR "provider system integration" OR "functional integration" OR "horizontal integration" OR "clinical integration" OR "case management" OR "delivery of health care, integrated" OR "disease management" OR "patient care management" OR "patient-centred care")) OR (TITLE-ABS-KEY ("accountable care organisations" OR "continuity of patient care" OR "case management" OR "comprehensive health care" OR "delivery of health care, integrated" OR "managed care programmes" OR "patient-centred care" OR "care delivery" OR "integrated care" OR "comprehensive care" OR "care coordination" OR "managed care" OR "accountable care " OR "accountable care organisations" OR "accountable care organisation" OR "collaborative care" OR "disease management" OR "case-management" OR "case management" OR "shared care" OR "accountable care" OR "patient-centred" OR "patient centred" OR "person-centred" OR "person centred" OR "multidisciplinary care" OR "interdisciplinary care" OR "inter-disciplinary care" OR "cross disciplinary care" OR "cross-disciplinary care" OR "multiple interventions" OR "care chain" OR "care chains" OR "care continuity" OR "care continuation" OR "care transition")) OR (TITLE-ABS-KEY ("care transitions" OR "chain of care" OR "chains of care" OR "continuity of care" OR "cross sectoral care" OR "delivery of health care integrated" OR "integrated medicine" OR "integrated social network" OR "integrated social networks" OR "integration of care" OR "intersectoral care" OR "intrasectoral care" OR "linked care" OR "management model" OR "patient care management" OR "seamless care" OR "service network" OR "service networks" OR "transition of care" OR "transitional care" OR "transmural care" OR "whole system thinking" OR "holistic care"))) AND ((TITLE-ABS-KEY (("elevated blood pressure" OR "high blood pressure" OR "raised blood pressure" OR "diastolic blood pressure" OR "systolic blood pressure" OR "arterial blood pressure" OR "uncontrolled blood pressure" OR "uncontrolled hypertension" OR "elevated blood pressure" OR "high blood pressure" OR "raised blood pressure" OR "diastolic blood pressure" OR "systolic blood pressure" OR "arterial blood pressure" OR "uncontrolled blood pressure" OR "uncontrolled hypertension"))) OR (TITLE-ABS-KEY (("diabetes mellitus" OR "diabetes mellitus, type 2" OR "diabetes type 2" OR "t2dm" OR "diabetes type ii" OR "diabetes" OR "glucose intolerance" OR "insulin resistance" OR "hyperglycemia" OR "hyperglycaemia" OR "hypoglycaemia" OR "hypoglycaemia" OR "high blood sugar" OR "elevated blood sugar" OR "raised blood sugar" OR "high blood glucose" OR "elevated blood

For peer review only - http://bmjopen.bmj.com/site/about/guidelines.xhtml

glucose" OR "raised blood glucose" OR "diabetes mellitus" OR "diabetes mellitus, type 2" OR "diabetes type 2" OR "t2dm" OR "diabetes type ii" OR "diabetes" OR "glucose intolerance" OR "insulin resistance" OR "hyperglycemia" OR "hyperglycaemia" OR "hypoglycemia" OR "hypoglycaemia" OR "high blood sugar" OR "elevated blood sugar" OR "raised blood sugar" OR "high blood glucose" OR "elevated blood glucose" OR "raised blood glucose"))) OR (TITLE-ABS-KEY (("stroke" OR "transient ischemic attack" OR "ischemic attack" OR "angina" OR "angina pectoris" OR "heart attack" OR "ischemic heart diseases " OR "transient ischaemic attack" OR "coronary heart disease" OR "coronary disease" OR "heart failure" OR "peripheral vascular disease" OR "peripheral vascular diseases" OR "atrial fibrillation" OR "cardiovascular disease" OR "heart disease" OR "stroke" OR "transient ischemic attack" OR "ischemic attack" OR "angina" OR "angina pectoris" OR "heart attack" OR "ischemic heart diseases " OR "transient ischaemic attack" OR "coronary heart disease" OR "coronary disease" OR "heart failure" OR "peripheral vascular disease" OR "peripheral vascular diseases" OR "atrial fibrillation" OR "cardiovascular disease" OR "heart disease"))) OR (TITLE-ABS-KEY (("hypercholesterolemia" OR "dyslipidemias" OR "blood lipid" OR "cholesterol" OR "high cholesterol" OR "elevated cholesterol" OR "raised cholesterol" OR "low density lipoprotein " OR "high density lipoprotein" OR "dyslipidemia" OR "dyslipidaemia" OR "hypercholesterolemia" OR "hypercholesterolaemia " OR "hypercholesterolimia " OR "hypertriglyceridemia" OR "hypertriglyceridemia" OR "hypertriglyceridaemia" OR "hyperlipidemia" OR "hyperlipidemias" OR "hyperlipidaemia" OR "ldl" OR "hdl" OR "hypercholesterolemia" OR "dyslipidemias" OR "blood lipid" OR "cholesterol" OR "high cholesterol" OR "elevated cholesterol" OR "raised cholesterol" OR "low density lipoprotein " OR "high density lipoprotein" OR "dyslipidemia" OR "dyslipidaemia" OR "hypercholesterolemia" OR "hypercholesterolaemia " OR "hypercholesterolimia " OR "hypertriglyceridemia" OR "hypertriglyceridemia" OR "hypertriglyceridaemia" OR "hyperlipidemia" OR "hyperlipidemias" OR "hyperlipidaemia" OR "ldl" OR "hdl"))) OR (TITLE-ABS-KEY (("comorbid" OR "co-morbid" OR "co-morbidity" OR "multimorbidity" OR "multi-morbid" OR "multi morbidity" OR "multimorbidity" OR "comorbidity" OR "multi-disease" OR "multidisease" OR multi AND disease OR "multiple condition" OR "multi-condition" OR "multi condition" OR "multiple illness" OR "multi-illness" OR "multi illness" OR "multiple syndrome" OR "multi-syndrome" OR "multi syndrome" OR "concurrent condition" OR "concurrent illness" OR "concurrent disease" OR "co-existing disease" OR "coexisting disease" OR "co-existing illness" OR "coexisting illness" OR "co-existing syndrome" OR "coexisting syndrome" OR "co-existing condition" OR "coexisting condition" OR "co-occurring disease" OR "co-occuring disease" OR "co-occuring disease" OR "co-occurring illness" OR "co occurring illness" OR "cooccurring illness" OR "co-occurring syndrome" OR "co occurring syndrome" OR "cooccurring syndrome" OR "co-occurring condition" OR "co occurring condition" OR "cooccurring condition")))) AND (TITLE-ABS-KEY (("angola " OR "benin " OR "botswana " OR "burkina faso" OR "upper volta" OR "burundi " OR "cameroon " OR "cameroons " OR "cape verde" OR "central african republic" OR chad OR "comoros " OR "comoro islands" OR "comores" OR "mayotte " OR "congo " OR "zaire " OR "cote d'ivoire" OR "ivory coast" OR "democratic republic of the congo" OR "djibouti " OR "french somaliland" OR "eritrea " OR "ethiopia " OR "gabon " OR "gabonese republic" OR "gambia " OR "ghana " OR "gold coast" OR "guinea " OR "kenya " OR "lesotho" OR "basutoland" OR "liberia " OR "madagascar " OR "malagasy republic " OR "malawi " OR "nyasaland " OR "mali " OR "mauritania " OR "mauritius " OR "mozambique" OR "namibia" OR "niger" OR "nigeria" OR "rwanda" OR "sao tome" OR "seychelles" OR "senegal " OR "sierra leone" OR "somalia " OR "south africa" OR "sudan " OR "Eswatini" OR "tanzania" OR "togo " OR "Togolese republic" OR "uganda" OR "zambia" OR "zimbabwe " OR "rhodesia" OR "sub-saharan africa" OR "africa south of the sahara")))) AND (LIMIT-TO (PUBYEAR , 2022) OR LIMIT-TO (PUBYEAR , 2021) OR LIMIT-TO (PUBYEAR , 2020) OR LIMIT-TO (PUBYEAR , 2019) OR LIMIT-TO (PUBYEAR , 2018) OR LIMIT-TO (PUBYEAR , 2017) OR LIMIT-TO (PUBYEAR , 2016) OR LIMIT-TO (PUBYEAR , 2015) OR LIMIT-TO (PUBYEAR , 2014) OR LIMIT-TO (PUBYEAR , 2013) OR LIMIT-TO (PUBYEAR , 2012) OR LIMIT-TO (PUBYEAR , 2011) OR LIMIT-TO (PUBYEAR , 2010) OR LIMIT-TO (PUBYEAR , 2009) OR LIMIT-TO (PUBYEAR , 2008) OR LIMIT-TO (PUBYEAR , 2007) OR LIMIT-TO (PUBYEAR , 2006) OR LIMIT-TO (PUBYEAR , 2005) OR LIMIT-

For peer review only - http://bmjopen.bmj.com/site/about/guidelines.xhtml

TO (PUBYEAR , 2004) OR LIMIT-TO (PUBYEAR , 2003) OR LIMIT-TO (PUBYEAR , 2002) OR LIMIT-TO (PUBYEAR , 2001) OR LIMIT-TO (PUBYEAR , 2000) OR LIMIT-TO (PUBYEAR , 1999))

4. Embase

#1 ("elevated blood pressure"/ OR "high blood pressure"/ OR "raised blood pressure"/ OR "diastolic blood pressure"/ OR "systolic blood pressure"/ OR "arterial blood pressure"/ OR "uncontrolled blood pressure"/ OR "uncontrolled hypertension" OR "elevated blood pressure" OR "high blood pressure" OR "raised blood pressure" OR "diastolic blood pressure" OR "systolic blood pressure" OR "arterial blood pressure" OR "uncontrolled blood pressure" OR "uncontrolled hypertension")

#2 ("diabetes mellitus"/ OR "diabetes mellitus, type 2"/ OR "diabetes type 2"/ OR "T2DM"/ OR "diabetes type II"/ OR "diabetes"/ OR "glucose intolerance"/ OR "insulin resistance"/ OR "hyperglycemia"/ OR "hyperglycaemia"/ OR "hypoglycaemia"/ OR "hypoglycaemia"/ OR "high blood sugar"/ OR "elevated blood sugar"/ OR "raised blood sugar"/ OR "high blood glucose"/ OR "elevated blood glucose"/ OR "raised blood glucose"/ OR "diabetes mellitus" OR "diabetes mellitus, type 2" OR "diabetes type 2" OR "T2DM" OR "diabetes type II" OR "diabetes" OR "glucose intolerance" OR "insulin resistance" OR "hyperglycemia" OR "hyperglycaemia" OR "hypoglycemia" OR "hypoglycaemia" OR "high blood sugar" OR "elevated blood sugar" OR "raised blood sugar" OR "high blood glucose" OR "elevated blood glucose" OR "raised blood glucose")

#3 ("stroke"/ OR "transient ischemic attack"/ OR "ischemic attack"/ OR "angina"/ OR "angina pectoris"/ OR "heart attack"/ OR "ischemic heart diseases "/ OR "transient ischaemic attack"/ OR "coronary heart disease"/ OR "coronary disease"/ OR "heart failure"/ OR "peripheral vascular disease"/ OR "Peripheral Vascular diseases"/ OR "atrial fibrillation"/ OR "cardiovascular disease"/ OR "heart disease"/ OR "stroke" OR "transient ischemic attack" OR "ischemic attack" OR "angina" OR "angina pectoris" OR "heart attack" OR "ischemic heart diseases " OR "transient ischaemic attack" OR "coronary heart disease" OR "coronary disease" OR "heart failure" OR "peripheral vascular disease" OR "Peripheral Vascular diseases" OR "atrial fibrillation" OR "cardiovascular disease" OR "heart disease")

#4 ("Hypercholesterolemia"/ OR "Dyslipidemias"/ OR "blood lipid"/ OR "Cholesterol"/ OR "high cholesterol"/ OR "elevated cholesterol"/ OR "raised cholesterol"/ OR "low density lipoprotein "/ OR "high density lipoprotein"/ OR "Dyslipidemia"/ OR "Dyslipidaemia"/ OR "Hypercholesterolemia"/ OR "hypercholesterolaemia "/ OR "hypercholesterolimia "/ OR "Hypertriglyceridemia"/ OR "Hypertriglyceridemia"/ OR "Hypertriglyceridaemia"/ OR "Hyperlipidemia"/ OR "Hyperlipidemias"/ OR "Hyperlipidaemia"/ OR "LDL"/ OR "HDL"/ OR "Hypercholesterolemia" OR "Dyslipidemias" OR "blood lipid" OR "Cholesterol" OR "high cholesterol" OR "elevated cholesterol" OR "raised cholesterol" OR "low density lipoprotein " OR "high density lipoprotein" OR "Dyslipidemia" OR "Dyslipidaemia" OR "Hypercholesterolemia" OR "hypercholesterolaemia " OR "hypercholesterolimia " OR "Hypertriglyceridemia" OR "Hypertriglyceridemia" OR "Hypertriglyceridaemia" OR "Hyperlipidemia" OR "Hyperlipidemias" OR "Hyperlipidaemia" OR "LDL" OR "HDL")

#5 ("Comorbid" OR "co-morbid" OR "co-morbidity" OR "Multimorbidity" OR "multi-morbid" OR "multi morbidity" OR "Multimorbidity" OR "Comorbidity" OR "multi-disease" OR "Multidisease" OR multi disease OR "multiple condition" OR "multi-condition" OR "multi condition" OR "multiple illness" OR "multi-illness" OR "multi illness" OR "multiple syndrome" OR "multi-syndrome" OR "multi syndrome" OR "concurrent condition" OR "concurrent illness" OR "concurrent disease" OR "co-existing disease" OR "coexisting disease" OR "co-existing illness" OR "coexisting illness" OR "co-existing syndrome" OR "coexisting syndrome" OR "co-existing condition" OR "coexisting condition" OR "co-occurring disease" OR "co-occuring disease" OR "co-occuring disease" OR "co-occurring illness" OR "co occurring illness" OR "cooccurring illness" OR "co-occurring syndrome" OR "co occurring syndrome" OR "cooccurring syndrome" OR "co-occurring condition" OR "co occurring condition" OR "cooccurring condition")

#6 ("Wagner model" OR "Wagner* model*" OR "Wagner* chronic care model*" OR "Wagner chronic care model" OR "chronic care model*" OR "model " OR "collaborative care" OR "chronic care framework" OR "chronic disease care" OR "chronic illness care" OR care model OR "model care" OR "Wagner* model*" OR "Wagner* chronic care model*" OR "Wagner chronic care model" OR "theory" OR "concept" OR "framework" OR "model"

For peer review only - http://bmjopen.bmj.com/site/about/guidelines.xhtml

OR "programme" OR "approach" OR "clinical pathway" OR "care pathway" OR "critical path" OR "vertical integration" OR "virtual integration" OR "physician system integration" OR "provider system integration" OR "functional integration" OR "horizontal integration" OR "clinical integration" OR "case management" OR "delivery of health care, integrated" OR "disease management" OR "patient care management" OR "patient-centred care" OR "accountable care organisations" OR "continuity of patient care" OR "case management" OR "comprehensive health care" OR "delivery of health care, integrated" OR "managed care programmes" OR "patient-centred care" OR "care delivery" OR "integrated care" OR "comprehensive care" OR "care coordination" OR "managed care" OR "accountable care " OR "accountable care organisations" OR "accountable care organisation" OR "collaborative care" OR "disease management" OR "case-management" OR "case management" OR "shared care" OR "accountable care" OR "patient-centred" OR "patient centred" OR "person-centred" OR "person centred" OR "multidisciplinary care" OR "interdisciplinary care" OR "inter-disciplinary care" OR "cross disciplinary care" OR "cross-disciplinary care" OR "multiple interventions" OR "care chain" OR "care chains" OR "care continuity" OR "care continuation" OR "care transition" OR "care transitions" OR "chain of care" OR "chains of care" OR "continuity of care" OR "cross sectoral care" OR "delivery of health care integrated" OR "integrated medicine" OR "integrated social network" OR "integrated social networks" OR "integration of care" OR "intersectoral care" OR "intrasectoral care" OR "linked care" OR "management model" OR "patient care management" OR "seamless care" OR "service network" OR "service networks" OR "transition of care" OR "transitional care" OR "transmural care" OR "whole system thinking" OR "holistic care")

#7 ("Angola " OR "Benin " OR "Botswana " OR "Burkina Faso" OR "Upper Volta" OR "Burundi " OR "Cameroon " OR "Cameroons " OR "Cape Verde" OR "Central African Republic" OR Chad OR "Comoros " OR "Comoro Islands" OR "Comores" OR "Mayotte " OR "Congo " OR "Zaire " OR "Cote d'Ivoire" OR "Ivory Coast" OR "Democratic Republic of the Congo" OR "Djibouti " OR "French Somaliland" OR "Eritrea " OR "Ethiopia " OR "Gabon " OR "Gabonese Republic" OR "Gambia " OR "Ghana " OR "Gold Coast" OR "Guinea " OR "Kenya " OR "Lesotho" OR "Basutoland" OR "Liberia " OR "Madagascar " OR "Malagasy Republic " OR "Malawi " OR "Nyasaland " OR "Mali " OR "Mauritania " OR "Mauritius " OR "Mozambique" OR "Namibia" OR "Niger" OR "Nigeria" OR "Rwanda" OR "Sao Tome" OR "Seychelles" OR "Senegal " OR "Sierra Leone" OR "Somalia " OR "South Africa" OR "Sudan " OR "Eswatini" OR "Tanzania" OR "Togo " OR "Togolese Republic" OR "Uganda" OR "Zambia" OR "Zimbabwe " OR "Rhodesia" OR "Sub-Saharan Africa" OR "Africa South of the Sahara")

#8 ((#1 OR #2 OR #3 OR #4 OR #5) AND #6 AND #7) & 1999 - 2022 & RCTs

5. Cochrane Library

#1 (("elevated blood pressure"(MeSH Terms) OR "high blood pressure"(MeSH Terms) OR "raised blood pressure"(MeSH Terms) OR "diastolic blood pressure"(MeSH Terms) OR "systolic blood pressure"(MeSH Terms) OR "arterial blood pressure"(MeSH Terms) OR "uncontrolled blood pressure"(MeSH Terms) OR "uncontrolled hypertension" OR "elevated blood pressure" OR "high blood pressure" OR "raised blood pressure" OR "diastolic blood pressure" OR "systolic blood pressure" OR "arterial blood pressure" OR "uncontrolled blood pressure" OR "uncontrolled hypertension")):ti,ab,kw (Word variations have been searched)

#2 (("diabetes mellitus"(MeSH Terms) OR "diabetes mellitus, type 2"(MeSH Terms) OR "diabetes type 2"(MeSH Terms) OR "T2DM"(MeSH Terms) OR "diabetes type II"(MeSH Terms) OR "diabetes"(MeSH Terms) OR "glucose intolerance"(MeSH Terms) OR "insulin resistance"(MeSH Terms) OR "hyperglycemia"(MeSH Terms) OR "hyperglycaemia"(MeSH Terms) OR "hypoglycaemia"(MeSH Terms) OR "hypoglycaemia"(MeSH Terms) OR "high blood sugar"(MeSH Terms) OR "elevated blood sugar"(MeSH Terms) OR "raised blood sugar"(MeSH Terms) OR "high blood glucose"(MeSH Terms) OR "elevated blood glucose"(MeSH Terms) OR "raised blood glucose"(MeSH Terms) OR "diabetes mellitus" OR "diabetes mellitus, type 2" OR "diabetes type 2" OR "T2DM" OR "diabetes type II" OR "diabetes" OR "glucose intolerance" OR "insulin resistance" OR "hyperglycemia" OR "hyperglycaemia" OR "hypoglycemia" OR "hypoglycaemia" OR "high blood sugar" OR "elevated blood sugar" OR "raised blood sugar" OR "high blood glucose" OR "elevated blood glucose" OR "raised blood glucose")):ti,ab,kw (Word variations have been searched)

For peer review only - http://bmjopen.bmj.com/site/about/guidelines.xhtml

#3 (("stroke"(MeSH Terms) OR "transient ischemic attack"(MeSH Terms) OR "ischemic attack"(MeSH Terms) OR "angina"(MeSH Terms) OR "angina pectoris"(MeSH Terms) OR "heart attack"(MeSH Terms) OR "ischemic heart diseases "(MeSH Terms) OR "transient ischaemic attack"(MeSH Terms) OR "coronary heart disease"(MeSH Terms) OR "coronary disease"(MeSH Terms) OR "heart failure"(MeSH Terms) OR "peripheral vascular disease"(MeSH Terms) OR "Peripheral Vascular diseases"(MeSH Terms) OR "atrial fibrillation"(MeSH Terms) OR "cardiovascular disease"(MeSH Terms) OR "heart disease"(MeSH Terms) OR "stroke" OR "transient ischemic attack" OR "ischemic attack" OR "angina" OR "angina pectoris" OR "heart attack" OR "ischemic heart diseases " OR "transient ischaemic attack" OR "coronary heart disease" OR "coronary disease" OR "heart failure" OR "peripheral vascular disease" OR "Peripheral Vascular diseases" OR "atrial fibrillation" OR "cardiovascular disease" OR "heart disease")):ti,ab,kw (Word variations have been searched)

#4 (("Hypercholesterolemia"(MeSH Terms) OR "Dyslipidemias"(MeSH Terms) OR "blood lipid"(MeSH Terms) OR "Cholesterol"(MeSH Terms) OR "high cholesterol"(MeSH Terms) OR "elevated cholesterol"(MeSH Terms) OR "raised cholesterol"(MeSH Terms) OR "low density lipoprotein "(MeSH Terms) OR "high density lipoprotein"(MeSH Terms) OR "Dyslipidemia"(MeSH Terms) OR "Dyslipidaemia"(MeSH Terms) OR "Hypercholesterolemia"(MeSH Terms) OR "hypercholesterolaemia "(MeSH Terms) OR "hypercholesterolimia "(MeSH Terms) OR "Hypertriglyceridemia"(MeSH Terms) OR "Hypertriglyceridemia"(MeSH Terms) OR "Hypertriglyceridaemia"(MeSH Terms) OR "Hyperlipidemia"(MeSH Terms) OR "Hyperlipidemias"(MeSH Terms) OR "Hyperlipidaemia"(MeSH Terms) OR "LDL"(MeSH Terms) OR "HDL"(MeSH Terms) OR "Hypercholesterolemia" OR "Dyslipidemias" OR "blood lipid" OR "Cholesterol" OR "high cholesterol" OR "elevated cholesterol" OR "raised cholesterol" OR "low density lipoprotein " OR "high density lipoprotein" OR "Dyslipidemia" OR "Dyslipidaemia" OR "Hypercholesterolemia" OR "hypercholesterolaemia " OR "hypercholesterolimia " OR "Hypertriglyceridemia" OR "Hypertriglyceridemia" OR "Hypertriglyceridaemia" OR "Hyperlipidemia" OR "Hyperlipidemias" OR "Hyperlipidaemia" OR "LDL" OR "HDL")):ti,ab,kw (Word variations have been searched)

#5 (("Comorbid" OR "co-morbid" OR "co-morbidity" OR "Multimorbidity" OR "multi-morbid" OR "multi morbidity" OR "Multimorbidity" OR "Comorbidity" OR "multi-disease" OR "Multidisease" OR multi disease OR "multiple condition" OR "multi-condition" OR "multi condition" OR "multiple illness" OR "multi-illness" OR "multi illness" OR "multiple syndrome" OR "multi-syndrome" OR "multi syndrome" OR "concurrent condition" OR "concurrent illness" OR "concurrent disease" OR "co-existing disease" OR "coexisting disease" OR "co-existing illness" OR "coexisting illness" OR "co-existing syndrome" OR "coexisting syndrome" OR "co-existing condition" OR "coexisting condition" OR "co-occurring disease" OR "co-occuring disease" OR "co-occuring disease" OR "co-occurring illness" OR "co occurring illness" OR "cooccurring illness" OR "co-occurring syndrome" OR "co occurring syndrome" OR "cooccurring syndrome" OR "co-occurring condition" OR "co occurring condition" OR "cooccurring condition")):ti,ab,kw (Word variations have been searched)

#6 (("Wagner model" OR "Wagner* model*" OR "Wagner* chronic care model*" OR "Wagner chronic care model" OR "chronic care model*" OR "model " OR "collaborative care" OR "chronic care framework" OR "chronic disease care" OR "chronic illness care" OR care model OR "model care" OR "Wagner* model*" OR "Wagner* chronic care model*" OR "Wagner chronic care model" OR "theory" OR "concept" OR "framework" OR "model" OR "programme" OR "approach" OR "clinical pathway" OR "care pathway" OR "critical path" OR "vertical integration" OR "virtual integration" OR "physician system integration" OR "provider system integration" OR "functional integration" OR "horizontal integration" OR "clinical integration" OR "case management" OR "delivery of health care, integrated" OR "disease management" OR "patient care management" OR "patient-centred care" OR "accountable care organisations" OR "continuity of patient care" OR "case management" OR "comprehensive health care" OR "delivery of health care, integrated" OR "managed care programmes" OR "patient-centred care" OR "care delivery" OR "integrated care" OR "comprehensive care" OR "care coordination" OR "managed care" OR "accountable care " OR "accountable care organisations" OR "accountable care organisation" OR "collaborative care" OR "disease management" OR "case-management" OR "case management" OR "shared care" OR "accountable care" OR "patient-centred" OR "patient centred" OR "person-centred" OR "person centred" OR "multidisciplinary care" OR "interdisciplinary care" OR "inter-disciplinary care" OR "cross disciplinary care" OR "cross-disciplinary care" OR "multiple interventions" OR "care chain" OR "care chains" OR "care continuity" OR

For peer review only - http://bmjopen.bmj.com/site/about/guidelines.xhtml

"care continuation" OR "care transition" OR "care transitions" OR "chain of care" OR "chains of care" OR "continuity of care" OR "cross sectoral care" OR "delivery of health care integrated" OR "integrated medicine" OR "integrated social network" OR "integrated social networks" OR "integration of care" OR "intersectoral care" OR "intrasectoral care" OR "linked care" OR "management model" OR "patient care management" OR "seamless care" OR "service network" OR "service networks" OR "transition of care" OR "transitional care" OR "transmural care" OR "whole system thinking" OR "holistic care")):ti,ab,kw (Word variations have been searched)

#7 (("Angola " OR "Benin " OR "Botswana " OR "Burkina Faso" OR "Upper Volta" OR "Burundi " OR "Cameroon " OR "Cameroons " OR "Cape Verde" OR "Central African Republic" OR Chad OR "Comoros " OR "Comoro Islands" OR "Comores" OR "Mayotte " OR "Congo " OR "Zaire " OR "Cote d'Ivoire" OR "Ivory Coast" OR "Democratic Republic of the Congo" OR "Djibouti " OR "French Somaliland" OR "Eritrea " OR "Ethiopia " OR "Gabon " OR "Gabonese Republic" OR "Gambia " OR "Ghana " OR "Gold Coast" OR "Guinea " OR "Kenya " OR "Lesotho" OR "Basutoland" OR "Liberia " OR "Madagascar " OR "Malagasy Republic " OR "Malawi " OR "Nyasaland " OR "Mali " OR "Mauritania " OR "Mauritius " OR "Mozambique" OR "Namibia" OR "Niger" OR "Nigeria" OR "Rwanda" OR "Sao Tome" OR "Seychelles" OR "Senegal " OR "Sierra Leone" OR "Somalia " OR "South Africa" OR "Sudan " OR "Eswatini" OR "Tanzania" OR "Togo " OR "Togolese Republic" OR "Uganda" OR "Zambia" OR "Zimbabwe " OR "Rhodesia" OR "Sub-Saharan Africa" OR "Africa South of the Sahara")):ti,ab,kw (Word variations have been searched)

#8 ((#1 OR #2 OR #3 OR #4 OR #5) AND #6 AND #7) AND Restricted (1999-2022, RCTs)

6. Psycinfo via Ovid

#1 ("elevated blood pressure"/ OR "high blood pressure"/ OR "raised blood pressure"/ OR "diastolic blood pressure"/ OR "systolic blood pressure"/ OR "arterial blood pressure"/ OR "uncontrolled blood pressure"/ OR "uncontrolled hypertension" OR "elevated blood pressure" OR "high blood pressure" OR "raised blood pressure" OR "diastolic blood pressure" OR "systolic blood pressure" OR "arterial blood pressure" OR "uncontrolled blood pressure" OR "uncontrolled hypertension")

#2 ("diabetes mellitus"/ OR "diabetes mellitus, type 2"/ OR "diabetes type 2"/ OR "T2DM"/ OR "diabetes type II"/ OR "diabetes"/ OR "glucose intolerance"/ OR "insulin resistance"/ OR "hyperglycemia"/ OR "hyperglycaemia"/ OR "hypoglycaemia"/ OR "hypoglycaemia"/ OR "high blood sugar"/ OR "elevated blood sugar"/ OR "raised blood sugar"/ OR "high blood glucose"/ OR "elevated blood glucose"/ OR "raised blood glucose"/ OR "diabetes mellitus" OR "diabetes mellitus, type 2" OR "diabetes type 2" OR "T2DM" OR "diabetes type II" OR "diabetes" OR "glucose intolerance" OR "insulin resistance" OR "hyperglycemia" OR "hyperglycaemia" OR "hypoglycemia" OR "hypoglycaemia" OR "high blood sugar" OR "elevated blood sugar" OR "raised blood sugar" OR "high blood glucose" OR "elevated blood glucose" OR "raised blood glucose")

#3 ("stroke"/ OR "transient ischemic attack"/ OR "ischemic attack"/ OR "angina"/ OR "angina pectoris"/ OR "heart attack"/ OR "ischemic heart diseases "/ OR "transient ischaemic attack"/ OR "coronary heart disease"/ OR "coronary disease"/ OR "heart failure"/ OR "peripheral vascular disease"/ OR "Peripheral Vascular diseases"/ OR "atrial fibrillation"/ OR "cardiovascular disease"/ OR "heart disease"/ OR "stroke" OR "transient ischemic attack" OR "ischemic attack" OR "angina" OR "angina pectoris" OR "heart attack" OR "ischemic heart diseases " OR "transient ischaemic attack" OR "coronary heart disease" OR "coronary disease" OR "heart failure" OR "peripheral vascular disease" OR "Peripheral Vascular diseases" OR "atrial fibrillation" OR "cardiovascular disease" OR "heart disease")

#4 ("Hypercholesterolemia"/ OR "Dyslipidemias"/ OR "blood lipid"/ OR "Cholesterol"/ OR "high cholesterol"/ OR "elevated cholesterol"/ OR "raised cholesterol"/ OR "low density lipoprotein "/ OR "high density lipoprotein"/ OR "Dyslipidemia"/ OR "Dyslipidaemia"/ OR "Hypercholesterolemia"/ OR "hypercholesterolaemia "/ OR "hypercholesterolimia "/ OR "Hypertriglyceridemia"/ OR "Hypertriglyceridemia"/ OR "Hypertriglyceridaemia"/ OR "Hyperlipidemia"/ OR "Hyperlipidemias"/ OR "Hyperlipidaemia"/ OR "LDL"/ OR "HDL"/ OR "Hypercholesterolemia" OR "Dyslipidemias" OR "blood lipid" OR "Cholesterol" OR "high cholesterol" OR "elevated cholesterol" OR "raised cholesterol" OR "low density lipoprotein " OR "high density lipoprotein" OR

For peer review only - http://bmjopen.bmj.com/site/about/guidelines.xhtml

"Dyslipidemia" OR "Dyslipidaemia" OR "Hypercholesterolemia" OR "hypercholesterolaemia " OR "hypercholesterolimia " OR "Hypertriglyceridemia" OR "Hypertriglyceridemia" OR "Hypertriglyceridaemia" OR "Hyperlipidemia" OR "Hyperlipidemias" OR "Hyperlipidaemia" OR "LDL" OR "HDL")

#5 ("Comorbid" OR "co-morbid" OR "co-morbidity" OR "Multimorbidity" OR "multi-morbid" OR "multi morbidity" OR "Multimorbidity" OR "Comorbidity" OR "multi-disease" OR "Multidisease" OR multi disease OR "multiple condition" OR "multi-condition" OR "multi condition" OR "multiple illness" OR "multi-illness" OR "multi illness" OR "multiple syndrome" OR "multi-syndrome" OR "multi syndrome" OR "concurrent condition" OR "concurrent illness" OR "concurrent disease" OR "co-existing disease" OR "coexisting disease" OR "co-existing illness" OR "coexisting illness" OR "co-existing syndrome" OR "coexisting syndrome" OR "co-existing condition" OR "coexisting condition" OR "co-occurring disease" OR "co-occuring disease" OR "co-occuring disease" OR "co-occurring illness" OR "co occurring illness" OR "cooccurring illness" OR "co-occurring syndrome" OR "co occurring syndrome" OR "cooccurring syndrome" OR "co-occurring condition" OR "co occurring condition" OR "cooccurring condition")

#6 ("Wagner model" OR "Wagner* model*" OR "Wagner* chronic care model*" OR "Wagner chronic care model" OR "chronic care model*" OR "model " OR "collaborative care" OR "chronic care framework" OR "chronic disease care" OR "chronic illness care" OR care model OR "model care" OR "Wagner* model*" OR "Wagner* chronic care model*" OR "Wagner chronic care model" OR "theory" OR "concept" OR "framework" OR "model" OR "programme" OR "approach" OR "clinical pathway" OR "care pathway" OR "critical path" OR "vertical integration" OR "virtual integration" OR "physician system integration" OR "provider system integration" OR "functional integration" OR "horizontal integration" OR "clinical integration" OR "case management" OR "delivery of health care, integrated" OR "disease management" OR "patient care management" OR "patient-centred care" OR "accountable care organisations" OR "continuity of patient care" OR "case management" OR "comprehensive health care" OR "delivery of health care, integrated" OR "managed care programmes" OR "patient-centred care" OR "care delivery" OR "integrated care" OR "comprehensive care" OR "care coordination" OR "managed care" OR "accountable care " OR "accountable care organisations" OR "accountable care organisation" OR "collaborative care" OR "disease management" OR "case-management" OR "case management" OR "shared care" OR "accountable care" OR "patient-centred" OR "patient centred" OR "person-centred" OR "person centred" OR "multidisciplinary care" OR "interdisciplinary care" OR "inter-disciplinary care" OR "cross disciplinary care" OR "cross-disciplinary care" OR "multiple interventions" OR "care chain" OR "care chains" OR "care continuity" OR "care continuation" OR "care transition" OR "care transitions" OR "chain of care" OR "chains of care" OR "continuity of care" OR "cross sectoral care" OR "delivery of health care integrated" OR "integrated medicine" OR "integrated social network" OR "integrated social networks" OR "integration of care" OR "intersectoral care" OR "intrasectoral care" OR "linked care" OR "management model" OR "patient care management" OR "seamless care" OR "service network" OR "service networks" OR "transition of care" OR "transitional care" OR "transmural care" OR "whole system thinking" OR "holistic care")

#7 ("Angola " OR "Benin " OR "Botswana " OR "Burkina Faso" OR "Upper Volta" OR "Burundi " OR "Cameroon " OR "Cameroons " OR "Cape Verde" OR "Central African Republic" OR Chad OR "Comoros " OR "Comoro Islands" OR "Comores" OR "Mayotte " OR "Congo " OR "Zaire " OR "Cote d'Ivoire" OR "Ivory Coast" OR "Democratic Republic of the Congo" OR "Djibouti " OR "French Somaliland" OR "Eritrea " OR "Ethiopia " OR "Gabon " OR "Gabonese Republic" OR "Gambia " OR "Ghana " OR "Gold Coast" OR "Guinea " OR "Kenya " OR "Lesotho" OR "Basutoland" OR "Liberia " OR "Madagascar " OR "Malagasy Republic " OR "Malawi " OR "Nyasaland " OR "Mali " OR "Mauritania " OR "Mauritius " OR "Mozambique" OR "Namibia" OR "Niger" OR "Nigeria" OR "Rwanda" OR "Sao Tome" OR "Seychelles" OR "Senegal " OR "Sierra Leone" OR "Somalia " OR "South Africa" OR "Sudan " OR "Eswatini" OR "Tanzania" OR "Togo " OR "Togolese Republic" OR "Uganda" OR "Zambia" OR "Zimbabwe " OR "Rhodesia" OR "Sub-Saharan Africa" OR "Africa South of the Sahara")

8 ((#1 OR #2 OR #3 OR #4 OR #5) AND #6 AND #7)

9 limit 8 to ("0300 clinical trial" and yr="1999 - 2022")

7. CINAHL

For peer review only - http://bmjopen.bmj.com/site/about/guidelines.xhtml

#1 ("elevated blood pressure" OR "high blood pressure" OR "raised blood pressure" OR "diastolic blood pressure" OR "systolic blood pressure" OR "arterial blood pressure" OR "uncontrolled blood pressure" OR "uncontrolled hypertension" OR "elevated blood pressure" OR "high blood pressure" OR "raised blood pressure" OR "diastolic blood pressure" OR "systolic blood pressure" OR "arterial blood pressure" OR "uncontrolled blood pressure" OR "uncontrolled hypertension")

#2 ("diabetes mellitus" OR "diabetes mellitus, type 2" OR "diabetes type 2" OR "T2DM" OR "diabetes type II" OR "diabetes" OR "glucose intolerance" OR "insulin resistance" OR "hyperglycemia" OR "hyperglycaemia" OR "hypoglycaemia" OR "hypoglycaemia" OR "high blood sugar" OR "elevated blood sugar" OR "raised blood sugar" OR "high blood glucose" OR "elevated blood glucose" OR "raised blood glucose" OR "diabetes mellitus" OR "diabetes mellitus, type 2" OR "diabetes type 2" OR "T2DM" OR "diabetes type II" OR "diabetes" OR "glucose intolerance" OR "insulin resistance" OR "hyperglycemia" OR "hyperglycaemia" OR "hypoglycemia" OR "hypoglycaemia" OR "high blood sugar" OR "elevated blood sugar" OR "raised blood sugar" OR "high blood glucose" OR "elevated blood glucose" OR "raised blood glucose")

#3 ("stroke" OR "transient ischemic attack" OR "ischemic attack" OR "angina" OR "angina pectoris" OR "heart attack" OR "ischemic heart diseases " OR "transient ischaemic attack" OR "coronary heart disease" OR "coronary disease" OR "heart failure" OR "peripheral vascular disease" OR "Peripheral Vascular diseases" OR "atrial fibrillation" OR "cardiovascular disease" OR "heart disease" OR "stroke" OR "transient ischemic attack" OR "ischemic attack" OR "angina" OR "angina pectoris" OR "heart attack" OR "ischemic heart diseases " OR "transient ischaemic attack" OR "coronary heart disease" OR "coronary disease" OR "heart failure" OR "peripheral vascular disease" OR "Peripheral Vascular diseases" OR "atrial fibrillation" OR "cardiovascular disease" OR "heart disease")

#4 ("Hypercholesterolemia" OR "Dyslipidemias" OR "blood lipid" OR "Cholesterol" OR "high cholesterol" OR "elevated cholesterol" OR "raised cholesterol" OR "low density lipoprotein " OR "high density lipoprotein" OR "Dyslipidemia" OR "Dyslipidaemia" OR "Hypercholesterolemia" OR "hypercholesterolaemia " OR "hypercholesterolimia " OR "Hypertriglyceridemia" OR "Hypertriglyceridemia" OR "Hypertriglyceridaemia" OR "Hyperlipidemia" OR "Hyperlipidemias" OR "Hyperlipidaemia" OR "LDL" OR "HDL" OR "Hypercholesterolemia" OR "Dyslipidemias" OR "blood lipid" OR "Cholesterol" OR "high cholesterol" OR "elevated cholesterol" OR "raised cholesterol" OR "low density lipoprotein " OR "high density lipoprotein" OR "Dyslipidemia" OR "Dyslipidaemia" OR "Hypercholesterolemia" OR "hypercholesterolaemia " OR "hypercholesterolimia " OR "Hypertriglyceridemia" OR "Hypertriglyceridemia" OR "Hypertriglyceridaemia" OR "Hyperlipidemia" OR "Hyperlipidemias" OR "Hyperlipidaemia" OR "LDL" OR "HDL")

#5 ("Comorbid" OR "co-morbid" OR "co-morbidity" OR "Multimorbidity" OR "multi-morbid" OR "multi morbidity" OR "Multimorbidity" OR "Comorbidity" OR "multi-disease" OR "Multidisease" OR multi disease OR "multiple condition" OR "multi-condition" OR "multi condition" OR "multiple illness" OR "multi-illness" OR "multi illness" OR "multiple syndrome" OR "multi-syndrome" OR "multi syndrome" OR "concurrent condition" OR "concurrent illness" OR "concurrent disease" OR "co-existing disease" OR "coexisting disease" OR "co-existing illness" OR "coexisting illness" OR "co-existing syndrome" OR "coexisting syndrome" OR "co-existing condition" OR "coexisting condition" OR "co-occurring disease" OR "co-occuring disease" OR "co-occuring disease" OR "co-occurring illness" OR "co occurring illness" OR "cooccurring illness" OR "co-occurring syndrome" OR "co occurring syndrome" OR "cooccurring syndrome" OR "co-occurring condition" OR "co occurring condition" OR "cooccurring condition")

#6 ("Wagner model" OR "Wagner* model*" OR "Wagner* chronic care model*" OR "Wagner chronic care model" OR "chronic care model*" OR "model " OR "collaborative care" OR "chronic care framework" OR "chronic disease care" OR "chronic illness care" OR care model OR "model care" OR "Wagner* model*" OR "Wagner* chronic care model*" OR "Wagner chronic care model" OR "theory" OR "concept" OR "framework" OR "model" OR "programme" OR "approach" OR "clinical pathway" OR "care pathway" OR "critical path" OR "vertical integration" OR "virtual integration" OR "physician system integration" OR "provider system integration" OR "functional integration" OR "horizontal integration" OR "clinical integration" OR "case management" OR "delivery of health care, integrated" OR "disease management" OR "patient care management" OR "patient-centred care" OR "accountable care organisations" OR "continuity of patient care" OR "case management" OR "comprehensive health

For peer review only - http://bmjopen.bmj.com/site/about/guidelines.xhtml

care" OR "delivery of health care, integrated" OR "managed care programmes" OR "patient-centred care" OR "care delivery" OR "integrated care" OR "comprehensive care" OR "care coordination" OR "managed care" OR "accountable care " OR "accountable care organisations" OR "accountable care organisation" OR "collaborative care" OR "disease management" OR "case-management" OR "case management" OR "shared care" OR "accountable care" OR "patient-centred" OR "patient centred" OR "person-centred" OR "person centred" OR "multidisciplinary care" OR "interdisciplinary care" OR "inter-disciplinary care" OR "cross disciplinary care" OR "cross-disciplinary care" OR "multiple interventions" OR "care chain" OR "care chains" OR "care continuity" OR "care continuation" OR "care transition" OR "care transitions" OR "chain of care" OR "chains of care" OR "continuity of care" OR "cross sectoral care" OR "delivery of health care integrated" OR "integrated medicine" OR "integrated social network" OR "integrated social networks" OR "integration of care" OR "intersectoral care" OR "intrasectoral care" OR "linked care" OR "management model" OR "patient care management" OR "seamless care" OR "service network" OR "service networks" OR "transition of care" OR "transitional care" OR "transmural care" OR "whole system thinking" OR "holistic care")

#7 ("Angola " OR "Benin " OR "Botswana " OR "Burkina Faso" OR "Upper Volta" OR "Burundi " OR "Cameroon " OR "Cameroons " OR "Cape Verde" OR "Central African Republic" OR Chad OR "Comoros " OR "Comoro Islands" OR "Comores" OR "Mayotte " OR "Congo " OR "Zaire " OR "Cote d'Ivoire" OR "Ivory Coast" OR "Democratic Republic of the Congo" OR "Djibouti " OR "French Somaliland" OR "Eritrea " OR "Ethiopia " OR "Gabon " OR "Gabonese Republic" OR "Gambia " OR "Ghana " OR "Gold Coast" OR "Guinea " OR "Kenya " OR "Lesotho" OR "Basutoland" OR "Liberia " OR "Madagascar " OR "Malagasy Republic " OR "Malawi " OR "Nyasaland " OR "Mali " OR "Mauritania " OR "Mauritius " OR "Mozambique" OR "Namibia" OR "Niger" OR "Nigeria" OR "Rwanda" OR "Sao Tome" OR "Seychelles" OR "Senegal " OR "Sierra Leone" OR "Somalia " OR "South Africa" OR "Sudan " OR "Eswatini" OR "Tanzania" OR "Togo " OR "Togolese Republic" OR "Uganda" OR "Zambia" OR "Zimbabwe " OR "Rhodesia" OR "Sub-Saharan Africa" OR "Africa South of the Sahara")

#8 ((S1 OR S2 OR S3 OR S4 OR S5) AND S6 AND S7) & 1999-2022 & research article& peer-reviewed

For peer review only - http://bmjopen.bmj.com/site/about/guidelines.xhtml

Online supplementary file 3: Sensitivity analysis of RCTs and cluster RCTs

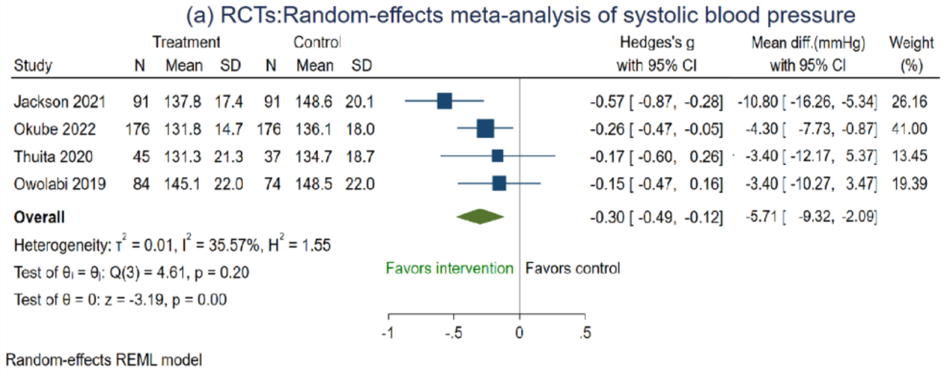

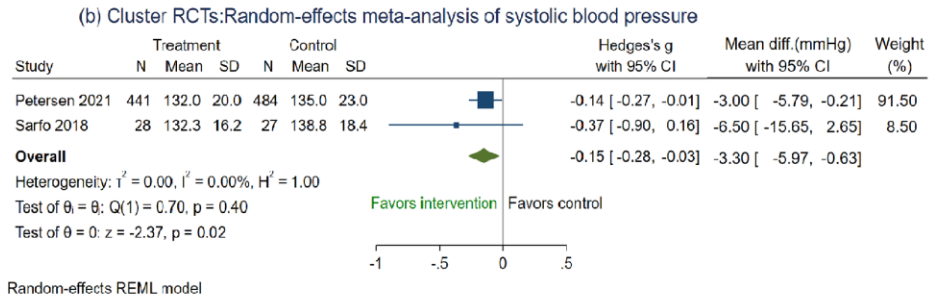

For peer review only - http://bmjopen.bmj.com/site/about/guidelines.xhtml

Online supplementary file 4: DOI plot of the random effects meta-analysis of SBP for integrated versus standard care for cardiometabolic multimorbidity in Sub-Saharan Africa

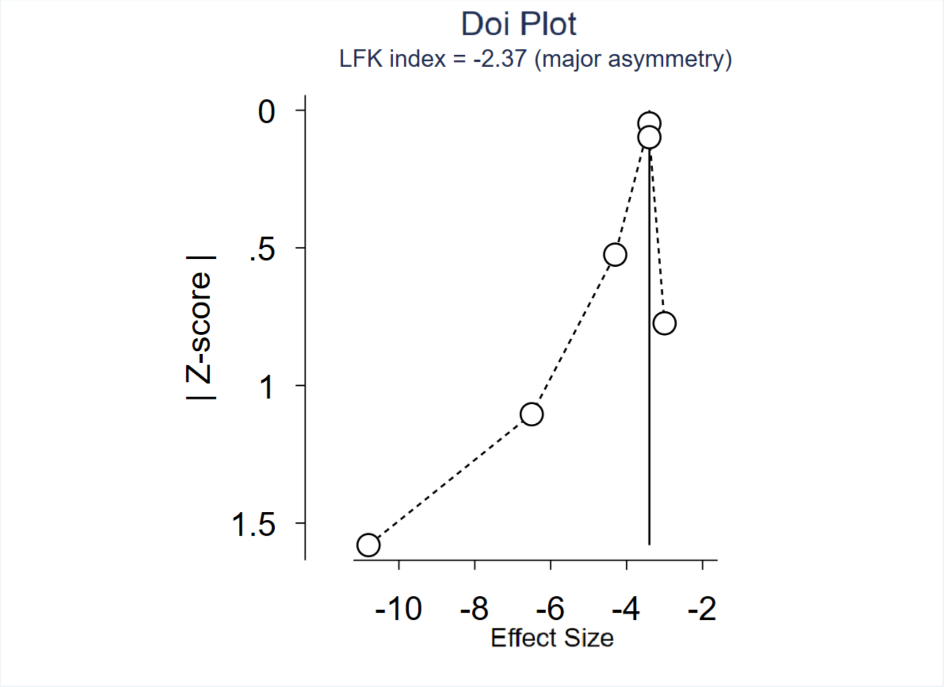

review only

For peer review only - http://bmjopen.bmj.com/site/about/guidelines.xhtml

PRISMA 2020 Checklist

Section and Topic	Item #	Checklist item	Location (Page) where item is reported
**TITLE : Effectiveness of integrated chronic care models for cardiometabolic multimorbidity in Sub-Saharan Africa: A systematic review and meta-analysis**			
Title	1	Identify the report as a systematic review.	1
**ABSTRACT**			
Abstract	2	See the PRISMA 2020 for Abstracts checklist.	2
**INTRODUCTION**			
Rationale	3	Describe the rationale for the review in the context of existing knowledge.	4
Objectives	4	Provide an explicit statement of the objective(s) or question(s) the review addresses.	5
**METHODS**			
Eligibility criteria	5	Specify the inclusion and exclusion criteria for the review and how studies were grouped for the syntheses.	7
Information sources	6	Specify all databases, registers, websites, organisations, reference lists and other sources searched or consulted to identify studies. Specify the date when each source was last searched or consulted.	7
Search strategy	7	Present the full search strategies for all databases, registers and websites, including any filters and limits used.	7
Selection process	8	Specify the methods used to decide whether a study met the inclusion criteria of the review, including how many reviewers screened each record and each report retrieved, whether they worked independently, and if applicable, details of automation tools used in the process.	8
Data collection process	9	Specify the methods used to collect data from reports, including how many reviewers collected data from each report, whether they worked independently, any processes for obtaining or confirming data from study investigators, and if applicable, details of automation tools used in the process.	8
Data items	10a	List and define all outcomes for which data were sought. Specify whether all results that were compatible with each outcome domain in each study were sought (e.g. for all measures, time points, analyses), and if not, the methods used to decide which results to collect.	7
	10b	List and define all other variables for which data were sought (e.g. participant and intervention characteristics, funding sources). Describe any assumptions made about any missing or unclear information.	7
Study risk of bias assessment	11	Specify the methods used to assess risk of bias in the included studies, including details of the tool(s) used, how many reviewers assessed each study and whether they worked independently, and if applicable, details of automation tools used in the process.	8
Effect measures	12	Specify for each outcome the effect measure(s) (e.g. risk ratio, mean difference) used in the synthesis or presentation of results.	9
Synthesis methods	13a	Describe the processes used to decide which studies were eligible for each synthesis (e.g. tabulating the study intervention characteristics and comparing against the planned groups for each synthesis (item #5)).	9
	13b	Describe any methods required to prepare the data for presentation or synthesis, such as handling of missing summary statistics, or data conversions.	9
	13c	Describe any methods used to tabulate or visually display results of individual studies and syntheses.	9
	13d	Describe any methods used to synthesize results and provide a rationale for the choice(s). If meta-analysis was performed, describe the model(s), method(s) to identify the presence and extent of statistical heterogeneity, and software package(s) used.	9
	13e	Describe any methods used to explore possible causes of heterogeneity among study results (e.g. subgroup analysis, meta-regression).	N/A
	13f	Describe any sensitivity analyses conducted to assess robustness of the synthesized results.	9
Reporting bias	14	Describe any methods used to assess risk of bias due to missing results in a synthesis (arising from reporting biases).	9

For peer review only

PRISMA 2020 Checklist

Section and Topic	Item #	Checklist item	Location (Page) where item is reported
assessment			
Certainty assessment	15	Describe any methods used to assess certainty (or confidence) in the body of evidence for an outcome.	9
**RESULTS**			
Study selection	16a	Describe the results of the search and selection process, from the number of records identified in the search to the number of studies included in the review, ideally using a flow diagram.	10
	16b	Cite studies that might appear to meet the inclusion criteria, but which were excluded, and explain why they were excluded.	10
Study characteristics	17	Cite each included study and present its characteristics.	10
Risk of bias in studies	18	Present assessments of risk of bias for each included study.	12
Results of individual studies	19	For all outcomes, present, for each study: (a) summary statistics for each group (where appropriate) and (b) an effect estimate and its precision (e.g. confidence/credible interval), ideally using structured tables or plots.	14
Results of syntheses	20a	For each synthesis, briefly summarise the characteristics and risk of bias among contributing studies.	12
	20b	Present results of all statistical syntheses conducted. If meta-analysis was done, present for each the summary estimate and its precision (e.g. confidence/credible interval) and measures of statistical heterogeneity. If comparing groups, describe the direction of the effect.	14
	20c	Present results of all investigations of possible causes of heterogeneity among study results.	14
	20d	Present results of all sensitivity analyses conducted to assess the robustness of the synthesized results.	14
Reporting biases	21	Present assessments of risk of bias due to missing results (arising from reporting biases) for each synthesis assessed.	14
Certainty of evidence	22	Present assessments of certainty (or confidence) in the body of evidence for each outcome assessed.	14
**DISCUSSION**			
Discussion	23a	Provide a general interpretation of the results in the context of other evidence.	15-16
	23b	Discuss any limitations of the evidence included in the review.	16
	23c	Discuss any limitations of the review processes used.	16
	23d	Discuss implications of the results for practice, policy, and future research.	17
**OTHER INFORMATION**			
Registration and protocol	24a	Provide registration information for the review, including register name and registration number, or state that the review was not registered.	5
	24b	Indicate where the review protocol can be accessed, or state that a protocol was not prepared.	The protocol was not published
	24c	Describe and explain any amendments to information provided at registration or in the protocol.	N/A
Support	25	Describe sources of financial or non-financial support for the review, and the role of the funders or sponsors in the review.	17
Competing interests	26	Declare any competing interests of review authors.	17-18

For peer review only

PRISMA 2020 Checklist

Section and Topic	Item #	Checklist item	Location (Page) where item is reported
Availability of data, code and other materials	27	Report which of the following are publicly available and where they can be found: template data collection forms; data extracted from included studies; data used for all analyses; analytic code; any other materials used in the review.	17-18

From: Page MJ, McKenzie JE, Bossuyt PM, Boutron I, Hoffmann TC, Mulrow CD, et al. The PRISMA 2020 statement: an updated guideline for reporting systematic reviews. BMJ 2021;372:n71. doi: 10.1136/bmj.n71

For more information, visit: http://www.prisma-statement.org/

For peer review only
